# Integrated bulk and single-cell RNA-sequencing reveals *SPOCK2* as a novel biomarker gene in the development of congenital pulmonary airway malformation

**DOI:** 10.1186/s12931-023-02436-z

**Published:** 2023-05-10

**Authors:** Zheng Tan, Fengxia Li, Qiang Chen, Hongyu Chen, Ziru Xue, Jian Zhang, Yue Gao, Liang Liang, Ting Huang, Shouhua Zhang, Jianhua Li, Qiang Shu, Lan Yu

**Affiliations:** 1 Department of Paediatric Thoracic Surgery, Children’s Hospital, Zhejiang University School of Medicine, National Clinical Research Center for Child Health, Hangzhou, Zhejiang China; 2 Children’s Hospital, Zhejiang University School of Medicine, National Clinical Research Center for Child Health, Hangzhou, Zhejiang China; 3 Department of Pediatrics, Jiangxi Provincial Children’s Hospital, Jiangxi, China; 4 Department of Molecular Genetics, University of Toronto, Toronto, ON Canada; 5 Department of General Surgery, Jiangxi Provincial Children’s Hospital, Jiangxi, China

**Keywords:** Congenital pulmonary airway malformation, Bulk RNA sequencing, Single-cell RNA sequencing, iWGCNA, *SPOCK2*, *STX11*, *ZNF331*

## Abstract

**Background:**

Congenital pulmonary airway malformation (CPAM) is the most frequent pulmonary developmental malformation and the pathophysiology remains poorly understood. This study aimed to identify the characteristic gene expression patterns and the marker genes essential to CPAM.

**Methods:**

Tissues from the cystic area displaying CPAM and the area of normal appearance were obtained during surgery. Bulk RNA sequencing (RNA-seq) and single-cell RNA sequencing (scRNA-seq) were performed for integrating analysis. Iterative weighted gene correlation network analysis (iWGCNA) was used to identify specifically expressed genes to CPAM.

**Results:**

In total, 2074 genes were significantly differentially expressed between the CPAM and control areas. Of these differentially expressed genes (DEGs), 1675 genes were up-regulated and 399 genes were down-regulated. Gene ontology analysis revealed these DEGs were specifically enriched in ciliated epithelium and involved in immune response. We also identified several CPAM-related modules by iWGCNA, among them, P15_I4_M3 module was the most influential module for distinguishing CPAMs from controls. By combining the analysis of the expression dataset from RNA-seq and scRNA-seq, *SPOCK2, STX11*, and *ZNF331* were highlighted in CPAM.

**Conclusions:**

Through our analysis of expression datasets from both scRNA-seq and bulk RNA-seq of tissues obtained from patients with CPAM, we identified the characteristic gene expression patterns associated with the condition. Our findings suggest that *SPOCK2* could be a potential biomarker gene for the diagnosis and therapeutic target in the development of CPAM, whereas *STX11* and *ZNF331* might serve as prognostic markers for this condition. Further investigations with larger samples and function studies are necessary to confirm the involvement of these genes in CPAM.

**Supplementary Information:**

The online version contains supplementary material available at 10.1186/s12931-023-02436-z.

## Background

Congenital pulmonary airway malformation (CPAM) is a rare pulmonary developmental malformation characterized by the presence of multiple cysts within lung parenchyma due to excessive proliferation and expansion of terminal bronchioles [1]. The incidence of CPAM is 0.94 per 10,000 to 4 per 10,000 births over the last years [2, 3], accounting for about 25% of all congenital lung lesions. The majority of CPAMs can be antenatally diagnosed by ultrasound screening and persist postnatally. The clinical manifestations are variable, which can be asymptomatic or be with respiratory distress or acute infection after birth. Surgical resection is the primary treatment for CPAM due to the risk of recurrent pneumonia and concern for malignant transformation [4–6].

The pathogenesis of CPAM is still unclear. It is thought to be the disturbance of the interaction between the epithelium and the underlying mesenchyme during lung development, resulting in an overgrowth of mesenchymal cells and decreased apoptosis, consequently forming the multicystic lung mass [1, 7]. Altered expression of factors such as fibroblast growth factors [8, 9], TGF-beta signaling [10], or transcription factors [11–13] were observed in the epithelial cells of the cysts in CPAM, indicating the involvement of these genes in CPAM.

However, because of the complex pathophysiology and cell heterogeneity in lung tissues, a comprehensive profile of mRNA expression in different cell types of CPAM is necessary [14]. Bulk mRNA analysis measures the average gene expression levels in a population of cells and may not reflect the complexity and heterogeneity of cells of disease. Single-cell RNA sequencing (scRNA-seq) technology facilitate the dissection of the hidden heterogeneity in cell populations [15] and offer opportunities to transfer gene signature from scRNA-seq to bulk data [16]. Here in this study, we performed an integrated analysis of scRNA-seq and bulk RNA-seq of tissues from patients with CPAM to identify the characteristic gene expression patterns and the marker genes essential to CPAM.

## Methods

### Subjects

Patients with CPAM were recruited from the Children’s Hospital, Zhejiang University School of Medicine. All patients included for analysis were excluded with infection at the time of surgery. The diagnosis and the classification of CPAM was confirmed by histological analysis after surgical resection according to the Stocker classification [17]. For each CPAM patient, we collected tissues from the cystic area and the area of normal appearance surrounding the lesion as a simply control. The samples were snap-frozen and kept in liquid nitrogen for storage at -80 ℃ before RNA extraction.

### RNA sequencing

Total RNA was extracted from frozen lung tissues of CPAM using TRIzolR Reagent (Invitrogen). Four micrograms of total RNA were used for cDNA sequencing library generation using NEBNext UltraTM RNA Library Prep Kit (NEB, USA). The purified products were evaluated with an Agilent Bioanalyzer (Agilent Technologies). Eligible libraries were sequenced on Illumina HiSeq 2000 platform 100-bp paired-end reads.

The original fastq data were evaluated and Low-quality reads were filtered by the Trimmomatic tool [18]. The filtered clean reads were aligned to the human reference genome (GRCh38) using Hisat2[19]. Only protein-coding genes were kept for analysis and genes with no mapped reads in at least half of the samples were filtered out. Gene expression levels were quantified with raw counts (FPKM) by StringTie [20].

### Differential expression analysis

Differentially expressed genes (DEGs) between cysts and simply controls were identified using the DESeq2 package in R [21]. DEGs were selected based on the thresholds of both the *P* value < 0.01 and fold change (FC) > 2. DEGs were visualized by volcano plot. A principal component analysis (PCA) was performed to detect the overall differences between the individual samples using the normalized counts of all genes.

### Functional enrichment and protein-protein interaction network analysis

To assess the functions of the DEGs identified in CPAM, Gene Ontology (GO) enrichment analysis was performed using the clusterProfiler package [22] with the up-regulated genes and down-regulated genes, respectively. GO terms with FDR < 0.05 were considered a significant event.

Protein-Protein Interaction (PPI) data were downloaded from the STRING database to investigate protein interactions among the DEGs enriched in the significant GO terms, the interactions with a PPI score > 500 were retained for further analysis, and the PPI network was visualized by Cytoscape software [23, 24].

### Discovering the influential gene modules in distinguishing CPAM from simply control

Iterative weighted gene correlation network analysis (iWGCNA) (https://github.com/cstoeckert/iterativeWGCNA), which is a gene network analysis based on correlation to identify highly co-expressed clusters of genes (modules) within whole-transcriptome datasets, was performed to identify specifically expressed genes to CPAM. Genes were grouped into modules and ordered by randomForest package and the mean decrease Gini index in iWGCNA. For each gene module, the difference of eigengenes between CPAMs and simply controls was compared by Wilcoxon test. The correlation coefficients between each module eigengene and clinical characteristics were also calculated.

### Single-cell RNA-seq (scRNA-seq) data analysis

scRNA-seq data was obtained from Zhang’s published paper, and was analyzed with Seurat (https://github.com/satijalab/seurat) [25]. Cells with < 200 or > 2500 genes and mitochondrial gene fragments > 20% were filtered. The remaining cells were merged into one gene expression count matrix, and the count data were normalized and scaled using Seurat’s functions of NormalizeData() and ScaleData(). Dimension reduction and clusters identification of cells were implemented by RunTSNE() and Findclusters() functions. Furthermore, marker genes of each cluster (defined as expressed in more than 25% of cells in each cluster) were identified by the FindAllMarkers() function and different cell clusters were annotated by the singleR package [26].

In each cluster, we calculated the average Z-score of marker genes and we defined these values as the cluster signatures. Differences in cluster signature between CPAM and simply control were tested by the Wilcoxon test. *P*-value threshold was set as 0.05.

### Real-time PCR

We performed experimental validation of the expression of *SPOCK2* in another 6 cDNA samples of CPAM and simply controls by using quantitative PCR (qPCR). The PCR primers were designed for the coding region of *SPOCK2* and synthesized by Ykang (Hangzhou, China). All qPCR reactions were performed in a total of 10 mL volume, comprising 5 mL 2× SYBR Green I Master Mix (Promega), 1 mL 10 nM of each primer, and 2 mL of 1:20 diluted cDNA in 96-well plates with QuantStudio™ 7 Flex (Applied Biosystems). All reactions were performed in triplicate, and the conditions were 5 min at 95 °C and then 40 cycles of 95 °C at 15 s and 60 °C at 30 s. The relative expression was calculated via the standard curve method relative to the GAPDH housekeeping gene. We used five-serial 4-fold dilutions of cDNA samples to construct the standard curves for *SPOCK2* and *GAPDH*.

## Results

### Altered gene expression during the development of CPAM in RNA-seq

**Fig. 1:**
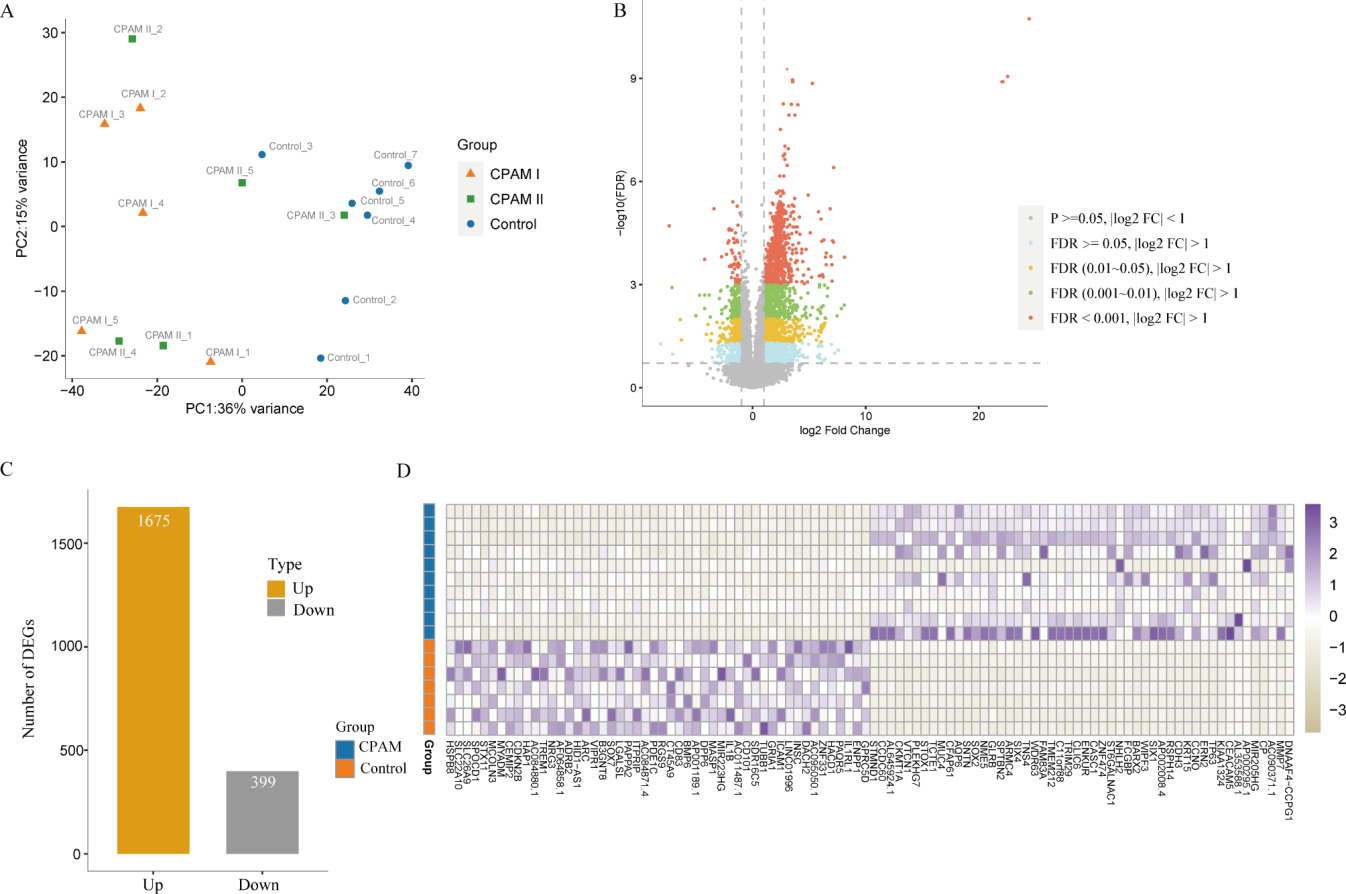
**Identification of DEGs between CPAM and control samples by transcriptome sequencings**. (**A**) Discrimination of CPAM to control by Principal Component Analysis (PCA) in RNA-seq expression profiles. Samples of CPAM and control were colored in blue and orange dots, respectively. (**B**) Volcano plot representation of DEGs between CPAM and control. Each point in the plot indicated one gene, vertical lines refer to 2-fold change and the horizontal line corresponds to *P*-value of 0.05. (**C**) The number of significantly up-regulated and down-regulated genes (*FDR* < 0.05 and a log2 FC > 1 or log2 FC < -1) were illustrated by yellow and gray bars, respectively. (**D**) Heatmap of the most DEGs in CPAM and control samples. Top 50 up-regulated or down-regulated genes between CPAM and control were shown

Five patients with type 1 and five patients with type 2 CPAM were recruited and their clinical characteristics were shown in Additional file 1: Table S1. The median age at surgery of the seven patients was 9 months. A total of 380 million reads were generated from the 17 transcriptome sequencing data. On average, 97% of reads were mapped to the human reference genome. PCA of gene expression profile showed that CPAM samples apparently separated from the simply controls (Fig. 1A). Additionally, we compared the expression of type 1 CPAM and type 2 CPAM to identify the DEGs. Our analysis revealed a total of 4 upregulated genes and 9 downregulated genes between the two types (Additional file 7: Fig S1). We thus consolidated all the CPAM samples to compare them against the control group. We identified 2074 DEGs between CPAM areas and the paired control areas (Fig. 1B). Of these, 1675 DEGs were up-regulated and 399 DEGs were down-regulated (Fig. 1C, Additional file 2: Table S2). The heatmap of the top 50 DEGs with up-regulated and 50 DEGs with down-regulated were shown in Fig. 1D.

### Gene ontology analysis and key network identification of DEGs

**Fig. 2:**
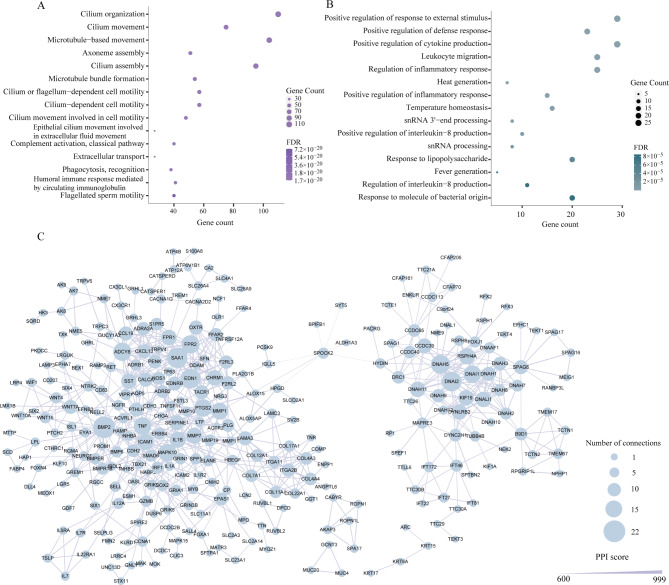
**Functional analysis of differentially expressed genes.** (**A, B**) Gene Ontology (GO) enrichment of up-regulated and down-regulated genes in CPAMs and controls, only the top 15 significant terms were displayed. The size of the dot indicates the value of FDR, and the color of the dot indicates the number of up or down regulated genes enriched in that pathway. (**C**) PPI network of DEGs. The nodes represent genes. The size of nodes indicates the number of connections. The edges denote the interactions between two genes, and the width of an edge denotes the score of the interaction

GO analysis showed that the up-regulated genes were markedly enriched in some biological processes such as cilium organization (FDR = 1.80 × 10^− 49^), cilium movement (FDR = 8.49 × 10^− 45^), epithelial cilium movement involved in extracellular fluid movement (FDR = 4.94 × 10^− 23^), phagocytosis, recognition (FDR = 5.14 × 10^− 21^) and humoral immune response mediated by circulating immunoglobulin (FDR = 5.85 × 10^− 21^) (Fig. 2A, Additional file 3: Table S3a). Down-regulated genes were mainly enriched in the biological processes of positive regulation of response to external stimulus (FDR = 8.38 × 10 − 8), positive regulation of defense response or cytokine production (FDR = 1.28 × 10 − 7, FDR = 1.64 × 10 − 7), leukocyte migration (FDR = 6.61 × 10 − 7), and regulation of inflammatory response (FDR = 8.99 × 10 − 7) (FDR = 1.12 × 10^− 4^) (Fig. 2B, Additional file 3: Table S3b). The GO enrichment based on molecular function also confirmed the DEGs were mainly involved in microtubule motor and immune factor activities (Additional file 4: Table S4). Genes were significantly associated with the components of cilium and granule epithelial cells (Additional file 5: Table S5).

A total of 747 DEGs were filtered into the PPI network with the PPI score ranging from 500 to 999, containing 492 nodes and 765 edges (Fig. 2C). Among them, *BMP2*, *TNF*, *DNAI1*, *DNAI2*, and *DNAH5*, have a relatively higher degree and betweenness centrality, which interacted strongly with other genes.

### Refinement of influential gene modules identifies CPAM-associated transcriptomic signals

**Fig. 3:**
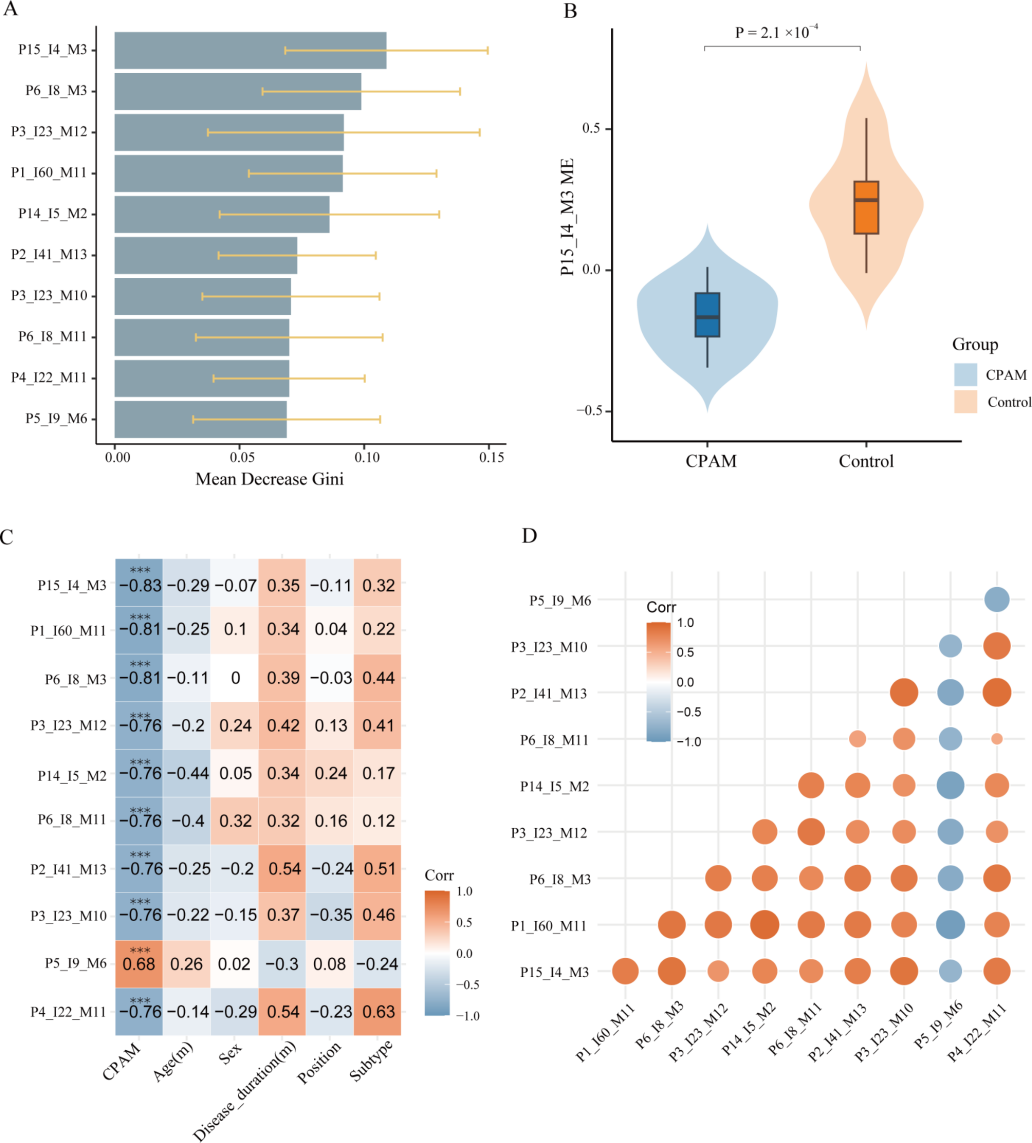
**Determining gene modules that are influential in distinguishing CPAM from control.** (**A**) Top 10 modules were listed according to the order of mean decrease Gini index for the discrimination of CPAMs. Error bars represent standard deviations of declines in the Gini index, which were calculated 100 times in the analysis. (**B**) P15_I4_M3 module eigengene (ME) in samples of CPAM and control. (Wilcoxon test, *P* = 2.1 × 10^− 4^). (**C**) Correlation between top 10 eigengene module expression and clinical parameters. Labels in each box indicate correlation coefficients, only *P* values that are significant are displayed by *. ‘***’ represented the *P* < 0.001, while ‘**’ represented the *P* < 0.01. (**D**) Correlation network of top 10 modules. The size and color of the dots show the Pearson’s correlation coefficient

A total of 171 modules were identified and ordered by iWGCNA. The top 10 crucial modules to CPAM were listed in Fig. 3A (Additional file 6: Table S6). The eigengene of the P15_I4_M3 module (P15_I4_M3 ME) was specifically lower in CPAMs than in simply controls (Fig. 3B, *P* = 2.1 × 10^− 4^).

In addition, we investigated the correlation between the top 10 ranked gene modules and the clinical characteristics of subjects (Fig. 3C). Intriguingly, all of the modules mentioned above significantly correlated with the existence of CPAM. As a consequence, the top 10 gene modules for distinguishing CPAMs from simply controls were defined as the ‘CPAM-related module’. Furthermore, the correlation coefficient of modules P15_I4_M3, P1_I60_M11, and P6_I8_M3 are greater than 0.8. We thus used these three modules for further integrative analysis. However, these modules were independent of age, sex, disease duration, site of pathological position, and subtype. Most importantly, by inter-modular correlation analysis, these highly ranked modules strongly correlated with each other and consisted a cluster on the network (Fig. 3D).

### Integrative analysis of scRNA-seq data from CPAM patient

**Fig. 4:**
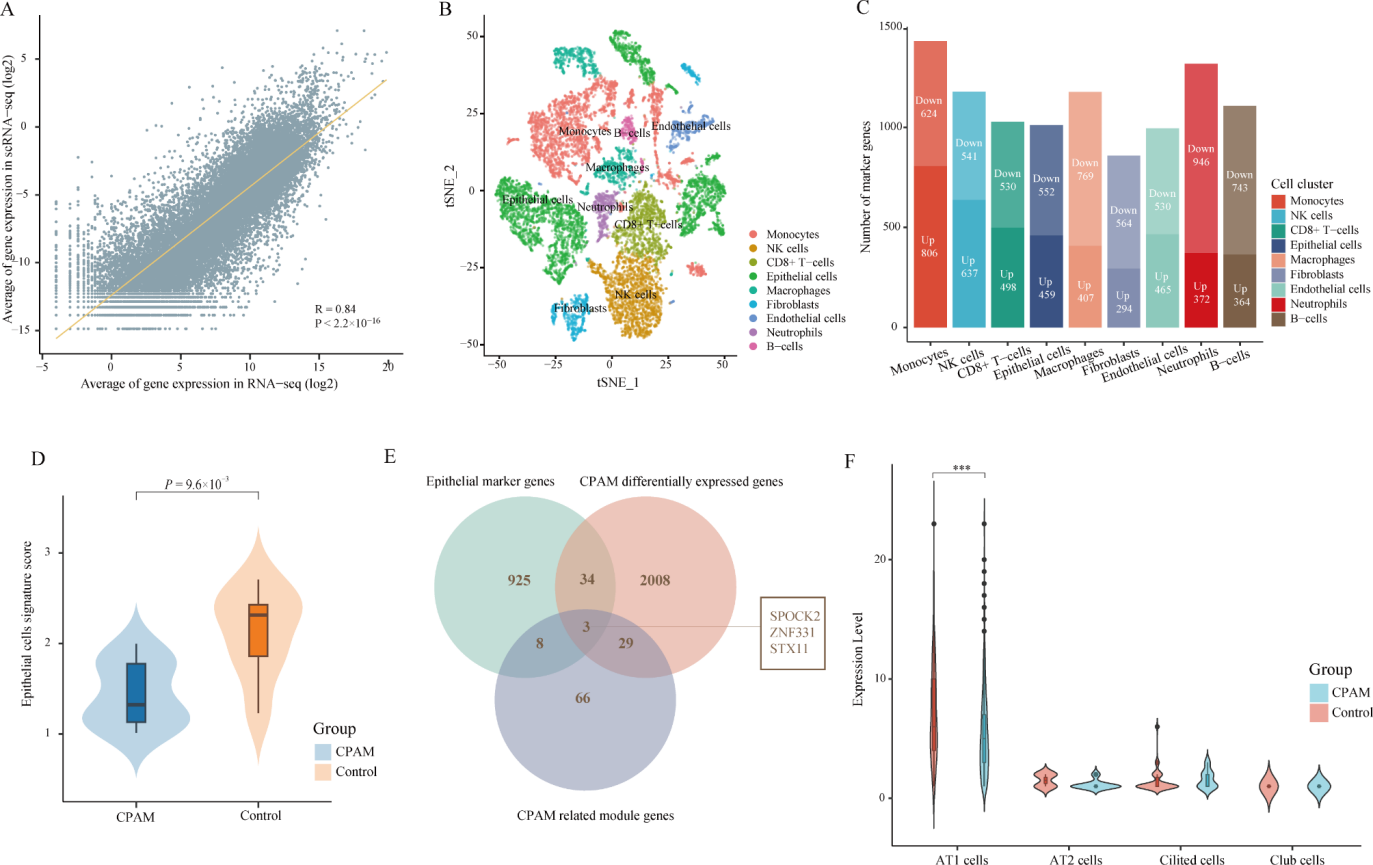
**scRNA-seq analysis of CPAM and its overlap with bulk RNA-seq analysis.** (**A**) Gene expression profiles from bulk RNA-seq and scRNA-seq were averaged and plotted on x and y axes, respectively. Correlation coefficient was calculated by Pearson’s test (R = 0.84, *P* < 2.2 × 10^− 16^). (**B**) t-SNE plot of cells from CPAM indicates distinct clusters predominantly determined by cell type. (**C**) The number of marker gene in 9 clusters. (**D**) Difference between samples of CPAM and control in epithelial cell signature score. (**E**) Venn diagram shows the intersection of epithelial cell marker genes, CPAM differentially expressed genes and iWGCNA CPAM-related module genes. (**F**) Expression levels of *SPOCK2* in proliferate subtypes of epithelial cells. ‘***’ represented the *P* < 0.001. AT1, alveolar type 1 cells; AT2, alveolar type 2 cells

To validate the completeness of our RNA-seq data and investigate more specific details of CPAM, we performed the integrative analysis with CPAM scRNA-seq data. We observed a great consistency between bulk RNA-seq data and scRNA-seq data of CPAM in gene expression (Fig. 4A, correlation coefficient (R) = 0.84, *P* < 2.2 × 10^− 16^). After quality control, a total of 14,814 cells were used for downstream analysis. Unsupervised clustering analysis displayed that these cells were divided into 26 distinct clusters corresponding to 9 cell types, including Monocytes, Epithelial cells, B-cells, CD8 + T-cells, and Endothelial cells, Fibroblasts, Macrophages, Neutrophils, and NK cells (Fig. 4B). Differentially gene expression analysis was utilized to determine cell type specific marker genes. We identified 1430 marker genes in monocytes, 1176 marker genes in macrophages, 995 marker genes in endothelial cells, and 981 marker genes in epithelial cells (Fig. 4C).

Epithelial cells were the main cell type of bronchi and alveoli. So, we focused on epithelial cells for further analysis. We found the signature score of epithelial cells was significantly lower in cystic areas than in control areas of CPAM (Fig. 4D, *P* = 9.6 × 10^− 3^). When we compared the genes from the three methods, we found 32 overlaps between the DEGs and the CPAM-related module genes, 37 gene-overlaps between the DEGs and the marker genes of epithelial cells, and 11 overlapping genes between the marker genes of epithelial cells and the CPAM-related module genes (Fig. 4E). By integrating all the data, we found that *SPOCK2, ZNF331*, and *STX11* were the only overlapped genes (Fig. 4E). All three genes were with decreased expression in cystic area (Additional file 8, Fig S2).

The qPCR validated the decreased expression of *SPOCK2* in CPAM tissues (Additional file 9: Fig. S3). We further investigated the expression of *SPOCK2* in epithelial subtypes of ciliated cells, alveolar type 1 (AT1), alveolar type 2 (AT2), club cells, and basal cells based on the scRNA-seq data [27]. No expression was detected in basal cells, but a relatively higher expression of *SPOCK2* was found in AT1 cells than in other cell types. Furthermore, *SPOCK2* expression was significantly decreased in CPAM sample compared to the simply control sample in AT1 cell type (Fig. 4F, *P* = 4.87 × 10^− 8^).

## Discussion

In this work, we identified a transcriptomic change in 10 cystic areas and their paired control areas of CPAM patients. DEGs between CPAM and simply control tissues were observed in previous transcriptomic studies [10, 28]. Decreased expression of fatty acid binding protein-7 (*FABP-7*) at both RNA and protein levels was found in fetal CPAM tissues compared with fetal control lungs based on a microarray analysis. However, in the same study, although *FABP-7* expression was underexpressed, no significant difference was investigated between postnatal CPAMs and simply controls [28], which is consistent with our results. It is not supervised since *FABP-7* was expressed in mesenchymal cells of the fetal lung at gestation but not in postnatal or adult lung tissues [28]. All of our samples are postnatal lung tissues. In another transcriptomic analysis of the epithelium of macrocytic lung malformations, *TGFBR1* and *TGFB2* were indicated for CPAM pathogenesis [10]. However, we didn’t observe the dysregulation of TGF beta signaling in our epithelial cells. The heterogeneous of samples used between these two studies may explain the conflict results.

Abnormal epithelial cell differentiation was indicated during lung development and resulted in the formation of lung mass [1]. Consistent with that, both our GO analysis and the results from a previous transcriptome study suggested the top upregulated transcripts in CPAM were highly enriched in airway epithelium, specifically in ciliated epithelium [29]. Furthermore, the strongest interacted genes from our PPI data were primary ciliary dyskinesia-related genes *DNAI1, DNAI2*, and *DNAH5* [30], suggesting the role of cilia in CPAM. Evidence showed that primary cilia could transduce and regulate sonic hedgehog (SHH) signaling which was required for embryonic lung development [31]. While deregulated SHH signaling was thought to be the pathogenesis of lung cystic abnormalities [32]. Of note, pseudotime trajectory analysis from scRNA-seq revealed that epithelial subtypes of ciliated cells, AT1 and AT2 originated from the club and basal cells and were at the end of pseudotime [27]. Our CPAM tissues and their paired simply controls were with the postnatal collection. Therefore, the DEGs enriched in ciliated epithelium from our bulk RNA-seq data might be the biomarkers of the consequence of the initial molecular dysregulation, rather than the triggering event in the lung cysts development [10]. More mechanism studies are necessary to discover the pathogenesis of CPAM. The DEGs were also significantly enriched in immune-related functions and inflammatory responses, suggesting the importance of the inflammatory process in CPAM. Indeed, inflammation is not rare in patients with CPAM, even in asymptomatic babies [33]. Histologic signs of inflammation or inflammation cells could be detected as an early feature of CPAM in the first few days of life without infection [34]. Our enrichment of DEGs in inflammatory response confirmed that malformation of the lung rather than infection induced the inflammatory reaction [34].

By integrating the datasets from bulk RNAseq, scRNA-seq, and iWGCNA, we highlighted *SPOCK2, STX11*, and *ZNF331* as candidate genes for CPAM. *SPOCK2* is one member of the testican group of extracellular chondroitin and heparin sulfate proteoglycans (HSPG). It is expressed in lung development and significantly increased during alveolarization [35]. Decreased expression of *SPOCK2* in epithelial cells may interrupt the balance of mesenchymal-epithelial interaction during alveolarization [36], consequently resulting in lung malformation. An in vitro study suggested a high expression and regulation role of *SPOCK2* in the transdifferentiation from AT2 to AT1 [37], consistent with our scRNA-seq data of higher expression of *SPOCK2* in AT1 cells. Upregulation of *SPOCK2* can decrease the matrix metalloproteinases (MMPs) expression and activation [38], while MMPs are known as zinc-dependent proteolytic enzymes to regulate extracellular matrix (ECM) turnover and involve in various pulmonary pathologies [39]. A recent study showed higher activity of *MMP-9* in mice models with CPAM [40]. Our data suggested a significant decreased expression of *SPOCK2* in CPAM especially in AT1 cells. All of the evidence indicates the decreased expression of *SPOCK2* may contribute to the activation of MMP-9 in CPAM, suggesting a possible role of *SPOCK2* in the development of CPAM. *SPOCK2* could be a potential early diagnostic marker by detecting its expression, and therapeutic target for the preventing the pulmonary pathologies involving MMPs through up-regulating the expression.

*STX11* is expressed at lower levels in the cystic area of CPAM and is known to be involved in intracellular vesicle trafficking and fusion. While current literatures do not show a clear correlation between *STX11* and lung development. But it is predominantly expressed in tissues of immune system [41]. Patients with mutated *STX11* gene result in loss of immune homeostasis and severe phenotypes of hyperinflammation, such as prolonged fever, hepatosplenomegaly, and hemophagocytosis [42]. As patients with CPAM are at high risk of persistent cough or recurrent lung infection [43], GO enrichment of DEG also show the dysregulation of inflammation in CPAM. These findings suggest the possibility of involvement of *STX11* in immune regulation in CPAM. It could be a possible therapeutic target to release the high risk of infections in CPAM patients. However, further experiments are needed to investigate the immune functions of *STX11* in CPAM.

*ZNF331* is a zinc finger transcriptional repressor [44]. Studies have shown that the promoter region of *ZNF331* is frequently methylated and serve as a poor prognostic marker for several types of cancers [45, 46]. While there is limited data on the association of *ZNF331* and lung development, one of the most concerns with CPAM is the risk of malignant transformation [47]. Therefore, it would be valuable to investigate the methylation status of *ZNF331* in cystic area of CPAM to see if it could monitor the progression of CPAM towards malignancy.

## Conclusions

In summary, we performed a global transcriptome analysis by RNA-sequencing and obtained a bunch of differently expressed genes and gene modules that may be essential for the development of CPAM. By integrating the analysis of the expression datasets from RNA-seq and scRNA-seq, we have identified *SPOCK2, STX11*, and *ZNF331* as promising candidate genes for further investigation. These genes may have potential diagnostic, therapeutic, prognostic biomarkers for CPAM. However, the small sample size and lack of detailed analysis of disease type limit our in-depth exploration of the etiology of CPAM. Replications and function assays are required to elucidate the molecular mechanisms of *SPOCK2* in CPAM.

## Electronic supplementary material

Below is the link to the electronic supplementary material.

**Additional file 1: table S1** Clinical characteristics of subjects

**Additional file 2: table S2** DEGs between CPAM and simply control areas

**Additional file3: table S3** GO enrichment analysis (biological process, BP) of up-regulated and down-regulated genes

**Additional file 4: table S4** GO enrichment analysis (molecular function, MF) of up-regulated and down-regulated genes

**Additional file 5: table S5** GO enrichment analysis (cellular component, CC) of up-regulated and down-regulated genes

**Additional file 6: table S6** Gene list for the top 10 modules performed by iWGCNA

**Additional file 7: figure S1** The numbers of DEGs in different groups

**Additional file 8: figure S2** Expressions of *SPOCK2*, *ZNF331* and *STX11* in RNA-seq data

**Additional file 9: figure S3** Validation of SPOCK2 by qPCR analysis in samples of 6 CPAMs and 6 simply controls

## Data Availability

The data that support the findings of this study are available from the corresponding author upon reasonable request.

## Abbreviations

CPAM: Congenital pulmonary airway malformation
RNA-seq: RNA-sequencing
scRNA-seq: Single-cell RNA-sequencing
iWGCNA: Iterative weighted gene correlation network analysis
DEG: Differentially expressed gene
FC: Fold change
PCA: Principal component analysis
GO: Gene Ontology
PPI: Protein-Protein Interaction
ME: Module eigengene
AT1: Alveolar type 1
AT2: Alveolar type 2
