# Supplementary material for: Integrated bulk and single-cell RNA-sequencing reveals SPOCK2 as a novel biomarker gene in the development of congenital pulmonary airway malformation
Source: Respir Res. 2023 May 10;24:127. doi: 10.1186/s12931-023-02436-z (PMC10170809; doi:10.1186/s12931-023-02436-z)
Supplement: Supplementary file 1 — Additional file 1: table S1 Clinical characteristics of subjects [file 12931_2023_2436_MOESM1_ESM.docx]

| **Table S1 Clinical characteristics of subjects** | | | | | |
| --- | --- | --- | --- | --- | --- |
| **Sample** | **Age at surgery**  **(m)** | **Sex** | **Disease duration (m)** | **Subtype** | **Site of pathological position** |
| CPAM I_1 | 7 | Male | 12 | I | Right lower lobe |
| CPAM I_2 | 7 | Male | 7 | I | Right lower lobe |
| CPAM I_3 | 44 | Female | 4 | I | Right lower lobe |
| CPAM I_4 | 11 | Female | 12 | I | Right lower lobe |
| CPAM I_5 | 7 | Male | 6 | I | Left upper lobe |
| CPAM II_1 | 9 | Male | 12 | II | Left lower lobe |
| CPAM II_2 | 16 | Female | 12 | II | Right upper lobe |
| CPAM II_3 | 7 | Female | 12 | II | Left upper and lower lobe |
| CPAM II_4 | 7 | Female | 12 | II | Right lower lobe |
| CPAM II_5 | 7 | Female | 12 | II | Right lower lobe |

Note: m: month; Disease duration: the time between the diagnosis and the surgery.
