# Supplementary material for: Integrated bulk and single-cell RNA-sequencing reveals SPOCK2 as a novel biomarker gene in the development of congenital pulmonary airway malformation
Source: Respir Res. 2023 May 10;24:127. doi: 10.1186/s12931-023-02436-z (PMC10170809; doi:10.1186/s12931-023-02436-z)
Supplement: Supplementary file 2 — Additional file 2: table S2 DEGs between CPAM and simply control areas [file 12931_2023_2436_MOESM2_ESM.docx]

**Table S2 DEGs between CPAM and simply control areas**

| **Gene Symbol** | **log2 Fold Change** | **lfcSE** | **stat** | **pvalue** | **padj** |
| --- | --- | --- | --- | --- | --- |
| DNAAF4-CCPG1 | 24.461812 | 3.0314212 | 8.0694205 | 7.06E-16 | 1.84E-11 |
| MMP7 | 3.0524414 | 0.4039614 | 7.5562705 | 4.15E-14 | 5.41E-10 |
| AC090371.1 | 22.560596 | 3.0324101 | 7.4398235 | 1.01E-13 | 8.76E-10 |
| CP | 3.5299316 | 0.4788322 | 7.3719591 | 1.68E-13 | 1.10E-09 |
| MIR205HG | 3.536758 | 0.4841875 | 7.304522 | 2.78E-13 | 1.25E-09 |
| AL353588.1 | 22.137681 | 3.0328858 | 7.2992135 | 2.89E-13 | 1.25E-09 |
| AP000295.1 | 22.071181 | 3.0318779 | 7.2797065 | 3.35E-13 | 1.25E-09 |
| CEACAM5 | 5.2912296 | 0.7302079 | 7.2461962 | 4.29E-13 | 1.40E-09 |
| KIAA1324 | 2.7087695 | 0.3847106 | 7.0410566 | 1.91E-12 | 5.53E-09 |
| TP63 | 3.4281021 | 0.4881503 | 7.0226359 | 2.18E-12 | 5.68E-09 |
| ERN2 | 4.0007242 | 0.571133 | 7.0048901 | 2.47E-12 | 5.86E-09 |
| CCNO | 3.208229 | 0.4660672 | 6.8836182 | 5.84E-12 | 1.17E-08 |
| KRT15 | 3.7369167 | 0.5419867 | 6.8948493 | 5.39E-12 | 1.17E-08 |
| CDH3 | 2.4551015 | 0.3645469 | 6.7346666 | 1.64E-11 | 3.06E-08 |
| RSPH14 | 2.8607945 | 0.4360913 | 6.5600821 | 5.38E-11 | 9.35E-08 |
| AP002008.4 | 3.1623591 | 0.4846748 | 6.5247031 | 6.81E-11 | 1.11E-07 |
| SIX1 | 2.8355398 | 0.4388045 | 6.4619665 | 1.03E-10 | 1.58E-07 |
| WIPF3 | 2.7020207 | 0.4199566 | 6.4340471 | 1.24E-10 | 1.80E-07 |
| BARX2 | 2.8549927 | 0.446742 | 6.3906973 | 1.65E-10 | 2.27E-07 |
| FCGBP | 3.0572123 | 0.483675 | 6.3207992 | 2.60E-10 | 3.39E-07 |
| NHLH2 | 7.1697226 | 1.1396417 | 6.2912079 | 3.15E-10 | 3.91E-07 |
| ZNF474 | 2.7693163 | 0.4475166 | 6.1881873 | 6.09E-10 | 6.90E-07 |
| ST6GALNAC1 | 2.3998784 | 0.3874358 | 6.1942615 | 5.86E-10 | 6.90E-07 |
| ENKUR | 2.6450123 | 0.4393183 | 6.02072 | 1.74E-09 | 1.89E-06 |
| CASC1 | 2.3480325 | 0.3904471 | 6.0137019 | 1.81E-09 | 1.89E-06 |
| CLIC6 | 2.1077891 | 0.3521284 | 5.9858534 | 2.15E-09 | 2.16E-06 |
| C11orf88 | 2.741352 | 0.4589632 | 5.9729234 | 2.33E-09 | 2.17E-06 |
| TRIM29 | 2.2107922 | 0.3698352 | 5.9777762 | 2.26E-09 | 2.17E-06 |
| TMEM212 | 2.6833649 | 0.4497552 | 5.9662783 | 2.43E-09 | 2.18E-06 |
| FAM83A | 4.9167239 | 0.8277548 | 5.9398311 | 2.85E-09 | 2.48E-06 |
| WDR63 | 2.4777432 | 0.4182405 | 5.9242073 | 3.14E-09 | 2.64E-06 |
| TNS4 | 3.7192077 | 0.6312148 | 5.8921429 | 3.81E-09 | 3.11E-06 |
| GPRC5D | -1.745287 | 0.2984992 | -5.846874 | 5.01E-09 | 3.88E-06 |
| SIX4 | 2.4017386 | 0.410909 | 5.84494 | 5.07E-09 | 3.88E-06 |
| ARMC4 | 2.4890816 | 0.4269697 | 5.8296443 | 5.55E-09 | 4.14E-06 |
| SPTBN2 | 1.7554563 | 0.3015576 | 5.8212968 | 5.84E-09 | 4.23E-06 |
| GLRB | 1.8101353 | 0.3122025 | 5.7979531 | 6.71E-09 | 4.60E-06 |
| NME5 | 2.2677305 | 0.3909503 | 5.8005594 | 6.61E-09 | 4.60E-06 |
| SNTN | 2.5580687 | 0.4418458 | 5.7895056 | 7.06E-09 | 4.69E-06 |
| SOX2 | 2.6974134 | 0.4661819 | 5.7861819 | 7.20E-09 | 4.69E-06 |
| MUC4 | 3.5129973 | 0.6087342 | 5.7709871 | 7.88E-09 | 4.80E-06 |
| TCTE1 | 2.5398004 | 0.4407435 | 5.7625364 | 8.29E-09 | 4.80E-06 |
| AQP6 | 2.3548053 | 0.4085662 | 5.763583 | 8.23E-09 | 4.80E-06 |
| CFAP61 | 2.5290271 | 0.438554 | 5.7667404 | 8.08E-09 | 4.80E-06 |
| STOX1 | 2.323729 | 0.404345 | 5.7468974 | 9.09E-09 | 5.15E-06 |
| PLEKHG7 | 3.189733 | 0.5555843 | 5.7412225 | 9.40E-09 | 5.21E-06 |
| VTCN1 | 3.6421686 | 0.6359943 | 5.7267317 | 1.02E-08 | 5.23E-06 |
| AL645924.1 | 2.6315644 | 0.4595108 | 5.7268831 | 1.02E-08 | 5.23E-06 |
| ENPP1 | -1.363831 | 0.2380961 | -5.72807 | 1.02E-08 | 5.23E-06 |
| CKMT1A | 2.2734282 | 0.3967505 | 5.7301212 | 1.00E-08 | 5.23E-06 |
| CCDC60 | 2.5541081 | 0.4472646 | 5.7105086 | 1.13E-08 | 5.65E-06 |
| STMND1 | 2.5643884 | 0.4500005 | 5.6986347 | 1.21E-08 | 5.94E-06 |
| WDR78 | 2.2065879 | 0.3877242 | 5.6911275 | 1.26E-08 | 6.00E-06 |
| ANKFN1 | 2.5128703 | 0.4415943 | 5.6904503 | 1.27E-08 | 6.00E-06 |
| DRC1 | 2.5677342 | 0.4524043 | 5.675751 | 1.38E-08 | 6.21E-06 |
| IL1RL1 | -3.41977 | 0.6022402 | -5.678415 | 1.36E-08 | 6.21E-06 |
| AC002064.2 | 6.867084 | 1.2087013 | 5.6813739 | 1.34E-08 | 6.21E-06 |
| FBXO15 | 2.5459206 | 0.449447 | 5.6645618 | 1.47E-08 | 6.51E-06 |
| CILP2 | 2.7188393 | 0.4804561 | 5.6588712 | 1.52E-08 | 6.62E-06 |
| FAM81B | 2.6236299 | 0.4644107 | 5.6493741 | 1.61E-08 | 6.88E-06 |
| TSPAN1 | 2.47541 | 0.4386553 | 5.643178 | 1.67E-08 | 7.02E-06 |
| ARMC3 | 2.5453028 | 0.4514739 | 5.6377627 | 1.72E-08 | 7.02E-06 |
| ARHGAP40 | 2.4983569 | 0.4431408 | 5.6378403 | 1.72E-08 | 7.02E-06 |
| CCDC65 | 2.309566 | 0.4100283 | 5.632699 | 1.77E-08 | 7.02E-06 |
| CFAP53 | 2.5027828 | 0.4443547 | 5.6323986 | 1.78E-08 | 7.02E-06 |
| RIBC2 | 2.463094 | 0.4376015 | 5.6286233 | 1.82E-08 | 7.07E-06 |
| CWH43 | 2.8801639 | 0.5121358 | 5.6238286 | 1.87E-08 | 7.16E-06 |
| UGT1A6 | 5.8245773 | 1.0389814 | 5.6060458 | 2.07E-08 | 7.60E-06 |
| ADAMDEC1 | 3.6136193 | 0.6445716 | 5.6062344 | 2.07E-08 | 7.60E-06 |
| C20orf85 | 2.5752787 | 0.4591852 | 5.6083665 | 2.04E-08 | 7.60E-06 |
| MDH1B | 2.4314044 | 0.4342259 | 5.5993992 | 2.15E-08 | 7.73E-06 |
| EYA1 | 2.5304978 | 0.4520055 | 5.5983784 | 2.16E-08 | 7.73E-06 |
| UBXN10 | 2.3055973 | 0.4123623 | 5.5911937 | 2.26E-08 | 7.84E-06 |
| SCGB1A1 | 2.9620474 | 0.5296214 | 5.5927635 | 2.23E-08 | 7.84E-06 |
| HSD17B13 | 3.4353869 | 0.6156582 | 5.5800235 | 2.40E-08 | 8.10E-06 |
| LRRC18 | 2.134597 | 0.3826334 | 5.5786996 | 2.42E-08 | 8.10E-06 |
| RSPH1 | 2.4716722 | 0.4427289 | 5.5828121 | 2.37E-08 | 8.10E-06 |
| EFCAB1 | 2.4320907 | 0.4363168 | 5.5741392 | 2.49E-08 | 8.21E-06 |
| SPATA17 | 2.287514 | 0.4107245 | 5.5694601 | 2.56E-08 | 8.29E-06 |
| DAW1 | 2.4403819 | 0.4384778 | 5.5655762 | 2.61E-08 | 8.29E-06 |
| NTRK2 | 2.4523425 | 0.4405711 | 5.5662811 | 2.60E-08 | 8.29E-06 |
| PAQR5 | -1.091489 | 0.1961804 | -5.563698 | 2.64E-08 | 8.29E-06 |
| C9orf135 | 2.435233 | 0.437865 | 5.5616068 | 2.67E-08 | 8.29E-06 |
| AL121956.6 | 2.443035 | 0.4394473 | 5.5593356 | 2.71E-08 | 8.30E-06 |
| RSPH4A | 2.4316841 | 0.4376257 | 5.5565387 | 2.75E-08 | 8.34E-06 |
| CA8 | 1.5710769 | 0.2829122 | 5.5532304 | 2.80E-08 | 8.37E-06 |
| KCNRG | 2.8192457 | 0.5079751 | 5.5499684 | 2.86E-08 | 8.37E-06 |
| TEKT1 | 2.5128944 | 0.4527451 | 5.5503511 | 2.85E-08 | 8.37E-06 |
| WDR86-AS1 | 2.4128041 | 0.4354478 | 5.5409723 | 3.01E-08 | 8.63E-06 |
| AC025154.2 | 2.9113816 | 0.5254519 | 5.5407199 | 3.01E-08 | 8.63E-06 |
| CHST9 | 2.7180056 | 0.490995 | 5.535709 | 3.10E-08 | 8.78E-06 |
| AK8 | 2.38459 | 0.4310442 | 5.5321237 | 3.16E-08 | 8.87E-06 |
| C6 | 2.3605676 | 0.4270298 | 5.5278759 | 3.24E-08 | 8.99E-06 |
| C6orf118 | 2.4827694 | 0.4494159 | 5.5244364 | 3.31E-08 | 9.05E-06 |
| PACRG | 2.3768216 | 0.4303504 | 5.5229911 | 3.33E-08 | 9.05E-06 |
| HACD1 | -1.011589 | 0.1835439 | -5.511427 | 3.56E-08 | 9.47E-06 |
| LRRC10B | 2.4665722 | 0.4474575 | 5.5124166 | 3.54E-08 | 9.47E-06 |
| CFAP126 | 2.3081231 | 0.419596 | 5.5008223 | 3.78E-08 | 9.95E-06 |
| RSPH9 | 2.2711983 | 0.4132174 | 5.4963765 | 3.88E-08 | 1.01E-05 |
| IGKV3-11 | 3.3436238 | 0.6087679 | 5.4924442 | 3.96E-08 | 1.02E-05 |
| EFHB | 2.2631463 | 0.4122915 | 5.4891897 | 4.04E-08 | 1.03E-05 |
| CPEB1 | 2.0325651 | 0.3704308 | 5.487031 | 4.09E-08 | 1.03E-05 |
| CCDC148 | 1.7629224 | 0.3216104 | 5.481546 | 4.22E-08 | 1.06E-05 |
| GLB1L2 | 2.0203949 | 0.3690412 | 5.4747136 | 4.38E-08 | 1.09E-05 |
| VSIG1 | 4.140563 | 0.7566107 | 5.4725141 | 4.44E-08 | 1.09E-05 |
| DRC7 | 2.4059398 | 0.4398551 | 5.4698459 | 4.50E-08 | 1.10E-05 |
| TP73 | 2.3912441 | 0.4375184 | 5.4654707 | 4.62E-08 | 1.10E-05 |
| BMPR1B | 2.5666744 | 0.4695103 | 5.466705 | 4.58E-08 | 1.10E-05 |
| CD24 | 2.0857128 | 0.3818224 | 5.4625206 | 4.69E-08 | 1.11E-05 |
| MS4A8 | 2.3646496 | 0.433744 | 5.4517172 | 4.99E-08 | 1.17E-05 |
| INHBB | 1.9712401 | 0.3621362 | 5.443367 | 5.23E-08 | 1.21E-05 |
| GSTA3 | 2.4653878 | 0.4530986 | 5.4411734 | 5.29E-08 | 1.21E-05 |
| AK7 | 2.2778244 | 0.4185813 | 5.4417726 | 5.28E-08 | 1.21E-05 |
| RSPH10B | 2.4103216 | 0.4437401 | 5.431832 | 5.58E-08 | 1.24E-05 |
| MUC5B | 6.0497259 | 1.1133699 | 5.4337068 | 5.52E-08 | 1.24E-05 |
| AP003064.2 | 3.6575128 | 0.6731924 | 5.4330871 | 5.54E-08 | 1.24E-05 |
| CCDC113 | 2.335566 | 0.4304612 | 5.4257288 | 5.77E-08 | 1.26E-05 |
| AC010624.1 | 2.388039 | 0.4401386 | 5.4256518 | 5.77E-08 | 1.26E-05 |
| ZBBX | 2.3142327 | 0.4271246 | 5.418168 | 6.02E-08 | 1.27E-05 |
| FAT2 | 3.0890894 | 0.5699269 | 5.4201502 | 5.95E-08 | 1.27E-05 |
| CFAP97D2 | 2.512418 | 0.4635868 | 5.4195198 | 5.98E-08 | 1.27E-05 |
| AC005962.2 | 2.8471495 | 0.5253972 | 5.4190415 | 5.99E-08 | 1.27E-05 |
| LINC00511 | 2.4604566 | 0.4542694 | 5.4162944 | 6.08E-08 | 1.27E-05 |
| FER1L4 | 1.7853798 | 0.3296121 | 5.4166085 | 6.07E-08 | 1.27E-05 |
| EFCAB6 | 2.2047177 | 0.4071478 | 5.4150302 | 6.13E-08 | 1.27E-05 |
| MORN5 | 2.4686299 | 0.4560921 | 5.4125682 | 6.21E-08 | 1.28E-05 |
| FNDC11 | 2.282962 | 0.4221244 | 5.4082687 | 6.36E-08 | 1.30E-05 |
| CFAP57 | 2.3685173 | 0.4385673 | 5.400579 | 6.64E-08 | 1.34E-05 |
| LINC00639 | 2.5208828 | 0.4673705 | 5.3937566 | 6.90E-08 | 1.38E-05 |
| DNAI2 | 2.3944192 | 0.4442059 | 5.3903364 | 7.03E-08 | 1.40E-05 |
| APOBEC4 | 2.6123902 | 0.4853261 | 5.3827518 | 7.34E-08 | 1.43E-05 |
| IQCG | 2.0908062 | 0.3886235 | 5.3800308 | 7.45E-08 | 1.43E-05 |
| CNGA4 | 2.4744065 | 0.4599756 | 5.3794297 | 7.47E-08 | 1.43E-05 |
| CCDC81 | 2.2212156 | 0.4127262 | 5.3818134 | 7.37E-08 | 1.43E-05 |
| USP2-AS1 | 2.0801881 | 0.386359 | 5.3840809 | 7.28E-08 | 1.43E-05 |
| CFAP77 | 2.6854393 | 0.4996206 | 5.3749571 | 7.66E-08 | 1.46E-05 |
| FERMT1 | 2.7712311 | 0.5160354 | 5.3702345 | 7.86E-08 | 1.49E-05 |
| CATSPERD | 2.6324881 | 0.4904559 | 5.3674308 | 7.99E-08 | 1.50E-05 |
| CALML4 | 1.8998576 | 0.3541162 | 5.3650679 | 8.09E-08 | 1.51E-05 |
| DNAH6 | 2.146795 | 0.4007924 | 5.356376 | 8.49E-08 | 1.54E-05 |
| VWA3B | 2.4025863 | 0.4485391 | 5.3564698 | 8.49E-08 | 1.54E-05 |
| CFAP43 | 2.3214434 | 0.4332613 | 5.3580677 | 8.41E-08 | 1.54E-05 |
| LCA5L | 2.2035584 | 0.411108 | 5.3600473 | 8.32E-08 | 1.54E-05 |
| FHOD3 | 2.0808178 | 0.3888622 | 5.3510417 | 8.74E-08 | 1.57E-05 |
| AQP5 | 2.0996737 | 0.3926063 | 5.3480391 | 8.89E-08 | 1.58E-05 |
| AC007906.2 | 2.5112346 | 0.4695568 | 5.3480951 | 8.89E-08 | 1.58E-05 |
| LINC01571 | 2.5159508 | 0.4707113 | 5.3449977 | 9.04E-08 | 1.58E-05 |
| CAPS | 2.2711249 | 0.42491 | 5.344955 | 9.04E-08 | 1.58E-05 |
| SPATS1 | 2.766966 | 0.5180122 | 5.3415078 | 9.22E-08 | 1.60E-05 |
| CAPSL | 2.4055566 | 0.4506604 | 5.3378472 | 9.41E-08 | 1.62E-05 |
| CFAP52 | 2.4040335 | 0.4504879 | 5.336511 | 9.48E-08 | 1.62E-05 |
| DNAH7 | 2.3744329 | 0.4450635 | 5.3350431 | 9.55E-08 | 1.63E-05 |
| C21orf58 | 2.2541673 | 0.4231556 | 5.3270412 | 9.98E-08 | 1.69E-05 |
| DNAH5 | 2.4122895 | 0.4532517 | 5.3221849 | 1.03E-07 | 1.72E-05 |
| DNAI1 | 2.2726822 | 0.4279196 | 5.3110023 | 1.09E-07 | 1.82E-05 |
| GRM4 | 3.2633225 | 0.6147493 | 5.3083795 | 1.11E-07 | 1.84E-05 |
| AQP4-AS1 | 2.0435978 | 0.3852926 | 5.3040147 | 1.13E-07 | 1.87E-05 |
| ZNF331 | -1.295337 | 0.2442574 | -5.303164 | 1.14E-07 | 1.87E-05 |
| TEKT4 | 2.5860114 | 0.4889447 | 5.2889647 | 1.23E-07 | 1.96E-05 |
| AC095050.1 | -7.379391 | 1.395381 | -5.288442 | 1.23E-07 | 1.96E-05 |
| C11orf16 | 2.4045157 | 0.4543904 | 5.2917397 | 1.21E-07 | 1.96E-05 |
| GAS2L2 | 2.3792572 | 0.4495291 | 5.2927766 | 1.20E-07 | 1.96E-05 |
| CCDC151 | 2.4310756 | 0.4595435 | 5.2901968 | 1.22E-07 | 1.96E-05 |
| NGEF | 2.1367137 | 0.4043202 | 5.2847064 | 1.26E-07 | 1.98E-05 |
| AC089999.1 | 2.7212791 | 0.5150353 | 5.2836752 | 1.27E-07 | 1.98E-05 |
| FOXJ1 | 2.4272814 | 0.4594213 | 5.2833449 | 1.27E-07 | 1.98E-05 |
| MNS1 | 2.0064873 | 0.3799483 | 5.2809481 | 1.29E-07 | 1.99E-05 |
| CCDC173 | 2.2795841 | 0.4318132 | 5.2790978 | 1.30E-07 | 2.00E-05 |
| C8orf34 | 2.1118087 | 0.4001293 | 5.2778157 | 1.31E-07 | 2.00E-05 |
| RHPN1-AS1 | 2.0555202 | 0.3895907 | 5.2761024 | 1.32E-07 | 2.00E-05 |
| FAM92B | 2.4357248 | 0.4616157 | 5.2765211 | 1.32E-07 | 2.00E-05 |
| ARX | 2.4282385 | 0.4603922 | 5.2742821 | 1.33E-07 | 2.01E-05 |
| NECTIN4 | 1.9085106 | 0.3619665 | 5.2726168 | 1.34E-07 | 2.01E-05 |
| ADGB | 2.3755977 | 0.450861 | 5.269025 | 1.37E-07 | 2.04E-05 |
| TUBA4B | 2.3880599 | 0.4534833 | 5.2660375 | 1.39E-07 | 2.06E-05 |
| SCGB3A1 | 3.9428551 | 0.7493071 | 5.2620018 | 1.42E-07 | 2.10E-05 |
| AMY1B | 2.0782663 | 0.3953291 | 5.2570536 | 1.46E-07 | 2.11E-05 |
| SRGAP3-AS2 | 2.6607392 | 0.5063544 | 5.2546976 | 1.48E-07 | 2.11E-05 |
| C3orf67 | 2.3380769 | 0.4449431 | 5.2547773 | 1.48E-07 | 2.11E-05 |
| SPP1 | 3.8279496 | 0.7280588 | 5.257748 | 1.46E-07 | 2.11E-05 |
| COL17A1 | 3.557081 | 0.6768257 | 5.2555351 | 1.48E-07 | 2.11E-05 |
| KIF19 | 2.3011775 | 0.4375565 | 5.259155 | 1.45E-07 | 2.11E-05 |
| CD164L2 | 2.2651775 | 0.4317459 | 5.2465519 | 1.55E-07 | 2.13E-05 |
| CCDC17 | 2.3254605 | 0.4434523 | 5.2439929 | 1.57E-07 | 2.13E-05 |
| AMY1C | 2.1213297 | 0.4045322 | 5.2439087 | 1.57E-07 | 2.13E-05 |
| CFAP45 | 2.3800725 | 0.4539818 | 5.2426612 | 1.58E-07 | 2.13E-05 |
| IGKV2-28 | 3.0463526 | 0.5804823 | 5.247968 | 1.54E-07 | 2.13E-05 |
| NEK10 | 2.2773357 | 0.4344327 | 5.2420913 | 1.59E-07 | 2.13E-05 |
| DZIP3 | 1.5673161 | 0.2985605 | 5.2495763 | 1.52E-07 | 2.13E-05 |
| LEKR1 | 1.895384 | 0.3615567 | 5.2422865 | 1.59E-07 | 2.13E-05 |
| CCDC146 | 1.9064405 | 0.363166 | 5.2495019 | 1.53E-07 | 2.13E-05 |
| STOML3 | 2.6331858 | 0.5017498 | 5.2480058 | 1.54E-07 | 2.13E-05 |
| PIH1D3 | 2.5531852 | 0.4869811 | 5.242884 | 1.58E-07 | 2.13E-05 |
| STK33 | 2.1425301 | 0.4089038 | 5.2396928 | 1.61E-07 | 2.14E-05 |
| AL357093.2 | 2.2331323 | 0.4261504 | 5.240245 | 1.60E-07 | 2.14E-05 |
| SPEF1 | 2.4037106 | 0.4594136 | 5.2321273 | 1.68E-07 | 2.22E-05 |
| RIBC1 | 2.1309406 | 0.4076239 | 5.2277131 | 1.72E-07 | 2.26E-05 |
| TEX9 | 2.2235864 | 0.4254626 | 5.2262798 | 1.73E-07 | 2.27E-05 |
| WNK2 | 2.1902548 | 0.4193014 | 5.2235805 | 1.75E-07 | 2.29E-05 |
| SAXO2 | 2.2579954 | 0.432656 | 5.2189161 | 1.80E-07 | 2.33E-05 |
| ESPN | 2.2694244 | 0.4354318 | 5.2118946 | 1.87E-07 | 2.41E-05 |
| PIFO | 2.1902113 | 0.4202698 | 5.2114405 | 1.87E-07 | 2.41E-05 |
| TEKT3 | 2.3765748 | 0.4565649 | 5.205338 | 1.94E-07 | 2.47E-05 |
| DACH2 | -2.050665 | 0.394086 | -5.203599 | 1.95E-07 | 2.49E-05 |
| LDLRAD1 | 2.4504406 | 0.4712219 | 5.2001839 | 1.99E-07 | 2.52E-05 |
| ACBD7 | 2.2166991 | 0.4264589 | 5.1979193 | 2.02E-07 | 2.54E-05 |
| AGBL2 | 2.1908916 | 0.4216624 | 5.1958429 | 2.04E-07 | 2.55E-05 |
| C9orf24 | 2.485275 | 0.4784838 | 5.1940632 | 2.06E-07 | 2.56E-05 |
| AP004608.1 | 2.5260347 | 0.4865652 | 5.1915641 | 2.09E-07 | 2.56E-05 |
| WDR66 | 2.1455154 | 0.4132518 | 5.1917878 | 2.08E-07 | 2.56E-05 |
| LINC02345 | 2.0706596 | 0.3988273 | 5.1918706 | 2.08E-07 | 2.56E-05 |
| MAP3K19 | 2.3689277 | 0.4564225 | 5.1902081 | 2.10E-07 | 2.57E-05 |
| INSC | -1.840787 | 0.3550103 | -5.185165 | 2.16E-07 | 2.60E-05 |
| SLC27A2 | 2.3859555 | 0.4600743 | 5.1860217 | 2.15E-07 | 2.60E-05 |
| LRRC46 | 2.416186 | 0.4658731 | 5.1863613 | 2.14E-07 | 2.60E-05 |
| AKAP14 | 2.3793661 | 0.4590716 | 5.1829954 | 2.18E-07 | 2.62E-05 |
| CFAP58 | 2.0624864 | 0.3982922 | 5.1783244 | 2.24E-07 | 2.68E-05 |
| PTPRT | 2.3008612 | 0.4444318 | 5.1770847 | 2.25E-07 | 2.68E-05 |
| SPA17 | 1.8972465 | 0.3665785 | 5.1755533 | 2.27E-07 | 2.69E-05 |
| RGS22 | 2.11815 | 0.4093282 | 5.1746981 | 2.28E-07 | 2.69E-05 |
| FHAD1 | 2.1517694 | 0.416092 | 5.1713783 | 2.32E-07 | 2.73E-05 |
| PPIL6 | 1.9864076 | 0.3852438 | 5.1562348 | 2.52E-07 | 2.95E-05 |
| NWD1 | 2.4736671 | 0.4798686 | 5.1548839 | 2.54E-07 | 2.95E-05 |
| PROM1 | 3.8874238 | 0.7543819 | 5.1531245 | 2.56E-07 | 2.97E-05 |
| SRD5A2 | 2.7926252 | 0.5423296 | 5.1493138 | 2.61E-07 | 3.02E-05 |
| PIGR | 1.9814592 | 0.3849377 | 5.1474802 | 2.64E-07 | 3.03E-05 |
| TTC29 | 2.3615102 | 0.4594097 | 5.1403145 | 2.74E-07 | 3.11E-05 |
| ACY3 | 5.0987141 | 0.9918032 | 5.1408525 | 2.73E-07 | 3.11E-05 |
| LINC01996 | -1.509674 | 0.2936116 | -5.14174 | 2.72E-07 | 3.11E-05 |
| TEKT2 | 2.3780351 | 0.4629491 | 5.1367096 | 2.80E-07 | 3.14E-05 |
| KCNJ16 | 2.283758 | 0.4445553 | 5.1371738 | 2.79E-07 | 3.14E-05 |
| KCNE1B | 2.1492941 | 0.4185314 | 5.1353236 | 2.82E-07 | 3.15E-05 |
| DCDC1 | 2.3133662 | 0.4507055 | 5.1327669 | 2.86E-07 | 3.18E-05 |
| HES2 | 2.0054 | 0.3911231 | 5.1272865 | 2.94E-07 | 3.22E-05 |
| C2orf50 | 2.2543446 | 0.4396225 | 5.12791 | 2.93E-07 | 3.22E-05 |
| CCDC74B | 2.2846446 | 0.4455381 | 5.1278318 | 2.93E-07 | 3.22E-05 |
| PITX1 | 3.457721 | 0.6750338 | 5.1222928 | 3.02E-07 | 3.29E-05 |
| DRC3 | 2.0437518 | 0.3990834 | 5.1211145 | 3.04E-07 | 3.30E-05 |
| CLGN | 2.0322999 | 0.3969674 | 5.1195632 | 3.06E-07 | 3.31E-05 |
| LINC01765 | 2.467141 | 0.4819872 | 5.1186858 | 3.08E-07 | 3.31E-05 |
| CDHR4 | 2.3970911 | 0.4688194 | 5.1130371 | 3.17E-07 | 3.39E-05 |
| LRRC43 | 2.3519721 | 0.4599966 | 5.113021 | 3.17E-07 | 3.39E-05 |
| KCNK2 | 4.1807531 | 0.8183055 | 5.1090371 | 3.24E-07 | 3.45E-05 |
| PRR7 | 2.0832975 | 0.4080386 | 5.1056378 | 3.30E-07 | 3.49E-05 |
| DYDC2 | 2.2621931 | 0.4432598 | 5.1035376 | 3.33E-07 | 3.52E-05 |
| PTPRZ1 | 3.1486479 | 0.6170783 | 5.1025094 | 3.35E-07 | 3.52E-05 |
| OMG | 2.4136719 | 0.473356 | 5.0990623 | 3.41E-07 | 3.56E-05 |
| CCDC114 | 2.1275434 | 0.4172614 | 5.0988257 | 3.42E-07 | 3.56E-05 |
| FAM183A | 2.3451874 | 0.4602148 | 5.0958542 | 3.47E-07 | 3.59E-05 |
| FAM166B | 2.2883129 | 0.4490204 | 5.0962335 | 3.46E-07 | 3.59E-05 |
| BCAS1 | 2.7722135 | 0.5443102 | 5.0930761 | 3.52E-07 | 3.63E-05 |
| EFHC2 | 2.286198 | 0.4490355 | 5.0913529 | 3.56E-07 | 3.65E-05 |
| HHLA2 | 2.2676786 | 0.445468 | 5.0905534 | 3.57E-07 | 3.65E-05 |
| ROPN1L | 2.3293778 | 0.4578203 | 5.0879745 | 3.62E-07 | 3.68E-05 |
| C1orf87 | 2.3626075 | 0.4646591 | 5.0846044 | 3.68E-07 | 3.70E-05 |
| CFC1 | 2.7897341 | 0.548642 | 5.0847987 | 3.68E-07 | 3.70E-05 |
| SPAG8 | 2.2636341 | 0.4451814 | 5.084745 | 3.68E-07 | 3.70E-05 |
| CCDC78 | 2.2799472 | 0.4484519 | 5.0840397 | 3.69E-07 | 3.70E-05 |
| TTC25 | 2.2240016 | 0.437642 | 5.0817826 | 3.74E-07 | 3.72E-05 |
| FAM81A | 2.050748 | 0.4036884 | 5.0800275 | 3.77E-07 | 3.74E-05 |
| CKMT1B | 2.0563036 | 0.4048599 | 5.07905 | 3.79E-07 | 3.75E-05 |
| C1orf194 | 2.4721127 | 0.4869824 | 5.0763903 | 3.85E-07 | 3.78E-05 |
| IGHV1-18 | 2.9036145 | 0.5720752 | 5.0755819 | 3.86E-07 | 3.79E-05 |
| DNAAF3 | 2.419783 | 0.4778968 | 5.0634011 | 4.12E-07 | 4.02E-05 |
| C7orf57 | 2.3266082 | 0.4599905 | 5.0579487 | 4.24E-07 | 4.12E-05 |
| RSPH10B2 | 2.7016163 | 0.5343774 | 5.0556334 | 4.29E-07 | 4.16E-05 |
| SPAG6 | 2.3129763 | 0.4578015 | 5.0523565 | 4.36E-07 | 4.20E-05 |
| LRRC23 | 2.0513769 | 0.4059795 | 5.052907 | 4.35E-07 | 4.20E-05 |
| PPP1R42 | 2.5109696 | 0.4971572 | 5.0506547 | 4.40E-07 | 4.22E-05 |
| AL353660.1 | 2.4823594 | 0.4917794 | 5.0477094 | 4.47E-07 | 4.27E-05 |
| TRIP13 | 1.900199 | 0.3766244 | 5.0453419 | 4.53E-07 | 4.31E-05 |
| KLK13 | 2.9689522 | 0.5887358 | 5.0429275 | 4.58E-07 | 4.35E-05 |
| AC019117.3 | 3.2348945 | 0.6419319 | 5.0393106 | 4.67E-07 | 4.40E-05 |
| TEX26 | 2.7554071 | 0.546814 | 5.0390211 | 4.68E-07 | 4.40E-05 |
| CYP2J2 | 1.7173001 | 0.3409734 | 5.036464 | 4.74E-07 | 4.45E-05 |
| AC096637.3 | 2.2321771 | 0.4438885 | 5.0286885 | 4.94E-07 | 4.60E-05 |
| IQCD | 2.2118005 | 0.4398449 | 5.0285921 | 4.94E-07 | 4.60E-05 |
| CERKL | 2.318263 | 0.4611458 | 5.0271803 | 4.98E-07 | 4.61E-05 |
| RP1 | 1.902455 | 0.3784955 | 5.0263612 | 5.00E-07 | 4.61E-05 |
| LMNTD1 | 2.5736081 | 0.5120525 | 5.0260633 | 5.01E-07 | 4.61E-05 |
| GABRP | 2.914272 | 0.5799365 | 5.0251574 | 5.03E-07 | 4.62E-05 |
| TMEM231 | 2.1006578 | 0.4180837 | 5.0244913 | 5.05E-07 | 4.62E-05 |
| ICAM1 | -1.682496 | 0.3349104 | -5.023719 | 5.07E-07 | 4.62E-05 |
| E2F8 | 1.8693694 | 0.3724117 | 5.0196319 | 5.18E-07 | 4.70E-05 |
| DYNLRB2 | 2.227083 | 0.4438953 | 5.0171363 | 5.24E-07 | 4.75E-05 |
| IQCH | 1.9857895 | 0.396227 | 5.0117471 | 5.39E-07 | 4.86E-05 |
| CBLC | 1.6946747 | 0.3382432 | 5.0102254 | 5.44E-07 | 4.89E-05 |
| SVOPL | 2.0152677 | 0.402516 | 5.0066777 | 5.54E-07 | 4.96E-05 |
| PLPP2 | 2.2220549 | 0.4440484 | 5.0040824 | 5.61E-07 | 5.01E-05 |
| CFAP65 | 2.3205155 | 0.4639727 | 5.0014052 | 5.69E-07 | 5.02E-05 |
| FBXO36 | 1.7532902 | 0.3504516 | 5.0029457 | 5.65E-07 | 5.02E-05 |
| TMEM232 | 2.236898 | 0.4473418 | 5.0004227 | 5.72E-07 | 5.02E-05 |
| WDR38 | 2.4961268 | 0.4992323 | 4.9999304 | 5.74E-07 | 5.02E-05 |
| ENO4 | 2.3766119 | 0.475289 | 5.0003516 | 5.72E-07 | 5.02E-05 |
| PTP4A1P2 | 6.3002177 | 1.2595127 | 5.0021073 | 5.67E-07 | 5.02E-05 |
| CABCOCO1 | 2.0705608 | 0.4142432 | 4.9984187 | 5.78E-07 | 5.04E-05 |
| MAATS1 | 1.8428532 | 0.3688368 | 4.9963921 | 5.84E-07 | 5.08E-05 |
| SPATA18 | 2.0984299 | 0.4201222 | 4.9948077 | 5.89E-07 | 5.10E-05 |
| GRIA1 | -1.381006 | 0.2767046 | -4.990904 | 6.01E-07 | 5.17E-05 |
| AL590491.2 | 2.1939927 | 0.4395892 | 4.9910066 | 6.01E-07 | 5.17E-05 |
| IGSF9 | 1.8765452 | 0.3760766 | 4.9897954 | 6.04E-07 | 5.18E-05 |
| EPN3 | 2.3023282 | 0.4616302 | 4.9873864 | 6.12E-07 | 5.23E-05 |
| TUBB1 | -2.098769 | 0.4210788 | -4.984266 | 6.22E-07 | 5.30E-05 |
| SORCS1 | 2.6102926 | 0.523906 | 4.9823682 | 6.28E-07 | 5.32E-05 |
| LIF-AS1 | 7.0700019 | 1.4190482 | 4.9822141 | 6.29E-07 | 5.32E-05 |
| PIH1D2 | 1.9448806 | 0.3909603 | 4.9746246 | 6.54E-07 | 5.48E-05 |
| USH1G | 6.1148055 | 1.2291096 | 4.9749881 | 6.53E-07 | 5.48E-05 |
| FAM216B | 2.1615699 | 0.4346896 | 4.9726748 | 6.60E-07 | 5.50E-05 |
| SRCIN1 | 2.1111072 | 0.424554 | 4.972529 | 6.61E-07 | 5.50E-05 |
| EFCAB12 | 2.1866058 | 0.4400591 | 4.9688917 | 6.73E-07 | 5.59E-05 |
| FANK1 | 2.1857084 | 0.4402354 | 4.9648628 | 6.87E-07 | 5.69E-05 |
| ZNF473 | 2.0374811 | 0.4104741 | 4.9637267 | 6.92E-07 | 5.70E-05 |
| SDR16C5 | -1.330246 | 0.2681488 | -4.960852 | 7.02E-07 | 5.76E-05 |
| CFAP300 | 2.2436815 | 0.4522942 | 4.9606678 | 7.03E-07 | 5.76E-05 |
| DNAH9 | 2.3386752 | 0.471498 | 4.9600956 | 7.05E-07 | 5.76E-05 |
| GAS2L1P2 | 7.1502534 | 1.4422053 | 4.9578612 | 7.13E-07 | 5.81E-05 |
| ZMYND12 | 2.3592878 | 0.4761489 | 4.9549371 | 7.24E-07 | 5.84E-05 |
| DNAH11 | 2.0070542 | 0.4050435 | 4.9551575 | 7.23E-07 | 5.84E-05 |
| AC027237.5 | 1.9413847 | 0.3917132 | 4.9561383 | 7.19E-07 | 5.84E-05 |
| DPY19L2P4 | 2.4029459 | 0.4852027 | 4.952458 | 7.33E-07 | 5.90E-05 |
| C1orf158 | 2.3927957 | 0.4833659 | 4.9502785 | 7.41E-07 | 5.93E-05 |
| DNAJB13 | 2.237854 | 0.4520474 | 4.9504852 | 7.40E-07 | 5.93E-05 |
| CCDC39 | 2.1402693 | 0.4327449 | 4.9457995 | 7.58E-07 | 6.03E-05 |
| ABCA13 | 2.9460493 | 0.5956461 | 4.9459728 | 7.58E-07 | 6.03E-05 |
| CD101 | -1.618498 | 0.3273026 | -4.944959 | 7.62E-07 | 6.03E-05 |
| AL163051.1 | 2.4242702 | 0.4906866 | 4.9405678 | 7.79E-07 | 6.15E-05 |
| CCDC74A | 2.1129464 | 0.4278189 | 4.9388805 | 7.86E-07 | 6.17E-05 |
| AL138885.3 | 4.0751626 | 0.8251204 | 4.9388701 | 7.86E-07 | 6.17E-05 |
| AC011487.1 | -2.185442 | 0.4425602 | -4.93818 | 7.89E-07 | 6.17E-05 |
| WFDC2 | 2.2831734 | 0.4624367 | 4.9372669 | 7.92E-07 | 6.18E-05 |
| MARCHF10 | 2.2767047 | 0.4614399 | 4.9339142 | 8.06E-07 | 6.25E-05 |
| GRIN3B | 2.0340157 | 0.4122288 | 4.9341917 | 8.05E-07 | 6.25E-05 |
| ELN-AS1 | 2.0543661 | 0.4165613 | 4.9317255 | 8.15E-07 | 6.29E-05 |
| CCNA1 | 2.473723 | 0.5016761 | 4.9309169 | 8.18E-07 | 6.29E-05 |
| TTC6 | 1.8891657 | 0.3830867 | 4.9314318 | 8.16E-07 | 6.29E-05 |
| TTLL10 | 2.2632169 | 0.4591205 | 4.9294616 | 8.25E-07 | 6.30E-05 |
| AC023796.1 | 3.1261415 | 0.6342253 | 4.9290711 | 8.26E-07 | 6.30E-05 |
| NELL2 | 2.1580185 | 0.4377252 | 4.9300756 | 8.22E-07 | 6.30E-05 |
| LINC00689 | 2.1089814 | 0.4280013 | 4.9275114 | 8.33E-07 | 6.33E-05 |
| SCUBE3 | 1.3847329 | 0.2810822 | 4.9264344 | 8.37E-07 | 6.35E-05 |
| CCDC89 | 2.0979433 | 0.4260617 | 4.9240369 | 8.48E-07 | 6.37E-05 |
| NHLRC4 | 1.7231525 | 0.3498942 | 4.924782 | 8.45E-07 | 6.37E-05 |
| FAM83F | 2.1511275 | 0.4368271 | 4.9244367 | 8.46E-07 | 6.37E-05 |
| GRIK5 | 1.6770341 | 0.3411947 | 4.9151823 | 8.87E-07 | 6.64E-05 |
| ZMYND10 | 2.1800967 | 0.4441064 | 4.9089514 | 9.16E-07 | 6.84E-05 |
| KCNH3 | 2.1046976 | 0.4288779 | 4.9074513 | 9.23E-07 | 6.87E-05 |
| C9orf116 | 2.2863429 | 0.4660043 | 4.9062695 | 9.28E-07 | 6.89E-05 |
| PLA2G7 | 2.9016388 | 0.5915344 | 4.9052747 | 9.33E-07 | 6.91E-05 |
| KRT5 | 3.5635848 | 0.726643 | 4.9041756 | 9.38E-07 | 6.93E-05 |
| DCDC2B | 2.143951 | 0.4374681 | 4.9008167 | 9.54E-07 | 6.99E-05 |
| FAM166C | 2.4631813 | 0.5026121 | 4.9007598 | 9.55E-07 | 6.99E-05 |
| ANKRD36BP2 | 1.6812724 | 0.3429936 | 4.9017604 | 9.50E-07 | 6.99E-05 |
| WDR93 | 2.2329074 | 0.4558106 | 4.8987619 | 9.64E-07 | 7.04E-05 |
| GSTA1 | 2.4328929 | 0.4969904 | 4.8952516 | 9.82E-07 | 7.13E-05 |
| KIF24 | 1.9252114 | 0.3932834 | 4.8952263 | 9.82E-07 | 7.13E-05 |
| BIK | 1.4427365 | 0.2949889 | 4.8908166 | 1.00E-06 | 7.27E-05 |
| DEUP1 | 2.2620683 | 0.4627452 | 4.8883667 | 1.02E-06 | 7.34E-05 |
| AC245297.1 | 1.58712 | 0.3248386 | 4.8858725 | 1.03E-06 | 7.39E-05 |
| CCDC157 | 1.9545659 | 0.4000247 | 4.8861126 | 1.03E-06 | 7.39E-05 |
| AMY1A | 1.9918006 | 0.4080499 | 4.8812676 | 1.05E-06 | 7.53E-05 |
| IGHV3-23 | 2.7815851 | 0.5698505 | 4.8812546 | 1.05E-06 | 7.53E-05 |
| C1orf189 | 2.4269395 | 0.4973567 | 4.8796758 | 1.06E-06 | 7.56E-05 |
| LRRIQ1 | 2.2498747 | 0.4611001 | 4.879363 | 1.06E-06 | 7.56E-05 |
| CSMD1 | 2.111429 | 0.432984 | 4.8764595 | 1.08E-06 | 7.65E-05 |
| C22orf15 | 2.3099649 | 0.473783 | 4.8755755 | 1.08E-06 | 7.66E-05 |
| CLDN8 | 2.6532113 | 0.54454 | 4.8723901 | 1.10E-06 | 7.75E-05 |
| RAB36 | 2.0170219 | 0.413961 | 4.8724924 | 1.10E-06 | 7.75E-05 |
| CCDC33 | 2.2394304 | 0.4599598 | 4.868753 | 1.12E-06 | 7.87E-05 |
| AC242022.1 | 2.3255069 | 0.4779357 | 4.8657317 | 1.14E-06 | 7.97E-05 |
| CDHR3 | 2.2014601 | 0.4525537 | 4.8645276 | 1.15E-06 | 8.00E-05 |
| TOGARAM2 | 2.0208118 | 0.4155578 | 4.8628892 | 1.16E-06 | 8.02E-05 |
| ALDH1L1 | 2.061162 | 0.4238594 | 4.8628434 | 1.16E-06 | 8.02E-05 |
| NEK11 | 1.8093657 | 0.3725837 | 4.856266 | 1.20E-06 | 8.23E-05 |
| CFAP299 | 2.3963577 | 0.4934139 | 4.8566886 | 1.19E-06 | 8.23E-05 |
| TMC3-AS1 | 1.5641511 | 0.322064 | 4.8566471 | 1.19E-06 | 8.23E-05 |
| PRR18 | 2.51762 | 0.519287 | 4.8482243 | 1.25E-06 | 8.54E-05 |
| MYB | 1.7979791 | 0.3709212 | 4.8473342 | 1.25E-06 | 8.56E-05 |
| GALNT15 | 1.9924438 | 0.4112234 | 4.8451622 | 1.27E-06 | 8.63E-05 |
| FABP6 | 2.2462774 | 0.4637589 | 4.8436323 | 1.27E-06 | 8.65E-05 |
| DYDC1 | 2.3727871 | 0.4898801 | 4.8436081 | 1.28E-06 | 8.65E-05 |
| CCDC160 | 1.9659273 | 0.405909 | 4.8432712 | 1.28E-06 | 8.65E-05 |
| HABP2 | 3.2324931 | 0.6676949 | 4.841273 | 1.29E-06 | 8.71E-05 |
| TUBA3FP | 1.7977579 | 0.3718143 | 4.835096 | 1.33E-06 | 8.96E-05 |
| TCTEX1D1 | 2.1119259 | 0.4368611 | 4.8343192 | 1.34E-06 | 8.98E-05 |
| IGKV3-20 | 2.5734798 | 0.5325578 | 4.8323013 | 1.35E-06 | 9.01E-05 |
| DNAH12 | 2.2442579 | 0.4644536 | 4.8320391 | 1.35E-06 | 9.01E-05 |
| PRR29 | 2.1067666 | 0.4359301 | 4.8328079 | 1.35E-06 | 9.01E-05 |
| IGHV2-26 | 3.8297934 | 0.7931691 | 4.8284702 | 1.38E-06 | 9.15E-05 |
| FOXA1 | 1.7698656 | 0.3666062 | 4.8277027 | 1.38E-06 | 9.16E-05 |
| AC097374.1 | 2.5017095 | 0.518332 | 4.8264616 | 1.39E-06 | 9.19E-05 |
| OSBPL6 | 1.5413971 | 0.319499 | 4.8244197 | 1.40E-06 | 9.27E-05 |
| CYP2F1 | 2.8104057 | 0.5826882 | 4.8231724 | 1.41E-06 | 9.30E-05 |
| AL133320.2 | 2.4183389 | 0.5015437 | 4.8217907 | 1.42E-06 | 9.33E-05 |
| MORN2 | 1.7194943 | 0.3566312 | 4.8214912 | 1.42E-06 | 9.33E-05 |
| CHIT1 | 4.3473429 | 0.902099 | 4.8191416 | 1.44E-06 | 9.39E-05 |
| AC007681.1 | 1.9895604 | 0.4129888 | 4.8174685 | 1.45E-06 | 9.39E-05 |
| GOLM1 | 1.194622 | 0.2479905 | 4.8172091 | 1.46E-06 | 9.39E-05 |
| CCDC153 | 1.9352699 | 0.4016417 | 4.8183984 | 1.45E-06 | 9.39E-05 |
| CFAP73 | 2.1582782 | 0.4478955 | 4.8187091 | 1.44E-06 | 9.39E-05 |
| TFAP2B | 4.7253769 | 0.9809448 | 4.8171691 | 1.46E-06 | 9.39E-05 |
| TSGA10 | 1.8307661 | 0.3801222 | 4.8162569 | 1.46E-06 | 9.41E-05 |
| CDH26 | 1.720094 | 0.3577683 | 4.8078432 | 1.53E-06 | 9.80E-05 |
| MORN3 | 2.0415677 | 0.4248768 | 4.8050819 | 1.55E-06 | 9.88E-05 |
| IGHV2-5 | 3.5279561 | 0.7341546 | 4.8054673 | 1.54E-06 | 9.88E-05 |
| PRDM8 | 1.5412018 | 0.3208381 | 4.803674 | 1.56E-06 | 9.93E-05 |
| DIO2 | 2.5262423 | 0.5259977 | 4.8027631 | 1.56E-06 | 9.95E-05 |
| IL1B | -1.879107 | 0.3913 | -4.802216 | 1.57E-06 | 9.95E-05 |
| CFAP100 | 2.2536618 | 0.469501 | 4.8001211 | 1.59E-06 | 0.0001003 |
| LRRC34 | 1.6484352 | 0.3436928 | 4.7962461 | 1.62E-06 | 0.000102 |
| LRRC6 | 1.8103473 | 0.3779429 | 4.7900025 | 1.67E-06 | 0.000105 |
| AL031283.1 | 2.0263921 | 0.4233843 | 4.7861766 | 1.70E-06 | 0.0001068 |
| FAM229B | 1.4843983 | 0.3101948 | 4.7853748 | 1.71E-06 | 0.0001069 |
| PLCH2 | 1.663486 | 0.3476964 | 4.7843061 | 1.72E-06 | 0.0001073 |
| RET | 1.6995546 | 0.355481 | 4.7810005 | 1.74E-06 | 0.0001088 |
| LRRC71 | 2.211259 | 0.4627109 | 4.7789214 | 1.76E-06 | 0.0001094 |
| PFN2 | 1.3307784 | 0.2785613 | 4.7773274 | 1.78E-06 | 0.00011 |
| POU2AF1 | 2.3312371 | 0.4880462 | 4.7766734 | 1.78E-06 | 0.0001101 |
| BEST4 | 2.0918014 | 0.4383334 | 4.7721694 | 1.82E-06 | 0.0001118 |
| AC008674.1 | 2.6942359 | 0.5645166 | 4.7726421 | 1.82E-06 | 0.0001118 |
| TTLL9 | 2.2042919 | 0.4618452 | 4.772794 | 1.82E-06 | 0.0001118 |
| CCDC170 | 2.0114658 | 0.4217741 | 4.7690593 | 1.85E-06 | 0.0001133 |
| CACNG6 | 2.3541939 | 0.4937608 | 4.7678831 | 1.86E-06 | 0.0001136 |
| KIAA2012 | 2.522141 | 0.5294367 | 4.7638195 | 1.90E-06 | 0.0001152 |
| EFCAB10 | 2.1589537 | 0.4531528 | 4.7642952 | 1.90E-06 | 0.0001152 |
| IGHV1-46 | 2.7450081 | 0.5761857 | 4.764103 | 1.90E-06 | 0.0001152 |
| IGLV3-19 | 2.9695721 | 0.6240546 | 4.7585132 | 1.95E-06 | 0.0001179 |
| COL10A1 | 3.885416 | 0.8168578 | 4.7565389 | 1.97E-06 | 0.0001188 |
| C4orf47 | 2.1649145 | 0.4552109 | 4.7558489 | 1.98E-06 | 0.000119 |
| CCDC96 | 2.0743818 | 0.4364519 | 4.75283 | 2.01E-06 | 0.0001205 |
| CCDC138 | 1.5363423 | 0.3233223 | 4.7517364 | 2.02E-06 | 0.0001206 |
| LCN2 | 4.3333649 | 0.9119722 | 4.7516416 | 2.02E-06 | 0.0001206 |
| AC137834.1 | 6.5176021 | 1.3719871 | 4.7504833 | 2.03E-06 | 0.0001208 |
| MIR223HG | -1.768777 | 0.3723404 | -4.75043 | 2.03E-06 | 0.0001208 |
| KIF6 | 1.9138657 | 0.403154 | 4.7472317 | 2.06E-06 | 0.0001224 |
| MASP1 | -1.179775 | 0.2486025 | -4.745627 | 2.08E-06 | 0.0001229 |
| DEGS2 | 1.8325686 | 0.3861488 | 4.745758 | 2.08E-06 | 0.0001229 |
| CETN2 | 1.6350034 | 0.3447778 | 4.7421941 | 2.11E-06 | 0.0001247 |
| MIR205 | 3.514676 | 0.7413816 | 4.7407111 | 2.13E-06 | 0.0001253 |
| C5orf49 | 2.137092 | 0.4509852 | 4.738719 | 2.15E-06 | 0.0001263 |
| LINC02166 | 1.9645861 | 0.414767 | 4.7366013 | 2.17E-06 | 0.0001273 |
| CFAP157 | 2.0972479 | 0.4428403 | 4.7359011 | 2.18E-06 | 0.0001274 |
| IGLC2 | 2.7688825 | 0.584708 | 4.7354964 | 2.19E-06 | 0.0001274 |
| NEK5 | 1.8754579 | 0.3961461 | 4.7342576 | 2.20E-06 | 0.0001279 |
| TSPAN19 | 2.8611607 | 0.6046009 | 4.7323129 | 2.22E-06 | 0.0001289 |
| RPGRIP1L | 1.6135277 | 0.3411538 | 4.729619 | 2.25E-06 | 0.0001303 |
| DPP6 | -2.310236 | 0.4887583 | -4.726746 | 2.28E-06 | 0.0001319 |
| CTHRC1 | 2.1006101 | 0.4445796 | 4.7249357 | 2.30E-06 | 0.0001325 |
| DNAH2 | 2.1038618 | 0.4452966 | 4.7246307 | 2.31E-06 | 0.0001325 |
| CCDC103 | 2.1790405 | 0.4612265 | 4.724448 | 2.31E-06 | 0.0001325 |
| LGR5 | 1.7391552 | 0.3685134 | 4.7193809 | 2.37E-06 | 0.0001355 |
| HYDIN | 2.0467356 | 0.4337354 | 4.7188572 | 2.37E-06 | 0.0001356 |
| AGR2 | 1.6318222 | 0.3459682 | 4.7166825 | 2.40E-06 | 0.0001367 |
| RHOV | 2.4622888 | 0.5221654 | 4.7155341 | 2.41E-06 | 0.0001372 |
| CFAP221 | 1.7870736 | 0.3791982 | 4.7127688 | 2.44E-06 | 0.0001388 |
| SPACA9 | 1.9261999 | 0.4091446 | 4.7078703 | 2.50E-06 | 0.0001418 |
| SPAG16 | 1.3437251 | 0.2854623 | 4.7071892 | 2.51E-06 | 0.000142 |
| KLK11 | 1.4506938 | 0.3082839 | 4.7057078 | 2.53E-06 | 0.0001427 |
| TNFAIP8L1 | 1.5574395 | 0.3310223 | 4.7049376 | 2.54E-06 | 0.000143 |
| IL5RA | 1.9802984 | 0.4213718 | 4.6996466 | 2.61E-06 | 0.0001464 |
| CNTN5 | 2.463919 | 0.5243798 | 4.6987296 | 2.62E-06 | 0.0001466 |
| LRRC74B | 2.1968801 | 0.4675645 | 4.6985604 | 2.62E-06 | 0.0001466 |
| ERICH6-AS1 | 1.9163245 | 0.4079901 | 4.6969875 | 2.64E-06 | 0.0001474 |
| EDARADD | 1.6623518 | 0.3539921 | 4.696014 | 2.65E-06 | 0.0001478 |
| C16orf71 | 2.1956285 | 0.4677673 | 4.6938473 | 2.68E-06 | 0.000149 |
| C5orf46 | 2.8586463 | 0.6091449 | 4.6928838 | 2.69E-06 | 0.0001494 |
| AL390755.2 | 2.4063479 | 0.512939 | 4.6912944 | 2.71E-06 | 0.0001502 |
| IQANK1 | 1.8482266 | 0.3940534 | 4.6902952 | 2.73E-06 | 0.0001504 |
| CCL11 | 2.0021419 | 0.4268778 | 4.6901993 | 2.73E-06 | 0.0001504 |
| SMIM31 | 2.8708368 | 0.6123261 | 4.6884117 | 2.75E-06 | 0.0001514 |
| LINC01267 | 2.4045774 | 0.5131213 | 4.6861776 | 2.78E-06 | 0.0001524 |
| SST | 6.925975 | 1.4781049 | 4.6857129 | 2.79E-06 | 0.0001524 |
| HSPA4L | 1.9046889 | 0.4064384 | 4.6862917 | 2.78E-06 | 0.0001524 |
| TPH1 | 1.361116 | 0.2905056 | 4.6853342 | 2.80E-06 | 0.0001524 |
| IGHV3-15 | 2.6391082 | 0.5634705 | 4.6836667 | 2.82E-06 | 0.0001533 |
| AP001189.1 | -2.748126 | 0.5870739 | -4.681056 | 2.85E-06 | 0.000155 |
| BMP6 | -1.558054 | 0.3331078 | -4.677326 | 2.91E-06 | 0.0001575 |
| CYP2W1 | 2.7031332 | 0.5782939 | 4.6743239 | 2.95E-06 | 0.0001586 |
| DNAAF1 | 2.1094734 | 0.4512547 | 4.6746849 | 2.94E-06 | 0.0001586 |
| CPNE7 | 1.9204669 | 0.4107882 | 4.6750779 | 2.94E-06 | 0.0001586 |
| AL354766.2 | 6.4459453 | 1.3790406 | 4.6742244 | 2.95E-06 | 0.0001586 |
| CD83 | -1.060162 | 0.2268355 | -4.673705 | 2.96E-06 | 0.0001587 |
| GRHL1 | 1.668261 | 0.3570427 | 4.6724406 | 2.98E-06 | 0.0001593 |
| DNAL1 | 1.2361436 | 0.2646392 | 4.6710521 | 3.00E-06 | 0.0001601 |
| AP001830.1 | 1.8378174 | 0.3935029 | 4.6704031 | 3.01E-06 | 0.0001601 |
| PCSK1N | 1.9766655 | 0.4232502 | 4.6702056 | 3.01E-06 | 0.0001601 |
| AL353747.3 | 8.1295934 | 1.7410099 | 4.6694698 | 3.02E-06 | 0.0001603 |
| CFAP47 | 2.1324318 | 0.4571304 | 4.6648215 | 3.09E-06 | 0.0001636 |
| KLHDC7A | 2.0575129 | 0.441174 | 4.6637222 | 3.11E-06 | 0.0001641 |
| IGKV3-15 | 2.7664152 | 0.5932238 | 4.6633581 | 3.11E-06 | 0.0001641 |
| ARMH2 | 2.3264355 | 0.4989969 | 4.6622241 | 3.13E-06 | 0.0001647 |
| KCTD1 | 1.5291192 | 0.3281531 | 4.6597731 | 3.17E-06 | 0.0001664 |
| USP2 | 1.7887386 | 0.3845222 | 4.6518474 | 3.29E-06 | 0.0001725 |
| CTXN1 | 2.2080175 | 0.4746922 | 4.6514724 | 3.30E-06 | 0.0001725 |
| TMEM144 | 1.0517186 | 0.2262822 | 4.647819 | 3.35E-06 | 0.0001752 |
| TMEM130 | 2.1058723 | 0.4532123 | 4.6465468 | 3.38E-06 | 0.000176 |
| ARHGAP39 | 1.6744582 | 0.360449 | 4.6454783 | 3.39E-06 | 0.0001765 |
| KIAA0319 | 2.0828669 | 0.448572 | 4.6433282 | 3.43E-06 | 0.000178 |
| DNAH10 | 2.0637063 | 0.4445018 | 4.6427396 | 3.44E-06 | 0.0001782 |
| CCDC189 | 2.1263095 | 0.458156 | 4.6410163 | 3.47E-06 | 0.0001793 |
| AC139100.1 | 1.9494903 | 0.4201272 | 4.6402383 | 3.48E-06 | 0.0001796 |
| MOBP | 2.135326 | 0.4602447 | 4.6395445 | 3.49E-06 | 0.0001799 |
| CFAP74 | 2.0318518 | 0.4380653 | 4.6382399 | 3.51E-06 | 0.0001807 |
| CFAP99 | 2.2035524 | 0.4751653 | 4.6374435 | 3.53E-06 | 0.000181 |
| TSNAXIP1 | 1.9354272 | 0.4173885 | 4.6369921 | 3.54E-06 | 0.000181 |
| BBOF1 | 1.7720119 | 0.3823266 | 4.6348124 | 3.57E-06 | 0.0001826 |
| MCIDAS | 2.9077966 | 0.6277397 | 4.6321691 | 3.62E-06 | 0.0001846 |
| CT45A9 | -4.250645 | 0.9178903 | -4.630886 | 3.64E-06 | 0.0001854 |
| KRT17 | 3.3750267 | 0.7289021 | 4.6302882 | 3.65E-06 | 0.0001855 |
| RGS9 | -1.51865 | 0.3281102 | -4.628475 | 3.68E-06 | 0.0001868 |
| CCDC181 | 2.1972062 | 0.4754549 | 4.6212716 | 3.81E-06 | 0.000193 |
| TMEM59L | 2.0349138 | 0.4405746 | 4.6187721 | 3.86E-06 | 0.000195 |
| CYP4X1 | 1.4105314 | 0.3054922 | 4.6172421 | 3.89E-06 | 0.0001961 |
| MROH9 | 2.3352191 | 0.5058559 | 4.6163724 | 3.91E-06 | 0.0001961 |
| IGLJ2 | 2.6640264 | 0.5770851 | 4.6163496 | 3.91E-06 | 0.0001961 |
| WHRN | 1.6190327 | 0.350831 | 4.614851 | 3.93E-06 | 0.0001972 |
| IGLV6-57 | 3.8933035 | 0.8442525 | 4.6115391 | 4.00E-06 | 0.0002 |
| AC233755.2 | 3.9425621 | 0.8553509 | 4.6092918 | 4.04E-06 | 0.0002018 |
| TRIM17 | 1.3786903 | 0.2993149 | 4.6061535 | 4.10E-06 | 0.0002044 |
| ANKRD44-AS1 | 2.1946915 | 0.4770251 | 4.6007879 | 4.21E-06 | 0.000209 |
| MUC2 | 4.4444541 | 0.9670852 | 4.5957214 | 4.31E-06 | 0.0002137 |
| MAP6 | 1.492739 | 0.3248395 | 4.5953123 | 4.32E-06 | 0.0002137 |
| BAAT | 2.9441736 | 0.6409352 | 4.5935589 | 4.36E-06 | 0.0002151 |
| SOX2-OT | 1.5307844 | 0.3334719 | 4.5904449 | 4.42E-06 | 0.0002175 |
| CCDC187 | 2.1694766 | 0.4725934 | 4.5905779 | 4.42E-06 | 0.0002175 |
| KLK12 | 2.573637 | 0.5610264 | 4.587372 | 4.49E-06 | 0.0002203 |
| OXTR | 2.2012034 | 0.4802253 | 4.5836894 | 4.57E-06 | 0.0002238 |
| AC004832.1 | 2.3718779 | 0.5179302 | 4.5795323 | 4.66E-06 | 0.0002279 |
| AC013470.2 | 2.4125508 | 0.5268656 | 4.5790629 | 4.67E-06 | 0.000228 |
| GPR87 | 3.778079 | 0.8253611 | 4.5774859 | 4.71E-06 | 0.0002293 |
| IGKV1D-33 | 3.0413672 | 0.6651608 | 4.5723785 | 4.82E-06 | 0.0002341 |
| PDE1C | -1.27268 | 0.2783259 | -4.572625 | 4.82E-06 | 0.0002341 |
| KLHL13 | 1.6042636 | 0.3510329 | 4.5701232 | 4.87E-06 | 0.0002362 |
| TTC16 | 1.9951335 | 0.4366805 | 4.5688633 | 4.90E-06 | 0.0002371 |
| ACSBG1 | 1.9479631 | 0.4266141 | 4.5661016 | 4.97E-06 | 0.0002398 |
| SPAG17 | 2.0761103 | 0.45479 | 4.5649871 | 5.00E-06 | 0.0002407 |
| B9D1 | 1.7264837 | 0.3783185 | 4.5635724 | 5.03E-06 | 0.0002419 |
| PROM2 | 1.1305423 | 0.2479744 | 4.5591087 | 5.14E-06 | 0.0002466 |
| ESRRG | 1.7036 | 0.3737612 | 4.5579907 | 5.16E-06 | 0.0002475 |
| AC084871.4 | -1.627861 | 0.3572514 | -4.556625 | 5.20E-06 | 0.0002486 |
| ANKRD66 | 2.2624262 | 0.496884 | 4.5532281 | 5.28E-06 | 0.0002502 |
| AL390778.2 | 2.6189988 | 0.5750797 | 4.5541496 | 5.26E-06 | 0.0002502 |
| IGHV4-39 | 2.7403035 | 0.6018295 | 4.5532889 | 5.28E-06 | 0.0002502 |
| MAP1A | 1.8332582 | 0.4025194 | 4.5544596 | 5.25E-06 | 0.0002502 |
| ELL3 | 1.5913517 | 0.3494821 | 4.5534566 | 5.28E-06 | 0.0002502 |
| IGLL5 | 2.6025399 | 0.5716149 | 4.5529606 | 5.29E-06 | 0.0002502 |
| HOXB2 | 1.1490868 | 0.2524566 | 4.5516213 | 5.32E-06 | 0.0002514 |
| IGHG1 | 3.2706466 | 0.7188587 | 4.5497765 | 5.37E-06 | 0.0002527 |
| UCKL1-AS1 | 2.0302014 | 0.446186 | 4.5501238 | 5.36E-06 | 0.0002527 |
| NRAD1 | 2.1466992 | 0.4720654 | 4.5474616 | 5.43E-06 | 0.000255 |
| DNER | 2.4795497 | 0.5456438 | 4.5442643 | 5.51E-06 | 0.0002583 |
| CFAP54 | 1.8359054 | 0.4040277 | 4.5440091 | 5.52E-06 | 0.0002583 |
| WDR31 | 1.4328562 | 0.3155235 | 4.541203 | 5.59E-06 | 0.0002608 |
| AC073257.2 | 2.3269446 | 0.5126725 | 4.5388522 | 5.66E-06 | 0.0002628 |
| LRGUK | 1.7794118 | 0.3922035 | 4.5369611 | 5.71E-06 | 0.0002641 |
| ITPRIP | -1.418903 | 0.3127311 | -4.537136 | 5.70E-06 | 0.0002641 |
| AC011407.1 | 7.0066127 | 1.544422 | 4.5367215 | 5.71E-06 | 0.0002641 |
| LINC01206 | 6.5023112 | 1.433481 | 4.5360288 | 5.73E-06 | 0.0002644 |
| NRG4 | 2.123866 | 0.4682557 | 4.5356968 | 5.74E-06 | 0.0002644 |
| IGHJ3 | 3.0713536 | 0.6773317 | 4.5344895 | 5.77E-06 | 0.0002655 |
| GIPR | 2.0014601 | 0.441475 | 4.533575 | 5.80E-06 | 0.0002661 |
| PAPPA2 | -1.935673 | 0.4274019 | -4.52893 | 5.93E-06 | 0.0002715 |
| LGALSL | -1.148192 | 0.2535629 | -4.528233 | 5.95E-06 | 0.0002715 |
| SOX7 | -1.756077 | 0.3877854 | -4.528477 | 5.94E-06 | 0.0002715 |
| SAMD15 | 1.9678118 | 0.4350943 | 4.5227244 | 6.10E-06 | 0.0002782 |
| DOC2A | 2.1582633 | 0.4778028 | 4.5170589 | 6.27E-06 | 0.0002852 |
| AC005041.1 | 2.6774744 | 0.592862 | 4.5161847 | 6.30E-06 | 0.0002859 |
| ATG9B | 1.8347828 | 0.4063398 | 4.5153903 | 6.32E-06 | 0.0002865 |
| B3GNT8 | -1.391148 | 0.3081307 | -4.514799 | 6.34E-06 | 0.0002868 |
| SLC44A4 | 2.0360398 | 0.4511225 | 4.5132749 | 6.38E-06 | 0.0002884 |
| ZNF214 | 1.3032592 | 0.2888691 | 4.5115908 | 6.43E-06 | 0.0002902 |
| VIPR1 | -2.045361 | 0.4539924 | -4.505275 | 6.63E-06 | 0.0002977 |
| ARC | -1.898452 | 0.4214101 | -4.505 | 6.64E-06 | 0.0002977 |
| IGHG4 | 2.8556418 | 0.6338051 | 4.5055522 | 6.62E-06 | 0.0002977 |
| COMP | 3.1842969 | 0.7068841 | 4.5046943 | 6.65E-06 | 0.0002977 |
| HID1-AS1 | -1.324273 | 0.2940874 | -4.502992 | 6.70E-06 | 0.0002996 |
| AC004923.1 | 2.316972 | 0.5146179 | 4.5023151 | 6.72E-06 | 0.0003 |
| HCAR1 | 2.001835 | 0.4449197 | 4.4993176 | 6.82E-06 | 0.0003038 |
| CCDC24 | 1.6425965 | 0.3652955 | 4.4966238 | 6.90E-06 | 0.0003071 |
| PLPPR3 | 1.8290264 | 0.4069121 | 4.4948937 | 6.96E-06 | 0.0003091 |
| SLC26A4 | 3.2198263 | 0.7167733 | 4.4921128 | 7.05E-06 | 0.0003126 |
| ADRB2 | -1.277469 | 0.2844554 | -4.490928 | 7.09E-06 | 0.0003133 |
| DPY19L2P2 | 1.9614892 | 0.4371002 | 4.4875052 | 7.21E-06 | 0.0003173 |
| RHPN1 | 1.3594022 | 0.3029169 | 4.487706 | 7.20E-06 | 0.0003173 |
| AL121899.2 | 2.2743929 | 0.5068743 | 4.487095 | 7.22E-06 | 0.0003174 |
| HOXB3 | 1.1726956 | 0.2614894 | 4.4846777 | 7.30E-06 | 0.0003204 |
| LY6H | 3.1491849 | 0.7026809 | 4.4816714 | 7.41E-06 | 0.000324 |
| LINC00683 | 1.5544118 | 0.3468425 | 4.4816066 | 7.41E-06 | 0.000324 |
| AC073439.1 | 2.1453748 | 0.4791969 | 4.4770214 | 7.57E-06 | 0.0003299 |
| TTLL6 | 1.8103219 | 0.4043569 | 4.47704 | 7.57E-06 | 0.0003299 |
| DLEC1 | 1.9068813 | 0.4259955 | 4.4762948 | 7.59E-06 | 0.00033 |
| TTC23L | 1.8252242 | 0.407753 | 4.4762986 | 7.59E-06 | 0.00033 |
| ADGRA1 | 2.5675515 | 0.5737738 | 4.47485 | 7.65E-06 | 0.0003316 |
| ECT2L | 2.033556 | 0.4546311 | 4.4729807 | 7.71E-06 | 0.000334 |
| AL117382.2 | 2.2762248 | 0.509287 | 4.4694346 | 7.84E-06 | 0.000339 |
| IGKJ4 | 3.4027839 | 0.7619084 | 4.4661326 | 7.96E-06 | 0.0003437 |
| HMGB3 | 1.3287527 | 0.2976173 | 4.4646358 | 8.02E-06 | 0.0003456 |
| AL590226.2 | 1.8056416 | 0.4045954 | 4.4628325 | 8.09E-06 | 0.000347 |
| ZFHX2 | 1.7401648 | 0.3899305 | 4.4627567 | 8.09E-06 | 0.000347 |
| HSD17B2 | 2.2919095 | 0.5135743 | 4.4626636 | 8.09E-06 | 0.000347 |
| CRISPLD1 | 1.3564776 | 0.3040712 | 4.4610517 | 8.16E-06 | 0.0003491 |
| AF064858.1 | -1.603322 | 0.3594453 | -4.460546 | 8.18E-06 | 0.0003493 |
| PAX9 | 3.1614521 | 0.7088574 | 4.4599269 | 8.20E-06 | 0.0003498 |
| AC010624.3 | 1.973177 | 0.4425853 | 4.4582975 | 8.26E-06 | 0.0003513 |
| LINC02362 | 2.510826 | 0.5633362 | 4.4570652 | 8.31E-06 | 0.0003524 |
| AC015802.7 | 2.274363 | 0.5102957 | 4.4569514 | 8.31E-06 | 0.0003524 |
| BPIFB1 | 4.950905 | 1.1110379 | 4.456108 | 8.35E-06 | 0.0003532 |
| DNAJC12 | 1.6136064 | 0.3623335 | 4.453374 | 8.45E-06 | 0.0003571 |
| RPL13AP17 | 2.3045055 | 0.5176202 | 4.4521167 | 8.50E-06 | 0.0003586 |
| ADGRF1 | 3.9674661 | 0.8915369 | 4.4501423 | 8.58E-06 | 0.0003608 |
| SYT16 | 2.3874531 | 0.5364798 | 4.4502199 | 8.58E-06 | 0.0003608 |
| WDR49 | 1.9701341 | 0.4431201 | 4.4460495 | 8.75E-06 | 0.0003665 |
| HCN4 | 2.1974751 | 0.494297 | 4.4456577 | 8.76E-06 | 0.0003666 |
| MSI2 | 1.1689879 | 0.2631142 | 4.4428915 | 8.88E-06 | 0.0003708 |
| CDC20B | 2.3821845 | 0.5363527 | 4.4414511 | 8.94E-06 | 0.0003727 |
| SLC22A4 | 2.0598273 | 0.4642025 | 4.437346 | 9.11E-06 | 0.0003786 |
| SMIM34B | 2.0249624 | 0.4563271 | 4.4375234 | 9.10E-06 | 0.0003786 |
| AMPD3 | 1.4178078 | 0.3196213 | 4.4358995 | 9.17E-06 | 0.0003806 |
| AL357093.1 | 2.4476032 | 0.5519303 | 4.4346235 | 9.22E-06 | 0.0003822 |
| HHATL | 2.1135181 | 0.4766998 | 4.4336464 | 9.27E-06 | 0.0003833 |
| MEIG1 | 2.2562119 | 0.509036 | 4.4323226 | 9.32E-06 | 0.0003851 |
| FLACC1 | 1.9973464 | 0.4507944 | 4.4307256 | 9.39E-06 | 0.0003867 |
| ERICH3 | 2.1245608 | 0.4799967 | 4.426199 | 9.59E-06 | 0.0003942 |
| EYA2 | 2.0889201 | 0.4719761 | 4.4259022 | 9.60E-06 | 0.0003942 |
| IGLV1-40 | 2.6651596 | 0.6022482 | 4.4253509 | 9.63E-06 | 0.0003946 |
| MYCBPAP | 2.0087587 | 0.4539587 | 4.4249811 | 9.65E-06 | 0.0003947 |
| AC005821.1 | 2.1799298 | 0.4933073 | 4.4190101 | 9.92E-06 | 0.0004051 |
| AL121790.2 | 3.1776624 | 0.7193737 | 4.4172622 | 1.00E-05 | 0.0004078 |
| CCDC13 | 2.4724008 | 0.5600524 | 4.4145884 | 1.01E-05 | 0.0004119 |
| LTF | 3.430537 | 0.7771293 | 4.4143708 | 1.01E-05 | 0.0004119 |
| DNAAF4 | 1.5178592 | 0.3440264 | 4.4120433 | 1.02E-05 | 0.0004158 |
| CDH2 | 1.3366809 | 0.3031493 | 4.4093148 | 1.04E-05 | 0.0004197 |
| SPAG1 | 1.4512441 | 0.3297041 | 4.4016559 | 1.07E-05 | 0.0004341 |
| CASC2 | 1.6571618 | 0.3765673 | 4.400706 | 1.08E-05 | 0.0004354 |
| IGHV3-48 | 2.7711682 | 0.6298402 | 4.3997958 | 1.08E-05 | 0.0004365 |
| AC127070.2 | 1.8928204 | 0.4304501 | 4.397305 | 1.10E-05 | 0.0004409 |
| HOXB-AS1 | 1.1269661 | 0.256479 | 4.3939907 | 1.11E-05 | 0.0004463 |
| C22orf23 | 1.6397606 | 0.373402 | 4.3914084 | 1.13E-05 | 0.0004509 |
| NRG3 | -1.771897 | 0.4035291 | -4.391002 | 1.13E-05 | 0.0004511 |
| IGLV3-21 | 2.8760312 | 0.6552947 | 4.3889125 | 1.14E-05 | 0.0004547 |
| WDR54 | 1.6937107 | 0.3860174 | 4.3876533 | 1.15E-05 | 0.0004567 |
| PARD6G-AS1 | 1.6501117 | 0.3761491 | 4.3868552 | 1.15E-05 | 0.0004577 |
| SLC23A1 | 2.1916359 | 0.4996504 | 4.3863384 | 1.15E-05 | 0.000458 |
| GDF7 | 1.3429861 | 0.306288 | 4.3847173 | 1.16E-05 | 0.0004608 |
| RTN4RL1 | 1.3251986 | 0.3024222 | 4.3819483 | 1.18E-05 | 0.000466 |
| F2RL2 | 1.8192814 | 0.415361 | 4.3800001 | 1.19E-05 | 0.0004692 |
| VSTM2L | 1.7342793 | 0.3959742 | 4.3797788 | 1.19E-05 | 0.0004692 |
| NSUN7 | 1.8838202 | 0.4303301 | 4.3776165 | 1.20E-05 | 0.0004731 |
| IQCK | 1.6581377 | 0.3788458 | 4.3768141 | 1.20E-05 | 0.0004742 |
| SLC45A3 | 1.5567967 | 0.355796 | 4.3755321 | 1.21E-05 | 0.0004763 |
| LINC00900 | 1.2001559 | 0.2744483 | 4.372977 | 1.23E-05 | 0.0004811 |
| AL138787.1 | 2.722919 | 0.6232099 | 4.3691843 | 1.25E-05 | 0.0004888 |
| AC026801.2 | 1.5765033 | 0.360945 | 4.3677109 | 1.26E-05 | 0.0004914 |
| MAPK10 | 1.1677351 | 0.2675473 | 4.3645935 | 1.27E-05 | 0.0004977 |
| AC122719.3 | 2.4940058 | 0.5719614 | 4.360444 | 1.30E-05 | 0.0005065 |
| IGKV2D-28 | 2.6965754 | 0.6185101 | 4.3597923 | 1.30E-05 | 0.0005072 |
| IGHG3 | 3.2335323 | 0.7417957 | 4.3590605 | 1.31E-05 | 0.0005082 |
| AC105052.5 | 2.1508599 | 0.4935183 | 4.3582173 | 1.31E-05 | 0.0005094 |
| FMN2 | 2.090937 | 0.4799123 | 4.3569148 | 1.32E-05 | 0.000511 |
| ABCB1 | 2.152947 | 0.4941483 | 4.3568844 | 1.32E-05 | 0.000511 |
| ACTBL2 | 6.1801223 | 1.4195242 | 4.3536576 | 1.34E-05 | 0.0005178 |
| ENTPD3 | 1.1080396 | 0.2546738 | 4.3508192 | 1.36E-05 | 0.0005234 |
| C11orf97 | 2.0664136 | 0.4749675 | 4.3506419 | 1.36E-05 | 0.0005234 |
| SMIM22 | 1.7502432 | 0.4023601 | 4.3499421 | 1.36E-05 | 0.0005243 |
| LINC00326 | 4.5049634 | 1.0362049 | 4.3475604 | 1.38E-05 | 0.0005292 |
| TREM1 | -1.351704 | 0.3111493 | -4.344229 | 1.40E-05 | 0.0005365 |
| DNALI1 | 1.4538343 | 0.3347334 | 4.3432606 | 1.40E-05 | 0.0005381 |
| DNAH3 | 2.2875708 | 0.5268241 | 4.3421907 | 1.41E-05 | 0.00054 |
| IFITM10 | 1.6547125 | 0.3811558 | 4.3413021 | 1.42E-05 | 0.0005414 |
| AC073464.1 | 6.0283744 | 1.3889006 | 4.3403931 | 1.42E-05 | 0.0005428 |
| IGKV1-5 | 2.5623615 | 0.590656 | 4.3381623 | 1.44E-05 | 0.0005461 |
| Z99496.2 | 3.5313012 | 0.8140206 | 4.3380982 | 1.44E-05 | 0.0005461 |
| FAM227A | 1.9141717 | 0.4412248 | 4.3383138 | 1.44E-05 | 0.0005461 |
| SHC4 | 1.4427233 | 0.3327548 | 4.3356943 | 1.45E-05 | 0.0005513 |
| HEPACAM2 | 2.5689126 | 0.5927686 | 4.3337525 | 1.47E-05 | 0.0005554 |
| FAM83A-AS1 | 5.8991675 | 1.361649 | 4.3323701 | 1.48E-05 | 0.0005581 |
| FANCF | 1.1330349 | 0.2615542 | 4.3319318 | 1.48E-05 | 0.0005584 |
| BX005040.1 | 2.262396 | 0.5223456 | 4.3312243 | 1.48E-05 | 0.0005594 |
| SERPINI2 | 1.839773 | 0.4250144 | 4.3287308 | 1.50E-05 | 0.0005637 |
| AC106818.2 | 2.8183924 | 0.6510336 | 4.3291042 | 1.50E-05 | 0.0005637 |
| PNMA8A | 1.7352903 | 0.400892 | 4.3285734 | 1.50E-05 | 0.0005637 |
| TIGD4 | 1.8813261 | 0.4346686 | 4.328185 | 1.50E-05 | 0.0005639 |
| IGHJ4 | 2.7867723 | 0.6441433 | 4.3263236 | 1.52E-05 | 0.0005678 |
| ATP6V1C2 | 1.1071159 | 0.2562635 | 4.3202249 | 1.56E-05 | 0.000582 |
| FAM238C | 1.835276 | 0.4248385 | 4.3199383 | 1.56E-05 | 0.000582 |
| MAPK15 | 1.9442357 | 0.4502229 | 4.3183848 | 1.57E-05 | 0.0005853 |
| PLEKHS1 | 3.2451347 | 0.751808 | 4.3164409 | 1.59E-05 | 0.0005896 |
| TSPAN6 | 1.0232869 | 0.2371936 | 4.3141428 | 1.60E-05 | 0.0005949 |
| AC020663.1 | 6.2382481 | 1.4465976 | 4.3123588 | 1.62E-05 | 0.0005989 |
| KIF21A | 1.5612434 | 0.3622758 | 4.3095437 | 1.64E-05 | 0.0006057 |
| DZIP1L | 1.6025535 | 0.3721712 | 4.3059582 | 1.66E-05 | 0.0006139 |
| MMP1 | 5.215457 | 1.2111618 | 4.3061605 | 1.66E-05 | 0.0006139 |
| SOX21 | 3.2007031 | 0.7433865 | 4.3055708 | 1.67E-05 | 0.0006141 |
| AC110998.1 | 2.0374907 | 0.4734503 | 4.3034944 | 1.68E-05 | 0.0006181 |
| REEP6 | 1.7277143 | 0.401652 | 4.3015207 | 1.70E-05 | 0.0006219 |
| AC084880.1 | -1.869539 | 0.4346797 | -4.300958 | 1.70E-05 | 0.0006226 |
| TMEM184A | 1.3175644 | 0.3066897 | 4.296083 | 1.74E-05 | 0.0006338 |
| CFAP70 | 1.80521 | 0.4201956 | 4.2961187 | 1.74E-05 | 0.0006338 |
| FAM181A-AS1 | 2.6573681 | 0.618749 | 4.2947433 | 1.75E-05 | 0.0006367 |
| BSCL2 | 1.1662735 | 0.2718505 | 4.2901291 | 1.79E-05 | 0.0006492 |
| KIF1A | 1.595542 | 0.3721888 | 4.2869157 | 1.81E-05 | 0.0006568 |
| CEP126 | 1.6240832 | 0.3788386 | 4.2870053 | 1.81E-05 | 0.0006568 |
| RGS17 | 1.302146 | 0.3038073 | 4.2860914 | 1.82E-05 | 0.0006583 |
| GON7 | 1.293873 | 0.3020011 | 4.2843325 | 1.83E-05 | 0.0006626 |
| CFAP46 | 1.9363538 | 0.4522815 | 4.2813024 | 1.86E-05 | 0.0006708 |
| HS3ST6 | 3.4025107 | 0.7950381 | 4.2796827 | 1.87E-05 | 0.0006748 |
| CAPS2 | 1.4090641 | 0.3294383 | 4.2771714 | 1.89E-05 | 0.0006815 |
| TESMIN | 1.8543226 | 0.4337641 | 4.2749564 | 1.91E-05 | 0.0006869 |
| HAP1 | -1.530828 | 0.3581304 | -4.274498 | 1.92E-05 | 0.0006869 |
| FUT2 | 1.841958 | 0.430912 | 4.2745572 | 1.92E-05 | 0.0006869 |
| COLCA1 | 1.3251453 | 0.3100775 | 4.2735939 | 1.92E-05 | 0.0006878 |
| IGHV3-33 | 2.732961 | 0.6395471 | 4.2732758 | 1.93E-05 | 0.0006878 |
| AC015813.8 | 1.9469923 | 0.4555894 | 4.2735684 | 1.92E-05 | 0.0006878 |
| TTC26 | 1.5427898 | 0.3612223 | 4.2710257 | 1.95E-05 | 0.0006938 |
| AC105916.2 | 1.8288464 | 0.4283673 | 4.269342 | 1.96E-05 | 0.0006962 |
| CDKN2B-AS1 | 1.9196784 | 0.4496332 | 4.2694324 | 1.96E-05 | 0.0006962 |
| SMIM6 | 1.9213362 | 0.449987 | 4.2697598 | 1.96E-05 | 0.0006962 |
| MAPRE3 | 1.4197609 | 0.3326123 | 4.2685164 | 1.97E-05 | 0.0006969 |
| VWA5B2 | 2.2834679 | 0.5349954 | 4.2682007 | 1.97E-05 | 0.0006969 |
| WNT16 | 2.741543 | 0.6423116 | 4.2682447 | 1.97E-05 | 0.0006969 |
| CYB5D1 | 1.6105108 | 0.3774155 | 4.2672085 | 1.98E-05 | 0.0006991 |
| LRRC63 | 2.220241 | 0.5203442 | 4.2668697 | 1.98E-05 | 0.0006992 |
| CROCC2 | 1.9722267 | 0.4622586 | 4.2665012 | 1.99E-05 | 0.0006994 |
| CDKN2B | -1.235887 | 0.2898239 | -4.264269 | 2.01E-05 | 0.0007045 |
| CHST6 | 1.9960239 | 0.4680737 | 4.2643367 | 2.00E-05 | 0.0007045 |
| AL354743.2 | 2.0938078 | 0.4911803 | 4.262809 | 2.02E-05 | 0.0007082 |
| KLHL32 | 1.9421821 | 0.4557591 | 4.2614222 | 2.03E-05 | 0.0007117 |
| CEMIP2 | -1.32496 | 0.3109975 | -4.260356 | 2.04E-05 | 0.0007137 |
| IGLV3-1 | 2.7237738 | 0.6393537 | 4.2601989 | 2.04E-05 | 0.0007137 |
| LRRC73 | 2.0231698 | 0.4752677 | 4.2569054 | 2.07E-05 | 0.0007233 |
| DRAIC | 1.8122216 | 0.4258254 | 4.2557856 | 2.08E-05 | 0.0007259 |
| DNAJC9-AS1 | 1.6846844 | 0.3959433 | 4.2548627 | 2.09E-05 | 0.000728 |
| ERICH5 | 1.888244 | 0.4439158 | 4.2536084 | 2.10E-05 | 0.0007302 |
| MYADM | -1.207921 | 0.2839778 | -4.253574 | 2.10E-05 | 0.0007302 |
| RIIAD1 | 1.9371776 | 0.4555647 | 4.252256 | 2.12E-05 | 0.0007335 |
| MCOLN3 | -1.361026 | 0.3200954 | -4.251939 | 2.12E-05 | 0.0007336 |
| STX11 | -1.090943 | 0.2567075 | -4.249751 | 2.14E-05 | 0.0007398 |
| AP003717.1 | 2.035836 | 0.4791873 | 4.2485179 | 2.15E-05 | 0.0007429 |
| SPOCD1 | -1.416054 | 0.3333581 | -4.247846 | 2.16E-05 | 0.0007441 |
| IQUB | 1.8484098 | 0.43517 | 4.247558 | 2.16E-05 | 0.0007441 |
| DPY19L2P1 | 2.1063067 | 0.4966793 | 4.2407785 | 2.23E-05 | 0.000763 |
| IFT22 | 1.3947699 | 0.3288587 | 4.2412436 | 2.22E-05 | 0.000763 |
| ANKK1 | 1.7519051 | 0.4130762 | 4.2411186 | 2.22E-05 | 0.000763 |
| RND2 | 1.448279 | 0.3415136 | 4.2407651 | 2.23E-05 | 0.000763 |
| CCDC180 | 1.6261152 | 0.3835562 | 4.2395745 | 2.24E-05 | 0.0007661 |
| HRK | 1.8662594 | 0.4403043 | 4.2385675 | 2.25E-05 | 0.0007665 |
| IGHV4-61 | 3.3445018 | 0.7890504 | 4.2386417 | 2.25E-05 | 0.0007665 |
| NGFR | 1.5556115 | 0.3669791 | 4.2389643 | 2.25E-05 | 0.0007665 |
| CFAP69 | 1.3730164 | 0.3243437 | 4.2332144 | 2.30E-05 | 0.000783 |
| MUC15 | 1.1756624 | 0.2777665 | 4.2325561 | 2.31E-05 | 0.0007842 |
| MEP1A | 7.0217719 | 1.6599885 | 4.2300124 | 2.34E-05 | 0.0007902 |
| LINC00475 | 2.0992583 | 0.4962692 | 4.2300797 | 2.34E-05 | 0.0007902 |
| ENPP5 | 1.4849978 | 0.3511737 | 4.2286702 | 2.35E-05 | 0.0007937 |
| HS3ST2 | 3.4061281 | 0.8059119 | 4.2264271 | 2.37E-05 | 0.0008006 |
| LMX1B | 2.1140754 | 0.5002524 | 4.2260179 | 2.38E-05 | 0.0008011 |
| AL121899.1 | 2.7212621 | 0.6439992 | 4.2255676 | 2.38E-05 | 0.0008016 |
| SLC26A9 | -1.24846 | 0.2955209 | -4.224609 | 2.39E-05 | 0.000804 |
| CDNF | 1.2316411 | 0.2915784 | 4.2240478 | 2.40E-05 | 0.000805 |
| IGKC | 2.9037158 | 0.6877231 | 4.2222166 | 2.42E-05 | 0.0008105 |
| RUNDC3B | 2.1509028 | 0.5096965 | 4.2199676 | 2.44E-05 | 0.0008176 |
| TTR | 3.889741 | 0.9219436 | 4.2190661 | 2.45E-05 | 0.0008198 |
| AP003721.4 | 2.4715828 | 0.5860281 | 4.2175161 | 2.47E-05 | 0.0008206 |
| TRPV4 | 1.603181 | 0.380133 | 4.2174208 | 2.47E-05 | 0.0008206 |
| IGHJ6 | 2.3303747 | 0.5524805 | 4.2180216 | 2.46E-05 | 0.0008206 |
| AC020663.2 | 4.3609281 | 1.0340121 | 4.2174827 | 2.47E-05 | 0.0008206 |
| AC009093.8 | 1.9465016 | 0.461539 | 4.2174151 | 2.47E-05 | 0.0008206 |
| HAGHL | 1.5658847 | 0.3713421 | 4.2168254 | 2.48E-05 | 0.0008217 |
| DCST2 | 1.7217775 | 0.4085614 | 4.214244 | 2.51E-05 | 0.000829 |
| AC104966.1 | 4.8539598 | 1.1527312 | 4.2108339 | 2.54E-05 | 0.0008406 |
| RAB3B | 2.0591103 | 0.4893706 | 4.2076708 | 2.58E-05 | 0.0008494 |
| SLC22A10 | -1.495442 | 0.3554132 | -4.207615 | 2.58E-05 | 0.0008494 |
| HSPB8 | -1.438345 | 0.3419708 | -4.206046 | 2.60E-05 | 0.0008542 |
| HNF1A | 2.974627 | 0.7075225 | 4.2042863 | 2.62E-05 | 0.0008598 |
| AP001085.1 | 2.2938714 | 0.545727 | 4.2033312 | 2.63E-05 | 0.0008624 |
| ACADL | -1.208799 | 0.2876203 | -4.20276 | 2.64E-05 | 0.0008635 |
| FAM149A | 1.0735289 | 0.2555042 | 4.20161 | 2.65E-05 | 0.0008668 |
| ASTN2 | 1.298938 | 0.3091837 | 4.2011859 | 2.66E-05 | 0.0008672 |
| GYG2 | 1.583566 | 0.3770828 | 4.1995179 | 2.67E-05 | 0.0008715 |
| THBS4 | 1.8714396 | 0.4458638 | 4.1973346 | 2.70E-05 | 0.000878 |
| PCP4 | 3.1740484 | 0.7562142 | 4.1972878 | 2.70E-05 | 0.000878 |
| DCST1 | 1.7305712 | 0.4125076 | 4.1952468 | 2.73E-05 | 0.0008845 |
| MAK | 1.6143352 | 0.3849826 | 4.1932674 | 2.75E-05 | 0.0008903 |
| AC233728.1 | 2.0244693 | 0.4829681 | 4.1917246 | 2.77E-05 | 0.0008942 |
| MMP11 | 1.4865842 | 0.3547205 | 4.1908602 | 2.78E-05 | 0.0008954 |
| OSCP1 | 1.5672997 | 0.3740643 | 4.1899208 | 2.79E-05 | 0.000898 |
| IGKV1-33 | 2.3502415 | 0.5610301 | 4.189154 | 2.80E-05 | 0.0008988 |
| LINC00886 | 1.335136 | 0.3187085 | 4.1892079 | 2.80E-05 | 0.0008988 |
| DZANK1 | 1.5413163 | 0.3681148 | 4.1870528 | 2.83E-05 | 0.0009061 |
| AC112204.1 | 2.6270736 | 0.6282038 | 4.1818812 | 2.89E-05 | 0.0009258 |
| FRMPD2 | 2.0190843 | 0.4829127 | 4.1810544 | 2.90E-05 | 0.000928 |
| AC008870.5 | 4.565687 | 1.0926419 | 4.1785756 | 2.93E-05 | 0.000937 |
| IFT81 | 1.1221608 | 0.2686169 | 4.1775509 | 2.95E-05 | 0.0009401 |
| EGLN3 | 1.1757529 | 0.2815996 | 4.1752653 | 2.98E-05 | 0.0009473 |
| FAM161A | 1.2640144 | 0.3027686 | 4.1748524 | 2.98E-05 | 0.0009478 |
| RCAN3AS | 2.223641 | 0.5326814 | 4.1744295 | 2.99E-05 | 0.0009485 |
| ZSCAN4 | 2.3311109 | 0.5584753 | 4.1740623 | 2.99E-05 | 0.0009488 |
| HMGB1P1 | 1.8256416 | 0.4375685 | 4.1722415 | 3.02E-05 | 0.0009553 |
| TMEM67 | 1.3591648 | 0.3258784 | 4.1707723 | 3.04E-05 | 0.0009603 |
| AP001207.3 | 2.3813524 | 0.5711444 | 4.1694404 | 3.05E-05 | 0.0009648 |
| GIHCG | 1.3979987 | 0.3353244 | 4.1690938 | 3.06E-05 | 0.0009651 |
| STARD4 | -1.16896 | 0.2804722 | -4.16783 | 3.08E-05 | 0.0009692 |
| CAPN13 | 1.9941516 | 0.4785181 | 4.1673481 | 3.08E-05 | 0.0009697 |
| SLC4A8 | 1.4757208 | 0.3541302 | 4.1671695 | 3.08E-05 | 0.0009697 |
| CYP2T1P | 1.0875445 | 0.2611674 | 4.1641671 | 3.12E-05 | 0.0009802 |
| MYO7B | -1.487925 | 0.3575685 | -4.161232 | 3.17E-05 | 0.0009917 |
| BTBD17 | 5.9364198 | 1.4274104 | 4.1588739 | 3.20E-05 | 0.0010008 |
| SMKR1 | 1.9807483 | 0.4767841 | 4.1543927 | 3.26E-05 | 0.0010194 |
| IGHV3-11 | 3.051007 | 0.7347907 | 4.1522122 | 3.29E-05 | 0.0010277 |
| IGLV2-11 | 2.8095007 | 0.6766658 | 4.1519768 | 3.30E-05 | 0.0010277 |
| FEZF1-AS1 | 5.9104047 | 1.4236629 | 4.1515478 | 3.30E-05 | 0.0010284 |
| C6orf141 | 1.1465249 | 0.2763371 | 4.1490081 | 3.34E-05 | 0.0010374 |
| HNF4A | 2.0241143 | 0.4878322 | 4.1492022 | 3.34E-05 | 0.0010374 |
| AXDND1 | 2.1977673 | 0.530021 | 4.1465664 | 3.37E-05 | 0.0010449 |
| AC112229.4 | 6.3673955 | 1.5354721 | 4.1468649 | 3.37E-05 | 0.0010449 |
| C15orf65 | 1.6491814 | 0.3977236 | 4.1465518 | 3.38E-05 | 0.0010449 |
| AC144831.1 | -1.174826 | 0.2833878 | -4.145649 | 3.39E-05 | 0.0010478 |
| TCTEX1D2 | 1.4222088 | 0.343147 | 4.1446047 | 3.40E-05 | 0.0010501 |
| NRAV | 1.4970862 | 0.3612149 | 4.1445855 | 3.40E-05 | 0.0010501 |
| LANCL1-AS1 | -1.471067 | 0.3550685 | -4.143052 | 3.43E-05 | 0.0010551 |
| IGHV4-34 | 2.1825641 | 0.5268112 | 4.1429718 | 3.43E-05 | 0.0010551 |
| C17orf97 | 1.6499511 | 0.3983337 | 4.142133 | 3.44E-05 | 0.0010577 |
| CYP3A5 | -1.456942 | 0.3519894 | -4.139165 | 3.49E-05 | 0.0010702 |
| TTC41P | 1.5201673 | 0.3675046 | 4.1364581 | 3.53E-05 | 0.0010816 |
| NEK2 | 1.3766336 | 0.3332273 | 4.1312144 | 3.61E-05 | 0.0010993 |
| EML6 | 1.573005 | 0.3807453 | 4.1313835 | 3.61E-05 | 0.0010993 |
| IL7 | 1.4919284 | 0.3611179 | 4.1314158 | 3.61E-05 | 0.0010993 |
| C9orf43 | 1.5051594 | 0.3643463 | 4.1311234 | 3.61E-05 | 0.0010993 |
| CPEB1-AS1 | 2.2692919 | 0.5491995 | 4.131999 | 3.60E-05 | 0.0010993 |
| SHISA6 | 1.584908 | 0.3836437 | 4.1311976 | 3.61E-05 | 0.0010993 |
| HOXC4 | 1.6211973 | 0.392586 | 4.1295342 | 3.63E-05 | 0.0011056 |
| RFX3 | 1.2480534 | 0.3023639 | 4.1276534 | 3.66E-05 | 0.0011134 |
| GJB7 | 2.0635314 | 0.5000211 | 4.1268888 | 3.68E-05 | 0.0011158 |
| TNFRSF12A | -1.592663 | 0.3860313 | -4.125734 | 3.70E-05 | 0.0011201 |
| FBXW10 | 2.3064614 | 0.5591619 | 4.1248544 | 3.71E-05 | 0.0011218 |
| TOX3 | 1.8767518 | 0.4550491 | 4.1242839 | 3.72E-05 | 0.0011233 |
| LPAR3 | 1.473523 | 0.3573394 | 4.1235956 | 3.73E-05 | 0.0011244 |
| AZIN1-AS1 | 1.3269086 | 0.3217975 | 4.123428 | 3.73E-05 | 0.0011244 |
| AL606469.1 | -1.531376 | 0.3714008 | -4.123243 | 3.74E-05 | 0.0011244 |
| GPR158 | -2.231291 | 0.5416266 | -4.119611 | 3.80E-05 | 0.001141 |
| TMEM190 | 2.4520382 | 0.5958242 | 4.1153717 | 3.87E-05 | 0.0011608 |
| ROPN1B | 2.1643465 | 0.5260205 | 4.1145668 | 3.88E-05 | 0.0011635 |
| NPHP1 | 1.3710031 | 0.3334024 | 4.1121573 | 3.92E-05 | 0.0011731 |
| AL451166.1 | 5.9132369 | 1.4380854 | 4.1118817 | 3.92E-05 | 0.0011731 |
| DPY19L2 | 1.085693 | 0.2640326 | 4.1119657 | 3.92E-05 | 0.0011731 |
| CCDC34 | 1.1824311 | 0.2875941 | 4.1114577 | 3.93E-05 | 0.0011732 |
| EPPIN-WFDC6 | 2.9320862 | 0.7132933 | 4.1106317 | 3.95E-05 | 0.0011741 |
| RHPN2 | 1.430473 | 0.3480901 | 4.1094902 | 3.97E-05 | 0.0011786 |
| AC092058.1 | 2.2487638 | 0.5472936 | 4.1088801 | 3.98E-05 | 0.0011791 |
| DNAJA4 | 1.5793153 | 0.3843678 | 4.1088648 | 3.98E-05 | 0.0011791 |
| IGKV1-16 | 2.6613093 | 0.6478751 | 4.1077506 | 4.00E-05 | 0.0011834 |
| DNPH1 | 1.0012207 | 0.2438322 | 4.106188 | 4.02E-05 | 0.0011889 |
| EFHC1 | 1.2989431 | 0.3163407 | 4.1061523 | 4.02E-05 | 0.0011889 |
| DPCD | 1.4167942 | 0.3450769 | 4.1057348 | 4.03E-05 | 0.0011891 |
| IGKV2-30 | 1.9936279 | 0.4860469 | 4.1017191 | 4.10E-05 | 0.0012065 |
| ANKMY1 | 1.2051021 | 0.2939086 | 4.1002614 | 4.13E-05 | 0.0012127 |
| MROH2A | -7.132745 | 1.739781 | -4.099795 | 4.14E-05 | 0.0012128 |
| COL7A1 | 1.3035394 | 0.3180933 | 4.0979779 | 4.17E-05 | 0.0012197 |
| CYP11A1 | 2.0571927 | 0.5022108 | 4.0962736 | 4.20E-05 | 0.0012255 |
| Z95115.1 | 1.3476842 | 0.3289865 | 4.0964725 | 4.19E-05 | 0.0012255 |
| RASSF10 | 1.6616354 | 0.4058744 | 4.0939644 | 4.24E-05 | 0.001235 |
| AC005100.1 | 3.5957012 | 0.8782474 | 4.0941781 | 4.24E-05 | 0.001235 |
| TTC30B | 1.502612 | 0.367061 | 4.0936302 | 4.25E-05 | 0.0012354 |
| SOX21-AS1 | 2.6184863 | 0.6397303 | 4.0931095 | 4.26E-05 | 0.0012368 |
| AC099521.4 | 2.1928696 | 0.5359202 | 4.0917841 | 4.28E-05 | 0.0012425 |
| AGR3 | 1.5654452 | 0.3826459 | 4.0911065 | 4.29E-05 | 0.0012448 |
| LINC00882 | 1.4529204 | 0.355165 | 4.0908323 | 4.30E-05 | 0.0012449 |
| GMNN | 1.0618492 | 0.259614 | 4.0901069 | 4.31E-05 | 0.0012474 |
| AMPD1 | 6.6405048 | 1.6247495 | 4.0870945 | 4.37E-05 | 0.0012595 |
| IGLJ3 | 2.2456068 | 0.5494241 | 4.0872012 | 4.37E-05 | 0.0012595 |
| IL17D | -1.260828 | 0.3085861 | -4.085822 | 4.39E-05 | 0.001265 |
| S100A2 | 2.0746644 | 0.5080113 | 4.0838937 | 4.43E-05 | 0.0012742 |
| RUVBL1 | 1.1773159 | 0.2884569 | 4.0814271 | 4.48E-05 | 0.0012853 |
| KNDC1 | 1.6477533 | 0.4037273 | 4.0813518 | 4.48E-05 | 0.0012853 |
| SNCAIP | 1.1238032 | 0.2754881 | 4.0793163 | 4.52E-05 | 0.0012952 |
| FAAH2 | 1.1696505 | 0.2867455 | 4.0790543 | 4.52E-05 | 0.0012953 |
| TSPAN32 | -1.102216 | 0.2703152 | -4.077523 | 4.55E-05 | 0.0012981 |
| DYNC2H1 | 1.2512549 | 0.3068495 | 4.0777481 | 4.55E-05 | 0.0012981 |
| IGHA2 | 2.946501 | 0.7226073 | 4.0775967 | 4.55E-05 | 0.0012981 |
| FPR2 | -1.834855 | 0.4499838 | -4.077602 | 4.55E-05 | 0.0012981 |
| EPB41L4B | 1.210684 | 0.2970679 | 4.0754457 | 4.59E-05 | 0.0013083 |
| AGBL4 | 1.870851 | 0.4591399 | 4.0746859 | 4.61E-05 | 0.0013112 |
| IGKV3D-15 | 3.2679538 | 0.8022185 | 4.0736457 | 4.63E-05 | 0.0013156 |
| TJP3 | 1.3558377 | 0.3329253 | 4.0724989 | 4.65E-05 | 0.0013207 |
| STRBP | 1.1056821 | 0.2717336 | 4.0689927 | 4.72E-05 | 0.0013392 |
| IGKJ3 | 2.5325166 | 0.6226075 | 4.0675971 | 4.75E-05 | 0.0013458 |
| IGKJ1 | 2.3565813 | 0.5798924 | 4.0638251 | 4.83E-05 | 0.0013663 |
| AL157700.1 | -1.331689 | 0.3277936 | -4.062583 | 4.85E-05 | 0.0013706 |
| SLC24A4 | -1.078539 | 0.265589 | -4.060933 | 4.89E-05 | 0.0013788 |
| PPP1R32 | 1.7662031 | 0.4350005 | 4.0602324 | 4.90E-05 | 0.0013795 |
| BAIAP3 | 1.5715432 | 0.3870714 | 4.0600857 | 4.91E-05 | 0.0013795 |
| AC108097.1 | 2.6007191 | 0.6405621 | 4.0600576 | 4.91E-05 | 0.0013795 |
| PPP1R36 | 1.7903685 | 0.4412726 | 4.0572848 | 4.96E-05 | 0.0013945 |
| CCDC30 | 1.3926901 | 0.3434467 | 4.0550403 | 5.01E-05 | 0.0014049 |
| AC010255.3 | 3.2780387 | 0.8086232 | 4.0538517 | 5.04E-05 | 0.0014091 |
| IGLV2-18 | 3.7081521 | 0.9153037 | 4.0512805 | 5.09E-05 | 0.0014231 |
| PSPH | 1.2356435 | 0.3053303 | 4.0469082 | 5.19E-05 | 0.0014468 |
| RAMP3 | -1.679801 | 0.4152619 | -4.045161 | 5.23E-05 | 0.0014561 |
| IQCA1 | 1.3635046 | 0.3370959 | 4.0448566 | 5.24E-05 | 0.0014564 |
| MAT1A | 2.0159278 | 0.4986022 | 4.0431585 | 5.27E-05 | 0.0014639 |
| FCN3 | -2.797531 | 0.692035 | -4.04247 | 5.29E-05 | 0.0014651 |
| NEXMIF | 1.2042553 | 0.2981949 | 4.0384838 | 5.38E-05 | 0.0014855 |
| B4GALNT4 | 1.224319 | 0.3034679 | 4.0344267 | 5.47E-05 | 0.0015082 |
| AC087392.2 | -1.910026 | 0.4735669 | -4.033276 | 5.50E-05 | 0.001514 |
| IRF1 | -1.209303 | 0.2999174 | -4.032119 | 5.53E-05 | 0.0015199 |
| ADAM28 | 1.5987911 | 0.3965606 | 4.031644 | 5.54E-05 | 0.0015213 |
| STEAP3 | 1.3407841 | 0.3326378 | 4.0307631 | 5.56E-05 | 0.0015238 |
| C2orf73 | 2.0865343 | 0.5181914 | 4.026571 | 5.66E-05 | 0.0015449 |
| LRRC4 | 1.572097 | 0.3904251 | 4.0266292 | 5.66E-05 | 0.0015449 |
| ALOX15 | 2.4006153 | 0.5961975 | 4.0265437 | 5.66E-05 | 0.0015449 |
| AWAT2 | -2.834155 | 0.7040947 | -4.025247 | 5.69E-05 | 0.0015518 |
| FBXW9 | 1.4438121 | 0.3587297 | 4.0247908 | 5.70E-05 | 0.0015532 |
| FAM86EP | 1.0525731 | 0.2615739 | 4.0239983 | 5.72E-05 | 0.0015552 |
| AC009119.1 | 2.3935158 | 0.5948048 | 4.024036 | 5.72E-05 | 0.0015552 |
| SORD | 1.4419738 | 0.3588655 | 4.018146 | 5.87E-05 | 0.0015927 |
| HMGA1P8 | 1.9645946 | 0.4891089 | 4.0166809 | 5.90E-05 | 0.0015993 |
| CLDN1 | 1.5426579 | 0.3840608 | 4.0167028 | 5.90E-05 | 0.0015993 |
| TMPRSS7 | 3.2771772 | 0.8162362 | 4.0149863 | 5.94E-05 | 0.0016091 |
| GLIS3 | 1.3200891 | 0.3289691 | 4.012806 | 6.00E-05 | 0.0016196 |
| RGMA | 1.0722445 | 0.2672114 | 4.0127204 | 6.00E-05 | 0.0016196 |
| GPR156 | 2.1798502 | 0.543757 | 4.0088687 | 6.10E-05 | 0.0016429 |
| ETV5 | -1.181015 | 0.2946447 | -4.00827 | 6.12E-05 | 0.0016453 |
| LHFPL3-AS2 | -1.452996 | 0.3625281 | -4.007954 | 6.12E-05 | 0.0016458 |
| SPATA4 | 2.1350025 | 0.5328804 | 4.0065324 | 6.16E-05 | 0.0016503 |
| IGLV2-23 | 2.6942439 | 0.6724937 | 4.0063481 | 6.17E-05 | 0.0016503 |
| KCNN3 | 1.1505077 | 0.2874554 | 4.0023862 | 6.27E-05 | 0.001673 |
| BIN2 | -1.007809 | 0.2518022 | -4.002383 | 6.27E-05 | 0.001673 |
| AL137139.2 | -1.724909 | 0.4309333 | -4.002728 | 6.26E-05 | 0.001673 |
| GPC5-AS1 | 2.53293 | 0.6331527 | 4.0005043 | 6.32E-05 | 0.0016788 |
| CCDC40 | 1.6914532 | 0.4227694 | 4.000888 | 6.31E-05 | 0.0016788 |
| HS6ST2 | 1.8957291 | 0.473849 | 4.0007033 | 6.32E-05 | 0.0016788 |
| AC010273.3 | 1.2865739 | 0.3216145 | 4.0003602 | 6.32E-05 | 0.0016788 |
| HNF1A-AS1 | 2.4857123 | 0.6220164 | 3.9962164 | 6.44E-05 | 0.001705 |
| ANXA13 | 2.7390047 | 0.6855265 | 3.9954758 | 6.46E-05 | 0.0017068 |
| UNC13D | -1.109308 | 0.2776273 | -3.995674 | 6.45E-05 | 0.0017068 |
| IGHV3-43 | 2.804185 | 0.7019843 | 3.9946549 | 6.48E-05 | 0.001711 |
| FSD1L | 1.1822905 | 0.2960464 | 3.9935985 | 6.51E-05 | 0.0017169 |
| CCDC141 | -1.432672 | 0.3588014 | -3.99294 | 6.53E-05 | 0.00172 |
| COL9A2 | 1.0260972 | 0.2570931 | 3.9911507 | 6.58E-05 | 0.0017295 |
| MUC13 | 4.8844626 | 1.2251139 | 3.9869458 | 6.69E-05 | 0.0017548 |
| AL035701.1 | 2.1342493 | 0.535412 | 3.986181 | 6.71E-05 | 0.0017572 |
| SPEF2 | 1.0956421 | 0.2748844 | 3.9858283 | 6.72E-05 | 0.0017581 |
| AC108681.1 | 3.7625944 | 0.9443243 | 3.9844303 | 6.76E-05 | 0.0017649 |
| IGLC3 | 2.7847928 | 0.699491 | 3.9811703 | 6.86E-05 | 0.0017857 |
| ZNF19 | 1.016244 | 0.2558107 | 3.9726402 | 7.11E-05 | 0.0018454 |
| LRRN4 | -1.652917 | 0.4161139 | -3.97227 | 7.12E-05 | 0.0018464 |
| IGHV3-74 | 2.1577299 | 0.5432478 | 3.9719076 | 7.13E-05 | 0.0018469 |
| SMIM34A | 1.9867779 | 0.500229 | 3.9717367 | 7.14E-05 | 0.0018469 |
| AL157871.2 | -1.727667 | 0.4353447 | -3.968503 | 7.23E-05 | 0.0018684 |
| CHRDL2 | 1.923732 | 0.484999 | 3.9664659 | 7.29E-05 | 0.0018826 |
| TTC34 | 1.4249439 | 0.3593228 | 3.9656368 | 7.32E-05 | 0.0018873 |
| ANGPTL6 | -1.415311 | 0.3570183 | -3.964252 | 7.36E-05 | 0.0018964 |
| IGKV1D-39 | 2.7409435 | 0.6918296 | 3.9618769 | 7.44E-05 | 0.0019116 |
| SPATA24 | 1.3107561 | 0.3314045 | 3.9551545 | 7.65E-05 | 0.0019642 |
| AL121820.3 | 1.0286917 | 0.2601356 | 3.954444 | 7.67E-05 | 0.0019681 |
| C2orf81 | 1.5469776 | 0.3912695 | 3.9537392 | 7.69E-05 | 0.001972 |
| FPR1 | -1.833122 | 0.4637213 | -3.953068 | 7.72E-05 | 0.0019756 |
| HNRNPA1P50 | 2.1816369 | 0.5522178 | 3.9506819 | 7.79E-05 | 0.0019934 |
| SPATA33 | 1.1894005 | 0.3011217 | 3.9499001 | 7.82E-05 | 0.001998 |
| CT45A2 | -3.839569 | 0.9727752 | -3.947026 | 7.91E-05 | 0.0020201 |
| CT45A1 | -4.24059 | 1.0744562 | -3.946731 | 7.92E-05 | 0.0020206 |
| FENDRR | -1.620803 | 0.4107798 | -3.945673 | 7.96E-05 | 0.0020276 |
| CT45A8 | -4.702213 | 1.1919657 | -3.944923 | 7.98E-05 | 0.002032 |
| INAVA | 1.0506879 | 0.2663915 | 3.9441499 | 8.01E-05 | 0.0020365 |
| SAP30-DT | 1.0725229 | 0.2721618 | 3.9407545 | 8.12E-05 | 0.0020596 |
| ECRG4 | 1.3879488 | 0.3524661 | 3.9378216 | 8.22E-05 | 0.0020788 |
| IL12A | 1.3776243 | 0.3498315 | 3.9379654 | 8.22E-05 | 0.0020788 |
| GPD1 | -1.611664 | 0.4093059 | -3.937553 | 8.23E-05 | 0.0020791 |
| NDRG4 | -1.56446 | 0.3973792 | -3.936944 | 8.25E-05 | 0.0020824 |
| IGHV1-3 | 2.5059198 | 0.6368983 | 3.9345682 | 8.33E-05 | 0.002099 |
| TMEM231P1 | 1.8724654 | 0.4761149 | 3.9328018 | 8.40E-05 | 0.0021084 |
| SIX2 | 3.2855493 | 0.8354848 | 3.9325063 | 8.41E-05 | 0.002109 |
| PNMA8C | 2.1263681 | 0.5408273 | 3.9316953 | 8.43E-05 | 0.0021141 |
| TMEM45B | 1.2396391 | 0.3155772 | 3.928165 | 8.56E-05 | 0.0021411 |
| AC026704.1 | 2.8226845 | 0.7191806 | 3.9248616 | 8.68E-05 | 0.0021646 |
| CEP19 | 1.2394418 | 0.315894 | 3.9236001 | 8.72E-05 | 0.0021739 |
| KRT80 | 1.4578994 | 0.371698 | 3.9222682 | 8.77E-05 | 0.0021818 |
| SMR3A | 6.6099457 | 1.6858128 | 3.9209251 | 8.82E-05 | 0.0021919 |
| CHGA | 2.0909481 | 0.5337325 | 3.9175953 | 8.94E-05 | 0.0022202 |
| AC010538.1 | 1.3779052 | 0.3517666 | 3.9171007 | 8.96E-05 | 0.0022227 |
| ATP2B3 | 2.6215605 | 0.6694131 | 3.9162075 | 9.00E-05 | 0.0022288 |
| DCST1-AS1 | 2.0167219 | 0.5155015 | 3.9121553 | 9.15E-05 | 0.0022623 |
| CFAP206 | 2.7122092 | 0.6935703 | 3.9105038 | 9.21E-05 | 0.0022756 |
| KRT4 | 1.6916398 | 0.4331935 | 3.9050438 | 9.42E-05 | 0.0023254 |
| PDE1A | 1.1144478 | 0.2854863 | 3.9036821 | 9.47E-05 | 0.0023363 |
| GOLGA2P5 | 1.3632143 | 0.3492411 | 3.9033612 | 9.49E-05 | 0.0023372 |
| HSPD1P21 | 2.1690572 | 0.5561488 | 3.900138 | 9.61E-05 | 0.0023641 |
| AC093159.1 | 2.6611677 | 0.6825104 | 3.8990874 | 9.66E-05 | 0.0023718 |
| CATSPER1 | -1.184442 | 0.303789 | -3.898896 | 9.66E-05 | 0.0023718 |
| IGLV1-47 | 2.3178653 | 0.5945331 | 3.8986309 | 9.67E-05 | 0.0023721 |
| NAP1L3 | 1.1568284 | 0.2967684 | 3.8980848 | 9.70E-05 | 0.0023753 |
| AKAP3 | 1.2405034 | 0.318522 | 3.8945607 | 9.84E-05 | 0.0024033 |
| Z92544.1 | 1.2505082 | 0.3210827 | 3.8946607 | 9.83E-05 | 0.0024033 |
| CEP83 | 1.049816 | 0.2696106 | 3.8938227 | 9.87E-05 | 0.0024083 |
| SOX9-AS1 | 1.844248 | 0.4739384 | 3.8913245 | 9.97E-05 | 0.002431 |
| AL390879.1 | 1.3952499 | 0.3586993 | 3.8897484 | 0.0001003 | 0.0024423 |
| AC079610.2 | 4.2754639 | 1.0994062 | 3.8888846 | 0.0001007 | 0.0024464 |
| SYT5 | 2.1216031 | 0.5457162 | 3.8877406 | 0.0001012 | 0.0024557 |
| CNIH2 | 1.5385375 | 0.3959182 | 3.8859982 | 0.0001019 | 0.0024665 |
| GNLY | -1.510144 | 0.3886982 | -3.885133 | 0.0001023 | 0.0024707 |
| ERBB4 | 1.6231674 | 0.4177675 | 3.8853368 | 0.0001022 | 0.0024707 |
| IGHV6-1 | 2.062256 | 0.5310494 | 3.8833604 | 0.000103 | 0.0024842 |
| DPEP2 | -1.219331 | 0.3140784 | -3.882249 | 0.0001035 | 0.0024924 |
| PCAT14 | -2.944174 | 0.7591403 | -3.8783 | 0.0001052 | 0.0025271 |
| TMPRSS3 | 1.4423779 | 0.3720322 | 3.8770238 | 0.0001057 | 0.0025357 |
| IGKJ2 | 2.3992394 | 0.6188991 | 3.8766244 | 0.0001059 | 0.0025375 |
| C6orf52 | 1.7166933 | 0.4430192 | 3.8749861 | 0.0001066 | 0.0025476 |
| SLC15A1 | 2.4142679 | 0.6230308 | 3.875038 | 0.0001066 | 0.0025476 |
| FP671120.6 | 3.1076648 | 0.802068 | 3.8745653 | 0.0001068 | 0.0025497 |
| LRP4 | -1.10886 | 0.2863741 | -3.872067 | 0.0001079 | 0.0025713 |
| UBASH3B | -1.060325 | 0.2742253 | -3.866619 | 0.0001104 | 0.0026245 |
| IGHV1-69 | 3.0530615 | 0.7896868 | 3.8661675 | 0.0001106 | 0.002627 |
| CFAP161 | 1.516372 | 0.3922807 | 3.8655281 | 0.0001108 | 0.0026315 |
| MAPK8IP1 | 1.113512 | 0.2881664 | 3.8641284 | 0.0001115 | 0.0026442 |
| IQCC | 1.1172601 | 0.2893757 | 3.8609324 | 0.000113 | 0.0026742 |
| PCAT6 | 1.2243762 | 0.3172207 | 3.8596987 | 0.0001135 | 0.0026829 |
| TRAF3IP1 | 1.0501376 | 0.2720774 | 3.8597024 | 0.0001135 | 0.0026829 |
| ODF3L1 | -1.642338 | 0.4259488 | -3.855716 | 0.0001154 | 0.0027195 |
| AK9 | 1.2514975 | 0.3246504 | 3.8549085 | 0.0001158 | 0.0027236 |
| IGLV1-44 | 2.2618683 | 0.5871045 | 3.8525821 | 0.0001169 | 0.0027471 |
| AC079848.2 | 1.7208079 | 0.4467466 | 3.8518658 | 0.0001172 | 0.0027527 |
| AC008915.3 | 1.7899796 | 0.4652816 | 3.8470885 | 0.0001195 | 0.0027993 |
| AL449403.3 | 2.3205682 | 0.6032427 | 3.8468233 | 0.0001197 | 0.0027999 |
| PRF1 | -1.587229 | 0.4127661 | -3.845346 | 0.0001204 | 0.0028142 |
| AL513477.1 | 1.1030083 | 0.2868972 | 3.8446122 | 0.0001207 | 0.0028176 |
| PART1 | 2.0099074 | 0.5228155 | 3.8443913 | 0.0001209 | 0.0028176 |
| WDR86 | 1.2222208 | 0.3180956 | 3.8423069 | 0.0001219 | 0.0028392 |
| CRYM | 1.2813422 | 0.3336186 | 3.8407398 | 0.0001227 | 0.0028548 |
| KLHDC9 | 1.6953454 | 0.4414635 | 3.8402844 | 0.0001229 | 0.002855 |
| AL137786.1 | 1.9640558 | 0.5114265 | 3.8403478 | 0.0001229 | 0.002855 |
| TTC21A | 1.3405073 | 0.3491106 | 3.839778 | 0.0001231 | 0.0028583 |
| FBXO16 | 1.5396275 | 0.4009914 | 3.8395529 | 0.0001233 | 0.0028584 |
| SLC11A1 | -1.280564 | 0.3337766 | -3.83659 | 0.0001248 | 0.0028905 |
| WNT4 | 1.2495075 | 0.3257061 | 3.8363033 | 0.0001249 | 0.0028913 |
| PKIB | 1.5620278 | 0.4072516 | 3.8355348 | 0.0001253 | 0.0028978 |
| C4orf19 | 1.0707352 | 0.2792339 | 3.8345463 | 0.0001258 | 0.0028997 |
| HECW1 | 1.7482915 | 0.4559298 | 3.8345631 | 0.0001258 | 0.0028997 |
| AC083862.1 | -1.238209 | 0.3229457 | -3.83411 | 0.000126 | 0.0029018 |
| NLRP12 | -2.06281 | 0.5380484 | -3.833874 | 0.0001261 | 0.002902 |
| NFE2 | -1.362332 | 0.355373 | -3.833527 | 0.0001263 | 0.0029036 |
| PZP | 1.5296124 | 0.3990981 | 3.8326726 | 0.0001268 | 0.0029085 |
| LINC02532 | 3.2939545 | 0.8596716 | 3.8316428 | 0.0001273 | 0.0029182 |
| ACBD3-AS1 | 2.8838151 | 0.752755 | 3.8310141 | 0.0001276 | 0.0029231 |
| USP43 | 1.5339047 | 0.400493 | 3.8300412 | 0.0001281 | 0.0029321 |
| TMPRSS4 | 3.5603419 | 0.929737 | 3.8294075 | 0.0001285 | 0.002937 |
| AC109583.1 | 1.1930662 | 0.3116391 | 3.8283591 | 0.000129 | 0.0029445 |
| AC097532.3 | 2.1827054 | 0.5701418 | 3.828355 | 0.000129 | 0.0029445 |
| GCNT3 | 3.2285472 | 0.8434495 | 3.8277893 | 0.0001293 | 0.0029487 |
| C8orf34-AS1 | 1.2342701 | 0.3224813 | 3.8274164 | 0.0001295 | 0.0029505 |
| CFC1B | 1.9004218 | 0.4965841 | 3.826989 | 0.0001297 | 0.0029531 |
| AC112204.3 | 2.3370265 | 0.6107245 | 3.826646 | 0.0001299 | 0.0029546 |
| CYP4F32P | 3.353996 | 0.8766324 | 3.8260004 | 0.0001302 | 0.0029598 |
| DAAM2-AS1 | -1.441365 | 0.3768385 | -3.824887 | 0.0001308 | 0.0029706 |
| CCN4 | 1.4652778 | 0.3832287 | 3.8235077 | 0.0001316 | 0.0029847 |
| NUP62CL | 1.656575 | 0.433436 | 3.8219603 | 0.0001324 | 0.0030009 |
| MORN1 | 1.367388 | 0.3577993 | 3.8216625 | 0.0001326 | 0.0030019 |
| CCDC191 | 1.0797252 | 0.2826011 | 3.8206692 | 0.0001331 | 0.0030114 |
| TTN | -1.186707 | 0.3109381 | -3.816537 | 0.0001353 | 0.0030589 |
| FAM189A1 | -1.687998 | 0.4423036 | -3.81638 | 0.0001354 | 0.0030589 |
| IGKJ5 | 2.204475 | 0.5779134 | 3.814542 | 0.0001364 | 0.0030764 |
| RABL2A | 1.222296 | 0.3205576 | 3.8130312 | 0.0001373 | 0.0030926 |
| IGHV3-13 | 2.8931303 | 0.7589746 | 3.8118934 | 0.0001379 | 0.0030989 |
| AC137630.1 | 1.8906487 | 0.4961909 | 3.8103253 | 0.0001388 | 0.0031159 |
| JCHAIN | 2.5781617 | 0.6767692 | 3.8095141 | 0.0001392 | 0.0031234 |
| IFT46 | 1.1058309 | 0.2903231 | 3.808966 | 0.0001395 | 0.0031277 |
| AL391840.1 | 2.2453398 | 0.5897004 | 3.8075939 | 0.0001403 | 0.0031424 |
| NOS1 | -1.71263 | 0.449896 | -3.806724 | 0.0001408 | 0.003148 |
| IGHV3-21 | 2.4082896 | 0.6326072 | 3.8069272 | 0.0001407 | 0.003148 |
| CDS1 | 1.0254946 | 0.2697046 | 3.8022877 | 0.0001434 | 0.0031995 |
| TBX21 | -1.441304 | 0.3798567 | -3.794337 | 0.000148 | 0.003298 |
| PSENEN | 1.2258664 | 0.3230954 | 3.7941315 | 0.0001482 | 0.003298 |
| EHMT2-AS1 | 1.1914508 | 0.3140872 | 3.7933753 | 0.0001486 | 0.0033046 |
| RMST | -1.895385 | 0.4996782 | -3.793212 | 0.0001487 | 0.0033046 |
| AL137847.2 | 1.9697588 | 0.5196539 | 3.7905207 | 0.0001503 | 0.0033348 |
| NQO1 | 1.1014108 | 0.2905852 | 3.7903195 | 0.0001505 | 0.0033348 |
| AC099050.1 | 2.2061346 | 0.5821891 | 3.7893778 | 0.000151 | 0.0033446 |
| CRACR2B | 1.1022984 | 0.290969 | 3.7883705 | 0.0001516 | 0.0033554 |
| MNDA | -1.247605 | 0.3295692 | -3.785564 | 0.0001534 | 0.0033877 |
| AC022107.1 | 1.5486852 | 0.4094177 | 3.782653 | 0.0001552 | 0.0034189 |
| TFF3 | 1.2456048 | 0.3295485 | 3.7797311 | 0.000157 | 0.0034534 |
| SPOCK3 | 3.3241325 | 0.8796642 | 3.7788654 | 0.0001575 | 0.0034556 |
| SOWAHA | 2.5776884 | 0.6820813 | 3.7791513 | 0.0001574 | 0.0034556 |
| FABP6-AS1 | 2.1271101 | 0.5629601 | 3.7784384 | 0.0001578 | 0.0034556 |
| C12orf75 | 1.6267345 | 0.4305688 | 3.7781062 | 0.000158 | 0.0034556 |
| TMEM107 | 1.0885756 | 0.2881238 | 3.7781521 | 0.000158 | 0.0034556 |
| CA2 | -1.497097 | 0.3965642 | -3.77517 | 0.0001599 | 0.0034907 |
| STK32B | 1.2020228 | 0.3186106 | 3.7727018 | 0.0001615 | 0.0035195 |
| STEAP2 | 1.3841102 | 0.3670444 | 3.7709611 | 0.0001626 | 0.0035412 |
| SMAD6 | -1.615645 | 0.428697 | -3.768735 | 0.0001641 | 0.00357 |
| BX005214.1 | 2.4486619 | 0.6499352 | 3.7675475 | 0.0001649 | 0.003581 |
| ZSCAN1 | 1.5838711 | 0.4205038 | 3.7666037 | 0.0001655 | 0.0035886 |
| ANKRD65 | 1.1620639 | 0.3086188 | 3.7653695 | 0.0001663 | 0.0036034 |
| HSD11B1L | 1.0930182 | 0.2905048 | 3.7624788 | 0.0001682 | 0.0036423 |
| MUC20 | 1.1913329 | 0.317197 | 3.7558136 | 0.0001728 | 0.0037344 |
| AL161772.1 | -1.079162 | 0.2874133 | -3.754737 | 0.0001735 | 0.0037474 |
| SRGAP3 | 1.4237627 | 0.3792578 | 3.7540766 | 0.000174 | 0.0037541 |
| IVL | 2.5029025 | 0.6668529 | 3.7533055 | 0.0001745 | 0.0037626 |
| AL161618.1 | 2.2887794 | 0.6098674 | 3.752913 | 0.0001748 | 0.0037654 |
| VENTX | -1.212963 | 0.3234609 | -3.749954 | 0.0001769 | 0.0038038 |
| ADRB1 | -1.328719 | 0.3544019 | -3.749188 | 0.0001774 | 0.0038123 |
| IGHV5-51 | 2.7780884 | 0.7410476 | 3.7488664 | 0.0001776 | 0.003814 |
| NME7 | 1.0834623 | 0.2890667 | 3.7481393 | 0.0001782 | 0.0038188 |
| REEP2 | 1.4662921 | 0.3911862 | 3.7483225 | 0.000178 | 0.0038188 |
| LINC00939 | 2.0803551 | 0.5554317 | 3.7454741 | 0.0001801 | 0.0038533 |
| IGKV1-17 | 3.6751533 | 0.9821203 | 3.7420601 | 0.0001825 | 0.0039017 |
| B3GNT3 | 3.9088096 | 1.0453967 | 3.7390682 | 0.0001847 | 0.0039366 |
| PLA2G4F | -1.603542 | 0.4289023 | -3.738712 | 0.000185 | 0.003939 |
| TTC30A | 1.1514339 | 0.3080111 | 3.7382873 | 0.0001853 | 0.0039424 |
| IL22RA1 | 1.0987812 | 0.2939628 | 3.7378235 | 0.0001856 | 0.0039437 |
| PGC | -1.53916 | 0.4117827 | -3.737797 | 0.0001856 | 0.0039437 |
| MYO16 | 1.0671233 | 0.2855291 | 3.7373536 | 0.000186 | 0.0039474 |
| AL807752.7 | 8.1023129 | 2.1682759 | 3.7367537 | 0.0001864 | 0.0039536 |
| AVPR1A | 2.1433067 | 0.5740912 | 3.7333905 | 0.0001889 | 0.0040036 |
| IL7R | -1.695777 | 0.4543019 | -3.732708 | 0.0001894 | 0.0040112 |
| ROPN1 | 2.6939087 | 0.7221206 | 3.7305521 | 0.0001911 | 0.0040325 |
| KLRD1 | -1.510011 | 0.4048392 | -3.729905 | 0.0001916 | 0.0040396 |
| AL096711.2 | -1.836429 | 0.4924657 | -3.72905 | 0.0001922 | 0.0040468 |
| IFT172 | 1.0629565 | 0.2850684 | 3.7287772 | 0.0001924 | 0.0040479 |
| LINC00643 | 2.2620657 | 0.6067893 | 3.7279263 | 0.0001931 | 0.0040583 |
| SELPLG | -1.099218 | 0.2949334 | -3.727005 | 0.0001938 | 0.0040699 |
| AC092802.2 | 1.6417188 | 0.4405483 | 3.7265354 | 0.0001941 | 0.0040742 |
| CNGA3 | 1.9958227 | 0.5356024 | 3.7263142 | 0.0001943 | 0.0040745 |
| AC074212.1 | 1.2245051 | 0.3287906 | 3.7242701 | 0.0001959 | 0.0041044 |
| WNT7A | -1.544261 | 0.4147031 | -3.723776 | 0.0001963 | 0.0041058 |
| ANKDD1B | 1.2703405 | 0.3411246 | 3.7239781 | 0.0001961 | 0.0041058 |
| AL359979.2 | 6.2407067 | 1.6763347 | 3.7228285 | 0.000197 | 0.004118 |
| FAM47E | 1.3083438 | 0.3514998 | 3.7221756 | 0.0001975 | 0.0041241 |
| MOK | 1.2867483 | 0.3457097 | 3.7220488 | 0.0001976 | 0.0041241 |
| GRIN1 | 1.374012 | 0.3692414 | 3.721175 | 0.0001983 | 0.0041351 |
| VWA7 | 1.5082882 | 0.4053748 | 3.720725 | 0.0001987 | 0.0041391 |
| C2orf15 | 1.1706646 | 0.3147038 | 3.7198938 | 0.0001993 | 0.0041495 |
| TENM3 | 1.7194285 | 0.4622856 | 3.7194074 | 0.0001997 | 0.0041541 |
| SNX18P3 | 1.9690033 | 0.5294362 | 3.7190569 | 0.0002 | 0.0041566 |
| AC023510.1 | 2.7522804 | 0.7407562 | 3.7155011 | 0.0002028 | 0.0042054 |
| SYNM | -1.090535 | 0.2934839 | -3.715827 | 0.0002025 | 0.0042054 |
| ANKRD42 | 1.0776077 | 0.2900495 | 3.7152545 | 0.000203 | 0.0042062 |
| AC012409.1 | 3.9229496 | 1.0561555 | 3.7143676 | 0.0002037 | 0.0042156 |
| CNTN6 | -1.012936 | 0.2727732 | -3.713473 | 0.0002044 | 0.0042258 |
| SCGB3A2 | 2.1095322 | 0.5681394 | 3.7130536 | 0.0002048 | 0.0042295 |
| ALDH3A1 | 1.5505884 | 0.4176852 | 3.7123377 | 0.0002054 | 0.0042324 |
| SERTAD1 | -1.47051 | 0.3961206 | -3.712279 | 0.0002054 | 0.0042324 |
| IGLV1-51 | 3.7238685 | 1.0032559 | 3.7117835 | 0.0002058 | 0.0042371 |
| ALDH3B2 | 2.3736762 | 0.6398322 | 3.7098421 | 0.0002074 | 0.0042632 |
| IGKV2-24 | 2.8877758 | 0.7786821 | 3.7085429 | 0.0002085 | 0.0042818 |
| GBP6 | 1.8951948 | 0.511069 | 3.7082958 | 0.0002087 | 0.0042826 |
| CFAP298 | 1.0118206 | 0.2729104 | 3.7075192 | 0.0002093 | 0.004289 |
| TMEM45A | 1.1827538 | 0.3190624 | 3.7069664 | 0.0002098 | 0.004295 |
| MEIS3P2 | 1.607727 | 0.433784 | 3.706285 | 0.0002103 | 0.0043032 |
| NDUFA5P8 | 3.5603849 | 0.9607343 | 3.7058995 | 0.0002106 | 0.0043063 |
| PRIMA1 | -1.004644 | 0.271193 | -3.704534 | 0.0002118 | 0.0043228 |
| AC011473.3 | 1.90297 | 0.5136866 | 3.7045348 | 0.0002118 | 0.0043228 |
| VWA3A | 1.4672588 | 0.3962138 | 3.7031992 | 0.0002129 | 0.0043388 |
| DUSP8 | -1.581623 | 0.4274792 | -3.699883 | 0.0002157 | 0.004372 |
| LINC00551 | -2.349233 | 0.6349331 | -3.699969 | 0.0002156 | 0.004372 |
| FSTL3 | -1.167188 | 0.3154395 | -3.700197 | 0.0002154 | 0.004372 |
| AC016590.3 | 1.7114775 | 0.4624684 | 3.700745 | 0.000215 | 0.004372 |
| AC010255.2 | 3.4020093 | 0.9198385 | 3.6984855 | 0.0002169 | 0.0043927 |
| OCA2 | 1.4162831 | 0.383301 | 3.6949632 | 0.0002199 | 0.0044472 |
| DAPL1 | 4.7082499 | 1.2745583 | 3.6940249 | 0.0002207 | 0.0044602 |
| TCTN1 | 1.1172455 | 0.302536 | 3.6929345 | 0.0002217 | 0.0044737 |
| CENPM | 1.3668024 | 0.3701203 | 3.6928603 | 0.0002217 | 0.0044737 |
| ALX1 | 3.9785173 | 1.077501 | 3.6923561 | 0.0002222 | 0.0044791 |
| IGHA1 | 2.8815494 | 0.7805702 | 3.6915955 | 0.0002229 | 0.0044891 |
| LINC02577 | -1.783326 | 0.4832793 | -3.690053 | 0.0002242 | 0.0045094 |
| CC2D2A | 1.1243022 | 0.3047687 | 3.6890347 | 0.0002251 | 0.004524 |
| AC093928.1 | 1.8859909 | 0.5116559 | 3.6860532 | 0.0002278 | 0.0045703 |
| AL357153.1 | 3.2553664 | 0.8832895 | 3.6855031 | 0.0002283 | 0.0045766 |
| AL161908.1 | 5.2925524 | 1.4365968 | 3.6840903 | 0.0002295 | 0.004595 |
| DTHD1 | 1.8350887 | 0.4983872 | 3.6820545 | 0.0002314 | 0.0046283 |
| ATRNL1 | -1.262266 | 0.3428445 | -3.681745 | 0.0002316 | 0.0046304 |
| SLC2A3 | -1.418422 | 0.3853302 | -3.681056 | 0.0002323 | 0.0046393 |
| LINC02623 | 6.0669089 | 1.6486357 | 3.6799573 | 0.0002333 | 0.0046558 |
| AL713852.1 | 1.4215438 | 0.3863336 | 3.6795757 | 0.0002336 | 0.0046572 |
| HK3 | -1.081737 | 0.2939906 | -3.679494 | 0.0002337 | 0.0046572 |
| ILDR2 | -1.193272 | 0.3246401 | -3.675675 | 0.0002372 | 0.0047175 |
| TPPP3 | 1.4204381 | 0.3864481 | 3.675625 | 0.0002373 | 0.0047175 |
| CLDN10 | 3.4403099 | 0.9363921 | 3.6740058 | 0.0002388 | 0.0047403 |
| IL1A | -2.452695 | 0.66787 | -3.672415 | 0.0002403 | 0.0047663 |
| DBH | -1.190475 | 0.3242847 | -3.67108 | 0.0002415 | 0.0047805 |
| MLF1 | 1.2397047 | 0.337766 | 3.6703062 | 0.0002423 | 0.0047912 |
| SEC14L5 | 1.462331 | 0.3984726 | 3.6698405 | 0.0002427 | 0.0047963 |
| AC011899.1 | 2.7018502 | 0.7366076 | 3.6679641 | 0.0002445 | 0.0048243 |
| CSMD2 | 1.9148871 | 0.5222563 | 3.6665661 | 0.0002458 | 0.004847 |
| IL13RA2 | 2.3688622 | 0.6461753 | 3.6659748 | 0.0002464 | 0.0048546 |
| NOXRED1 | 1.39852 | 0.3816686 | 3.6642261 | 0.0002481 | 0.0048805 |
| EDN1 | -1.551007 | 0.4236654 | -3.660924 | 0.0002513 | 0.004929 |
| PPP1R26-AS1 | 1.024994 | 0.2800266 | 3.6603451 | 0.0002519 | 0.0049364 |
| ALOX5AP | -1.182207 | 0.3230009 | -3.660074 | 0.0002521 | 0.0049379 |
| AC131649.2 | -1.633339 | 0.4463328 | -3.659464 | 0.0002527 | 0.004946 |
| AC006230.1 | 1.2842143 | 0.3510273 | 3.6584451 | 0.0002538 | 0.0049582 |
| CT45A7 | -4.242159 | 1.1595154 | -3.658562 | 0.0002536 | 0.0049582 |
| H2BU1 | 1.8154243 | 0.4962805 | 3.6580605 | 0.0002541 | 0.0049619 |
| VIPR1-AS1 | -1.697863 | 0.4642954 | -3.65686 | 0.0002553 | 0.0049778 |
| ATP12A | 3.7481831 | 1.0249705 | 3.6568692 | 0.0002553 | 0.0049778 |
| VAT1L | -1.252824 | 0.3426229 | -3.656569 | 0.0002556 | 0.0049797 |
| BPIFA1 | 7.8488969 | 2.1469286 | 3.6558723 | 0.0002563 | 0.0049895 |
| PDLIM4 | 1.251432 | 0.342791 | 3.650714 | 0.0002615 | 0.005087 |
| SALL4 | 1.4479223 | 0.3969935 | 3.647219 | 0.0002651 | 0.005149 |
| DUSP6 | -1.17452 | 0.3221631 | -3.645732 | 0.0002666 | 0.005175 |
| LINC01783 | 1.9045007 | 0.5224434 | 3.6453724 | 0.000267 | 0.0051784 |
| CCL15 | 4.5879264 | 1.259168 | 3.6436174 | 0.0002688 | 0.0052093 |
| RHBDL2 | 1.4925335 | 0.409698 | 3.6430088 | 0.0002695 | 0.0052145 |
| STEAP1 | 1.6808999 | 0.4615419 | 3.6419223 | 0.0002706 | 0.0052288 |
| EFNB3 | 1.5516638 | 0.4261787 | 3.6408762 | 0.0002717 | 0.0052424 |
| AC254633.1 | 1.3789122 | 0.3791168 | 3.6371695 | 0.0002757 | 0.0053144 |
| COL4A4 | -1.127488 | 0.3103555 | -3.632892 | 0.0002803 | 0.0053892 |
| EFCAB11 | 1.0481553 | 0.2885195 | 3.6328753 | 0.0002803 | 0.0053892 |
| ATP10B | 5.7913203 | 1.5940853 | 3.6330053 | 0.0002801 | 0.0053892 |
| SERPINE1 | -1.806385 | 0.4974581 | -3.63123 | 0.0002821 | 0.0054182 |
| AL354696.1 | 1.346349 | 0.3708052 | 3.6308798 | 0.0002825 | 0.0054216 |
| AC233263.6 | -2.718984 | 0.7495036 | -3.627712 | 0.0002859 | 0.0054804 |
| AC025279.1 | 1.3650464 | 0.3762647 | 3.627889 | 0.0002857 | 0.0054804 |
| IGHV3-64 | 3.8552892 | 1.0641456 | 3.6228963 | 0.0002913 | 0.0055672 |
| LPL | -1.18254 | 0.3266243 | -3.620491 | 0.000294 | 0.0056151 |
| IGLV3-25 | 2.4744952 | 0.6835112 | 3.62027 | 0.0002943 | 0.0056158 |
| B3GALT5 | 2.347325 | 0.6485486 | 3.6193513 | 0.0002953 | 0.0056275 |
| GLB1L | 1.3507627 | 0.3732506 | 3.6189165 | 0.0002958 | 0.0056314 |
| ARMC2 | 1.1880263 | 0.3283236 | 3.6184615 | 0.0002964 | 0.0056314 |
| ABCD2 | 1.6784226 | 0.46388 | 3.6182261 | 0.0002966 | 0.0056314 |
| CEACAM3 | -1.692339 | 0.4677255 | -3.618232 | 0.0002966 | 0.0056314 |
| AFF2 | -1.353561 | 0.3740436 | -3.618726 | 0.0002961 | 0.0056314 |
| IFTAP | 1.0822366 | 0.2991348 | 3.6178892 | 0.000297 | 0.0056346 |
| AC005264.1 | -1.959656 | 0.5421174 | -3.614818 | 0.0003006 | 0.0056812 |
| AC004066.1 | 3.1472248 | 0.8705823 | 3.6150803 | 0.0003003 | 0.0056812 |
| H4C15 | 1.0030263 | 0.2774983 | 3.6145318 | 0.0003009 | 0.0056834 |
| AL590004.3 | 2.8506586 | 0.7887169 | 3.6142991 | 0.0003012 | 0.0056843 |
| BHLHA15 | 1.4770277 | 0.4087038 | 3.6139324 | 0.0003016 | 0.0056883 |
| KLK10 | 1.1025122 | 0.3050991 | 3.6136198 | 0.000302 | 0.005691 |
| AL135960.1 | -1.416261 | 0.3920951 | -3.612034 | 0.0003038 | 0.0057177 |
| AC092718.3 | 1.4381862 | 0.3982352 | 3.6113985 | 0.0003046 | 0.0057276 |
| UCN3 | 3.5356255 | 0.9809962 | 3.6041174 | 0.0003132 | 0.0058736 |
| FAM86JP | 1.5999132 | 0.4441328 | 3.6023308 | 0.0003154 | 0.0059099 |
| SLC38A4 | 1.9606228 | 0.5443114 | 3.6020242 | 0.0003157 | 0.0059126 |
| RABL2B | 1.1711541 | 0.3251906 | 3.6014389 | 0.0003165 | 0.0059217 |
| TP53AIP1 | 2.2808403 | 0.6334628 | 3.6005905 | 0.0003175 | 0.0059368 |
| KLF10 | -1.061449 | 0.295062 | -3.597375 | 0.0003214 | 0.006002 |
| MIR6510 | 2.2600487 | 0.6285135 | 3.5958636 | 0.0003233 | 0.006024 |
| AL121768.1 | 2.2236526 | 0.6184264 | 3.5956624 | 0.0003236 | 0.0060244 |
| TUBB8P7 | 5.9657218 | 1.6600862 | 3.5936216 | 0.0003261 | 0.0060674 |
| LINC01513 | 1.9352981 | 0.5391134 | 3.5897791 | 0.000331 | 0.0061522 |
| ADGRF2 | 2.5959733 | 0.7237277 | 3.5869474 | 0.0003346 | 0.0062027 |
| AL139220.2 | -1.35093 | 0.3767102 | -3.586126 | 0.0003356 | 0.0062178 |
| GREM1 | 2.297617 | 0.6408142 | 3.5854652 | 0.0003365 | 0.0062291 |
| LRRC37A5P | 2.6760585 | 0.7465805 | 3.5844205 | 0.0003378 | 0.0062453 |
| IGKV2D-30 | 2.654217 | 0.7405989 | 3.583879 | 0.0003385 | 0.0062538 |
| GREB1L | -1.729036 | 0.4824787 | -3.583653 | 0.0003388 | 0.0062548 |
| PCSK4 | 1.3538882 | 0.3779367 | 3.5823148 | 0.0003406 | 0.0062825 |
| STC2 | -1.664811 | 0.4648098 | -3.581704 | 0.0003414 | 0.0062927 |
| GRIK2 | 1.8052746 | 0.5040847 | 3.5812919 | 0.0003419 | 0.0062982 |
| DISP1 | -1.085754 | 0.30328 | -3.58004 | 0.0003435 | 0.006318 |
| ZNF396 | 1.0660633 | 0.2977898 | 3.579919 | 0.0003437 | 0.006318 |
| AGAP12P | 1.3838757 | 0.3868885 | 3.5769372 | 0.0003476 | 0.0063824 |
| STYK1 | 1.7641203 | 0.4931981 | 3.5769002 | 0.0003477 | 0.0063824 |
| GHRL | 1.6028466 | 0.4482273 | 3.5759684 | 0.0003489 | 0.0064006 |
| ZNF750 | 1.040329 | 0.2909705 | 3.5753761 | 0.0003497 | 0.0064106 |
| ENPP3 | 1.7907621 | 0.5012016 | 3.5729381 | 0.000353 | 0.0064616 |
| BMP2 | -1.301662 | 0.3644334 | -3.571742 | 0.0003546 | 0.006482 |
| EPAS1 | -1.171007 | 0.3278818 | -3.57143 | 0.000355 | 0.0064852 |
| ANKRD18A | 1.3930095 | 0.3901969 | 3.5700168 | 0.000357 | 0.0065157 |
| EFCAB2 | 1.2956669 | 0.3629932 | 3.5693969 | 0.0003578 | 0.006522 |
| SH2D1B | -1.773428 | 0.4969733 | -3.568457 | 0.0003591 | 0.0065409 |
| ANKRD45 | 2.7732482 | 0.7774357 | 3.5671737 | 0.0003609 | 0.0065684 |
| AC020763.1 | 2.6416469 | 0.7409268 | 3.5653279 | 0.0003634 | 0.0066102 |
| PPM1E | 1.8546221 | 0.5204191 | 3.5637086 | 0.0003657 | 0.0066459 |
| AC103809.1 | 2.341464 | 0.6570626 | 3.5635328 | 0.0003659 | 0.0066459 |
| HOXB1 | 3.484065 | 0.9779448 | 3.5626397 | 0.0003671 | 0.0066597 |
| GPR162 | 1.1968622 | 0.3361975 | 3.5599968 | 0.0003709 | 0.0066993 |
| LINC01146 | -1.338675 | 0.3759796 | -3.560498 | 0.0003702 | 0.0066993 |
| PADI3 | 4.6959046 | 1.3205293 | 3.5560774 | 0.0003764 | 0.006781 |
| AL031663.1 | 2.6900191 | 0.7570798 | 3.5531515 | 0.0003806 | 0.0068441 |
| CLCNKA | 1.7029198 | 0.4794609 | 3.5517384 | 0.0003827 | 0.0068709 |
| AC008752.3 | 2.8649872 | 0.8066513 | 3.5517048 | 0.0003827 | 0.0068709 |
| EBF3 | 1.527322 | 0.4301128 | 3.5509804 | 0.0003838 | 0.0068804 |
| AC124067.3 | 3.358518 | 0.9461013 | 3.5498503 | 0.0003855 | 0.006884 |
| MPPED2 | 1.2032367 | 0.338963 | 3.5497586 | 0.0003856 | 0.006884 |
| LINC01170 | 6.0207195 | 1.6962007 | 3.5495324 | 0.0003859 | 0.0068852 |
| AC068389.1 | 3.1031336 | 0.8747524 | 3.5474421 | 0.000389 | 0.0069353 |
| AL136985.3 | 1.666193 | 0.4700303 | 3.5448631 | 0.0003928 | 0.0069702 |
| RGS13 | 1.7458833 | 0.4924924 | 3.5449956 | 0.0003926 | 0.0069702 |
| SYT8 | 1.3702852 | 0.3865237 | 3.5451523 | 0.0003924 | 0.0069702 |
| SV2B | 1.2324494 | 0.3479358 | 3.5421746 | 0.0003968 | 0.007032 |
| SLC6A4 | -2.726704 | 0.7698357 | -3.54193 | 0.0003972 | 0.0070338 |
| LRRC32 | -1.436135 | 0.4055728 | -3.541004 | 0.0003986 | 0.0070515 |
| LINC00589 | 2.6906589 | 0.7600188 | 3.540253 | 0.0003997 | 0.0070642 |
| FAM221B | 1.4920066 | 0.4215584 | 3.5392644 | 0.0004012 | 0.0070763 |
| WFDC6 | 2.3235937 | 0.656739 | 3.538078 | 0.0004031 | 0.0071034 |
| PLEKHB1 | 1.2068878 | 0.3413893 | 3.5352243 | 0.0004074 | 0.007166 |
| BMPER | -1.206305 | 0.3414226 | -3.533172 | 0.0004106 | 0.0072121 |
| CATSPERE | 1.2385058 | 0.3505964 | 3.5325684 | 0.0004115 | 0.0072154 |
| MYRF | -1.013374 | 0.2868512 | -3.532753 | 0.0004113 | 0.0072154 |
| IGHG2 | 2.726878 | 0.7719355 | 3.5325205 | 0.0004116 | 0.0072154 |
| GLDN | -1.599742 | 0.4529046 | -3.532183 | 0.0004121 | 0.0072197 |
| SDS | 2.471338 | 0.6997497 | 3.5317455 | 0.0004128 | 0.0072268 |
| CCDC190 | 2.2583428 | 0.6402555 | 3.5272522 | 0.0004199 | 0.0073309 |
| PTPRN | 2.1775177 | 0.6175474 | 3.5260741 | 0.0004218 | 0.0073411 |
| AL606760.1 | 1.3686081 | 0.3881972 | 3.5255483 | 0.0004226 | 0.0073438 |
| COL11A1 | 2.3572546 | 0.6685911 | 3.5257043 | 0.0004224 | 0.0073438 |
| ZNHIT2 | 1.0798744 | 0.3064828 | 3.5234423 | 0.000426 | 0.0073925 |
| EHF | 1.3805066 | 0.3918665 | 3.5229003 | 0.0004269 | 0.0073995 |
| SIAH3 | 2.0666723 | 0.5866497 | 3.5228387 | 0.000427 | 0.0073995 |
| NPM2 | 1.1009257 | 0.3125287 | 3.522639 | 0.0004273 | 0.0074002 |
| TRGC1 | -1.023096 | 0.2906252 | -3.520328 | 0.000431 | 0.0074551 |
| FTO | 1.0879333 | 0.3092764 | 3.5176729 | 0.0004353 | 0.0075002 |
| RUVBL2 | 1.0277139 | 0.2922177 | 3.5169463 | 0.0004365 | 0.0075147 |
| AL445489.1 | 3.1820216 | 0.905023 | 3.5159565 | 0.0004382 | 0.0075253 |
| ZC2HC1C | 1.252735 | 0.3564239 | 3.5147337 | 0.0004402 | 0.0075537 |
| DNAJC22 | 1.3498161 | 0.3841563 | 3.5137162 | 0.0004419 | 0.0075728 |
| AL136982.4 | -1.784994 | 0.5080968 | -3.513099 | 0.0004429 | 0.0075804 |
| NAT1 | 1.2798603 | 0.3643704 | 3.512525 | 0.0004439 | 0.0075918 |
| TAL1 | -1.227733 | 0.349864 | -3.509171 | 0.0004495 | 0.0076781 |
| ADH7 | 4.6298321 | 1.3195854 | 3.5085505 | 0.0004506 | 0.007691 |
| HIPK1-AS1 | 1.1920797 | 0.3399244 | 3.5068965 | 0.0004534 | 0.0077238 |
| MMP10 | 5.1038416 | 1.4558556 | 3.5057334 | 0.0004554 | 0.0077475 |
| LRWD1 | 1.1478042 | 0.3274556 | 3.5052208 | 0.0004562 | 0.0077523 |
| AC125611.4 | 1.6236743 | 0.4631994 | 3.5053466 | 0.000456 | 0.0077523 |
| ADGRF4 | 2.3804087 | 0.6793196 | 3.5041071 | 0.0004581 | 0.0077747 |
| COL12A1 | -1.001428 | 0.2858487 | -3.503351 | 0.0004594 | 0.0077917 |
| IGLV3-9 | 1.9611512 | 0.5604148 | 3.4994638 | 0.0004662 | 0.0078857 |
| AC104809.1 | 1.8698224 | 0.5344012 | 3.4989116 | 0.0004672 | 0.0078922 |
| PLA1A | -1.272236 | 0.3636105 | -3.498899 | 0.0004672 | 0.0078922 |
| AC244090.1 | 1.0773897 | 0.3080263 | 3.4977196 | 0.0004693 | 0.007922 |
| LINC00861 | -1.030372 | 0.2946351 | -3.497111 | 0.0004703 | 0.0079321 |
| AC011511.5 | -1.863318 | 0.5328282 | -3.497033 | 0.0004705 | 0.0079321 |
| CFAP44 | 1.1139862 | 0.3186158 | 3.4963307 | 0.0004717 | 0.0079479 |
| LNCTAM34A | 1.2104201 | 0.3463266 | 3.4950251 | 0.000474 | 0.0079817 |
| NOL4 | 2.4397448 | 0.6981467 | 3.4946017 | 0.0004748 | 0.0079892 |
| PRRT3 | 1.051573 | 0.3009624 | 3.4940346 | 0.0004758 | 0.0079986 |
| ZNF440 | 1.0870009 | 0.3111405 | 3.4936017 | 0.0004766 | 0.0079986 |
| CATIP | 1.4433792 | 0.4134704 | 3.4908889 | 0.0004814 | 0.0080491 |
| CAPN5 | 1.1342857 | 0.3249268 | 3.4908964 | 0.0004814 | 0.0080491 |
| DPEP3 | -5.017297 | 1.4371654 | -3.491106 | 0.000481 | 0.0080491 |
| AP002852.1 | 1.8162704 | 0.5202872 | 3.4908999 | 0.0004814 | 0.0080491 |
| MEFV | -1.331588 | 0.3815132 | -3.49028 | 0.0004825 | 0.0080623 |
| NME9 | 1.5251037 | 0.4370344 | 3.4896652 | 0.0004836 | 0.0080757 |
| AL359813.1 | -1.849762 | 0.5302064 | -3.488759 | 0.0004853 | 0.0080928 |
| MATR3 | 7.4670594 | 2.1406385 | 3.4882393 | 0.0004862 | 0.0081033 |
| SUGCT | 1.1695436 | 0.3353595 | 3.4874323 | 0.0004877 | 0.0081175 |
| SMC2-AS1 | 1.8023458 | 0.5168866 | 3.4869266 | 0.0004886 | 0.0081224 |
| RAB26 | 1.5501783 | 0.4446683 | 3.4861455 | 0.00049 | 0.008141 |
| CYCSP10 | 1.3835895 | 0.3969554 | 3.4855038 | 0.0004912 | 0.008145 |
| C11orf21 | -1.204964 | 0.3459744 | -3.482813 | 0.0004962 | 0.0081998 |
| AC012178.1 | 2.4016264 | 0.6899143 | 3.4810503 | 0.0004995 | 0.0082397 |
| AC011503.1 | 4.3896631 | 1.26237 | 3.4773189 | 0.0005065 | 0.0083236 |
| SLC4A11 | 1.9078368 | 0.5485655 | 3.4778651 | 0.0005054 | 0.0083236 |
| CLBA1 | 1.2168924 | 0.3502134 | 3.4747172 | 0.0005114 | 0.0083889 |
| TMC5 | 1.2409433 | 0.3571942 | 3.4741417 | 0.0005125 | 0.0084016 |
| LNC-LBCS | 1.3935327 | 0.4011819 | 3.473568 | 0.0005136 | 0.0084047 |
| TSPEAR-AS1 | -1.297638 | 0.3735784 | -3.473537 | 0.0005136 | 0.0084047 |
| TDO2 | 1.5645137 | 0.4508425 | 3.4702005 | 0.0005201 | 0.0084832 |
| IGHV4-59 | 1.5191622 | 0.4379084 | 3.4691328 | 0.0005221 | 0.0085117 |
| AL161910.1 | 1.7447976 | 0.5030582 | 3.468381 | 0.0005236 | 0.0085302 |
| ADRA2A | 2.0305859 | 0.5855752 | 3.4676776 | 0.000525 | 0.0085443 |
| AC007695.1 | 1.5261584 | 0.4401988 | 3.4669756 | 0.0005264 | 0.0085535 |
| XG | 1.7123676 | 0.4938962 | 3.4670598 | 0.0005262 | 0.0085535 |
| LINC00365 | 1.3369892 | 0.3856857 | 3.4665256 | 0.0005272 | 0.0085625 |
| SYN2 | -1.95592 | 0.5645455 | -3.464593 | 0.000531 | 0.0086028 |
| CERNA2 | 3.7381995 | 1.0792308 | 3.4637628 | 0.0005327 | 0.0086134 |
| SYT12 | 2.8009646 | 0.8088735 | 3.4627968 | 0.0005346 | 0.008639 |
| ZDHHC1 | 1.1514329 | 0.3325994 | 3.4619212 | 0.0005363 | 0.0086618 |
| AC093866.1 | 3.0920265 | 0.8932715 | 3.4614632 | 0.0005372 | 0.0086658 |
| MYO1G | -1.017336 | 0.2940053 | -3.460263 | 0.0005396 | 0.0086884 |
| MAP7D2 | 1.4075888 | 0.4069462 | 3.4589065 | 0.0005424 | 0.0087263 |
| PAG1 | -1.011608 | 0.292638 | -3.456856 | 0.0005465 | 0.0087719 |
| CT45A3 | -3.745502 | 1.0836039 | -3.456523 | 0.0005472 | 0.0087773 |
| TMEM100 | -1.908216 | 0.5521126 | -3.456209 | 0.0005478 | 0.0087821 |
| AC104035.1 | 2.8747902 | 0.8323104 | 3.4539883 | 0.0005524 | 0.0088493 |
| RPL21P54 | 2.3271909 | 0.673964 | 3.4529901 | 0.0005544 | 0.0088712 |
| AC124947.2 | 3.2951459 | 0.9545551 | 3.4520229 | 0.0005564 | 0.0088922 |
| ADH6 | 1.3170208 | 0.381714 | 3.4502816 | 0.00056 | 0.0089407 |
| CDKN2A | 2.2082587 | 0.640192 | 3.4493694 | 0.0005619 | 0.0089581 |
| AL391807.1 | -1.083848 | 0.3143544 | -3.447854 | 0.0005651 | 0.009003 |
| MMP19 | -1.551212 | 0.4500141 | -3.44703 | 0.0005668 | 0.009025 |
| WFDC1 | -1.026403 | 0.2978802 | -3.445692 | 0.0005696 | 0.0090642 |
| UBE3D | 1.2225852 | 0.3550833 | 3.4430938 | 0.0005751 | 0.0091462 |
| EDNRB | -1.484663 | 0.4312367 | -3.442804 | 0.0005757 | 0.0091504 |
| HYAL1 | -1.345264 | 0.3909476 | -3.441033 | 0.0005795 | 0.0092049 |
| SAA1 | 4.7867251 | 1.3915382 | 3.4398805 | 0.000582 | 0.0092349 |
| AC124067.4 | 2.023501 | 0.5884856 | 3.4384884 | 0.000585 | 0.0092661 |
| CST1 | 6.4104501 | 1.8643612 | 3.4384164 | 0.0005851 | 0.0092661 |
| ITGA11 | 1.097724 | 0.3193526 | 3.4373416 | 0.0005875 | 0.0092973 |
| AL138828.1 | -1.664689 | 0.4843524 | -3.436938 | 0.0005883 | 0.009302 |
| PWWP3B | 1.6792225 | 0.488652 | 3.4364386 | 0.0005894 | 0.0093113 |
| LINC00381 | 2.815875 | 0.8196458 | 3.4354777 | 0.0005915 | 0.0093388 |
| AC093904.4 | 2.2448352 | 0.6535649 | 3.4347548 | 0.0005931 | 0.009358 |
| ELF5 | 1.4436185 | 0.4203296 | 3.4344915 | 0.0005937 | 0.0093615 |
| S100A8 | -1.827576 | 0.5330722 | -3.428383 | 0.0006072 | 0.0095458 |
| G0S2 | -1.081937 | 0.3155564 | -3.428665 | 0.0006066 | 0.0095458 |
| SERPINF1 | 1.1461545 | 0.3342992 | 3.4285295 | 0.0006069 | 0.0095458 |
| AC138356.3 | 6.4494179 | 1.8822142 | 3.4265058 | 0.0006114 | 0.0095889 |
| C5AR2 | -1.018122 | 0.2973411 | -3.424088 | 0.0006169 | 0.0096688 |
| CD52 | -1.19223 | 0.3483475 | -3.422531 | 0.0006204 | 0.0097127 |
| C1GALT1C1L | 1.5599939 | 0.4560222 | 3.4208725 | 0.0006242 | 0.0097545 |
| PRMT8 | 1.6259673 | 0.4753051 | 3.4208917 | 0.0006242 | 0.0097545 |
| BNIP5 | 5.7233806 | 1.6731925 | 3.4206348 | 0.0006248 | 0.0097572 |
| SLCO4A1-AS1 | -2.222837 | 0.6498884 | -3.420337 | 0.0006254 | 0.009762 |
| GLIS1 | 2.2273686 | 0.6515319 | 3.4186639 | 0.0006293 | 0.0098012 |
| IGKV4-1 | 2.0069331 | 0.5870524 | 3.4186607 | 0.0006293 | 0.0098012 |
| FBXL13 | 1.2346612 | 0.361152 | 3.4186746 | 0.0006293 | 0.0098012 |
| AC012313.8 | 1.020787 | 0.2985982 | 3.4185977 | 0.0006294 | 0.0098012 |
| TTLL13P | 1.4063586 | 0.4114468 | 3.4180817 | 0.0006306 | 0.0098081 |
| LILRA2 | -1.1468 | 0.3356131 | -3.417031 | 0.0006331 | 0.0098401 |
| COL28A1 | 1.5001004 | 0.4390539 | 3.4166657 | 0.0006339 | 0.0098416 |
| PKNOX2 | -1.001116 | 0.2929984 | -3.416798 | 0.0006336 | 0.0098416 |
| LRTOMT | 1.2461493 | 0.3647939 | 3.416037 | 0.0006354 | 0.0098585 |
| SNORA7B | 1.5854277 | 0.4642381 | 3.4151177 | 0.0006375 | 0.0098859 |
| ADCY2 | 1.0946772 | 0.3206554 | 3.4138744 | 0.0006405 | 0.0099193 |
| LINC02795 | 1.3419992 | 0.3931396 | 3.4135439 | 0.0006412 | 0.0099218 |
| KIAA1257 | 1.0861592 | 0.3181971 | 3.4134792 | 0.0006414 | 0.0099218 |
| AC020612.4 | 3.2133246 | 0.9417794 | 3.4119717 | 0.0006449 | 0.0099572 |
| SLCO4A1 | -1.574941 | 0.461604 | -3.411887 | 0.0006451 | 0.0099572 |
| FSIP1 | 1.5486275 | 0.4539843 | 3.411192 | 0.0006468 | 0.0099716 |
| PRR36 | 1.2282772 | 0.3600772 | 3.4111497 | 0.0006469 | 0.0099716 |
| PCOLCE2 | -1.219992 | 0.3576994 | -3.410663 | 0.0006481 | 0.0099835 |
| DMRTA1 | 1.1329845 | 0.3322498 | 3.4100381 | 0.0006495 | 0.0099889 |
| BBOX1 | 3.9411777 | 1.1558142 | 3.4098714 | 0.0006499 | 0.0099889 |
| TMEM132E | 1.4443749 | 0.4235747 | 3.4099654 | 0.0006497 | 0.0099889 |
| MYLK3 | 1.5643596 | 0.4589045 | 3.4089001 | 0.0006523 | 0.0100112 |
| AC073896.3 | 1.072287 | 0.3146484 | 3.4078896 | 0.0006547 | 0.0100212 |
| LAMA3 | -1.106335 | 0.3246397 | -3.407887 | 0.0006547 | 0.0100212 |
| RNF183 | 1.9258704 | 0.5659598 | 3.4028395 | 0.0006669 | 0.0101538 |
| USH1C | 1.588012 | 0.4668115 | 3.4018274 | 0.0006694 | 0.0101855 |
| FUZ | 1.110938 | 0.3268851 | 3.398558 | 0.0006774 | 0.0102679 |
| PRR29-AS1 | 1.4367598 | 0.4228939 | 3.3974473 | 0.0006802 | 0.0102899 |
| SPRY4 | -1.242018 | 0.365703 | -3.396247 | 0.0006832 | 0.0103172 |
| CST7 | -1.14527 | 0.3372532 | -3.395876 | 0.0006841 | 0.0103252 |
| PGM2L1 | 1.0710088 | 0.3154347 | 3.3953418 | 0.0006854 | 0.0103394 |
| GOLGA2P10 | 1.1371562 | 0.3349582 | 3.3949199 | 0.0006865 | 0.0103459 |
| CHGB | 1.791862 | 0.5279008 | 3.3943157 | 0.000688 | 0.0103602 |
| HBEGF | -1.484546 | 0.4375172 | -3.393115 | 0.000691 | 0.0103877 |
| FAM86C2P | 1.1482437 | 0.3383957 | 3.393198 | 0.0006908 | 0.0103877 |
| CDK20 | 1.0103263 | 0.2978958 | 3.3915426 | 0.000695 | 0.0104415 |
| AL353770.4 | 2.1935678 | 0.6469008 | 3.3908876 | 0.0006967 | 0.0104448 |
| LINC02218 | -6.398682 | 1.8869145 | -3.391082 | 0.0006962 | 0.0104448 |
| ERVMER34-1 | -1.428935 | 0.4215092 | -3.390045 | 0.0006988 | 0.0104625 |
| IGLV5-45 | 3.6364066 | 1.0732805 | 3.3881233 | 0.0007037 | 0.01053 |
| AC004130.2 | 1.5125458 | 0.4465479 | 3.3871974 | 0.0007061 | 0.0105535 |
| EPHA7 | 1.4001122 | 0.4133817 | 3.3869714 | 0.0007067 | 0.0105562 |
| MUC16 | 4.2001988 | 1.2404765 | 3.3859558 | 0.0007093 | 0.0105892 |
| ESM1 | -1.647704 | 0.4867721 | -3.384961 | 0.0007119 | 0.0106216 |
| IL18R1 | -1.477076 | 0.4364322 | -3.384434 | 0.0007133 | 0.0106298 |
| MPP3 | -1.348656 | 0.3985079 | -3.384265 | 0.0007137 | 0.0106303 |
| AC110619.1 | 1.3686098 | 0.4044762 | 3.3836599 | 0.0007153 | 0.0106477 |
| PTBP1P | 2.4201992 | 0.7156173 | 3.3819742 | 0.0007197 | 0.0107071 |
| AC092171.3 | 1.0981039 | 0.324911 | 3.3797065 | 0.0007256 | 0.0107897 |
| ZNF295-AS1 | 2.6495987 | 0.7842012 | 3.3787231 | 0.0007282 | 0.010816 |
| LINC01602 | 3.5511878 | 1.0515236 | 3.3771831 | 0.0007323 | 0.0108291 |
| GEMIN7-AS1 | 1.0658515 | 0.3156078 | 3.3771398 | 0.0007324 | 0.0108291 |
| C10orf95 | 1.5522422 | 0.459667 | 3.3768839 | 0.0007331 | 0.0108331 |
| AP000553.7 | 2.3160635 | 0.686166 | 3.3753692 | 0.0007372 | 0.0108744 |
| COLCA2 | 1.0937269 | 0.3241248 | 3.3744005 | 0.0007398 | 0.0109065 |
| CU634019.4 | 2.4934099 | 0.7391618 | 3.3732936 | 0.0007427 | 0.0109319 |
| IFT27 | 1.0701789 | 0.3175058 | 3.3705801 | 0.0007501 | 0.0110117 |
| AL021396.1 | 2.486276 | 0.7384228 | 3.3670088 | 0.0007599 | 0.0111213 |
| COL4A3 | -1.356925 | 0.4031575 | -3.365744 | 0.0007634 | 0.0111599 |
| AC130456.2 | 1.5461528 | 0.4600399 | 3.3609098 | 0.0007769 | 0.0113317 |
| AL137781.1 | 2.4781617 | 0.737492 | 3.3602558 | 0.0007787 | 0.0113522 |
| LINC00299 | -1.115087 | 0.3319564 | -3.359136 | 0.0007819 | 0.0113836 |
| SYTL3 | 1.1236714 | 0.3345227 | 3.3590288 | 0.0007822 | 0.0113836 |
| KATNAL2 | 1.0882573 | 0.3241176 | 3.3575996 | 0.0007862 | 0.0114108 |
| AC128685.1 | 4.0315396 | 1.2006955 | 3.3576704 | 0.000786 | 0.0114108 |
| LINC02785 | -1.210343 | 0.3609471 | -3.353242 | 0.0007987 | 0.0115469 |
| PITPNM1 | 1.0788523 | 0.3219248 | 3.3512558 | 0.0008045 | 0.0116108 |
| LRRC56 | 1.4637073 | 0.4369231 | 3.3500337 | 0.000808 | 0.0116492 |
| SUSD5 | 1.3659299 | 0.4079172 | 3.3485468 | 0.0008124 | 0.011699 |
| LINC01977 | 1.3934849 | 0.4164892 | 3.3457887 | 0.0008205 | 0.0117834 |
| MYOZ1 | -1.383156 | 0.4135831 | -3.344324 | 0.0008248 | 0.0118198 |
| MB | 2.359074 | 0.7055259 | 3.3437098 | 0.0008267 | 0.01183 |
| LAMP3 | -1.039817 | 0.3111157 | -3.342219 | 0.0008311 | 0.0118836 |
| IGLV3-10 | 2.5990322 | 0.7781019 | 3.3402207 | 0.0008371 | 0.0119498 |
| RARRES1 | 1.2833624 | 0.3842481 | 3.339932 | 0.000838 | 0.0119557 |
| NBEA | 1.242607 | 0.3721884 | 3.3386502 | 0.0008419 | 0.0119978 |
| SEMA6A | -1.194991 | 0.358019 | -3.337787 | 0.0008445 | 0.0120286 |
| LINC00656 | -1.913092 | 0.5732065 | -3.337527 | 0.0008453 | 0.0120333 |
| ZNF273 | 1.3293178 | 0.3983455 | 3.3370978 | 0.0008466 | 0.012041 |
| TNNI3 | 1.5550008 | 0.4661653 | 3.3357287 | 0.0008508 | 0.0120916 |
| AC010442.1 | 1.1490914 | 0.3445891 | 3.3346713 | 0.000854 | 0.012131 |
| C6orf201 | 1.748269 | 0.5244518 | 3.3335171 | 0.0008576 | 0.0121748 |
| AC011405.1 | 2.3679986 | 0.7105322 | 3.3327111 | 0.00086 | 0.0122035 |
| MIR429 | 1.1776349 | 0.3534397 | 3.3319256 | 0.0008625 | 0.0122314 |
| CR392000.2 | 2.3040838 | 0.6916058 | 3.3314987 | 0.0008638 | 0.0122435 |
| LRRC26 | 1.9331312 | 0.5803754 | 3.3308288 | 0.0008659 | 0.0122663 |
| SUSD4 | 1.0618585 | 0.3189393 | 3.3293434 | 0.0008705 | 0.0123118 |
| AP003393.1 | 1.8388101 | 0.5524567 | 3.3284236 | 0.0008734 | 0.0123459 |
| NMBR | 2.9877129 | 0.8979734 | 3.3271731 | 0.0008773 | 0.0123947 |
| ADAM12 | 1.2491222 | 0.3755182 | 3.3263961 | 0.0008798 | 0.0124112 |
| FOXN4 | 2.4166972 | 0.7269143 | 3.3245973 | 0.0008855 | 0.0124827 |
| NPTX1 | 1.5079829 | 0.4536444 | 3.3241521 | 0.0008869 | 0.0124959 |
| PKDCC | -1.29053 | 0.3883718 | -3.322925 | 0.0008908 | 0.0125374 |
| ANKRD44 | -1.049538 | 0.3159126 | -3.322242 | 0.000893 | 0.0125546 |
| UBXN11 | 1.2148521 | 0.3656983 | 3.322006 | 0.0008937 | 0.0125568 |
| MUC12-AS1 | 1.5952622 | 0.480227 | 3.3218921 | 0.0008941 | 0.0125568 |
| PCDH15 | -1.05198 | 0.3167718 | -3.320939 | 0.0008972 | 0.012593 |
| AC005840.3 | -3.159533 | 0.9515645 | -3.320357 | 0.000899 | 0.0126125 |
| PRR15 | 1.7625989 | 0.5310474 | 3.3190986 | 0.0009031 | 0.0126537 |
| IGHV3-30 | 2.4231804 | 0.7300945 | 3.3189956 | 0.0009034 | 0.0126537 |
| LMOD3 | -1.048746 | 0.3160554 | -3.318235 | 0.0009059 | 0.0126814 |
| AL353622.1 | 1.2393951 | 0.3735315 | 3.3180471 | 0.0009065 | 0.0126831 |
| AL353608.3 | 2.9841925 | 0.8994272 | 3.317881 | 0.000907 | 0.0126838 |
| FAM107A | -1.57339 | 0.4743136 | -3.317194 | 0.0009093 | 0.0127083 |
| SLFN13 | 1.0494498 | 0.3164035 | 3.3168089 | 0.0009105 | 0.012719 |
| NIPAL1 | 1.0766624 | 0.3246793 | 3.3160795 | 0.0009129 | 0.0127454 |
| PLG | 1.3671893 | 0.4123585 | 3.3155353 | 0.0009147 | 0.0127634 |
| CCDC162P | 1.7375286 | 0.5241526 | 3.3149286 | 0.0009167 | 0.0127843 |
| IGKV3D-11 | 3.6397766 | 1.0981161 | 3.3145644 | 0.0009179 | 0.0127941 |
| AC044849.1 | 1.3471581 | 0.4065527 | 3.3136123 | 0.000921 | 0.012824 |
| C3orf86 | -1.501239 | 0.4533249 | -3.311618 | 0.0009276 | 0.0128982 |
| ARMC4P1 | 2.8722856 | 0.868131 | 3.3085853 | 0.0009377 | 0.0130286 |
| AC104984.2 | -2.636677 | 0.7971771 | -3.307517 | 0.0009413 | 0.0130615 |
| CCL28 | 1.1087533 | 0.3353355 | 3.3064002 | 0.000945 | 0.0130983 |
| KRT6B | 6.3815658 | 1.9300967 | 3.3063452 | 0.0009452 | 0.0130983 |
| GPM6A | -1.301714 | 0.3944087 | -3.30042 | 0.0009654 | 0.0133426 |
| AC007996.1 | 1.3079057 | 0.3963092 | 3.3002153 | 0.0009661 | 0.0133453 |
| C6orf132 | 1.0138791 | 0.3074003 | 3.2982378 | 0.0009729 | 0.0134183 |
| MCEMP1 | -1.396419 | 0.4233804 | -3.298261 | 0.0009729 | 0.0134183 |
| ANKUB1 | 2.151765 | 0.6525455 | 3.2974939 | 0.0009755 | 0.0134468 |
| AC007743.1 | -1.139641 | 0.3458528 | -3.295164 | 0.0009836 | 0.0135302 |
| F2RL3 | -1.75387 | 0.5323058 | -3.294855 | 0.0009847 | 0.013532 |
| AP001372.1 | 2.0759325 | 0.6303366 | 3.2933712 | 0.0009899 | 0.0135809 |
| CCL4 | -1.300632 | 0.3954569 | -3.288935 | 0.0010057 | 0.0137678 |
| ITPRID1 | 2.5778245 | 0.7840159 | 3.2879746 | 0.0010091 | 0.0138059 |
| AL390719.2 | 1.1236256 | 0.3418694 | 3.2867102 | 0.0010137 | 0.0138552 |
| BCO2 | 1.0782849 | 0.3281473 | 3.285978 | 0.0010163 | 0.0138767 |
| AC012317.1 | 6.3014967 | 1.9193714 | 3.2831045 | 0.0010267 | 0.0139975 |
| PDE11A | 1.6619619 | 0.5063555 | 3.282204 | 0.00103 | 0.0140344 |
| RNF32 | 1.1388489 | 0.3470405 | 3.2816028 | 0.0010322 | 0.014057 |
| ATP4B | 3.8318814 | 1.1679274 | 3.2809242 | 0.0010347 | 0.0140761 |
| DLL4 | -1.101011 | 0.3357753 | -3.279011 | 0.0010417 | 0.0141497 |
| AC092134.1 | 1.8371268 | 0.5602619 | 3.2790504 | 0.0010416 | 0.0141497 |
| CACNA1G | 1.2810498 | 0.3908197 | 3.277854 | 0.001046 | 0.0141946 |
| PLA2G1B | -1.502352 | 0.4583942 | -3.277425 | 0.0010476 | 0.0142073 |
| RBKS | 1.279514 | 0.3905255 | 3.2763903 | 0.0010514 | 0.014252 |
| KIF26B | 1.1759851 | 0.3589695 | 3.2760029 | 0.0010529 | 0.0142642 |
| TMEM17 | 1.0083644 | 0.3078748 | 3.2752416 | 0.0010557 | 0.0142878 |
| AC132942.1 | -1.063735 | 0.3248 | -3.275046 | 0.0010564 | 0.0142903 |
| SLC17A8 | 1.9499707 | 0.5955161 | 3.2744212 | 0.0010588 | 0.0143137 |
| FP236315.2 | 2.6493666 | 0.8091424 | 3.2742895 | 0.0010593 | 0.0143137 |
| RPS3AP16 | 2.1494533 | 0.6567278 | 3.2729744 | 0.0010642 | 0.0143731 |
| AC009070.1 | -3.953821 | 1.2087503 | -3.270999 | 0.0010717 | 0.0144589 |
| VN2R17P | 2.3348618 | 0.7138655 | 3.270731 | 0.0010727 | 0.0144651 |
| OLR1 | -1.2637 | 0.3865883 | -3.268852 | 0.0010799 | 0.0145314 |
| PAK6 | 1.0099138 | 0.3091306 | 3.2669492 | 0.0010871 | 0.0145918 |
| KRT42P | 1.3215189 | 0.4048302 | 3.2643781 | 0.001097 | 0.0146795 |
| APOD | 1.1725902 | 0.359347 | 3.2631143 | 0.001102 | 0.01473 |
| ARHGEF35-AS1 | -1.064051 | 0.3260812 | -3.263149 | 0.0011018 | 0.01473 |
| GZMB | -1.402051 | 0.4299589 | -3.260896 | 0.0011106 | 0.0148305 |
| AL590714.1 | 1.1498752 | 0.3528672 | 3.2586625 | 0.0011194 | 0.0149324 |
| WIF1 | -1.136495 | 0.348817 | -3.258141 | 0.0011214 | 0.0149523 |
| TMCO1-AS1 | 1.0288535 | 0.3158043 | 3.2578831 | 0.0011225 | 0.0149549 |
| RBM20 | 1.2692046 | 0.3895895 | 3.2578 | 0.0011228 | 0.0149549 |
| OSGIN1 | -1.0977 | 0.3369768 | -3.257494 | 0.001124 | 0.0149634 |
| LCA5 | 1.2974044 | 0.3983642 | 3.2568298 | 0.0011266 | 0.0149832 |
| AC009244.2 | -1.424664 | 0.4374681 | -3.256612 | 0.0011275 | 0.014987 |
| IGHV3-7 | 1.9327016 | 0.5935525 | 3.2561593 | 0.0011293 | 0.0150033 |
| RNF157-AS1 | 1.7907323 | 0.5499872 | 3.2559524 | 0.0011301 | 0.0150066 |
| DENND6B | 1.2249984 | 0.3765285 | 3.2534012 | 0.0011403 | 0.0151189 |
| C11orf96 | -1.347103 | 0.4140862 | -3.253195 | 0.0011412 | 0.0151191 |
| FGF14 | 1.0570104 | 0.3249426 | 3.2529138 | 0.0011423 | 0.0151212 |
| AP3B2 | 1.1988106 | 0.3685496 | 3.2527797 | 0.0011428 | 0.0151212 |
| IGKV3D-20 | 2.164274 | 0.6654149 | 3.2525179 | 0.0011439 | 0.0151275 |
| LILRA1 | -1.30496 | 0.4012879 | -3.25193 | 0.0011462 | 0.0151511 |
| DALRD3 | 1.0074195 | 0.3098703 | 3.2511008 | 0.0011496 | 0.0151794 |
| LY6D | 6.090083 | 1.8747761 | 3.2484322 | 0.0011604 | 0.0152998 |
| STRA6 | 1.6883934 | 0.5199472 | 3.2472402 | 0.0011653 | 0.0153485 |
| ALOX12P2 | 1.3480655 | 0.4152487 | 3.2464055 | 0.0011687 | 0.015378 |
| CYSRT1 | 1.4235783 | 0.438553 | 3.2460805 | 0.0011701 | 0.0153878 |
| AL118505.1 | 3.3268738 | 1.025427 | 3.2443789 | 0.0011771 | 0.0154567 |
| HECW2 | -1.199574 | 0.3698442 | -3.243459 | 0.0011809 | 0.0154989 |
| EMP2 | -1.107766 | 0.3415564 | -3.243287 | 0.0011816 | 0.0155004 |
| LINC01132 | 1.5241446 | 0.4699799 | 3.2429993 | 0.0011828 | 0.0155083 |
| AL033384.1 | 6.2391851 | 1.9242596 | 3.2423823 | 0.0011853 | 0.0155341 |
| ATP6V1B1 | 1.5672107 | 0.4835222 | 3.2412386 | 0.0011901 | 0.0155878 |
| GLS2 | 1.0765749 | 0.3321917 | 3.2408239 | 0.0011918 | 0.0155957 |
| SERPINB5 | 4.3719774 | 1.349347 | 3.2400691 | 0.001195 | 0.0156292 |
| AC242022.2 | 1.8225173 | 0.5626063 | 3.2394185 | 0.0011977 | 0.0156492 |
| EPHX3 | 1.0409748 | 0.3214863 | 3.2380072 | 0.0012037 | 0.015719 |
| EPPIN | 2.3408204 | 0.7236603 | 3.2346953 | 0.0012177 | 0.0158786 |
| AC131944.1 | 6.3082278 | 1.9511859 | 3.2330224 | 0.0012249 | 0.0159639 |
| TEX21P | 1.0892109 | 0.3369257 | 3.2327929 | 0.0012259 | 0.0159687 |
| ARHGEF10 | -1.121005 | 0.3468602 | -3.231863 | 0.0012299 | 0.0160128 |
| LINC01708 | 3.901707 | 1.2075406 | 3.2311187 | 0.0012331 | 0.0160465 |
| CLDN16 | 2.4982171 | 0.7734747 | 3.2298626 | 0.0012385 | 0.0160931 |
| MT1L | -1.247535 | 0.3865091 | -3.227698 | 0.0012479 | 0.0161911 |
| AC099063.4 | 1.2081327 | 0.3743951 | 3.2268921 | 0.0012514 | 0.0162287 |
| SPIRE2 | 1.0245753 | 0.3175464 | 3.2265376 | 0.001253 | 0.0162407 |
| KCNIP1 | -1.400116 | 0.4339767 | -3.226247 | 0.0012542 | 0.0162411 |
| TCTN2 | 1.0938651 | 0.3390513 | 3.2262519 | 0.0012542 | 0.0162411 |
| SLCO2A1 | -1.388773 | 0.4307138 | -3.224352 | 0.0012626 | 0.0163108 |
| LINC00598 | 1.14406 | 0.3547999 | 3.2245213 | 0.0012618 | 0.0163108 |
| SFN | 1.5219145 | 0.4720609 | 3.2239793 | 0.0012642 | 0.0163216 |
| MKX | 1.5766771 | 0.4892658 | 3.2225368 | 0.0012706 | 0.016388 |
| LINC00937 | -1.139474 | 0.3535958 | -3.222534 | 0.0012706 | 0.016388 |
| TREML2 | -1.332829 | 0.413819 | -3.220802 | 0.0012783 | 0.0164791 |
| BTG4 | 2.1289334 | 0.6611196 | 3.2201941 | 0.001281 | 0.016506 |
| RANBP3L | -1.161118 | 0.360627 | -3.21972 | 0.0012832 | 0.0165087 |
| AL009177.1 | 1.4194225 | 0.4408719 | 3.2195807 | 0.0012838 | 0.0165087 |
| AC027281.1 | 3.3483277 | 1.0398978 | 3.2198623 | 0.0012825 | 0.0165087 |
| DLX4 | 1.4394997 | 0.4471678 | 3.2191489 | 0.0012857 | 0.0165172 |
| AL160408.3 | 1.7381629 | 0.5400478 | 3.2185351 | 0.0012885 | 0.0165445 |
| APOBEC3A | -2.1658 | 0.6732794 | -3.216793 | 0.0012963 | 0.0166125 |
| RPP38-DT | 1.2490246 | 0.3887725 | 3.2127389 | 0.0013148 | 0.0167768 |
| PLCXD3 | -1.414323 | 0.4403131 | -3.212084 | 0.0013178 | 0.0168036 |
| AP002008.1 | 3.4237914 | 1.0659501 | 3.2119621 | 0.0013183 | 0.0168036 |
| STPG1 | 1.0888096 | 0.3393428 | 3.2085832 | 0.0013339 | 0.0169587 |
| DLK1 | 4.0794357 | 1.2714044 | 3.208606 | 0.0013338 | 0.0169587 |
| SYNDIG1L | -1.408703 | 0.439341 | -3.206401 | 0.0013441 | 0.0170473 |
| CPXCR1 | 6.0655351 | 1.8921002 | 3.2057156 | 0.0013473 | 0.0170643 |
| IGKV1-12 | 1.8399269 | 0.5740242 | 3.2053122 | 0.0013492 | 0.0170798 |
| LINC02331 | 3.4625944 | 1.0804429 | 3.2047917 | 0.0013516 | 0.0170941 |
| LYPD5 | -1.034462 | 0.3228006 | -3.204647 | 0.0013523 | 0.0170944 |
| AC078795.1 | 1.1242753 | 0.3510227 | 3.2028566 | 0.0013607 | 0.0171594 |
| MIR8083 | -1.354265 | 0.4231372 | -3.200533 | 0.0013717 | 0.0172874 |
| NCKAP5 | -1.239988 | 0.3874917 | -3.200038 | 0.0013741 | 0.017303 |
| TFAP2A | 3.1457583 | 0.9835819 | 3.1982676 | 0.0013826 | 0.0173509 |
| KCNJ2-AS1 | 1.2525535 | 0.3916214 | 3.1983786 | 0.001382 | 0.0173509 |
| AC009652.1 | 2.1280567 | 0.6661152 | 3.1947277 | 0.0013996 | 0.0175566 |
| CLSTN2 | 1.0266754 | 0.3214108 | 3.1942776 | 0.0014018 | 0.0175756 |
| AL449363.2 | 2.6070302 | 0.8165979 | 3.1925506 | 0.0014102 | 0.0176725 |
| IL17REL | 5.0249926 | 1.5742541 | 3.1919832 | 0.001413 | 0.0176903 |
| TPRG1 | -1.085117 | 0.3400082 | -3.191444 | 0.0014156 | 0.0177148 |
| AC106820.3 | 1.5068627 | 0.4721841 | 3.1912607 | 0.0014165 | 0.0177175 |
| KLK14 | 1.6461974 | 0.5159077 | 3.1908758 | 0.0014184 | 0.0177327 |
| AC092155.1 | 2.8582394 | 0.8958419 | 3.1905623 | 0.00142 | 0.0177349 |
| TUBB4B | 1.0645985 | 0.333665 | 3.1906203 | 0.0014197 | 0.0177349 |
| AL590438.1 | 2.2009426 | 0.6903718 | 3.1880542 | 0.0014323 | 0.0178403 |
| AL445685.3 | 4.011395 | 1.2584096 | 3.1876703 | 0.0014342 | 0.0178403 |
| LRRIQ3 | 1.8084548 | 0.5673396 | 3.1876054 | 0.0014346 | 0.0178403 |
| SPDEF | 2.6852528 | 0.8422897 | 3.1880393 | 0.0014324 | 0.0178403 |
| FEZF1 | 3.9251196 | 1.2312271 | 3.1879737 | 0.0014327 | 0.0178403 |
| LILRB2 | -1.127694 | 0.3537681 | -3.187664 | 0.0014343 | 0.0178403 |
| LINC02669 | 1.7358108 | 0.5447119 | 3.1866587 | 0.0014393 | 0.0178647 |
| AP000812.4 | 6.2135774 | 1.9498283 | 3.1867305 | 0.0014389 | 0.0178647 |
| RBP2 | -1.65177 | 0.5184362 | -3.186062 | 0.0014422 | 0.0178846 |
| IGHV1OR15-1 | 1.433913 | 0.4500393 | 3.1861949 | 0.0014416 | 0.0178846 |
| C3orf36 | -1.62506 | 0.5104475 | -3.183599 | 0.0014546 | 0.0179776 |
| ATP8B5P | -1.190608 | 0.3740138 | -3.183327 | 0.0014559 | 0.0179859 |
| TNNT3 | 1.5967684 | 0.5017505 | 3.182395 | 0.0014606 | 0.0180268 |
| AC090950.2 | -1.609141 | 0.5057646 | -3.181601 | 0.0014646 | 0.0180673 |
| HBG1 | -2.888752 | 0.9079927 | -3.18147 | 0.0014653 | 0.0180673 |
| ADCY8 | -1.705087 | 0.536702 | -3.176972 | 0.0014882 | 0.0182808 |
| INHBA | -1.125002 | 0.3541495 | -3.176631 | 0.00149 | 0.018285 |
| DIO1 | 1.4733351 | 0.4640592 | 3.1748859 | 0.001499 | 0.0183608 |
| CGB7 | 1.1789316 | 0.3713868 | 3.1744038 | 0.0015014 | 0.0183742 |
| ODAM | -1.376392 | 0.433658 | -3.173911 | 0.001504 | 0.0183967 |
| LGI3 | -1.268084 | 0.3997568 | -3.172139 | 0.0015132 | 0.0184857 |
| AC099518.4 | 1.9838749 | 0.6254137 | 3.1721001 | 0.0015134 | 0.0184857 |
| TGFBR3L | 1.9101808 | 0.6022459 | 3.171762 | 0.0015152 | 0.0184986 |
| TMEM200C | 1.6450305 | 0.5188427 | 3.1705765 | 0.0015214 | 0.0185395 |
| TLCD1 | 1.0945965 | 0.3454966 | 3.1681827 | 0.001534 | 0.0186841 |
| PDZD2 | -1.199472 | 0.3786602 | -3.167673 | 0.0015366 | 0.0187081 |
| KIF26B-AS1 | 4.1451349 | 1.3090827 | 3.1664424 | 0.0015432 | 0.0187267 |
| LINC01123 | 1.0279979 | 0.3246348 | 3.1666284 | 0.0015422 | 0.0187267 |
| CT75 | -1.986845 | 0.6273443 | -3.167073 | 0.0015398 | 0.0187267 |
| ALDH1A3 | 1.7754512 | 0.5607097 | 3.1664354 | 0.0015432 | 0.0187267 |
| MIR9-3HG | 1.0772556 | 0.3404629 | 3.1640907 | 0.0015557 | 0.0188431 |
| HPGD | -1.422339 | 0.4496583 | -3.163156 | 0.0015607 | 0.0188862 |
| CFAP298-TCP10L | 1.1798569 | 0.3729937 | 3.1632089 | 0.0015604 | 0.0188862 |
| ALAS2 | -2.50894 | 0.7935781 | -3.161554 | 0.0015693 | 0.0189464 |
| NXF2 | 1.9116135 | 0.605221 | 3.1585381 | 0.0015856 | 0.0191186 |
| TACR1 | 1.2561133 | 0.3978022 | 3.1576326 | 0.0015906 | 0.0191587 |
| CDH19 | -1.168421 | 0.3701802 | -3.156358 | 0.0015975 | 0.0192249 |
| SPOCK2 | -1.352531 | 0.428541 | -3.15613 | 0.0015988 | 0.0192311 |
| MZB1 | 1.4618306 | 0.4636845 | 3.1526404 | 0.001618 | 0.019425 |
| AL590822.3 | 1.3775578 | 0.437119 | 3.1514478 | 0.0016246 | 0.0194702 |
| TNR | -2.348057 | 0.7450607 | -3.151497 | 0.0016244 | 0.0194702 |
| AL359736.1 | 2.5905077 | 0.8221816 | 3.1507733 | 0.0016284 | 0.0195062 |
| NAPSA | -1.096053 | 0.348117 | -3.148518 | 0.001641 | 0.0196123 |
| CCDC68 | -1.050144 | 0.3335831 | -3.148072 | 0.0016435 | 0.0196333 |
| CYP2A13 | 2.6657117 | 0.8469263 | 3.1475133 | 0.0016467 | 0.0196618 |
| AL159156.1 | -2.402997 | 0.7639342 | -3.145555 | 0.0016577 | 0.0197758 |
| TRPV6 | 1.2919984 | 0.4109491 | 3.1439376 | 0.0016669 | 0.0198582 |
| AC013268.1 | 2.825822 | 0.8989008 | 3.1436417 | 0.0016686 | 0.0198692 |
| CX3CR1 | -1.454539 | 0.4631626 | -3.14045 | 0.0016869 | 0.0200595 |
| SPINK1 | 3.6765966 | 1.1706843 | 3.1405535 | 0.0016863 | 0.0200595 |
| AC068418.3 | 6.0747463 | 1.9347462 | 3.1398156 | 0.0016905 | 0.0200938 |
| LINC01088 | -1.146658 | 0.3652176 | -3.139659 | 0.0016914 | 0.0200954 |
| CLDN9 | 1.3384875 | 0.4263638 | 3.1393082 | 0.0016935 | 0.0201012 |
| FFAR2 | -1.657087 | 0.527851 | -3.139307 | 0.0016935 | 0.0201012 |
| CDRT1 | 1.0801356 | 0.3441882 | 3.1382124 | 0.0016998 | 0.0201672 |
| AC087752.3 | 1.2413179 | 0.3957635 | 3.1365146 | 0.0017097 | 0.0202663 |
| AC091806.1 | -1.841407 | 0.5871132 | -3.136375 | 0.0017105 | 0.0202663 |
| SLC38A11 | 1.8062864 | 0.5759791 | 3.1360274 | 0.0017125 | 0.0202812 |
| RNU6-343P | -1.253322 | 0.3997701 | -3.135107 | 0.0017179 | 0.0203357 |
| GSDMC | 2.1369975 | 0.6818672 | 3.1340378 | 0.0017242 | 0.0203833 |
| SEMA3G | -1.813879 | 0.5791086 | -3.132191 | 0.0017351 | 0.0204922 |
| REM2 | 1.1568249 | 0.3693758 | 3.1318369 | 0.0017372 | 0.0204984 |
| FBXO2 | 1.1399279 | 0.3641436 | 3.1304349 | 0.0017455 | 0.0205689 |
| AC020922.1 | 4.5070713 | 1.4397576 | 3.1304377 | 0.0017455 | 0.0205689 |
| AC113349.1 | 1.8014333 | 0.575543 | 3.1299719 | 0.0017482 | 0.0205917 |
| NXF2B | 3.7803181 | 1.2080968 | 3.1291515 | 0.0017531 | 0.0206399 |
| AC018557.3 | 1.3550658 | 0.433151 | 3.1283913 | 0.0017577 | 0.0206747 |
| NRK | 1.2529595 | 0.4005069 | 3.1284345 | 0.0017574 | 0.0206747 |
| BEX1 | -1.204648 | 0.385306 | -3.12647 | 0.0017692 | 0.0208009 |
| AC105206.1 | -1.588859 | 0.5083364 | -3.125604 | 0.0017744 | 0.020834 |
| AFF3 | -1.050662 | 0.336192 | -3.125186 | 0.0017769 | 0.0208543 |
| TCTEX1D4 | 1.8548522 | 0.5935941 | 3.1247822 | 0.0017794 | 0.0208641 |
| RFX2 | 1.0456201 | 0.334902 | 3.1221675 | 0.0017952 | 0.0209937 |
| AC016394.2 | 1.0667467 | 0.3416996 | 3.1218846 | 0.001797 | 0.0210044 |
| SMIM5 | 1.1674324 | 0.374017 | 3.1213349 | 0.0018003 | 0.0210343 |
| TTLL10-AS1 | 1.3603003 | 0.4359594 | 3.1202457 | 0.001807 | 0.0210839 |
| PRRX1 | 1.5147727 | 0.4856418 | 3.1191148 | 0.001814 | 0.021146 |
| ACVRL1 | -1.192231 | 0.3822754 | -3.118775 | 0.001816 | 0.0211568 |
| SMOC1 | 1.0763018 | 0.3451123 | 3.1187005 | 0.0018165 | 0.0211568 |
| LINC02284 | -1.711086 | 0.548733 | -3.118248 | 0.0018193 | 0.0211799 |
| PCDH19 | 1.2542296 | 0.4023109 | 3.1175631 | 0.0018235 | 0.0212197 |
| PPP1R1A | 1.0354401 | 0.3322007 | 3.1169112 | 0.0018276 | 0.0212572 |
| AC018553.1 | 1.1198713 | 0.3595575 | 3.1145823 | 0.0018421 | 0.0213875 |
| AC023510.2 | 1.306195 | 0.4194161 | 3.1143175 | 0.0018437 | 0.0213879 |
| PRND | 1.9432875 | 0.624156 | 3.1134645 | 0.001849 | 0.021424 |
| CCL24 | -1.371939 | 0.4413032 | -3.108835 | 0.0018783 | 0.0217209 |
| AC093635.1 | 1.6431837 | 0.5288608 | 3.1070249 | 0.0018898 | 0.0218446 |
| MSMB | 5.4701656 | 1.760791 | 3.1066524 | 0.0018922 | 0.0218528 |
| SMIM25 | -1.032952 | 0.3324928 | -3.106692 | 0.0018919 | 0.0218528 |
| ULBP2 | -1.037361 | 0.3340972 | -3.104969 | 0.001903 | 0.0219581 |
| TMC3 | 2.5750926 | 0.8296253 | 3.1039224 | 0.0019097 | 0.0220104 |
| MGAT5B | -1.487041 | 0.479092 | -3.103873 | 0.0019101 | 0.0220104 |
| SMCR2 | 5.875241 | 1.8960279 | 3.0987102 | 0.0019437 | 0.0223394 |
| GPR135 | 1.1452341 | 0.3698924 | 3.096128 | 0.0019607 | 0.0224633 |
| XDH | 1.6259398 | 0.5256117 | 3.0934238 | 0.0019786 | 0.0226326 |
| AC010422.2 | 1.3759072 | 0.4451099 | 3.0911632 | 0.0019937 | 0.0227734 |
| SCEL | -1.249934 | 0.4044521 | -3.090437 | 0.0019986 | 0.0227992 |
| GLT1D1 | -1.197332 | 0.3876226 | -3.088912 | 0.0020089 | 0.0228864 |
| PRSS12 | 1.1036101 | 0.3573256 | 3.0885282 | 0.0020115 | 0.022906 |
| FMO6P | 1.4025436 | 0.4541994 | 3.0879466 | 0.0020154 | 0.0229309 |
| AC010478.1 | 3.2106562 | 1.0399636 | 3.0872776 | 0.00202 | 0.0229525 |
| AC007877.1 | -1.202246 | 0.3895218 | -3.086468 | 0.0020255 | 0.0229951 |
| E2F7 | 1.2375039 | 0.4009954 | 3.0860802 | 0.0020281 | 0.023015 |
| AC021127.1 | 2.0398511 | 0.6613353 | 3.0844433 | 0.0020393 | 0.023132 |
| PLEKHH2 | -1.019701 | 0.3306411 | -3.084012 | 0.0020423 | 0.0231555 |
| CACNA2D2 | -1.029169 | 0.3338125 | -3.083076 | 0.0020487 | 0.0231914 |
| SPOCK1 | 1.3289194 | 0.4310428 | 3.0830334 | 0.002049 | 0.0231914 |
| HSD11B1 | -1.105744 | 0.3587648 | -3.082085 | 0.0020556 | 0.0232436 |
| AC023300.2 | 2.6960746 | 0.8749003 | 3.0815791 | 0.0020591 | 0.0232545 |
| MAFG-DT | 1.2255142 | 0.3977418 | 3.0811805 | 0.0020618 | 0.0232756 |
| AC023296.1 | 3.1365875 | 1.0195191 | 3.0765363 | 0.0020942 | 0.0235902 |
| LINC02031 | 3.2910168 | 1.0705936 | 3.0740112 | 0.002112 | 0.023719 |
| AC118755.2 | 3.3473448 | 1.0890265 | 3.0737037 | 0.0021142 | 0.023733 |
| PTCH2 | 1.0536188 | 0.3428672 | 3.0729648 | 0.0021194 | 0.0237569 |
| MIR3945HG | -1.339182 | 0.4357777 | -3.073086 | 0.0021186 | 0.0237569 |
| TNF | -1.806349 | 0.5878834 | -3.072631 | 0.0021218 | 0.0237675 |
| AC011700.1 | 1.9758279 | 0.6438186 | 3.0689202 | 0.0021483 | 0.0240131 |
| LINC01091 | 1.0407691 | 0.3391216 | 3.069014 | 0.0021477 | 0.0240131 |
| GZMH | -1.241191 | 0.4045142 | -3.068348 | 0.0021525 | 0.024031 |
| BX470102.1 | -1.114379 | 0.3634629 | -3.066005 | 0.0021694 | 0.0241862 |
| FCN1 | -1.214623 | 0.3963001 | -3.064908 | 0.0021774 | 0.0242567 |
| SIGLEC5 | -1.023797 | 0.3342448 | -3.063016 | 0.0021912 | 0.0243355 |
| SAA2-SAA4 | 6.047764 | 1.9753717 | 3.0615828 | 0.0022017 | 0.0244315 |
| AC055714.1 | 2.0346496 | 0.6647524 | 3.0607633 | 0.0022077 | 0.0244677 |
| IGLC1 | 2.6436447 | 0.8637226 | 3.0607567 | 0.0022078 | 0.0244677 |
| AC022613.2 | -1.025666 | 0.3351774 | -3.060069 | 0.0022129 | 0.0245136 |
| ST8SIA5 | 2.2697133 | 0.742203 | 3.0580762 | 0.0022276 | 0.0246458 |
| UNC5B-AS1 | 2.0198303 | 0.6607415 | 3.0569146 | 0.0022363 | 0.0246995 |
| AL049555.1 | 2.9349759 | 0.9606377 | 3.0552369 | 0.0022488 | 0.0247961 |
| SCAMP5 | 1.076694 | 0.3526536 | 3.0531205 | 0.0022648 | 0.0249505 |
| TMEM61 | 1.6698289 | 0.5470372 | 3.0524962 | 0.0022695 | 0.0249603 |
| AL035420.3 | 2.7304035 | 0.8944798 | 3.0525045 | 0.0022694 | 0.0249603 |
| AL109947.1 | 2.840063 | 0.9307546 | 3.0513552 | 0.0022781 | 0.0249921 |
| STXBP6 | -1.257846 | 0.412478 | -3.049485 | 0.0022923 | 0.0251264 |
| OASL | -1.218274 | 0.3996366 | -3.048454 | 0.0023002 | 0.0251711 |
| AC018629.1 | 2.0049022 | 0.6577859 | 3.0479558 | 0.002304 | 0.0251917 |
| FAM181B | 1.7422987 | 0.5716933 | 3.047611 | 0.0023067 | 0.0252101 |
| AC121758.2 | 2.9040873 | 0.9532283 | 3.046581 | 0.0023146 | 0.0252648 |
| CHRM1 | -1.610905 | 0.5291572 | -3.044284 | 0.0023324 | 0.0253863 |
| LONRF3 | -1.096195 | 0.3601295 | -3.04389 | 0.0023354 | 0.0254068 |
| HEG1 | -1.069976 | 0.351824 | -3.041226 | 0.0023562 | 0.02559 |
| AC105101.2 | 2.1271084 | 0.7000507 | 3.0385064 | 0.0023775 | 0.0257791 |
| RNF224 | 1.3094499 | 0.4310704 | 3.0376707 | 0.0023841 | 0.0258078 |
| AL136528.2 | 3.7291969 | 1.2277106 | 3.0375211 | 0.0023853 | 0.0258099 |
| SELL | -1.306475 | 0.4301966 | -3.036924 | 0.0023901 | 0.0258503 |
| GRHL3 | 1.9508431 | 0.6424493 | 3.0365712 | 0.0023929 | 0.0258698 |
| PTGS2 | -1.653903 | 0.5447331 | -3.036172 | 0.002396 | 0.0258827 |
| AC104162.1 | 1.7278966 | 0.569216 | 3.0355729 | 0.0024008 | 0.0258915 |
| FBN3 | -1.064833 | 0.3510382 | -3.033382 | 0.0024183 | 0.0260359 |
| HYDIN2 | 1.3332723 | 0.4399362 | 3.030604 | 0.0024407 | 0.0262234 |
| C1orf141 | 1.5898891 | 0.5246543 | 3.0303554 | 0.0024427 | 0.0262342 |
| TNFSF11 | 1.7816431 | 0.5879889 | 3.0300624 | 0.002445 | 0.0262488 |
| LINC02185 | -1.261294 | 0.4163252 | -3.02959 | 0.0024489 | 0.0262791 |
| AC026191.1 | 1.0616401 | 0.3505069 | 3.0288706 | 0.0024547 | 0.0263309 |
| AL137847.1 | 2.8708852 | 0.9479893 | 3.0283943 | 0.0024586 | 0.0263488 |
| ICAM2 | -1.05879 | 0.3499402 | -3.025633 | 0.0024811 | 0.0265272 |
| PPP1R14C | 1.0212843 | 0.3376989 | 3.0242454 | 0.0024925 | 0.0265946 |
| AC010931.1 | 1.4791229 | 0.4890706 | 3.0243544 | 0.0024916 | 0.0265946 |
| DNMBP-AS1 | 1.7803814 | 0.5887806 | 3.0238451 | 0.0024958 | 0.0266189 |
| CT45A5 | -3.624205 | 1.1992441 | -3.022075 | 0.0025105 | 0.0267525 |
| BFSP2 | 4.4046331 | 1.4581559 | 3.0206874 | 0.002522 | 0.0268431 |
| SP6 | -1.325853 | 0.4389763 | -3.020328 | 0.002525 | 0.026864 |
| BTNL8 | -1.573011 | 0.5208383 | -3.020152 | 0.0025265 | 0.0268685 |
| PRSS2 | 4.8650294 | 1.6109836 | 3.0199124 | 0.0025285 | 0.0268685 |
| AC009093.10 | 1.3177112 | 0.4365786 | 3.0182681 | 0.0025422 | 0.0269483 |
| KRT6A | 5.1557084 | 1.7091743 | 3.0164908 | 0.0025572 | 0.0270848 |
| AC010255.1 | 2.399308 | 0.7956732 | 3.0154439 | 0.002566 | 0.0271496 |
| KCNQ3 | 1.1297822 | 0.3746643 | 3.0154516 | 0.002566 | 0.0271496 |
| PENK | 1.3268653 | 0.4400882 | 3.0149985 | 0.0025698 | 0.0271743 |
| SLC27A6 | 1.4058914 | 0.4663283 | 3.0148104 | 0.0025714 | 0.0271801 |
| COL25A1 | 1.4784188 | 0.4907006 | 3.0128732 | 0.0025879 | 0.0273099 |
| RADX | 1.1530438 | 0.3827981 | 3.0121458 | 0.0025941 | 0.0273517 |
| AC243772.2 | 1.1897584 | 0.3951213 | 3.0111217 | 0.0026028 | 0.0273793 |
| GUCY1A2 | -1.087207 | 0.3610612 | -3.011144 | 0.0026027 | 0.0273793 |
| AC091214.1 | 1.9300695 | 0.6409802 | 3.0111216 | 0.0026028 | 0.0273793 |
| AL033519.4 | -1.985255 | 0.6593499 | -3.010928 | 0.0026045 | 0.0273857 |
| PHOSPHO1 | -1.426814 | 0.4739825 | -3.010268 | 0.0026102 | 0.0274342 |
| AC004846.2 | 1.1097438 | 0.3687619 | 3.0093778 | 0.0026178 | 0.0274848 |
| RNF7P1 | 2.3938145 | 0.7959573 | 3.0074659 | 0.0026344 | 0.0276167 |
| ACOT4 | -1.030007 | 0.3425705 | -3.0067 | 0.002641 | 0.0276579 |
| CD244 | -1.000656 | 0.3329932 | -3.005035 | 0.0026555 | 0.0277491 |
| TUSC8 | 3.7366386 | 1.2440379 | 3.0036373 | 0.0026677 | 0.027826 |
| AC018450.1 | -2.649083 | 0.8821658 | -3.002931 | 0.0026739 | 0.0278683 |
| IGHV1-2 | 2.3709653 | 0.7895309 | 3.0030052 | 0.0026733 | 0.0278683 |
| AL034397.2 | -4.08845 | 1.3619572 | -3.001893 | 0.0026831 | 0.0279523 |
| FAM3D | 1.4477582 | 0.4824827 | 3.000643 | 0.0026941 | 0.0279963 |
| CTSE | 1.0016821 | 0.3339204 | 2.9997635 | 0.0027019 | 0.0280476 |
| R3HDML | 2.4335195 | 0.8124591 | 2.9952515 | 0.0027422 | 0.0284206 |
| LAMC3 | -1.199467 | 0.4005331 | -2.994675 | 0.0027474 | 0.0284291 |
| AC068896.3 | 1.5618088 | 0.5216662 | 2.9938852 | 0.0027545 | 0.0284464 |
| SDR42E2 | 1.54076 | 0.5149117 | 2.9922799 | 0.002769 | 0.0285738 |
| AC097493.1 | 2.0844971 | 0.6968187 | 2.9914484 | 0.0027766 | 0.028629 |
| TPTE2P2 | 1.8421903 | 0.6157974 | 2.9915528 | 0.0027756 | 0.028629 |
| AADACP1 | 1.2430292 | 0.4156774 | 2.9903697 | 0.0027864 | 0.0286963 |
| MOXD1 | 1.119079 | 0.3743011 | 2.9897826 | 0.0027918 | 0.0287401 |
| COX6B2 | 1.96771 | 0.6582462 | 2.9893225 | 0.002796 | 0.0287721 |
| SLC7A10 | 3.9251604 | 1.3138687 | 2.987483 | 0.0028128 | 0.028923 |
| AC034139.1 | 1.7453283 | 0.5842597 | 2.9872477 | 0.002815 | 0.0289338 |
| DOK5 | 1.5023158 | 0.5033629 | 2.9845577 | 0.0028399 | 0.029132 |
| IGHJ5 | 2.3249548 | 0.7794667 | 2.9827504 | 0.0028567 | 0.0292356 |
| RBBP8NL | 1.1377491 | 0.3816698 | 2.9809773 | 0.0028733 | 0.0293823 |
| AC036214.4 | 1.8272449 | 0.6134774 | 2.9785043 | 0.0028966 | 0.0295856 |
| CAPNS2 | 1.8425281 | 0.6187477 | 2.9778344 | 0.0029029 | 0.0296388 |
| LINC02299 | 1.8993701 | 0.6379016 | 2.9775283 | 0.0029058 | 0.0296502 |
| LINC01484 | 1.1455505 | 0.3851794 | 2.9740702 | 0.0029388 | 0.0299333 |
| LINC02863 | -1.626194 | 0.5468856 | -2.973554 | 0.0029437 | 0.0299381 |
| AL132801.2 | 4.1813778 | 1.4067674 | 2.9723305 | 0.0029555 | 0.0300342 |
| AL589765.7 | 2.3083667 | 0.7768147 | 2.9715798 | 0.0029627 | 0.0300842 |
| LINC01277 | -1.36568 | 0.459728 | -2.970626 | 0.0029719 | 0.0301543 |
| FIBCD1 | -1.051413 | 0.3539329 | -2.970656 | 0.0029716 | 0.0301543 |
| CORT | 1.1835922 | 0.3986002 | 2.9693719 | 0.0029841 | 0.0302541 |
| AC005532.2 | 1.9413266 | 0.6545495 | 2.9658972 | 0.003018 | 0.0305742 |
| FCRL4 | 3.9507252 | 1.332584 | 2.96471 | 0.0030297 | 0.0306686 |
| AL358113.1 | 1.2797614 | 0.4316992 | 2.9644747 | 0.003032 | 0.0306802 |
| AC099521.2 | 1.510825 | 0.5099419 | 2.9627392 | 0.0030491 | 0.0308082 |
| COL22A1 | -1.157605 | 0.3908405 | -2.961834 | 0.0030581 | 0.0308267 |
| CLEC4E | -1.578516 | 0.5329556 | -2.961815 | 0.0030583 | 0.0308267 |
| TENT5B | -1.127023 | 0.3806085 | -2.961108 | 0.0030653 | 0.0308618 |
| MKRN2OS | 1.3755762 | 0.4647248 | 2.9599801 | 0.0030766 | 0.0309392 |
| AL121992.1 | 1.7227547 | 0.5821583 | 2.9592545 | 0.0030838 | 0.0310002 |
| GUCY2C | 1.6450303 | 0.5560126 | 2.9586206 | 0.0030902 | 0.031052 |
| ISL1 | 3.5724378 | 1.2078858 | 2.9575957 | 0.0031005 | 0.031131 |
| LINC00648 | -2.542392 | 0.8596323 | -2.957534 | 0.0031011 | 0.031131 |
| CHST5 | 1.8581558 | 0.6283018 | 2.9574253 | 0.0031022 | 0.031131 |
| LINC00885 | 1.0121339 | 0.342309 | 2.9567842 | 0.0031087 | 0.0311381 |
| BDH1 | 1.0422117 | 0.3524788 | 2.9568069 | 0.0031084 | 0.0311381 |
| AL596442.2 | -1.664698 | 0.5630259 | -2.956699 | 0.0031095 | 0.0311381 |
| S100A12 | -2.90201 | 0.9816847 | -2.956153 | 0.003115 | 0.0311575 |
| AC104590.1 | 1.6479113 | 0.5577749 | 2.954438 | 0.0031324 | 0.0313071 |
| FABP4 | -1.275133 | 0.4316788 | -2.953892 | 0.0031379 | 0.0313265 |
| HTRA4 | 1.7443683 | 0.5908712 | 2.9521971 | 0.0031552 | 0.0314508 |
| TRIM31 | 2.4875661 | 0.8429101 | 2.9511643 | 0.0031658 | 0.0315286 |
| FAM187A | 1.6001233 | 0.5423134 | 2.9505512 | 0.0031721 | 0.0315465 |
| AL356608.3 | 1.7127974 | 0.5805519 | 2.9502918 | 0.0031747 | 0.0315489 |
| AC111149.2 | 5.8521424 | 1.9848835 | 2.9483556 | 0.0031947 | 0.0316783 |
| OVCH1 | -1.254321 | 0.4254357 | -2.948321 | 0.0031951 | 0.0316783 |
| WNT10A | 1.1707783 | 0.3972138 | 2.9474761 | 0.0032038 | 0.0317194 |
| CCDC178 | 1.6768721 | 0.5689757 | 2.9471769 | 0.0032069 | 0.0317354 |
| IGHV3-49 | 1.8647674 | 0.6331222 | 2.9453514 | 0.0032259 | 0.0318636 |
| CX3CL1 | -1.016117 | 0.3449957 | -2.945302 | 0.0032264 | 0.0318636 |
| AJAP1 | 1.3762368 | 0.467322 | 2.9449431 | 0.0032301 | 0.0318807 |
| CABYR | 1.1082289 | 0.3765671 | 2.942979 | 0.0032507 | 0.0320109 |
| GGT1 | -1.154776 | 0.3923794 | -2.943008 | 0.0032504 | 0.0320109 |
| AC004593.1 | 1.7199725 | 0.5844667 | 2.9428069 | 0.0032525 | 0.0320166 |
| LINC00271 | 1.1686104 | 0.3972304 | 2.9418957 | 0.0032621 | 0.0320885 |
| MEOX1 | 1.5201953 | 0.5168677 | 2.9411689 | 0.0032698 | 0.03215 |
| CLCA2 | 3.2325899 | 1.0999121 | 2.9389529 | 0.0032932 | 0.0323441 |
| CALCA | 2.0579035 | 0.7007768 | 2.9366034 | 0.0033183 | 0.0325289 |
| VWA8-AS1 | 2.4357198 | 0.8301333 | 2.9341311 | 0.0033448 | 0.0327192 |
| DQX1 | 2.0080705 | 0.6846363 | 2.9330471 | 0.0033565 | 0.032771 |
| AC008163.1 | 4.5794546 | 1.5612355 | 2.9332247 | 0.0033546 | 0.032771 |
| C9orf163 | 2.0282963 | 0.6916641 | 2.9324875 | 0.0033626 | 0.0328151 |
| PMS2P9 | 1.66298 | 0.5671614 | 2.9321105 | 0.0033667 | 0.0328427 |
| C1QTNF8 | 1.8001887 | 0.6143507 | 2.9302295 | 0.0033871 | 0.0330051 |
| TXK | -1.137992 | 0.3886245 | -2.928257 | 0.0034087 | 0.0331656 |
| ACAN | 1.4567653 | 0.4976428 | 2.9273311 | 0.0034188 | 0.0332397 |
| CPNE5 | 1.0866839 | 0.3712506 | 2.9270901 | 0.0034215 | 0.0332531 |
| OR7E36P | 1.8289842 | 0.6249836 | 2.9264514 | 0.0034285 | 0.0332966 |
| AC112229.1 | 2.1926804 | 0.7495439 | 2.925353 | 0.0034407 | 0.0333646 |
| HTR3E | 4.068502 | 1.3907382 | 2.9254262 | 0.0034398 | 0.0333646 |
| AC069120.1 | 2.3492372 | 0.803342 | 2.9243302 | 0.003452 | 0.0334372 |
| SIGLEC11 | -1.001607 | 0.3425327 | -2.92412 | 0.0034543 | 0.0334474 |
| JAKMIP2-AS1 | 4.0955447 | 1.4015644 | 2.9221239 | 0.0034765 | 0.0336386 |
| AL121950.1 | 3.6342548 | 1.2437077 | 2.9221134 | 0.0034767 | 0.0336386 |
| RGCC | -1.047291 | 0.3584227 | -2.921944 | 0.0034785 | 0.0336444 |
| FRMPD2B | 1.5603529 | 0.5342391 | 2.9207013 | 0.0034924 | 0.0337663 |
| WWOX-AS1 | 2.7528238 | 0.9426556 | 2.9202859 | 0.0034971 | 0.0337899 |
| GJA5 | -1.161768 | 0.3978659 | -2.919998 | 0.0035003 | 0.0337925 |
| FRMD5 | 1.4367484 | 0.492271 | 2.9186128 | 0.0035159 | 0.0339053 |
| AC140479.2 | 1.5490273 | 0.5312534 | 2.9157976 | 0.0035478 | 0.0341118 |
| IGLV2-14 | 2.2355021 | 0.766686 | 2.9157988 | 0.0035478 | 0.0341118 |
| FDPSP2 | 2.2670539 | 0.7785898 | 2.9117437 | 0.0035942 | 0.0345067 |
| TSLP | -1.241064 | 0.4262576 | -2.911536 | 0.0035966 | 0.0345169 |
| CD207 | 1.1212118 | 0.3853462 | 2.9096217 | 0.0036187 | 0.0347163 |
| IGLV4-69 | 2.0135087 | 0.692309 | 2.9083959 | 0.0036329 | 0.0348101 |
| MIR132 | 1.7131011 | 0.5891653 | 2.907675 | 0.0036413 | 0.0348689 |
| CRISP2 | 2.3916202 | 0.8228296 | 2.9065799 | 0.003654 | 0.0349655 |
| MPIG6B | -1.380542 | 0.4750005 | -2.906402 | 0.0036561 | 0.0349725 |
| LINC01488 | -2.751464 | 0.9473222 | -2.904465 | 0.0036788 | 0.0351124 |
| ROR1-AS1 | 2.3196701 | 0.7987802 | 2.9040154 | 0.0036841 | 0.03515 |
| ANKRD18B | 4.0132482 | 1.3820433 | 2.9038512 | 0.003686 | 0.0351556 |
| PRLR | -1.62397 | 0.5593632 | -2.903248 | 0.0036931 | 0.0352105 |
| KCNK17 | -1.126237 | 0.3880331 | -2.902425 | 0.0037029 | 0.0352515 |
| MGAT4D | 2.4582767 | 0.8472286 | 2.9015505 | 0.0037132 | 0.0353243 |
| ADPRS | 1.0127075 | 0.3490379 | 2.9014254 | 0.0037147 | 0.0353255 |
| DLG5-AS1 | 1.2922519 | 0.4454043 | 2.9013009 | 0.0037162 | 0.035326 |
| PLAC4 | 2.3971782 | 0.8262734 | 2.9011924 | 0.0037175 | 0.035326 |
| PSAPL1 | -1.922223 | 0.662695 | -2.900614 | 0.0037243 | 0.0353654 |
| FAM27E3 | 1.2700711 | 0.4379936 | 2.8997484 | 0.0037346 | 0.0354375 |
| BX255923.2 | 3.2069972 | 1.1070471 | 2.8968931 | 0.0037688 | 0.0356577 |
| SEC14L3 | 1.3956755 | 0.4818268 | 2.896633 | 0.0037719 | 0.0356744 |
| GPAA1P2 | 1.3821605 | 0.4772095 | 2.8963389 | 0.0037754 | 0.0356949 |
| LINC02832 | 3.4127104 | 1.1794576 | 2.8934575 | 0.0038103 | 0.0359396 |
| AL355297.3 | 1.1843944 | 0.4093228 | 2.8935459 | 0.0038092 | 0.0359396 |
| MOGAT2 | 3.3905528 | 1.1725728 | 2.8915499 | 0.0038335 | 0.0361124 |
| TMEM40 | 1.6016545 | 0.5543473 | 2.8892617 | 0.0038615 | 0.0363107 |
| CADM2 | 1.529894 | 0.5295467 | 2.8890632 | 0.0038639 | 0.0363205 |
| MAPK4 | -1.082056 | 0.3745524 | -2.88893 | 0.0038656 | 0.0363229 |
| LINC01607 | 2.9524558 | 1.0220414 | 2.8887829 | 0.0038674 | 0.0363267 |
| MS4A2 | 1.4041055 | 0.4862668 | 2.887521 | 0.0038829 | 0.0364237 |
| PTP4A1 | 1.0510908 | 0.3642574 | 2.8855714 | 0.003907 | 0.0365545 |
| C16orf46 | 1.153102 | 0.399643 | 2.8853298 | 0.00391 | 0.0365694 |
| AL021918.4 | 2.9139828 | 1.0110937 | 2.8820108 | 0.0039515 | 0.0368775 |
| MIR22HG | -1.272312 | 0.4415742 | -2.88131 | 0.0039603 | 0.0369199 |
| AC104389.6 | -2.596961 | 0.9016291 | -2.880299 | 0.003973 | 0.0369856 |
| PMS2P11 | 1.141201 | 0.3962838 | 2.8797568 | 0.0039798 | 0.0370361 |
| MIR6891 | -1.397868 | 0.4855433 | -2.878978 | 0.0039897 | 0.0371012 |
| AP002364.1 | 1.682051 | 0.5843175 | 2.878659 | 0.0039937 | 0.0371104 |
| FUT1 | -1.068751 | 0.3712762 | -2.878587 | 0.0039946 | 0.0371104 |
| ITGA2B | -1.033309 | 0.3590612 | -2.877808 | 0.0040045 | 0.0371823 |
| AL160408.6 | 1.7391926 | 0.6050638 | 2.8743953 | 0.004048 | 0.0375473 |
| Z97192.4 | -1.522909 | 0.5298355 | -2.874306 | 0.0040492 | 0.0375473 |
| ST18 | 1.1658026 | 0.4057278 | 2.8733615 | 0.0040613 | 0.0376464 |
| DNAH1 | 1.0639792 | 0.370497 | 2.871762 | 0.0040819 | 0.0377867 |
| MMP12 | 3.4864167 | 1.2140316 | 2.8717677 | 0.0040818 | 0.0377867 |
| LINC01927 | 2.6468451 | 0.9220677 | 2.870554 | 0.0040975 | 0.0378477 |
| FOXI2 | 2.3330479 | 0.8128628 | 2.8701621 | 0.0041026 | 0.0378796 |
| SHISA8 | 3.0157497 | 1.0509324 | 2.8695944 | 0.00411 | 0.037909 |
| IGKV1-39 | 1.8487369 | 0.6444094 | 2.8688855 | 0.0041192 | 0.0379621 |
| PCDH20 | 1.5473103 | 0.5393216 | 2.8689935 | 0.0041178 | 0.0379621 |
| AC117395.1 | 1.8041753 | 0.6293788 | 2.8665969 | 0.0041491 | 0.0381888 |
| CYP24A1 | 3.6428928 | 1.2709917 | 2.8661813 | 0.0041546 | 0.0382006 |
| RIMS4 | -1.19001 | 0.4157275 | -2.862477 | 0.0042034 | 0.0385316 |
| FCGR3B | -1.911265 | 0.6679684 | -2.861311 | 0.0042189 | 0.0386405 |
| SCD | -1.003954 | 0.3509182 | -2.860934 | 0.004224 | 0.0386592 |
| IGFL4 | 1.3472096 | 0.4708821 | 2.861034 | 0.0042226 | 0.0386592 |
| B3GALT5-AS1 | 2.4413592 | 0.8535478 | 2.8602491 | 0.0042331 | 0.0386942 |
| CLEC4GP1 | -2.400107 | 0.8393961 | -2.859326 | 0.0042454 | 0.0387877 |
| P3H2 | -1.072715 | 0.3753554 | -2.857865 | 0.004265 | 0.0389045 |
| RPL30P7 | 1.550958 | 0.5430715 | 2.8559001 | 0.0042915 | 0.0390202 |
| AL121694.1 | 2.6285852 | 0.9202889 | 2.856261 | 0.0042866 | 0.0390202 |
| LINC02099 | 3.668706 | 1.2845459 | 2.8560335 | 0.0042897 | 0.0390202 |
| CLIC3 | -1.176341 | 0.4120693 | -2.854716 | 0.0043075 | 0.0390948 |
| NEUROD1 | 2.0150786 | 0.7062739 | 2.8531122 | 0.0043293 | 0.0392107 |
| FFAR4 | -1.277676 | 0.4479208 | -2.85246 | 0.0043382 | 0.039264 |
| AL035425.4 | 1.7041239 | 0.5979747 | 2.8498261 | 0.0043743 | 0.0395494 |
| TRPC3 | -1.02481 | 0.3596282 | -2.849637 | 0.0043769 | 0.0395592 |
| GKN2 | -1.320292 | 0.4634322 | -2.848942 | 0.0043865 | 0.0396124 |
| AC012313.6 | 1.021153 | 0.3584542 | 2.8487682 | 0.0043889 | 0.0396124 |
| FOXN1 | 1.3557596 | 0.476039 | 2.848001 | 0.0043995 | 0.0396943 |
| RPLP0P2 | -1.001963 | 0.3518756 | -2.847493 | 0.0044065 | 0.0397302 |
| LRRC9 | 2.0893565 | 0.734403 | 2.8449726 | 0.0044415 | 0.0399656 |
| THBD | -1.054743 | 0.370742 | -2.844952 | 0.0044418 | 0.0399656 |
| LYPD6B | 1.0455567 | 0.3678849 | 2.8420755 | 0.0044821 | 0.0402446 |
| LCN12 | 1.5347806 | 0.5413487 | 2.8351054 | 0.0045811 | 0.0409546 |
| SERPINB3 | 5.6470732 | 1.9918423 | 2.8351005 | 0.0045811 | 0.0409546 |
| AL772337.1 | -6.294519 | 2.2208691 | -2.83426 | 0.0045932 | 0.0410303 |
| LINC02604 | 1.048117 | 0.3699392 | 2.8332141 | 0.0046083 | 0.0411378 |
| C16orf96 | 1.107267 | 0.3909903 | 2.8319552 | 0.0046264 | 0.0412423 |
| HNRNPA1P54 | 1.5525529 | 0.5484234 | 2.8309383 | 0.0046412 | 0.0412901 |
| NCF1 | -1.117702 | 0.3948182 | -2.830929 | 0.0046413 | 0.0412901 |
| PCYT1B | 1.023787 | 0.3618054 | 2.8296622 | 0.0046597 | 0.0414116 |
| GBP4 | -1.016669 | 0.3594973 | -2.828029 | 0.0046836 | 0.0415385 |
| LINC02018 | 1.3479121 | 0.4766098 | 2.8281251 | 0.0046822 | 0.0415385 |
| IGLC6 | 1.6597296 | 0.5868818 | 2.8280472 | 0.0046833 | 0.0415385 |
| RTKN2 | -1.290078 | 0.4563237 | -2.827111 | 0.004697 | 0.0415869 |
| SLC4A1 | -2.127836 | 0.7527253 | -2.826843 | 0.0047009 | 0.0415936 |
| MFSD6L | 1.2487476 | 0.4419106 | 2.8257924 | 0.0047164 | 0.0417081 |
| AC235565.2 | 1.765189 | 0.6246809 | 2.8257451 | 0.0047171 | 0.0417081 |
| TDRD5 | 2.1405889 | 0.7578037 | 2.8247276 | 0.0047321 | 0.0418265 |
| SAA2 | 4.2585054 | 1.5083821 | 2.8232272 | 0.0047543 | 0.0419659 |
| AC105429.1 | -1.004043 | 0.3557037 | -2.822694 | 0.0047622 | 0.0420043 |
| TM4SF18 | 1.0392938 | 0.3684173 | 2.8209694 | 0.0047879 | 0.042147 |
| AP000941.1 | -1.025305 | 0.3635162 | -2.82052 | 0.0047946 | 0.0421646 |
| FCRL5 | 1.6882735 | 0.5986478 | 2.8201448 | 0.0048002 | 0.0421997 |
| GASAL1 | -1.552598 | 0.5507252 | -2.819189 | 0.0048145 | 0.0422828 |
| AL021392.1 | 1.0322981 | 0.3661999 | 2.818947 | 0.0048181 | 0.0423004 |
| AC073862.3 | -1.442736 | 0.5121706 | -2.816906 | 0.0048489 | 0.0425415 |
| ELANE | -1.035205 | 0.3676058 | -2.816074 | 0.0048615 | 0.0426232 |
| IGKV2D-29 | 3.3095928 | 1.1755211 | 2.8154262 | 0.0048713 | 0.0426804 |
| RN7SL8P | -2.040077 | 0.7248034 | -2.814663 | 0.0048828 | 0.0427244 |
| AC007728.2 | -1.19241 | 0.4236245 | -2.814781 | 0.004881 | 0.0427244 |
| SLC2A14 | -1.787279 | 0.6352348 | -2.813572 | 0.0048994 | 0.0427835 |
| CT45A10 | -3.69963 | 1.3150722 | -2.813252 | 0.0049043 | 0.0428117 |
| LINC02363 | 2.3175718 | 0.8238706 | 2.8130288 | 0.0049077 | 0.0428131 |
| PACRG-AS1 | 1.9365202 | 0.6884754 | 2.8127658 | 0.0049117 | 0.0428333 |
| CXCL13 | 3.063957 | 1.0895605 | 2.8121035 | 0.0049219 | 0.0428931 |
| AC233755.1 | 3.0570312 | 1.0871539 | 2.8119581 | 0.0049241 | 0.0428982 |
| AC010980.2 | -1.836736 | 0.6533125 | -2.81142 | 0.0049323 | 0.0429341 |
| IGSF21 | -1.257055 | 0.4472219 | -2.810808 | 0.0049417 | 0.0429657 |
| ADGRE3 | -1.415936 | 0.5039132 | -2.809882 | 0.004956 | 0.0430464 |
| OR51E1 | 2.3529279 | 0.8374134 | 2.8097567 | 0.0049579 | 0.0430488 |
| IGLV8-61 | 2.6222096 | 0.9340943 | 2.8072214 | 0.0049971 | 0.0432738 |
| SLC26A4-AS1 | 3.5424016 | 1.262528 | 2.8058004 | 0.0050192 | 0.0434074 |
| AC020978.4 | 1.2078312 | 0.4306166 | 2.8048877 | 0.0050334 | 0.0435016 |
| SERPINA6 | 4.1348951 | 1.4751018 | 2.8031254 | 0.005061 | 0.0436532 |
| ITGBL1 | 1.1387812 | 0.4064069 | 2.8020713 | 0.0050776 | 0.0437671 |
| TXLNB | 1.2122724 | 0.4328705 | 2.8005429 | 0.0051017 | 0.0439459 |
| SVIL2P | -1.278961 | 0.4568114 | -2.799756 | 0.0051141 | 0.0440094 |
| IL1R2 | -1.727505 | 0.6175421 | -2.797389 | 0.0051518 | 0.0442772 |
| RBM24 | 1.1807295 | 0.4221058 | 2.797236 | 0.0051542 | 0.0442772 |
| IGHV3-66 | 2.2463407 | 0.8030826 | 2.7971476 | 0.0051556 | 0.0442772 |
| AC004556.4 | 1.2260228 | 0.4383402 | 2.7969664 | 0.0051585 | 0.0442772 |
| ABHD17AP4 | -2.86561 | 1.0250521 | -2.795575 | 0.0051807 | 0.0444068 |
| PCSK9 | -1.041349 | 0.3726763 | -2.794247 | 0.0052021 | 0.0445458 |
| MPO | -1.610412 | 0.5763989 | -2.793919 | 0.0052073 | 0.0445616 |
| S1PR5 | -1.326194 | 0.47486 | -2.792811 | 0.0052252 | 0.044615 |
| NAT8L | 1.3965667 | 0.5002507 | 2.7917336 | 0.0052427 | 0.0447169 |
| AC008278.2 | 2.8428303 | 1.019573 | 2.7882557 | 0.0052993 | 0.0450966 |
| CR559946.2 | 2.0911091 | 0.7500123 | 2.7880998 | 0.0053018 | 0.0451035 |
| STAC | -1.011486 | 0.3629153 | -2.787112 | 0.005318 | 0.0451964 |
| ARMH1 | 1.0001322 | 0.3589322 | 2.7864094 | 0.0053296 | 0.0452364 |
| YBX1P4 | -1.862318 | 0.6687922 | -2.784598 | 0.0053594 | 0.0454133 |
| IGLV7-46 | 2.3576126 | 0.8467863 | 2.7841885 | 0.0053662 | 0.0454313 |
| AC079140.6 | 1.0985623 | 0.3946824 | 2.783408 | 0.0053791 | 0.0454938 |
| FAM27B | 1.1769827 | 0.4228494 | 2.7834561 | 0.0053783 | 0.0454938 |
| FAM237B | 2.1338937 | 0.7667216 | 2.7831402 | 0.0053836 | 0.0455166 |
| PPIAP74 | 2.7459552 | 0.9868775 | 2.7824683 | 0.0053947 | 0.0455962 |
| MTTP | 1.3161811 | 0.4735934 | 2.7791372 | 0.0054504 | 0.0459919 |
| GATA2-AS1 | -1.041955 | 0.3749865 | -2.778647 | 0.0054586 | 0.0460167 |
| ETV5-AS1 | -1.868086 | 0.6725313 | -2.777695 | 0.0054746 | 0.0460475 |
| CRABP2 | 1.0370844 | 0.3734306 | 2.7771812 | 0.0054833 | 0.0460864 |
| IGKV5-2 | 3.1866166 | 1.1475021 | 2.7770027 | 0.0054863 | 0.0460864 |
| CCL19 | 1.2675202 | 0.456435 | 2.7770008 | 0.0054863 | 0.0460864 |
| LINC01644 | 1.3964421 | 0.5028519 | 2.7770447 | 0.0054856 | 0.0460864 |
| AC024941.2 | 1.1512194 | 0.4145868 | 2.7767873 | 0.0054899 | 0.0461018 |
| RPL21P7 | 2.944518 | 1.0612264 | 2.7746371 | 0.0055263 | 0.0463629 |
| LINC02135 | 6.0670285 | 2.1875308 | 2.7734597 | 0.0055464 | 0.0464711 |
| CASP12 | -1.020769 | 0.3680829 | -2.773203 | 0.0055508 | 0.0464728 |
| PCARE | -1.536807 | 0.5543397 | -2.77232 | 0.0055658 | 0.0465444 |
| SLC44A5 | 1.5126217 | 0.5456552 | 2.77212 | 0.0055693 | 0.0465581 |
| AC018529.1 | -1.88299 | 0.6795662 | -2.770871 | 0.0055907 | 0.0466771 |
| AL449106.1 | 1.7860079 | 0.6450323 | 2.7688659 | 0.0056252 | 0.0469053 |
| PTHLH | 1.0280106 | 0.3713311 | 2.7684473 | 0.0056324 | 0.046941 |
| C2orf91 | -1.470631 | 0.5314088 | -2.76742 | 0.0056502 | 0.0470536 |
| AGTR2 | 2.2375593 | 0.8086108 | 2.7671647 | 0.0056546 | 0.0470755 |
| DMBX1 | 3.504322 | 1.2668744 | 2.7661163 | 0.0056728 | 0.0471819 |
| AC025539.1 | 2.3602146 | 0.8536179 | 2.7649544 | 0.0056931 | 0.0472899 |
| HAMP | 2.5172995 | 0.9104096 | 2.7650188 | 0.005692 | 0.0472899 |
| AC020658.7 | 2.4678204 | 0.8929797 | 2.7635796 | 0.0057171 | 0.047452 |
| SSTR3 | -1.221799 | 0.4424342 | -2.761537 | 0.005753 | 0.0476661 |
| LHFPL3 | -1.828011 | 0.6622788 | -2.760183 | 0.0057769 | 0.0478337 |
| AC005487.1 | 3.0472436 | 1.1039982 | 2.7601888 | 0.0057768 | 0.0478337 |
| ADAMTS7P3 | -1.389343 | 0.5035841 | -2.758909 | 0.0057995 | 0.0479507 |
| AC127526.2 | 2.8350723 | 1.0277374 | 2.7585572 | 0.0058057 | 0.0479732 |
| CYTL1 | 1.5043454 | 0.5454041 | 2.7582214 | 0.0058117 | 0.0479998 |
| SIRPB1 | -1.158501 | 0.420146 | -2.757379 | 0.0058267 | 0.0480305 |
| NXF3 | -2.158647 | 0.7828807 | -2.757313 | 0.0058279 | 0.0480305 |
| AC004863.1 | 1.8929963 | 0.68693 | 2.7557341 | 0.0058561 | 0.048244 |
| LINC00958 | 1.6473096 | 0.5978985 | 2.7551659 | 0.0058662 | 0.0482973 |
| AC009754.2 | 2.7075153 | 0.9827578 | 2.7550179 | 0.0058689 | 0.0483039 |
| CLEC4M | -3.02594 | 1.0998231 | -2.751297 | 0.005936 | 0.048733 |
| DNAH17-AS1 | -1.156263 | 0.4203022 | -2.751027 | 0.0059409 | 0.0487579 |
| LINC02265 | 2.1419382 | 0.7787081 | 2.7506303 | 0.0059481 | 0.0488015 |
| AP001453.2 | 1.1040607 | 0.4016814 | 2.7485979 | 0.0059851 | 0.0490587 |
| CRLF1 | 1.008237 | 0.3668558 | 2.7483193 | 0.0059902 | 0.0490696 |
| PADI4 | -2.371645 | 0.8632467 | -2.747354 | 0.0060078 | 0.0491679 |
| AP001021.1 | 1.8616423 | 0.6776851 | 2.7470609 | 0.0060132 | 0.0491965 |
| AL356417.3 | 1.4142695 | 0.5151178 | 2.7455266 | 0.0060414 | 0.0493342 |
| VWC2L | 3.0436827 | 1.1097337 | 2.7427145 | 0.0060934 | 0.0496999 |
| BCAR3-AS1 | 1.1075801 | 0.4039036 | 2.7421895 | 0.0061031 | 0.0497137 |
| AC004556.2 | 1.2397513 | 0.4521684 | 2.7417913 | 0.0061105 | 0.0497429 |
| SFTPA1 | -1.046551 | 0.3818236 | -2.740928 | 0.0061266 | 0.049827 |
