# Supplementary material for: Integrated bulk and single-cell RNA-sequencing reveals SPOCK2 as a novel biomarker gene in the development of congenital pulmonary airway malformation
Source: Respir Res. 2023 May 10;24:127. doi: 10.1186/s12931-023-02436-z (PMC10170809; doi:10.1186/s12931-023-02436-z)
Supplement: Supplementary file 3 — Additional file3: table S3 GO enrichment analysis (biological process, BP) of up-regulated and down-regulated genes [file 12931_2023_2436_MOESM3_ESM.docx]

**Table S3a GO enrichment analysis (biological process, BP) of up-regulated genes**

| **ID** | **Description** | **Gene Ratio** | **Bg Ratio** | ***P*-value** | **FDR** | **Gene ID** |
| --- | --- | --- | --- | --- | --- | --- |
| GO:0044782 | cilium organization | 110/947 | 384/18800 | 4.15E-53 | 1.80E-49 | CCNO/NME5/CFAP61/DRC1/CCDC65/CFAP53/UBXN10/RSPH1/DAW1/RSPH4A/TEKT1/CFAP126/RSPH9/DRC7/CCDC113/DNAI2/IQCG/CFAP43/LCA5L/CFAP52/DNAH7/DNAH5/DNAI1/TEKT4/FOXJ1/MNS1/KIF19/SPEF1/PIFO/TEKT3/CFAP58/TTC29/TEKT2/DNAAF3/SPAG6/RP1/TMEM231/CFAP65/ENO4/DNAJB13/CCDC39/ZMYND10/KIF24/CFAP73/CFAP100/CCDC96/CFAP157/RPGRIP1L/DNAH2/CCDC103/HYDIN/CFAP221/SPAG16/DNAAF1/DNAL1/CFAP47/CFAP74/BBOF1/MCIDAS/SPAG17/B9D1/CFAP54/LRGUK/MEIG1/CCDC13/DNAAF4/SPAG1/MAPK15/DZIP1L/CFAP70/CEP126/CFAP46/TTC26/IQUB/CFAP69/MAK/IFT81/FAM161A/TMEM67/RFX3/ROPN1B/TTC30B/DYNC2H1/CCDC40/SPEF2/CEP19/CFAP206/CEP83/CFAP161/TRAF3IP1/TTC21A/IFT46/TMEM107/TTC30A/ROPN1/IFT172/CFAP298/TCTN1/CC2D2A/ARMC2/RABL2B/CFAP44/CATIP/FUZ/IFT27/TMEM17/LCA5/TCTN2/RFX2/DNAH1 |
| GO:0003341 | cilium movement | 75/947 | 187/18800 | 3.92E-48 | 8.49E-45 | ENKUR/NME5/TCTE1/CFAP61/DRC1/CCDC65/CFAP53/DAW1/RSPH4A/TEKT1/RSPH9/DRC7/ZBBX/DNAI2/IQCG/CATSPERD/CFAP43/CFAP52/DNAH7/DNAH5/DNAI1/TEKT4/GAS2L2/MNS1/CFAP45/NEK10/SPEF1/TEKT3/CFAP58/SPA17/TTC29/TEKT2/ROPN1L/SPAG6/CFAP65/ENO4/DNAH9/DNAH11/CCDC39/ZMYND10/CFAP73/CFAP100/TTLL9/CFAP157/CCDC103/HYDIN/CFAP221/SPAG16/DNAAF1/CFAP47/SPAG17/CFAP54/TTLL6/MEIG1/DNAAF4/CFAP70/CFAP46/CFAP69/RFX3/ROPN1B/DPCD/SORD/CCDC40/SPEF2/CFAP206/TTC21A/ROPN1/CFAP298/ARMC2/CATSPERE/CFAP44/EPPIN/TACR1/CABYR/DNAH1 |
| GO:0007018 | microtubule-based movement | 104/947 | 395/18800 | 2.16E-46 | 3.12E-43 | ENKUR/NME5/TCTE1/CFAP61/DRC1/CCDC65/CFAP53/DAW1/RSPH4A/TEKT1/RSPH9/DRC7/ZBBX/DNAI2/IQCG/CATSPERD/DNAH6/CFAP43/LCA5L/CFAP52/DNAH7/DNAH5/DNAI1/TEKT4/GAS2L2/MNS1/KIF19/CFAP45/NEK10/SPEF1/TEKT3/CFAP58/SPA17/TTC29/TEKT2/ROPN1L/SPAG6/DYNLRB2/CFAP65/ENO4/DNAH9/DNAH11/CCDC39/ZMYND10/KIF24/DNAH12/CFAP73/CFAP100/TTLL9/KIF6/CFAP157/DNAH2/CCDC103/HYDIN/CFAP221/SPAG16/DNAAF1/CFAP47/DNAH10/SPAG17/MAP1A/CFAP54/TTLL6/MEIG1/DNAAF4/FMN2/DNAH3/KIF21A/CFAP70/KIF1A/CFAP46/TTC26/CFAP69/MAK/IFT81/RFX3/ROPN1B/DPCD/TTC30B/DYNC2H1/AGBL4/SORD/CCDC40/SPEF2/CFAP206/CNIH2/TRAF3IP1/TTC21A/IFT46/TTC30A/ROPN1/IFT172/CFAP298/ARMC2/RABL2B/CATSPERE/CFAP44/IFT27/LCA5/AP3B2/EPPIN/TACR1/CABYR/DNAH1 |
| GO:0035082 | axoneme assembly | 51/947 | 87/18800 | 1.14E-43 | 1.24E-40 | DRC1/CCDC65/RSPH1/DAW1/RSPH4A/RSPH9/DRC7/DNAI2/IQCG/CFAP43/DNAH7/DNAH5/DNAI1/FOXJ1/MNS1/SPEF1/CFAP58/TEKT2/DNAAF3/SPAG6/RP1/CFAP65/DNAJB13/CCDC39/ZMYND10/CFAP73/CFAP100/CFAP157/DNAH2/CCDC103/HYDIN/SPAG16/DNAAF1/DNAL1/CFAP47/CFAP74/SPAG17/LRGUK/MEIG1/DNAAF4/SPAG1/CFAP46/TTC26/CFAP69/CCDC40/SPEF2/CFAP206/CC2D2A/ARMC2/CFAP44/DNAH1 |
| GO:0060271 | cilium assembly | 95/947 | 355/18800 | 4.84E-43 | 4.19E-40 | CCNO/NME5/DRC1/CCDC65/CFAP53/UBXN10/RSPH1/DAW1/RSPH4A/TEKT1/RSPH9/DRC7/CCDC113/DNAI2/IQCG/CFAP43/CFAP52/DNAH7/DNAH5/DNAI1/TEKT4/FOXJ1/MNS1/SPEF1/TEKT3/CFAP58/TEKT2/DNAAF3/SPAG6/RP1/TMEM231/CFAP65/DNAJB13/CCDC39/ZMYND10/KIF24/CFAP73/CFAP100/CCDC96/CFAP157/RPGRIP1L/DNAH2/CCDC103/HYDIN/CFAP221/SPAG16/DNAAF1/DNAL1/CFAP47/CFAP74/BBOF1/MCIDAS/SPAG17/B9D1/CFAP54/LRGUK/MEIG1/CCDC13/DNAAF4/SPAG1/MAPK15/DZIP1L/CFAP70/CEP126/CFAP46/TTC26/IQUB/CFAP69/MAK/IFT81/FAM161A/TMEM67/RFX3/DYNC2H1/CCDC40/SPEF2/CEP19/CFAP206/CEP83/CFAP161/TRAF3IP1/IFT46/TMEM107/IFT172/CFAP298/TCTN1/CC2D2A/ARMC2/RABL2B/CFAP44/FUZ/TMEM17/TCTN2/RFX2/DNAH1 |
| GO:0001578 | microtubule bundle formation | 54/947 | 118/18800 | 1.42E-38 | 1.03E-35 | DRC1/CCDC65/RSPH1/DAW1/RSPH4A/RSPH9/DRC7/DNAI2/IQCG/CFAP43/DNAH7/DNAH5/DNAI1/GAS2L2/FOXJ1/MNS1/SPEF1/CFAP58/TEKT2/DNAAF3/SPAG6/RP1/CFAP65/DNAJB13/CCDC39/ZMYND10/CFAP73/CFAP100/CFAP157/DNAH2/CCDC103/HYDIN/SPAG16/DNAAF1/DNAL1/CFAP47/CFAP74/SPAG17/LRGUK/TTLL6/MEIG1/DNAAF4/SPAG1/CFAP46/TTC26/CFAP69/CCDC40/SPEF2/CFAP206/CC2D2A/TPPP3/ARMC2/CFAP44/DNAH1 |
| GO:0001539 | cilium or flagellum-dependent cell motility | 57/947 | 151/18800 | 4.49E-35 | 2.43E-32 | ENKUR/TCTE1/DRC1/CCDC65/RSPH4A/TEKT1/RSPH9/DRC7/IQCG/CATSPERD/DNAH6/CFAP43/CFAP52/DNAH7/DNAH5/DNAI1/TEKT4/GAS2L2/MNS1/CFAP45/TEKT3/CFAP58/TEKT2/EFHC2/ROPN1L/SPAG6/CFAP65/ENO4/DNAH11/CCDC39/TTLL9/CFAP157/DNAH2/SPAG16/CFAP47/CFAP54/TTLL6/MEIG1/DNAH3/CFAP46/CFAP69/RFX3/ROPN1B/EFHC1/DPCD/SORD/CCDC40/SPEF2/CFAP206/TTC21A/ROPN1/ARMC2/CATSPERE/CFAP44/EPPIN/TACR1/DNAH1 |
| GO:0060285 | cilium-dependent cell motility | 57/947 | 151/18800 | 4.49E-35 | 2.43E-32 | ENKUR/TCTE1/DRC1/CCDC65/RSPH4A/TEKT1/RSPH9/DRC7/IQCG/CATSPERD/DNAH6/CFAP43/CFAP52/DNAH7/DNAH5/DNAI1/TEKT4/GAS2L2/MNS1/CFAP45/TEKT3/CFAP58/TEKT2/EFHC2/ROPN1L/SPAG6/CFAP65/ENO4/DNAH11/CCDC39/TTLL9/CFAP157/DNAH2/SPAG16/CFAP47/CFAP54/TTLL6/MEIG1/DNAH3/CFAP46/CFAP69/RFX3/ROPN1B/EFHC1/DPCD/SORD/CCDC40/SPEF2/CFAP206/TTC21A/ROPN1/ARMC2/CATSPERE/CFAP44/EPPIN/TACR1/DNAH1 |
| GO:0060294 | cilium movement involved in cell motility | 48/947 | 138/18800 | 7.88E-28 | 3.79E-25 | ENKUR/TCTE1/RSPH4A/TEKT1/RSPH9/DRC7/IQCG/CATSPERD/CFAP43/CFAP52/DNAH5/DNAI1/TEKT4/GAS2L2/MNS1/CFAP45/TEKT3/CFAP58/TEKT2/ROPN1L/SPAG6/CFAP65/ENO4/DNAH11/CCDC39/TTLL9/CFAP157/SPAG16/CFAP47/CFAP54/TTLL6/MEIG1/CFAP46/CFAP69/ROPN1B/DPCD/SORD/CCDC40/SPEF2/CFAP206/TTC21A/ROPN1/ARMC2/CATSPERE/CFAP44/EPPIN/TACR1/DNAH1 |
| GO:0003351 | epithelial cilium movement involved in extracellular fluid movement | 27/947 | 41/18800 | 1.14E-25 | 4.94E-23 | NME5/CFAP53/DAW1/CFAP43/DNAH5/DNAI1/CFAP45/NEK10/SPA17/ROPN1L/SPAG6/DNAH9/DNAH11/CCDC39/CCDC103/CFAP221/SPAG16/DNAAF1/SPAG17/CFAP54/DNAAF4/RFX3/DPCD/CCDC40/SPEF2/CABYR/DNAH1 |
| GO:0006958 | complement activation, classical pathway | 40/947 | 108/18800 | 1.35E-24 | 5.32E-22 | C6/IGHV1-18/IGHV3-23/IGHV2-26/IGHV2-5/IGLC2/IGHV3-15/IGHV4-39/IGLL5/IGHG1/IGHG4/IGHV3-48/IGHG3/IGHV3-33/IGHV4-61/IGKC/IGHV3-11/IGHV4-34/IGHA2/IGHV3-43/IGLC3/IGHV3-74/IGHV1-3/IGHV6-1/IGHV1-69/IGHV3-13/IGHV3-21/IGHV5-51/IGHA1/IGHV3-64/IGHG2/IGHV4-59/SUSD4/IGHV3-30/IGHV3-7/IGHV1OR15-1/IGLC1/IGHV3-49/IGLC6/IGHV3-66 |
| GO:0006858 | extracellular transport | 27/947 | 44/18800 | 1.92E-24 | 6.93E-22 | NME5/CFAP53/DAW1/CFAP43/DNAH5/DNAI1/CFAP45/NEK10/SPA17/ROPN1L/SPAG6/DNAH9/DNAH11/CCDC39/CCDC103/CFAP221/SPAG16/DNAAF1/SPAG17/CFAP54/DNAAF4/RFX3/DPCD/CCDC40/SPEF2/CABYR/DNAH1 |
| GO:0006910 | phagocytosis, recognition | 38/947 | 102/18800 | 1.55E-23 | 5.14E-21 | IGHV1-18/IGHV3-23/IGHV2-26/IGHV2-5/IGLC2/IGHV3-15/IGHV4-39/IGLL5/IGHG1/IGHG4/IGHV3-48/IGHG3/IGHV3-33/IGHV4-61/IGKC/IGHV3-11/IGHV4-34/IGHA2/IGHV3-43/IGLC3/IGHV3-74/IGHV1-3/IGHV6-1/IGHV1-69/IGHV3-13/IGHV3-21/IGHV5-51/IGHA1/IGHV3-64/IGHG2/IGHV4-59/IGHV3-30/IGHV3-7/IGHV1OR15-1/IGLC1/IGHV3-49/IGLC6/IGHV3-66 |
| GO:0002455 | humoral immune response mediated by circulating immunoglobulin | 41/947 | 121/18800 | 1.89E-23 | 5.85E-21 | C6/FOXJ1/IGHV1-18/IGHV3-23/IGHV2-26/IGHV2-5/IGLC2/IGHV3-15/IGHV4-39/IGLL5/IGHG1/IGHG4/IGHV3-48/IGHG3/IGHV3-33/IGHV4-61/IGKC/IGHV3-11/IGHV4-34/IGHA2/IGHV3-43/IGLC3/IGHV3-74/IGHV1-3/IGHV6-1/IGHV1-69/IGHV3-13/IGHV3-21/IGHV5-51/IGHA1/IGHV3-64/IGHG2/IGHV4-59/SUSD4/IGHV3-30/IGHV3-7/IGHV1OR15-1/IGLC1/IGHV3-49/IGLC6/IGHV3-66 |
| GO:0030317 | flagellated sperm motility | 40/947 | 122/18800 | 2.67E-22 | 7.22E-20 | ENKUR/TCTE1/DRC7/IQCG/CATSPERD/CFAP43/CFAP52/DNAH5/DNAI1/MNS1/CFAP45/TEKT3/CFAP58/TEKT2/ROPN1L/SPAG6/CFAP65/ENO4/DNAH11/CCDC39/TTLL9/CFAP157/SPAG16/CFAP47/MEIG1/CFAP69/ROPN1B/DPCD/SORD/CCDC40/SPEF2/CFAP206/TTC21A/ROPN1/ARMC2/CATSPERE/CFAP44/EPPIN/TACR1/DNAH1 |
| GO:0097722 | sperm motility | 40/947 | 122/18800 | 2.67E-22 | 7.22E-20 | ENKUR/TCTE1/DRC7/IQCG/CATSPERD/CFAP43/CFAP52/DNAH5/DNAI1/MNS1/CFAP45/TEKT3/CFAP58/TEKT2/ROPN1L/SPAG6/CFAP65/ENO4/DNAH11/CCDC39/TTLL9/CFAP157/SPAG16/CFAP47/MEIG1/CFAP69/ROPN1B/DPCD/SORD/CCDC40/SPEF2/CFAP206/TTC21A/ROPN1/ARMC2/CATSPERE/CFAP44/EPPIN/TACR1/DNAH1 |
| GO:0044458 | motile cilium assembly | 28/947 | 57/18800 | 1.15E-21 | 2.94E-19 | RSPH9/DRC7/IQCG/CFAP43/FOXJ1/MNS1/CFAP58/DNAAF3/SPAG6/CFAP65/CCDC39/ZMYND10/CFAP157/CFAP221/SPAG16/DNAAF1/CFAP47/BBOF1/MCIDAS/MEIG1/CFAP69/CCDC40/SPEF2/CFAP206/CC2D2A/ARMC2/CFAP44/DNAH1 |
| GO:0006956 | complement activation | 40/947 | 131/18800 | 5.21E-21 | 1.25E-18 | C6/IGHV1-18/IGHV3-23/IGHV2-26/IGHV2-5/IGLC2/IGHV3-15/IGHV4-39/IGLL5/IGHG1/IGHG4/IGHV3-48/IGHG3/IGHV3-33/IGHV4-61/IGKC/IGHV3-11/IGHV4-34/IGHA2/IGHV3-43/IGLC3/IGHV3-74/IGHV1-3/IGHV6-1/IGHV1-69/IGHV3-13/IGHV3-21/IGHV5-51/IGHA1/IGHV3-64/IGHG2/IGHV4-59/SUSD4/IGHV3-30/IGHV3-7/IGHV1OR15-1/IGLC1/IGHV3-49/IGLC6/IGHV3-66 |
| GO:0006911 | phagocytosis, engulfment | 39/947 | 128/18800 | 1.77E-20 | 4.02E-18 | IGHV1-18/IGHV3-23/IGHV2-26/IGHV2-5/IGLC2/IGHV3-15/IGHV4-39/IGLL5/IGHG1/IGHG4/IGHV3-48/IGHG3/IGHV3-33/IGHV4-61/IGKC/IGHV3-11/IGHV4-34/IGHA2/ALOX15/IGHV3-43/IGLC3/IGHV3-74/IGHV1-3/IGHV6-1/IGHV1-69/IGHV3-13/IGHV3-21/IGHV5-51/IGHA1/IGHV3-64/IGHG2/IGHV4-59/IGHV3-30/IGHV3-7/IGHV1OR15-1/IGLC1/IGHV3-49/IGLC6/IGHV3-66 |
| GO:0099024 | plasma membrane invagination | 40/947 | 137/18800 | 3.24E-20 | 7.01E-18 | IGHV1-18/IGHV3-23/IGHV2-26/IGHV2-5/IGLC2/IGHV3-15/IGHV4-39/IGLL5/IGHG1/IGHG4/IGHV3-48/IGHG3/IGHV3-33/IGHV4-61/IGKC/IGHV3-11/IGHV4-34/IGHA2/ALOX15/IGHV3-43/IGLC3/IGHV3-74/IGHV1-3/IGHV6-1/IGHV1-69/IGHV3-13/IGHV3-21/IGHV5-51/IGHA1/IGHV3-64/IGHG2/IGHV4-59/IGHV3-30/IGHV3-7/SPIRE2/IGHV1OR15-1/IGLC1/IGHV3-49/IGLC6/IGHV3-66 |
| GO:0010324 | membrane invagination | 40/947 | 144/18800 | 2.39E-19 | 4.92E-17 | IGHV1-18/IGHV3-23/IGHV2-26/IGHV2-5/IGLC2/IGHV3-15/IGHV4-39/IGLL5/IGHG1/IGHG4/IGHV3-48/IGHG3/IGHV3-33/IGHV4-61/IGKC/IGHV3-11/IGHV4-34/IGHA2/ALOX15/IGHV3-43/IGLC3/IGHV3-74/IGHV1-3/IGHV6-1/IGHV1-69/IGHV3-13/IGHV3-21/IGHV5-51/IGHA1/IGHV3-64/IGHG2/IGHV4-59/IGHV3-30/IGHV3-7/SPIRE2/IGHV1OR15-1/IGLC1/IGHV3-49/IGLC6/IGHV3-66 |
| GO:0050853 | B cell receptor signaling pathway | 38/947 | 131/18800 | 3.67E-19 | 7.21E-17 | IGHV1-18/IGHV3-23/IGHV2-26/IGHV2-5/IGLC2/IGHV3-15/IGHV4-39/IGLL5/IGHG1/IGHG4/IGHV3-48/IGHG3/IGHV3-33/IGHV4-61/IGKC/IGHV3-11/IGHV4-34/IGHA2/IGHV3-43/IGLC3/IGHV3-74/IGHV1-3/IGHV6-1/IGHV1-69/IGHV3-13/IGHV3-21/IGHV5-51/IGHA1/IGHV3-64/IGHG2/IGHV4-59/IGHV3-30/IGHV3-7/IGHV1OR15-1/IGLC1/IGHV3-49/IGLC6/IGHV3-66 |
| GO:0099111 | microtubule-based transport | 46/947 | 198/18800 | 1.24E-18 | 2.34E-16 | NME5/CFAP53/DAW1/CFAP43/LCA5L/DNAH5/DNAI1/CFAP45/NEK10/SPA17/ROPN1L/SPAG6/DNAH9/DNAH11/CCDC39/CCDC103/CFAP221/SPAG16/DNAAF1/SPAG17/MAP1A/CFAP54/DNAAF4/KIF1A/TTC26/MAK/IFT81/RFX3/DPCD/TTC30B/DYNC2H1/AGBL4/CCDC40/SPEF2/CNIH2/TRAF3IP1/TTC21A/IFT46/TTC30A/IFT172/RABL2B/IFT27/LCA5/AP3B2/CABYR/DNAH1 |
| GO:0070286 | axonemal dynein complex assembly | 21/947 | 38/18800 | 5.72E-18 | 1.03E-15 | DRC1/CCDC65/DAW1/DNAI2/DNAH7/DNAH5/DNAI1/TEKT2/DNAAF3/CCDC39/ZMYND10/CFAP73/CFAP100/DNAH2/CCDC103/DNAAF1/DNAL1/DNAAF4/SPAG1/CCDC40/DNAH1 |
| GO:0050871 | positive regulation of B cell activation | 39/947 | 152/18800 | 1.39E-17 | 2.41E-15 | IGHV1-18/IGHV3-23/IGHV2-26/IGHV2-5/IGLC2/IGHV3-15/IGHV4-39/IGLL5/IGHG1/IGHG4/IGHV3-48/IGHG3/IGHV3-33/IGHV4-61/IGKC/IGHV3-11/IGHV4-34/IL7/IGHA2/IGHV3-43/IGLC3/IGHV3-74/IGHV1-3/IGHV6-1/IGHV1-69/IGHV3-13/IGHV3-21/IGHV5-51/IGHA1/IGHV3-64/IGHG2/IGHV4-59/IGHV3-30/IGHV3-7/IGHV1OR15-1/IGLC1/IGHV3-49/IGLC6/IGHV3-66 |
| GO:0007288 | sperm axoneme assembly | 17/947 | 28/18800 | 9.57E-16 | 1.59E-13 | DRC7/IQCG/CFAP43/MNS1/CFAP58/SPAG6/CFAP65/CFAP157/SPAG16/CFAP47/MEIG1/CFAP69/SPEF2/CFAP206/ARMC2/CFAP44/DNAH1 |
| GO:0050864 | regulation of B cell activation | 42/947 | 200/18800 | 1.88E-15 | 3.02E-13 | FOXJ1/IGHV1-18/IGHV3-23/IGHV2-26/IGHV2-5/IGLC2/IGHV3-15/IGHV4-39/IGLL5/IGHG1/IGHG4/HMGB3/IGHV3-48/IGHG3/IGHV3-33/IGHV4-61/IGKC/IGHV3-11/IGHV4-34/IL7/IGHA2/IGHV3-43/IGLC3/IGHV3-74/IGHV1-3/IGHV6-1/IGHV1-69/IGHV3-13/IGHV3-21/IGHV5-51/IGHA1/IGHV3-64/IGHG2/IGHV4-59/IGHV3-30/IGHV3-7/IGHV1OR15-1/MZB1/IGLC1/IGHV3-49/IGLC6/IGHV3-66 |
| GO:0008037 | cell recognition | 45/947 | 228/18800 | 2.13E-15 | 3.29E-13 | SPA17/CLGN/IGHV1-18/IGSF9/IGHV3-23/IGHV2-26/IGHV2-5/IGLC2/IGHV3-15/IGHV4-39/IGLL5/IGHG1/IGHG4/IGHV3-48/VSTM2L/IGHG3/IGHV3-33/IGHV4-61/IGKC/IGHV3-11/IGHV4-34/IGHA2/IGHV3-43/IGLC3/IGHV3-74/IGHV1-3/IGHV6-1/IGHV1-69/IGHV3-13/IGHV3-21/IGHV5-51/IGHA1/EFNB3/IGHV3-64/PCSK4/IGHG2/IGHV4-59/IGHV3-30/IGHV3-7/IGHV1OR15-1/IGLC1/IGHV3-49/IGLC6/IGHV3-66/CCL19 |
| GO:0048515 | spermatid differentiation | 39/947 | 183/18800 | 1.15E-14 | 1.72E-12 | NME5/RSPH1/PACRG/DRC7/IQCG/CATSPERD/CFAP43/MNS1/CFAP58/ROPN1L/SPAG6/TRIP13/CFAP65/CFAP157/SPAG16/CFAP47/DPY19L2P2/MEIG1/TTC26/DPY19L2P1/CFAP69/ROPN1B/NPHP1/DPY19L2/STRBP/SPEF2/CFAP206/TTC21A/ROPN1/OCA2/ARMC2/PCSK4/CATSPERE/CFAP44/SPINK1/RFX2/CABYR/DNAH1/TDRD5 |
| GO:0007286 | spermatid development | 38/947 | 177/18800 | 1.97E-14 | 2.83E-12 | NME5/RSPH1/PACRG/DRC7/IQCG/CATSPERD/CFAP43/MNS1/CFAP58/ROPN1L/SPAG6/TRIP13/CFAP65/CFAP157/SPAG16/CFAP47/DPY19L2P2/MEIG1/TTC26/DPY19L2P1/CFAP69/ROPN1B/DPY19L2/STRBP/SPEF2/CFAP206/TTC21A/ROPN1/OCA2/ARMC2/PCSK4/CATSPERE/CFAP44/SPINK1/RFX2/CABYR/DNAH1/TDRD5 |
| GO:0120316 | sperm flagellum assembly | 17/947 | 32/18800 | 2.08E-14 | 2.91E-12 | DRC7/IQCG/CFAP43/MNS1/CFAP58/SPAG6/CFAP65/CFAP157/SPAG16/CFAP47/MEIG1/CFAP69/SPEF2/CFAP206/ARMC2/CFAP44/DNAH1 |
| GO:0016064 | immunoglobulin mediated immune response | 42/947 | 216/18800 | 3.18E-14 | 4.30E-12 | C6/FOXJ1/IGHV1-18/IGHV3-23/IGHV2-26/IGHV2-5/IGLC2/IGHV3-15/IGHV4-39/IGLL5/IGHG1/IGHG4/IGHV3-48/IGHG3/IGHV3-33/IGHV4-61/IGKC/IGHV3-11/IGHV4-34/IGHA2/IGHV3-43/IGLC3/IGHV3-74/IGHV1-3/IGHV6-1/IGHV1-69/IGHV3-13/IGHV3-21/IGHV5-51/IGHA1/IL13RA2/IGHV3-64/IGHG2/IGHV4-59/SUSD4/IGHV3-30/IGHV3-7/IGHV1OR15-1/IGLC1/IGHV3-49/IGLC6/IGHV3-66 |
| GO:0002377 | immunoglobulin production | 42/947 | 218/18800 | 4.43E-14 | 5.81E-12 | IGKV2-28/IGKV3-20/IGLV3-19/IGKV3-15/IGLV6-57/IGKV1D-33/IGLV1-40/IGLV3-21/IGKV2D-28/IGKV1-5/IGLV3-1/IGKC/IGLV2-11/IGKV1-16/IGKV2-30/IGKV3D-15/IGLV2-18/IGLV2-23/IGKV1D-39/IGLV1-47/IGLV1-44/IGKV1-17/IGLV1-51/IGKV2-24/IL13RA2/IGLV3-25/IGKV2D-30/IGLV3-9/IGKV4-1/IGLV5-45/IGLV3-10/IGKV3D-11/IGKV3D-20/IGKV1-12/MZB1/IGLV2-14/IGLV4-69/IGKV1-39/IGKV2D-29/IGLV8-61/IGLV7-46/IGKV5-2 |
| GO:0019724 | B cell mediated immunity | 42/947 | 219/18800 | 5.22E-14 | 6.64E-12 | C6/FOXJ1/IGHV1-18/IGHV3-23/IGHV2-26/IGHV2-5/IGLC2/IGHV3-15/IGHV4-39/IGLL5/IGHG1/IGHG4/IGHV3-48/IGHG3/IGHV3-33/IGHV4-61/IGKC/IGHV3-11/IGHV4-34/IGHA2/IGHV3-43/IGLC3/IGHV3-74/IGHV1-3/IGHV6-1/IGHV1-69/IGHV3-13/IGHV3-21/IGHV5-51/IGHA1/IL13RA2/IGHV3-64/IGHG2/IGHV4-59/SUSD4/IGHV3-30/IGHV3-7/IGHV1OR15-1/IGLC1/IGHV3-49/IGLC6/IGHV3-66 |
| GO:0006959 | humoral immune response | 51/947 | 317/18800 | 1.56E-13 | 1.93E-11 | C6/FOXJ1/IGHV1-18/WFDC2/IGHV3-23/IGKV3-20/IGHV2-26/IGHV2-5/POU2AF1/IGLC2/IGHV3-15/IGHV4-39/IGLL5/IGHG1/IGHG4/LTF/IGHV3-48/IGHG3/IGHV3-33/IGHV4-61/IGKC/IGHV3-11/IGHV4-34/IL7/IGHA2/IGHV3-43/IGLC3/IGHV3-74/IGHV1-3/IGHV6-1/IGHV1-69/IGHV3-13/JCHAIN/IGHV3-21/IGHV5-51/IGHA1/BPIFA1/IGHV3-64/IGHG2/IGHV4-59/SUSD4/IGHV3-30/IGHV3-7/IGHV1OR15-1/IGLC1/PRSS2/KRT6A/IGHV3-49/IGLC6/CXCL13/IGHV3-66 |
| GO:0036159 | inner dynein arm assembly | 12/947 | 17/18800 | 1.22E-12 | 1.47E-10 | DNAH7/TEKT2/CCDC39/ZMYND10/CFAP73/CFAP100/DNAH2/CCDC103/DNAAF1/DNAAF4/CCDC40/DNAH1 |
| GO:0042742 | defense response to bacterium | 53/947 | 364/18800 | 2.99E-12 | 3.49E-10 | IGHV1-18/WFDC2/IGHV3-23/IGKV3-20/IGHV2-26/IGHV2-5/LCN2/IGLC2/IGHV3-15/IGHV4-39/IGLL5/IGHG1/IGHG4/LTF/IGHV3-48/IGHG3/IGHV3-33/IGHV4-61/IGKC/IGHV3-11/IGHV4-34/IGHA2/IGHV3-43/IGLC3/IGHV3-74/IL12A/IGHV1-3/CHGA/IGHV6-1/IGHV1-69/IGHV3-13/JCHAIN/IGHV3-21/IGHV5-51/IL22RA1/GBP6/IGHA1/BPIFA1/IGHV3-64/IGHG2/IGHV4-59/IGHV3-30/IGHV3-7/EPPIN/IGHV1OR15-1/GSDMC/IGLC1/KRT6A/IGHV3-49/IGLC6/CXCL13/IGHV3-66/HAMP |
| GO:0003352 | regulation of cilium movement | 16/947 | 39/18800 | 1.92E-11 | 2.18E-09 | DRC1/CCDC65/RSPH4A/CFAP43/GAS2L2/CFAP45/DNAH11/CCDC39/DNAAF1/TTLL6/CFAP69/CCDC40/CFAP206/CFAP298/EPPIN/TACR1 |
| GO:0050851 | antigen receptor-mediated signaling pathway | 40/947 | 244/18800 | 3.70E-11 | 4.11E-09 | VTCN1/HHLA2/IGHV1-18/IGHV3-23/IGHV2-26/IGHV2-5/IGLC2/IGHV3-15/IGHV4-39/IGLL5/IGHG1/IGHG4/IGHV3-48/IGHG3/IGHV3-33/IGHV4-61/IGKC/IGHV3-11/IGHV4-34/IGHA2/IGHV3-43/IGLC3/IGHV3-74/IGHV1-3/IGHV6-1/IGHV1-69/IGHV3-13/IGHV3-21/IGHV5-51/IGHA1/IGHV3-64/IGHG2/IGHV4-59/IGHV3-30/IGHV3-7/IGHV1OR15-1/IGLC1/IGHV3-49/IGLC6/IGHV3-66 |
| GO:0007281 | germ cell development | 45/947 | 299/18800 | 4.37E-11 | 4.72E-09 | NME5/RSPH1/PACRG/DRC7/BMPR1B/INHBB/IQCG/CATSPERD/CFAP43/MNS1/CFAP58/ROPN1L/SPAG6/TRIP13/CFAP65/CFAP157/LGR5/SPAG16/CFAP47/DPY19L2P2/MEIG1/FMN2/TTC26/DPY19L2P1/CFAP69/ROPN1B/DPY19L2/STRBP/SPEF2/CFAP206/TTC21A/WNT4/ROPN1/OCA2/ARMC2/PCSK4/CATSPERE/NPM2/CFAP44/DMRTA1/SPINK1/RFX2/CABYR/DNAH1/TDRD5 |
| GO:0060632 | regulation of microtubule-based movement | 17/947 | 54/18800 | 6.07E-10 | 6.41E-08 | DRC1/CCDC65/RSPH4A/CFAP43/GAS2L2/CFAP45/DNAH11/CCDC39/DNAAF1/TTLL6/CFAP69/CCDC40/CFAP206/CNIH2/CFAP298/EPPIN/TACR1 |
| GO:0007368 | determination of left/right symmetry | 26/947 | 128/18800 | 9.35E-10 | 9.63E-08 | ENKUR/DRC1/CFAP53/DAW1/DNAI2/CFAP52/DNAH5/DNAI1/FOXJ1/CFAP45/CFC1/DNAH11/CCDC39/RPGRIP1L/CCDC103/DNAAF1/DNAAF4/RFX3/DPCD/DYNC2H1/CCDC40/CFC1B/TMEM107/IFT172/CC2D2A/FOXN4 |
| GO:0009855 | determination of bilateral symmetry | 27/947 | 138/18800 | 1.07E-09 | 1.08E-07 | ENKUR/DRC1/CFAP53/DAW1/DNAI2/CFAP52/DNAH5/DNAI1/FOXJ1/CFAP45/CFC1/DNAH11/CCDC39/RPGRIP1L/CCDC103/DNAAF1/DNAAF4/RFX3/DPCD/DYNC2H1/CCDC40/CFC1B/TMEM107/IFT172/CC2D2A/GREM1/FOXN4 |
| GO:0009799 | specification of symmetry | 27/947 | 139/18800 | 1.27E-09 | 1.25E-07 | ENKUR/DRC1/CFAP53/DAW1/DNAI2/CFAP52/DNAH5/DNAI1/FOXJ1/CFAP45/CFC1/DNAH11/CCDC39/RPGRIP1L/CCDC103/DNAAF1/DNAAF4/RFX3/DPCD/DYNC2H1/CCDC40/CFC1B/TMEM107/IFT172/CC2D2A/GREM1/FOXN4 |
| GO:0051251 | positive regulation of lymphocyte activation | 48/947 | 371/18800 | 1.92E-09 | 1.84E-07 | VTCN1/CD24/HHLA2/IGHV1-18/IGHV3-23/MYB/IGHV2-26/IGHV2-5/IGLC2/IGHV3-15/IGHV4-39/IGLL5/IGHG1/IGHG4/IGHV3-48/IGHG3/IGHV3-33/IGHV4-61/IGKC/IGHV3-11/IGHV4-34/IL7/IGHA2/IGHV3-43/IGLC3/IGHV3-74/IL12A/IGHV1-3/IGHV6-1/IGHV1-69/IGHV3-13/IGHV3-21/IGHV5-51/IGHA1/EFNB3/IGHV3-64/IGHG2/IGHV4-59/IGHV3-30/IGHV3-7/IGHV1OR15-1/TACR1/IGLC1/TNFSF11/IGHV3-49/IGLC6/IGHV3-66/CCL19 |
| GO:0002440 | production of molecular mediator of immune response | 43/947 | 312/18800 | 1.96E-09 | 1.84E-07 | IGKV2-28/IGKV3-20/IGLV3-19/IGKV3-15/IGLV6-57/IGKV1D-33/IGLV1-40/IGLV3-21/IGKV2D-28/IGKV1-5/IGLV3-1/IGKC/IGLV2-11/IGKV1-16/IGKV2-30/IGKV3D-15/IGLV2-18/IGLV2-23/IGKV1D-39/INAVA/IGLV1-47/IGLV1-44/IGKV1-17/IGLV1-51/IGKV2-24/IL13RA2/IGLV3-25/IGKV2D-30/IGLV3-9/IGKV4-1/IGLV5-45/IGLV3-10/IGKV3D-11/IGKV3D-20/IGKV1-12/MZB1/IGLV2-14/IGLV4-69/IGKV1-39/IGKV2D-29/IGLV8-61/IGLV7-46/IGKV5-2 |
| GO:0042073 | intraciliary transport | 14/947 | 39/18800 | 2.85E-09 | 2.63E-07 | LCA5L/TTC26/MAK/IFT81/TTC30B/DYNC2H1/TRAF3IP1/TTC21A/IFT46/TTC30A/IFT172/RABL2B/IFT27/LCA5 |
| GO:0002768 | immune response-regulating cell surface receptor signaling pathway | 44/947 | 328/18800 | 2.98E-09 | 2.68E-07 | VTCN1/CD24/PIGR/HHLA2/IGHV1-18/IGHV3-23/IGHV2-26/IGHV2-5/IGLC2/IGHV3-15/IGHV4-39/IGLL5/IGHG1/IGHG4/IGHV3-48/MAPK10/IGHG3/IGHV3-33/IGHV4-61/IGKC/IGHV3-11/IGHV4-34/IGHA2/IGHV3-43/IGLC3/IGHV3-74/IGHV1-3/IGHV6-1/IGHV1-69/IGHV3-13/IGHV3-21/IGHV5-51/IGHA1/IGHV3-64/IGHG2/IGHV4-59/IGHV3-30/IGHV3-7/IGHV1OR15-1/IGLC1/IGHV3-49/MS4A2/IGLC6/IGHV3-66 |
| GO:0060287 | epithelial cilium movement involved in determination of left/right asymmetry | 8/947 | 12/18800 | 1.66E-08 | 1.45E-06 | CFAP53/CFAP45/DNAH11/CCDC39/CCDC103/DNAAF1/RFX3/CCDC40 |
| GO:0003356 | regulation of cilium beat frequency | 9/947 | 16/18800 | 1.67E-08 | 1.45E-06 | CFAP43/GAS2L2/CFAP45/DNAH11/CCDC39/DNAAF1/TTLL6/CCDC40/CFAP206 |
| GO:0002429 | immune response-activating cell surface receptor signaling pathway | 40/947 | 300/18800 | 1.85E-08 | 1.52E-06 | VTCN1/HHLA2/IGHV1-18/IGHV3-23/IGHV2-26/IGHV2-5/IGLC2/IGHV3-15/IGHV4-39/IGLL5/IGHG1/IGHG4/IGHV3-48/IGHG3/IGHV3-33/IGHV4-61/IGKC/IGHV3-11/IGHV4-34/IGHA2/IGHV3-43/IGLC3/IGHV3-74/IGHV1-3/IGHV6-1/IGHV1-69/IGHV3-13/IGHV3-21/IGHV5-51/IGHA1/IGHV3-64/IGHG2/IGHV4-59/IGHV3-30/IGHV3-7/IGHV1OR15-1/IGLC1/IGHV3-49/IGLC6/IGHV3-66 |
| GO:0002757 | immune response-activating signal transduction | 40/947 | 300/18800 | 1.85E-08 | 1.52E-06 | VTCN1/HHLA2/IGHV1-18/IGHV3-23/IGHV2-26/IGHV2-5/IGLC2/IGHV3-15/IGHV4-39/IGLL5/IGHG1/IGHG4/IGHV3-48/IGHG3/IGHV3-33/IGHV4-61/IGKC/IGHV3-11/IGHV4-34/IGHA2/IGHV3-43/IGLC3/IGHV3-74/IGHV1-3/IGHV6-1/IGHV1-69/IGHV3-13/IGHV3-21/IGHV5-51/IGHA1/IGHV3-64/IGHG2/IGHV4-59/IGHV3-30/IGHV3-7/IGHV1OR15-1/IGLC1/IGHV3-49/IGLC6/IGHV3-66 |
| GO:0042113 | B cell activation | 43/947 | 336/18800 | 1.86E-08 | 1.52E-06 | FOXJ1/IGHV1-18/IGHV3-23/IGHV2-26/IGHV2-5/POU2AF1/IGLC2/IGHV3-15/IGHV4-39/IGLL5/IGHG1/IGHG4/HMGB3/IGHV3-48/IGHG3/IGHV3-33/IGHV4-61/IGKC/IGHV3-11/IGHV4-34/IL7/IGHA2/IGHV3-43/IGLC3/IGHV3-74/IGHV1-3/IGHV6-1/IGHV1-69/IGHV3-13/IGHV3-21/IGHV5-51/IGHA1/IGHV3-64/IGHG2/IGHV4-59/IGHV3-30/IGHV3-7/IGHV1OR15-1/MZB1/IGLC1/IGHV3-49/IGLC6/IGHV3-66 |
| GO:0022412 | cellular process involved in reproduction in multicellular organism | 48/947 | 406/18800 | 3.55E-08 | 2.85E-06 | NME5/RSPH1/PACRG/DRC7/BMPR1B/INHBB/IQCG/CATSPERD/CFAP43/MNS1/CFAP58/ROPN1L/SPAG6/TRIP13/CFAP65/CCNA1/CFAP157/LGR5/SPAG16/CFAP47/DPY19L2P2/MEIG1/FMN2/TTC26/DPY19L2P1/CFAP69/ROPN1B/NPHP1/DPY19L2/STRBP/SPEF2/CFAP206/TTC21A/WNT4/ROPN1/OCA2/ARMC2/PCSK4/CATSPERE/NPM2/CFAP44/DMRTA1/SPIRE2/SPINK1/RFX2/CABYR/DNAH1/TDRD5 |
| GO:0002460 | adaptive immune response based on somatic recombination of immune receptors built from immunoglobulin superfamily domains | 45/947 | 370/18800 | 4.11E-08 | 3.23E-06 | C6/FOXJ1/IGHV1-18/IGHV3-23/IGHV2-26/IGHV2-5/IGLC2/IGHV3-15/IGHV4-39/IGLL5/IGHG1/IGHG4/IGHV3-48/IGHG3/IGHV3-33/IGHV4-61/IGKC/IGHV3-11/IGHV4-34/IGHA2/IGHV3-43/IGLC3/IGHV3-74/IL12A/IGHV1-3/IGHV6-1/IGHV1-69/IGHV3-13/IGHV3-21/IGHV5-51/IGHA1/IL13RA2/IGHV3-64/IGHG2/IGHV4-59/SUSD4/IGHV3-30/IGHV3-7/IGHV1OR15-1/IGLC1/IGHV3-49/IGLC6/CXCL13/IGHV3-66/CCL19 |
| GO:0002696 | positive regulation of leukocyte activation | 49/947 | 421/18800 | 4.21E-08 | 3.25E-06 | VTCN1/CD24/HHLA2/IGHV1-18/IGHV3-23/MYB/IGHV2-26/IGHV2-5/IGLC2/IGHV3-15/IGHV4-39/IGLL5/IGHG1/IGHG4/IGHV3-48/IGHG3/IGHV3-33/IGHV4-61/IGKC/IGHV3-11/IGHV4-34/IL7/IGHA2/IGHV3-43/IGLC3/IGHV3-74/IL12A/IGHV1-3/IGHV6-1/IGHV1-69/IGHV3-13/IGHV3-21/IGHV5-51/IGHA1/EFNB3/IGHV3-64/IGHG2/IGHV4-59/IGHV3-30/IGHV3-7/IGHV1OR15-1/TACR1/IGLC1/TNFSF11/IGHV3-49/IGLC6/IGHV3-66/CCL19/HAMP |
| GO:0007389 | pattern specification process | 52/947 | 463/18800 | 5.27E-08 | 4.00E-06 | TP63/SIX1/ENKUR/DRC1/CFAP53/EYA1/DAW1/BMPR1B/DNAI2/CFAP52/DNAH5/DNAI1/FOXJ1/MNS1/CFAP45/PIFO/HES2/CFC1/DNAH11/CCDC39/FOXA1/RPGRIP1L/CCDC103/DNAAF1/HOXB2/HOXB3/DNAAF4/DZIP1L/LMX1B/HOXC4/RFX3/DPCD/DYNC2H1/CCDC40/SIX2/ERBB4/CFC1B/TMEM107/IFT172/TCTN1/CC2D2A/GREM1/HOXB1/FUZ/CDK20/FOXN4/FEZF1/GRHL3/ISL1/MEOX1/NEUROD1/TDRD5 |
| GO:0002449 | lymphocyte mediated immunity | 44/947 | 365/18800 | 7.53E-08 | 5.61E-06 | C6/FOXJ1/IGHV1-18/IGHV3-23/IGHV2-26/IGHV2-5/IGLC2/IGHV3-15/IGHV4-39/IGLL5/IGHG1/IGHG4/IGHV3-48/IGHG3/IGHV3-33/IGHV4-61/IGKC/IGHV3-11/IGHV4-34/IGHA2/IGHV3-43/IGLC3/IGHV3-74/IL12A/IGHV1-3/IGHV6-1/IGHV1-69/IGHV3-13/IGHV3-21/IGHV5-51/IGHA1/IL13RA2/IGHV3-64/IGHG2/IGHV4-59/SUSD4/IGHV3-30/IGHV3-7/TUBB4B/IGHV1OR15-1/IGLC1/IGHV3-49/IGLC6/IGHV3-66 |
| GO:0090660 | cerebrospinal fluid circulation | 8/947 | 14/18800 | 9.22E-08 | 6.76E-06 | DAW1/CFAP43/CFAP45/DNAH9/CCDC39/CFAP221/SPAG16/CFAP54 |
| GO:0050867 | positive regulation of cell activation | 49/947 | 436/18800 | 1.26E-07 | 9.06E-06 | VTCN1/CD24/HHLA2/IGHV1-18/IGHV3-23/MYB/IGHV2-26/IGHV2-5/IGLC2/IGHV3-15/IGHV4-39/IGLL5/IGHG1/IGHG4/IGHV3-48/IGHG3/IGHV3-33/IGHV4-61/IGKC/IGHV3-11/IGHV4-34/IL7/IGHA2/IGHV3-43/IGLC3/IGHV3-74/IL12A/IGHV1-3/IGHV6-1/IGHV1-69/IGHV3-13/IGHV3-21/IGHV5-51/IGHA1/EFNB3/IGHV3-64/IGHG2/IGHV4-59/IGHV3-30/IGHV3-7/IGHV1OR15-1/TACR1/IGLC1/TNFSF11/IGHV3-49/IGLC6/IGHV3-66/CCL19/HAMP |
| GO:0006909 | phagocytosis | 39/947 | 310/18800 | 1.35E-07 | 9.55E-06 | IGHV1-18/IGHV3-23/IGHV2-26/IGHV2-5/IGLC2/IGHV3-15/IGHV4-39/IGLL5/IGHG1/IGHG4/IGHV3-48/IGHG3/IGHV3-33/IGHV4-61/IGKC/IGHV3-11/IGHV4-34/IGHA2/ALOX15/IGHV3-43/IGLC3/IGHV3-74/IGHV1-3/IGHV6-1/IGHV1-69/IGHV3-13/IGHV3-21/IGHV5-51/IGHA1/IGHV3-64/IGHG2/IGHV4-59/IGHV3-30/IGHV3-7/IGHV1OR15-1/IGLC1/IGHV3-49/IGLC6/IGHV3-66 |
| GO:0036158 | outer dynein arm assembly | 9/947 | 22/18800 | 5.52E-07 | 3.85E-05 | DAW1/DNAI2/DNAH5/DNAI1/ZMYND10/CCDC103/DNAAF1/DNAL1/DNAAF4 |
| GO:0061512 | protein localization to cilium | 16/947 | 75/18800 | 7.86E-07 | 5.40E-05 | CFAP58/DNAH11/CCDC39/ZMYND10/MAPK15/DZIP1L/TTC26/ROPN1B/DYNC2H1/TTC21A/TMEM107/ROPN1/TCTN1/CC2D2A/CDK20/TCTN2 |
| GO:0002253 | activation of immune response | 43/947 | 386/18800 | 9.39E-07 | 6.35E-05 | VTCN1/C6/HHLA2/IGHV1-18/IGHV3-23/IGHV2-26/IGHV2-5/IGLC2/IGHV3-15/IGHV4-39/IGLL5/IGHG1/IGHG4/IGHV3-48/IGHG3/IGHV3-33/IGHV4-61/IGKC/IGHV3-11/IGHV4-34/IGHA2/IGHV3-43/IGLC3/IGHV3-74/IGHV1-3/IGHV6-1/IGHV1-69/IGHV3-13/IGHV3-21/IGHV5-51/IGHA1/IGHV3-64/IGHG2/MATR3/IGHV4-59/SUSD4/IGHV3-30/IGHV3-7/IGHV1OR15-1/IGLC1/IGHV3-49/IGLC6/IGHV3-66 |
| GO:0007586 | digestion | 21/947 | 137/18800 | 4.97E-06 | 0.0003308 | MUC4/VSIG1/AQP5/ARX/CHIT1/SST/MUC2/OXTR/MUC13/INAVA/GCNT3/TFF3/UCN3/GHRL/ADRA2A/TACR1/SPINK1/PRSS2/MOGAT2/NEUROD1/HAMP |
| GO:0002764 | immune response-regulating signaling pathway | 48/947 | 482/18800 | 5.41E-06 | 0.0003549 | VTCN1/CD24/PIGR/HHLA2/IGHV1-18/IGHV3-23/IGHV2-26/IGHV2-5/IGLC2/IGHV3-15/IGHV4-39/IGLL5/IGHG1/IGHG4/BPIFB1/LTF/IGHV3-48/MAPK10/IGHG3/TSPAN6/IGHV3-33/IGHV4-61/IGKC/IGHV3-11/IGHV4-34/IGHA2/IGHV3-43/IGLC3/IGHV3-74/INAVA/IGHV1-3/IGHV6-1/IGHV1-69/IGHV3-13/IGHV3-21/IGHV5-51/IGHA1/IGHV3-64/IGHG2/IGHV4-59/IGHV3-30/IGHV3-7/IGHV1OR15-1/IGLC1/IGHV3-49/MS4A2/IGLC6/IGHV3-66 |
| GO:0001895 | retina homeostasis | 15/947 | 80/18800 | 9.37E-06 | 0.0006049 | CDH3/PROM1/PIGR/RP1/USH1G/WHRN/LTF/IGHG3/IGKC/MAK/IGHA2/JCHAIN/IGHA1/USH1C/LCA5 |
| GO:0060249 | anatomical structure homeostasis | 35/947 | 319/18800 | 1.32E-05 | 0.0008418 | CDH3/MUC4/VSIG1/SPP1/PROM1/PIGR/RP1/USH1G/NELL2/WHRN/MUC2/MAP1A/COMP/LTF/IGHG3/IGKC/MAK/IL7/IGHA2/TJP3/CLDN1/MUC13/INAVA/ATP2B3/JCHAIN/TFF3/IGHA1/USH1C/PLG/LCA5/TNFSF11/CALCA/NEUROD1/HAMP/CYTL1 |
| GO:0002443 | leukocyte mediated immunity | 45/947 | 457/18800 | 1.41E-05 | 0.0008836 | C6/FOXJ1/IGHV1-18/IGHV3-23/IGHV2-26/IGHV2-5/IGLC2/IGHV3-15/IGHV4-39/IGLL5/IGHG1/IGHG4/IGHV3-48/IGHG3/IGHV3-33/IGHV4-61/IGKC/IGHV3-11/IGHV4-34/IGHA2/IGHV3-43/IGLC3/IGHV3-74/IL12A/IGHV1-3/CHGA/IGHV6-1/IGHV1-69/IGHV3-13/IGHV3-21/IGHV5-51/IGHA1/IL13RA2/IGHV3-64/IGHG2/IGHV4-59/SUSD4/IGHV3-30/IGHV3-7/TUBB4B/IGHV1OR15-1/IGLC1/IGHV3-49/IGLC6/IGHV3-66 |
| GO:0001894 | tissue homeostasis | 31/947 | 272/18800 | 1.93E-05 | 0.0011954 | CDH3/MUC4/VSIG1/SPP1/PROM1/PIGR/RP1/USH1G/WHRN/MUC2/COMP/LTF/IGHG3/IGKC/MAK/IL7/IGHA2/TJP3/CLDN1/MUC13/INAVA/JCHAIN/TFF3/IGHA1/USH1C/LCA5/TNFSF11/CALCA/NEUROD1/HAMP/CYTL1 |
| GO:0048839 | inner ear development | 24/947 | 192/18800 | 3.75E-05 | 0.0022858 | SIX1/SIX4/SOX2/STOX1/EYA1/KCNK2/USH1G/RPGRIP1L/CTHRC1/LGR5/WHRN/SLC44A4/COL11A1/LRTOMT/USH1C/IFT27/SLC17A8/ATP6V1B1/TFAP2A/ALDH1A3/PRRX1/GRHL3/NEUROD1/CYTL1 |
| GO:0043583 | ear development | 26/947 | 219/18800 | 4.44E-05 | 0.0026678 | SIX1/SIX4/SOX2/STOX1/EYA1/KCNK2/USH1G/RPGRIP1L/CTHRC1/LGR5/WHRN/SLC44A4/SIX2/COL11A1/LRTOMT/USH1C/IFT27/SLC17A8/STRA6/ATP6V1B1/TFAP2A/ALDH1A3/PRRX1/GRHL3/NEUROD1/CYTL1 |
| GO:0022600 | digestive system process | 16/947 | 104/18800 | 6.21E-05 | 0.003678 | MUC4/VSIG1/AQP5/MUC2/OXTR/MUC13/INAVA/GCNT3/TFF3/GHRL/ADRA2A/TACR1/SPINK1/MOGAT2/NEUROD1/HAMP |
| GO:0030277 | maintenance of gastrointestinal epithelium | 7/947 | 22/18800 | 7.05E-05 | 0.00412 | MUC4/VSIG1/MUC2/MUC13/INAVA/TFF3/NEUROD1 |
| GO:0007224 | smoothened signaling pathway | 19/947 | 140/18800 | 7.79E-05 | 0.0044931 | TMEM231/FOXA1/RPGRIP1L/B9D1/DZIP1L/TTC26/IQUB/IFT81/DYNC2H1/IFT172/TCTN1/CC2D2A/FUZ/CDK20/IFT27/TMEM17/TCTN2/PRRX1/PTCH2 |
| GO:0048562 | embryonic organ morphogenesis | 31/947 | 294/18800 | 8.58E-05 | 0.0048871 | SIX1/SIX4/STOX1/EYA1/USH1G/CCDC39/CTHRC1/CCDC103/DNAAF1/WHRN/HOXB2/SLC44A4/HOXB3/WNT16/HOXC4/CCDC40/SIX2/IFT172/ALX1/HOXB1/COL11A1/USH1C/FUZ/FOXN4/STRA6/ATP6V1B1/TFAP2A/ALDH1A3/PRRX1/GRHL3/NEUROD1 |
| GO:0048240 | sperm capacitation | 8/947 | 31/18800 | 0.0001126 | 0.0063253 | CATSPERD/ROPN1L/ROPN1B/ROPN1/PCSK4/CATSPERE/SPINK1/CABYR |
| GO:0017158 | regulation of calcium ion-dependent exocytosis | 9/947 | 40/18800 | 0.0001338 | 0.0074215 | DOC2A/BAIAP3/SYT5/SYT8/ADRA2A/SYT12/CACNA1G/TRPV6/SCAMP5 |
| GO:0090596 | sensory organ morphogenesis | 28/947 | 266/18800 | 0.0001907 | 0.0104462 | SIX1/SIX4/STOX1/EYA1/NTRK2/AQP5/PROM1/RP1/USH1G/TFAP2B/RPGRIP1L/CTHRC1/WHRN/SLC44A4/WNT16/DZANK1/SIX2/IFT172/TENM3/COL11A1/USH1C/FOXN4/STRA6/ATP6V1B1/TFAP2A/ALDH1A3/PRRX1/GRHL3 |
| GO:0042472 | inner ear morphogenesis | 15/947 | 103/18800 | 0.0001958 | 0.0105071 | SIX1/SIX4/STOX1/EYA1/USH1G/CTHRC1/WHRN/SLC44A4/COL11A1/USH1C/ATP6V1B1/TFAP2A/ALDH1A3/PRRX1/GRHL3 |
| GO:0016331 | morphogenesis of embryonic epithelium | 19/947 | 150/18800 | 0.0001967 | 0.0105071 | TP63/SIX1/SIX4/RET/CTHRC1/GDF7/WNT16/RGMA/WNT4/IFT172/TCTN1/CC2D2A/SALL4/GREM1/FUZ/CDK20/TFAP2A/ALDH1A3/GRHL3 |
| GO:0008544 | epidermis development | 34/947 | 355/18800 | 0.0002589 | 0.0136635 | TP63/KRT15/CDH3/FERMT1/COL17A1/KRT5/LGR5/GRHL1/KRT17/WHRN/SLC44A4/SOX21/WNT16/NGFR/COL7A1/KRT80/KRT4/IVL/IFT172/ZNF750/LRTOMT/TMEM132E/USH1C/FUZ/KRT6B/SFN/KLK14/PTCH2/GRHL3/KRT6A/WNT10A/FOXN1/CRABP2/PTHLH |
| GO:1905515 | non-motile cilium assembly | 11/947 | 63/18800 | 0.0002773 | 0.0144549 | RP1/RPGRIP1L/CCDC13/CEP126/MAK/DYNC2H1/TMEM107/IFT172/CC2D2A/FUZ/TMEM17 |
| GO:0010970 | transport along microtubule | 19/947 | 157/18800 | 0.0003552 | 0.0182961 | LCA5L/MAP1A/KIF1A/TTC26/MAK/IFT81/TTC30B/DYNC2H1/AGBL4/CNIH2/TRAF3IP1/TTC21A/IFT46/TTC30A/IFT172/RABL2B/IFT27/LCA5/AP3B2 |
| GO:0032886 | regulation of microtubule-based process | 26/947 | 249/18800 | 0.0003609 | 0.0183715 | STMND1/DRC1/CCDC65/RSPH4A/CFAP43/GAS2L2/CFAP45/SPEF1/DNAH11/CCDC39/DNAAF1/MAP6/MAP1A/TTLL6/MAPK15/MAPRE3/CFAP69/TRPV4/TMEM67/CCDC40/CFAP206/CNIH2/TRAF3IP1/CFAP298/EPPIN/TACR1 |
| GO:0043584 | nose development | 5/947 | 14/18800 | 0.0004387 | 0.0220713 | SIX1/SIX4/RPGRIP1L/STRA6/ALDH1A3 |
| GO:0042471 | ear morphogenesis | 16/947 | 123/18800 | 0.0004535 | 0.0225539 | SIX1/SIX4/STOX1/EYA1/USH1G/CTHRC1/WHRN/SLC44A4/SIX2/COL11A1/USH1C/ATP6V1B1/TFAP2A/ALDH1A3/PRRX1/GRHL3 |
| GO:0060295 | regulation of cilium movement involved in cell motility | 7/947 | 29/18800 | 0.0004734 | 0.023016 | GAS2L2/CFAP45/TTLL6/CFAP69/CFAP206/EPPIN/TACR1 |
| GO:1902019 | regulation of cilium-dependent cell motility | 7/947 | 29/18800 | 0.0004734 | 0.023016 | GAS2L2/CFAP45/TTLL6/CFAP69/CFAP206/EPPIN/TACR1 |
| GO:0003002 | regionalization | 33/947 | 354/18800 | 0.0005132 | 0.0246757 | TP63/SIX1/BMPR1B/FOXJ1/MNS1/PIFO/HES2/CFC1/FOXA1/RPGRIP1L/DNAAF1/HOXB2/HOXB3/DZIP1L/LMX1B/HOXC4/DPCD/DYNC2H1/SIX2/CFC1B/TMEM107/IFT172/TCTN1/GREM1/HOXB1/FUZ/CDK20/FOXN4/FEZF1/ISL1/MEOX1/NEUROD1/TDRD5 |
| GO:0060972 | left/right pattern formation | 6/947 | 22/18800 | 0.0005981 | 0.0284399 | FOXJ1/MNS1/PIFO/DNAAF1/DPCD/IFT172 |
| GO:0048704 | embryonic skeletal system morphogenesis | 13/947 | 94/18800 | 0.0008456 | 0.0397697 | SIX1/SIX4/EYA1/HOXB2/HOXB3/HOXC4/SIX2/ALX1/HOXB1/COL11A1/FUZ/TFAP2A/PRRX1 |
| GO:0010669 | epithelial structure maintenance | 7/947 | 32/18800 | 0.0008948 | 0.0411873 | MUC4/VSIG1/MUC2/MUC13/INAVA/TFF3/NEUROD1 |
| GO:0090183 | regulation of kidney development | 7/947 | 32/18800 | 0.0008948 | 0.0411873 | SIX1/SIX4/RET/SIX2/WNT4/GREM1/AGTR2 |

**Table S3b GO enrichment analysis (biological process, BP) of down-regulated genes**

| **ID** | **Description** | **Gene Ratio** | **Bg Ratio** | **P-value** | **FDR** | **Gene ID** |
| --- | --- | --- | --- | --- | --- | --- |
| GO:0032103 | positive regulation of response to external stimulus | 29/280 | 442/18800 | 2.43E-11 | 8.83E-08 | IL1RL1/IL1B/BMP6/FPR2/NLRP12/MNDA/PGC/KLRD1/EDN1/ALOX5AP/SERPINE1/LPL/SH2D1B/S100A8/LILRA2/CCL4/CX3CR1/FFAR2/CCL24/TNF/FCN1/OASL/PTGS2/S100A12/FABP4/CX3CL1/TXK/TSLP/THBD |
| GO:0031349 | positive regulation of defense response | 23/280 | 289/18800 | 7.06E-11 | 1.28E-07 | IL1RL1/IL1B/FPR2/NLRP12/MNDA/PGC/KLRD1/ALOX5AP/SERPINE1/LPL/SH2D1B/S100A8/LILRA2/FFAR2/CCL24/TNF/FCN1/PTGS2/S100A12/FABP4/CX3CL1/TXK/TSLP |
| GO:0001819 | positive regulation of cytokine production | 29/280 | 475/18800 | 1.36E-10 | 1.64E-07 | IL1RL1/IL1B/CD83/IL17D/IRF1/SLC11A1/NLRP12/TBX21/MNDA/IL1A/SERPINE1/LPL/MEFV/LILRA2/IL18R1/PLA2G1B/LILRB2/FFAR2/TNF/FCN1/HEG1/PTGS2/CD244/CLEC4E/CX3CL1/TXK/RGCC/TSLP/ELANE |
| GO:0050900 | leukocyte migration | 25/280 | 384/18800 | 7.30E-10 | 6.61E-07 | ICAM1/IL1B/TREM1/FPR2/NLRP12/TBX21/SELPLG/IL1A/DBH/EDN1/SERPINE1/MYO1G/EDNRB/S100A8/C5AR2/CCL4/PLA2G1B/CX3CR1/FFAR2/CCL24/TNF/SELL/S100A12/CX3CL1/ELANE |
| GO:0050727 | regulation of inflammatory response | 25/280 | 394/18800 | 1.24E-09 | 8.99E-07 | IL1RL1/IL1B/FPR2/NLRP12/ALOX5AP/SERPINE1/LPL/MEFV/WFDC1/EDNRB/S100A8/CST7/FFAR2/CCL24/TNF/PTGS2/S100A12/FABP4/CX3CL1/GGT1/TSLP/FFAR4/NCF1/ELANE/CASP12 |
| GO:0031649 | heat generation | 7/280 | 16/18800 | 1.54E-09 | 9.30E-07 | IL1B/ADRB2/ADRB1/IL1A/EDNRB/TNF/PTGS2 |
| GO:0050729 | positive regulation of inflammatory response | 15/280 | 145/18800 | 4.56E-09 | 2.36E-06 | IL1RL1/IL1B/NLRP12/ALOX5AP/SERPINE1/LPL/S100A8/FFAR2/CCL24/TNF/PTGS2/S100A12/FABP4/CX3CL1/TSLP |
| GO:0001659 | temperature homeostasis | 16/280 | 177/18800 | 9.85E-09 | 4.47E-06 | IL1B/ADRB2/ACADL/ADRB1/IL1A/DBH/EPAS1/EDNRB/G0S2/IL18R1/TNF/PTGS2/FABP4/PRLR/SCD/FFAR4 |
| GO:0034472 | snRNA 3'-end processing | 8/280 | 31/18800 | 1.28E-08 | 5.17E-06 | CT45A9/CT45A2/CT45A1/CT45A8/CT45A7/CT45A3/CT45A5/CT45A10 |
| GO:0032757 | positive regulation of interleukin-8 production | 10/280 | 62/18800 | 2.49E-08 | 9.01E-06 | IL1B/IL17D/SERPINE1/LILRA2/PLA2G1B/FFAR2/TNF/FCN1/CD244/ELANE |
| GO:0016180 | snRNA processing | 8/280 | 36/18800 | 4.62E-08 | 1.52E-05 | CT45A9/CT45A2/CT45A1/CT45A8/CT45A7/CT45A3/CT45A5/CT45A10 |
| GO:0032496 | response to lipopolysaccharide | 20/280 | 333/18800 | 1.46E-07 | 4.41E-05 | IL1B/BMP6/SLC11A1/NOS1/SMAD6/IL1A/EDN1/SERPINE1/EDNRB/S100A8/LILRA2/LILRB2/HPGD/CX3CR1/TNF/PTGS2/CX3CL1/THBD/ELANE/MPO |
| GO:0001660 | fever generation | 5/280 | 11/18800 | 3.04E-07 | 8.47E-05 | IL1B/IL1A/EDNRB/TNF/PTGS2 |
| GO:0032677 | regulation of interleukin-8 production | 11/280 | 102/18800 | 3.53E-07 | 8.54E-05 | IL1B/IL17D/SERPINE1/C5AR2/LILRA2/PLA2G1B/FFAR2/TNF/FCN1/CD244/ELANE |
| GO:0002237 | response to molecule of bacterial origin | 20/280 | 354/18800 | 3.88E-07 | 8.54E-05 | IL1B/BMP6/SLC11A1/NOS1/SMAD6/IL1A/EDN1/SERPINE1/EDNRB/S100A8/LILRA2/LILRB2/HPGD/CX3CR1/TNF/PTGS2/CX3CL1/THBD/ELANE/MPO |
| GO:0032637 | interleukin-8 production | 11/280 | 103/18800 | 3.90E-07 | 8.54E-05 | IL1B/IL17D/SERPINE1/C5AR2/LILRA2/PLA2G1B/FFAR2/TNF/FCN1/CD244/ELANE |
| GO:0072503 | cellular divalent inorganic cation homeostasis | 24/280 | 494/18800 | 4.19E-07 | 8.54E-05 | GRIA1/HAP1/MCOLN3/FPR2/SLC24A4/RAMP3/FPR1/UBASH3B/SLC11A1/NOS1/EDN1/STC2/EDNRB/S100A8/C5AR2/CD52/F2RL3/PLA2G1B/ADCY8/CX3CR1/CX3CL1/FFAR4/TRPC3/ELANE |
| GO:0051480 | regulation of cytosolic calcium ion concentration | 20/280 | 356/18800 | 4.24E-07 | 8.54E-05 | GRIA1/HAP1/MCOLN3/FPR2/SLC24A4/RAMP3/FPR1/UBASH3B/NOS1/EDN1/EDNRB/C5AR2/CD52/F2RL3/PLA2G1B/ADCY8/CX3CR1/CX3CL1/FFAR4/TRPC3 |
| GO:0007204 | positive regulation of cytosolic calcium ion concentration | 19/280 | 325/18800 | 4.55E-07 | 8.68E-05 | HAP1/MCOLN3/FPR2/SLC24A4/RAMP3/FPR1/UBASH3B/NOS1/EDN1/EDNRB/C5AR2/CD52/F2RL3/PLA2G1B/ADCY8/CX3CR1/CX3CL1/FFAR4/TRPC3 |
| GO:0043628 | ncRNA 3'-end processing | 8/280 | 50/18800 | 6.84E-07 | 0.0001201 | CT45A9/CT45A2/CT45A1/CT45A8/CT45A7/CT45A3/CT45A5/CT45A10 |
| GO:0070371 | ERK1 and ERK2 cascade | 19/280 | 335/18800 | 7.20E-07 | 0.0001201 | ICAM1/IL1B/FPR2/RAMP3/NDRG4/NLRP12/IL1A/EDN1/DUSP6/BMP2/BMPER/C5AR2/SPRY4/SEMA6A/CCL4/CCL24/TNF/CX3CL1/FFAR4 |
| GO:0003158 | endothelium development | 12/280 | 133/18800 | 7.29E-07 | 0.0001201 | ICAM1/IL1B/BMP6/MYADM/TMEM100/EDNRB/DLL4/ACVRL1/TNF/HEG1/COL22A1/GJA5 |
| GO:0043270 | positive regulation of ion transport | 17/280 | 273/18800 | 7.78E-07 | 0.0001211 | IL1B/ARC/ADRB2/HAP1/RAMP3/NOS1/IL1A/EDN1/SLC6A4/LILRA2/F2RL3/CCL4/PLA2G1B/CHRM1/CX3CL1/TRPC3/STAC |
| GO:0007162 | negative regulation of cell adhesion | 18/280 | 305/18800 | 8.02E-07 | 0.0001211 | BMP6/MYADM/IRF1/UBASH3B/TBX21/ILDR2/SERPINE1/BMP2/LRRC32/PAG1/SPRY4/SEMA6A/FAM107A/LILRB2/TNR/ACVRL1/CX3CL1/RGCC |
| GO:0070372 | regulation of ERK1 and ERK2 cascade | 18/280 | 311/18800 | 1.06E-06 | 0.000154 | ICAM1/IL1B/FPR2/RAMP3/NDRG4/NLRP12/IL1A/DUSP6/BMP2/BMPER/C5AR2/SPRY4/SEMA6A/CCL4/CCL24/TNF/CX3CL1/FFAR4 |
| GO:0007159 | leukocyte cell-cell adhesion | 20/280 | 381/18800 | 1.22E-06 | 0.0001708 | ICAM1/IL1B/CD83/IRF1/TBX21/IL7R/SELPLG/ILDR2/IL1A/LRRC32/PAG1/S100A8/OLR1/LILRB2/CX3CR1/TNF/SELL/ELANE/SIRPB1/CLEC4M |
| GO:0006874 | cellular calcium ion homeostasis | 22/280 | 456/18800 | 1.45E-06 | 0.0001948 | GRIA1/HAP1/MCOLN3/FPR2/SLC24A4/RAMP3/FPR1/UBASH3B/NOS1/EDN1/STC2/EDNRB/C5AR2/CD52/F2RL3/PLA2G1B/ADCY8/CX3CR1/CX3CL1/FFAR4/TRPC3/ELANE |
| GO:0006816 | calcium ion transport | 21/280 | 424/18800 | 1.67E-06 | 0.0002167 | HAP1/MCOLN3/SLC24A4/RAMP3/CATSPER1/UBASH3B/NOS1/EDN1/STC2/EDNRB/LILRA2/GPM6A/F2RL3/CCL4/PLA2G1B/LILRB2/CACNA2D2/PTGS2/CX3CL1/TRPC3/STAC |
| GO:0097529 | myeloid leukocyte migration | 15/280 | 229/18800 | 1.88E-06 | 0.0002345 | IL1B/TREM1/FPR2/IL1A/EDN1/SERPINE1/EDNRB/S100A8/C5AR2/CCL4/PLA2G1B/CX3CR1/CCL24/S100A12/CX3CL1 |
| GO:0042060 | wound healing | 21/280 | 429/18800 | 2.01E-06 | 0.0002365 | TNFRSF12A/TSPAN32/UBASH3B/SLC11A1/WNT7A/IL1A/EDN1/SERPINE1/WFDC1/S100A8/HBEGF/MYOZ1/F2RL3/ODAM/ACVRL1/TNF/CX3CL1/TXK/MPIG6B/THBD/SLC4A1 |
| GO:0120162 | positive regulation of cold-induced thermogenesis | 10/280 | 98/18800 | 2.02E-06 | 0.0002365 | ADRB2/ACADL/ADRB1/DBH/EPAS1/G0S2/FABP4/PRLR/SCD/FFAR4 |
| GO:0055074 | calcium ion homeostasis | 22/280 | 468/18800 | 2.22E-06 | 0.0002514 | GRIA1/HAP1/MCOLN3/FPR2/SLC24A4/RAMP3/FPR1/UBASH3B/NOS1/EDN1/STC2/EDNRB/C5AR2/CD52/F2RL3/PLA2G1B/ADCY8/CX3CR1/CX3CL1/FFAR4/TRPC3/ELANE |
| GO:0030595 | leukocyte chemotaxis | 15/280 | 236/18800 | 2.72E-06 | 0.0002992 | IL1B/TREM1/FPR2/EDN1/SERPINE1/EDNRB/S100A8/C5AR2/CCL4/PLA2G1B/CX3CR1/FFAR2/CCL24/S100A12/CX3CL1 |
| GO:1990266 | neutrophil migration | 11/280 | 128/18800 | 3.43E-06 | 0.0003654 | IL1B/TREM1/IL1A/EDN1/S100A8/C5AR2/CCL4/PLA2G1B/CCL24/S100A12/CX3CL1 |
| GO:0030593 | neutrophil chemotaxis | 10/280 | 106/18800 | 4.14E-06 | 0.0004209 | IL1B/TREM1/EDN1/S100A8/C5AR2/CCL4/PLA2G1B/CCL24/S100A12/CX3CL1 |
| GO:0001906 | cell killing | 13/280 | 185/18800 | 4.26E-06 | 0.0004209 | ICAM1/TREM1/UNC13D/GNLY/PRF1/IL7R/KLRD1/GZMB/EMP2/CX3CR1/ULBP2/S100A12/ELANE |
| GO:0001909 | leukocyte mediated cytotoxicity | 11/280 | 131/18800 | 4.29E-06 | 0.0004209 | ICAM1/TREM1/UNC13D/PRF1/IL7R/KLRD1/GZMB/EMP2/CX3CR1/ULBP2/ELANE |
| GO:0016073 | snRNA metabolic process | 8/280 | 64/18800 | 4.71E-06 | 0.0004494 | CT45A9/CT45A2/CT45A1/CT45A8/CT45A7/CT45A3/CT45A5/CT45A10 |
| GO:0022407 | regulation of cell-cell adhesion | 21/280 | 456/18800 | 5.22E-06 | 0.0004851 | IL1B/BMP6/CD83/MYADM/IRF1/UBASH3B/TBX21/IL7R/FSTL3/ILDR2/IL1A/BMP2/LRRC32/PAG1/LILRB2/TNR/TNF/CX3CL1/RGCC/ELANE/SIRPB1 |
| GO:0060326 | cell chemotaxis | 17/280 | 315/18800 | 5.43E-06 | 0.0004923 | IL1B/TREM1/FPR2/BIN2/EDN1/SERPINE1/EDNRB/S100A8/C5AR2/HBEGF/CCL4/PLA2G1B/CX3CR1/FFAR2/CCL24/S100A12/CX3CL1 |
| GO:0002683 | negative regulation of immune system process | 20/280 | 425/18800 | 6.39E-06 | 0.0005654 | IL1RL1/IL17D/TSPAN32/IRF1/UBASH3B/TBX21/MNDA/IL7R/KLRD1/FSTL3/ILDR2/LRRC32/PAG1/C5AR2/CST7/LILRB2/INHBA/CX3CR1/TNF/CX3CL1 |
| GO:0002523 | leukocyte migration involved in inflammatory response | 5/280 | 19/18800 | 6.93E-06 | 0.0005982 | S100A8/FFAR2/TNF/CX3CL1/ELANE |
| GO:0032102 | negative regulation of response to external stimulus | 20/280 | 429/18800 | 7.34E-06 | 0.0006072 | FPR2/UBASH3B/NLRP12/KLRD1/EDN1/SERPINE1/MEFV/WFDC1/C5AR2/LILRA2/CST7/MYOZ1/SEMA6A/TNR/SEMA3G/TNF/CX3CL1/FFAR4/THBD/ELANE |
| GO:0002526 | acute inflammatory response | 10/280 | 113/18800 | 7.37E-06 | 0.0006072 | IL1B/TREM1/IL1A/ALOX5AP/EDNRB/S100A8/FFAR2/TNF/PTGS2/ELANE |
| GO:0045446 | endothelial cell differentiation | 10/280 | 117/18800 | 1.00E-05 | 0.0008096 | ICAM1/IL1B/BMP6/MYADM/TMEM100/EDNRB/ACVRL1/TNF/HEG1/COL22A1 |
| GO:0050805 | negative regulation of synaptic transmission | 8/280 | 71/18800 | 1.03E-05 | 0.0008156 | GRIA1/IL1B/ARC/SLC6A4/LILRB2/ADCY8/TNR/PTGS2 |
| GO:0010959 | regulation of metal ion transport | 19/280 | 403/18800 | 1.07E-05 | 0.0008217 | DPP6/ADRB2/HAP1/RAMP3/UBASH3B/NOS1/STC2/LILRA2/F2RL3/CCL4/PLA2G1B/HECW2/KCNIP1/LILRB2/PTGS2/CX3CL1/TRPC3/PCSK9/STAC |
| GO:0106106 | cold-induced thermogenesis | 11/280 | 146/18800 | 1.22E-05 | 0.0009003 | ADRB2/ACADL/ADRB1/DBH/EPAS1/G0S2/IL18R1/FABP4/PRLR/SCD/FFAR4 |
| GO:0120161 | regulation of cold-induced thermogenesis | 11/280 | 146/18800 | 1.22E-05 | 0.0009003 | ADRB2/ACADL/ADRB1/DBH/EPAS1/G0S2/IL18R1/FABP4/PRLR/SCD/FFAR4 |
| GO:0150076 | neuroinflammatory response | 8/280 | 73/18800 | 1.27E-05 | 0.0009201 | IL1B/FPR2/CST7/ADCY8/CX3CR1/TNF/PTGS2/CX3CL1 |
| GO:0050832 | defense response to fungus | 7/280 | 53/18800 | 1.29E-05 | 0.0009201 | GNLY/S100A8/CX3CR1/CLEC4E/S100A12/ELANE/MPO |
| GO:0032602 | chemokine production | 9/280 | 98/18800 | 1.57E-05 | 0.0010936 | IL1RL1/IL1B/TREM1/LPL/MEFV/FFAR2/TNF/TSLP/ELANE |
| GO:0043410 | positive regulation of MAPK cascade | 21/280 | 491/18800 | 1.60E-05 | 0.0010943 | ICAM1/IL1B/ADRB2/FPR2/RAMP3/NDRG4/WNT7A/IL1A/EDN1/BMP2/BMPER/C5AR2/CCL4/PLA2G1B/CCL24/TNF/S100A12/CX3CL1/FFAR4/NCF1/ELANE |
| GO:2001236 | regulation of extrinsic apoptotic signaling pathway | 11/280 | 153/18800 | 1.89E-05 | 0.0012703 | ICAM1/IL1B/ITPRIP/TNFRSF12A/IL1A/DBH/SERPINE1/G0S2/INHBA/TNF/CX3CL1 |
| GO:0045926 | negative regulation of growth | 14/280 | 245/18800 | 1.96E-05 | 0.0012927 | ENPP1/ADRB2/ADRB1/STC2/SLC6A4/WFDC1/HYAL1/MYOZ1/SEMA6A/OSGIN1/INHBA/TNR/SEMA3G/ACVRL1 |
| GO:0097530 | granulocyte migration | 11/280 | 154/18800 | 2.01E-05 | 0.001302 | IL1B/TREM1/IL1A/EDN1/S100A8/C5AR2/CCL4/PLA2G1B/CCL24/S100A12/CX3CL1 |
| GO:0046456 | icosanoid biosynthetic process | 7/280 | 57/18800 | 2.11E-05 | 0.0013422 | IL1B/PLA2G4F/EDN1/ALOX5AP/PLA2G1B/PTGS2/GGT1 |
| GO:0031650 | regulation of heat generation | 4/280 | 12/18800 | 2.17E-05 | 0.0013565 | IL1B/EDNRB/TNF/PTGS2 |
| GO:0071621 | granulocyte chemotaxis | 10/280 | 128/18800 | 2.21E-05 | 0.0013606 | IL1B/TREM1/EDN1/S100A8/C5AR2/CCL4/PLA2G1B/CCL24/S100A12/CX3CL1 |
| GO:0050866 | negative regulation of cell activation | 13/280 | 216/18800 | 2.26E-05 | 0.0013648 | TSPAN32/IRF1/UBASH3B/TBX21/MNDA/ILDR2/LRRC32/PAG1/CST7/LILRB2/INHBA/CX3CL1/THBD |
| GO:0032612 | interleukin-1 production | 10/280 | 129/18800 | 2.37E-05 | 0.0013854 | NLRP12/MNDA/LPL/MEFV/LILRA2/CX3CR1/TNF/CX3CL1/FFAR4/IL1R2 |
| GO:0032652 | regulation of interleukin-1 production | 10/280 | 129/18800 | 2.37E-05 | 0.0013854 | NLRP12/MNDA/LPL/MEFV/LILRA2/CX3CR1/TNF/CX3CL1/FFAR4/IL1R2 |
| GO:0007200 | phospholipase C-activating G protein-coupled receptor signaling pathway | 9/280 | 104/18800 | 2.53E-05 | 0.0014308 | FPR2/FPR1/EDN1/EDNRB/C5AR2/F2RL3/CX3CR1/CHRM1/FFAR4 |
| GO:0050804 | modulation of chemical synaptic transmission | 19/280 | 429/18800 | 2.54E-05 | 0.0014308 | GRIA1/IL1B/ARC/ADRB2/NRG3/HAP1/CA2/WNT7A/EDN1/SLC6A4/FAM107A/LILRB2/ADCY8/TNR/CX3CR1/TNF/PTGS2/CX3CL1/RIMS4 |
| GO:0051924 | regulation of calcium ion transport | 14/280 | 251/18800 | 2.56E-05 | 0.0014308 | HAP1/RAMP3/UBASH3B/NOS1/STC2/LILRA2/F2RL3/CCL4/PLA2G1B/LILRB2/PTGS2/CX3CL1/TRPC3/STAC |
| GO:0099177 | regulation of trans-synaptic signaling | 19/280 | 430/18800 | 2.62E-05 | 0.0014391 | GRIA1/IL1B/ARC/ADRB2/NRG3/HAP1/CA2/WNT7A/EDN1/SLC6A4/FAM107A/LILRB2/ADCY8/TNR/CX3CR1/TNF/PTGS2/CX3CL1/RIMS4 |
| GO:1990845 | adaptive thermogenesis | 11/280 | 159/18800 | 2.71E-05 | 0.00146 | ADRB2/ACADL/ADRB1/DBH/EPAS1/G0S2/IL18R1/FABP4/PRLR/SCD/FFAR4 |
| GO:0070374 | positive regulation of ERK1 and ERK2 cascade | 13/280 | 220/18800 | 2.74E-05 | 0.00146 | ICAM1/FPR2/RAMP3/NDRG4/IL1A/BMP2/BMPER/C5AR2/CCL4/CCL24/TNF/CX3CL1/FFAR4 |
| GO:0045860 | positive regulation of protein kinase activity | 18/280 | 396/18800 | 2.96E-05 | 0.0015534 | GPRC5D/IL1B/ADRB2/NRG3/SLC11A1/EDN1/BMP2/TAL1/HBEGF/PLA2G1B/EMP2/ADCY8/TNF/S100A12/RGCC/PRLR/NCF1/ELANE |
| GO:0032611 | interleukin-1 beta production | 9/280 | 110/18800 | 3.96E-05 | 0.0020234 | NLRP12/MNDA/LPL/MEFV/LILRA2/CX3CR1/TNF/CX3CL1/FFAR4 |
| GO:0032651 | regulation of interleukin-1 beta production | 9/280 | 110/18800 | 3.96E-05 | 0.0020234 | NLRP12/MNDA/LPL/MEFV/LILRA2/CX3CR1/TNF/CX3CL1/FFAR4 |
| GO:0031644 | regulation of nervous system process | 10/280 | 138/18800 | 4.24E-05 | 0.0020733 | ADRB2/WNT7A/IL1A/EDN1/MYRF/TMEM100/EDNRB/CST7/TNR/TNF |
| GO:0007171 | activation of transmembrane receptor protein tyrosine kinase activity | 4/280 | 14/18800 | 4.29E-05 | 0.0020733 | ADRB2/NRG3/TAL1/PRLR |
| GO:0002675 | positive regulation of acute inflammatory response | 5/280 | 27/18800 | 4.36E-05 | 0.0020733 | IL1B/ALOX5AP/FFAR2/TNF/PTGS2 |
| GO:0019221 | cytokine-mediated signaling pathway | 20/280 | 486/18800 | 4.39E-05 | 0.0020733 | IL1RL1/IL1B/IRF1/IL7R/IL1A/EDN1/LILRA2/IL18R1/CCL4/LILRA1/LILRB2/CX3CR1/CCL24/TNF/OASL/CX3CL1/TXK/TSLP/PRLR/IL1R2 |
| GO:0001885 | endothelial cell development | 7/280 | 64/18800 | 4.54E-05 | 0.0020733 | ICAM1/IL1B/MYADM/EDNRB/TNF/HEG1/COL22A1 |
| GO:0009620 | response to fungus | 7/280 | 64/18800 | 4.54E-05 | 0.0020733 | GNLY/S100A8/CX3CR1/CLEC4E/S100A12/ELANE/MPO |
| GO:0002456 | T cell mediated immunity | 9/280 | 112/18800 | 4.57E-05 | 0.0020733 | ICAM1/IL1B/PRF1/TBX21/IL7R/KLRD1/MYO1G/IL18R1/EMP2 |
| GO:0022408 | negative regulation of cell-cell adhesion | 12/280 | 199/18800 | 4.57E-05 | 0.0020733 | BMP6/MYADM/IRF1/UBASH3B/TBX21/ILDR2/BMP2/LRRC32/PAG1/LILRB2/TNR/RGCC |
| GO:0071674 | mononuclear cell migration | 12/280 | 199/18800 | 4.57E-05 | 0.0020733 | ICAM1/FPR2/NLRP12/TBX21/SERPINE1/MYO1G/CCL4/CX3CR1/CCL24/TNF/S100A12/CX3CL1 |
| GO:0051091 | positive regulation of DNA-binding transcription factor activity | 14/280 | 265/18800 | 4.65E-05 | 0.0020796 | IL1B/EDN1/BMP2/S100A8/IL18R1/PLA2G1B/ADCY8/CX3CR1/BEX1/TNF/S100A12/CX3CL1/RGCC/RTKN2 |
| GO:0051047 | positive regulation of secretion | 15/280 | 300/18800 | 4.70E-05 | 0.0020796 | IL1B/BMP6/UNC13D/TTN/IL1A/EDN1/BMP2/SLC6A4/EDNRB/S100A8/PLA2G1B/ADCY8/INHBA/RGCC/FFAR4 |
| GO:0034341 | response to interferon-gamma | 10/280 | 140/18800 | 4.79E-05 | 0.0020919 | IRF1/SLC11A1/EDN1/MEFV/CCL4/CCL24/TNF/CX3CL1/TXK/GBP4 |
| GO:0002687 | positive regulation of leukocyte migration | 10/280 | 141/18800 | 5.09E-05 | 0.0021449 | ICAM1/FPR2/IL1A/EDN1/SERPINE1/CCL4/CX3CR1/CCL24/TNF/CX3CL1 |
| GO:0035296 | regulation of tube diameter | 10/280 | 141/18800 | 5.09E-05 | 0.0021449 | ADRB2/NOS1/ADRB1/DBH/EDN1/SLC6A4/EDNRB/TNF/PTGS2/GJA5 |
| GO:0097746 | blood vessel diameter maintenance | 10/280 | 141/18800 | 5.09E-05 | 0.0021449 | ADRB2/NOS1/ADRB1/DBH/EDN1/SLC6A4/EDNRB/TNF/PTGS2/GJA5 |
| GO:0035150 | regulation of tube size | 10/280 | 142/18800 | 5.40E-05 | 0.0022514 | ADRB2/NOS1/ADRB1/DBH/EDN1/SLC6A4/EDNRB/TNF/PTGS2/GJA5 |
| GO:0032635 | interleukin-6 production | 11/280 | 172/18800 | 5.57E-05 | 0.0022705 | IL1B/IL17D/NLRP12/IL1A/LPL/C5AR2/LILRA2/LILRB2/TNF/CX3CL1/TSLP |
| GO:0032675 | regulation of interleukin-6 production | 11/280 | 172/18800 | 5.57E-05 | 0.0022705 | IL1B/IL17D/NLRP12/IL1A/LPL/C5AR2/LILRA2/LILRB2/TNF/CX3CL1/TSLP |
| GO:0050890 | cognition | 15/280 | 306/18800 | 5.89E-05 | 0.002372 | GRIA1/ARC/LRRN4/NDRG4/DBH/AFF2/SLC6A4/FAM107A/LILRB2/ADCY8/TNR/CX3CR1/TNF/CHRM1/PTGS2 |
| GO:0002443 | leukocyte mediated immunity | 19/280 | 457/18800 | 5.96E-05 | 0.0023738 | ICAM1/IL1B/TREM1/UNC13D/PRF1/TBX21/IL7R/KLRD1/DBH/SH2D1B/MYO1G/IL18R1/PLA2G1B/GZMB/EMP2/CX3CR1/ULBP2/TNF/ELANE |
| GO:0002825 | regulation of T-helper 1 type immune response | 5/280 | 29/18800 | 6.26E-05 | 0.002469 | IL1RL1/IL1B/SLC11A1/TBX21/IL18R1 |
| GO:0002673 | regulation of acute inflammatory response | 6/280 | 47/18800 | 6.65E-05 | 0.0025935 | IL1B/ALOX5AP/EDNRB/FFAR2/TNF/PTGS2 |
| GO:1903035 | negative regulation of response to wounding | 8/280 | 92/18800 | 6.91E-05 | 0.0026673 | UBASH3B/EDN1/SERPINE1/WFDC1/MYOZ1/TNR/TNF/THBD |
| GO:0051928 | positive regulation of calcium ion transport | 9/280 | 119/18800 | 7.35E-05 | 0.0028042 | HAP1/RAMP3/LILRA2/F2RL3/CCL4/PLA2G1B/CX3CL1/TRPC3/STAC |
| GO:0042311 | vasodilation | 6/280 | 48/18800 | 7.51E-05 | 0.0028358 | ADRB2/NOS1/ADRB1/EDNRB/TNF/GJA5 |
| GO:0070588 | calcium ion transmembrane transport | 15/280 | 314/18800 | 7.87E-05 | 0.0029336 | HAP1/MCOLN3/SLC24A4/RAMP3/CATSPER1/UBASH3B/NOS1/EDN1/EDNRB/GPM6A/F2RL3/CACNA2D2/CX3CL1/TRPC3/STAC |
| GO:0002831 | regulation of response to biotic stimulus | 16/280 | 351/18800 | 7.93E-05 | 0.0029336 | IL1B/BMP6/TSPAN32/FPR2/IRF1/MNDA/PGC/KLRD1/SH2D1B/LILRA2/FFAR2/FCN1/OASL/CX3CL1/TXK/NCF1 |
| GO:0002548 | monocyte chemotaxis | 7/280 | 70/18800 | 8.11E-05 | 0.0029718 | FPR2/SERPINE1/CCL4/CX3CR1/CCL24/S100A12/CX3CL1 |
| GO:0006690 | icosanoid metabolic process | 9/280 | 121/18800 | 8.36E-05 | 0.0030119 | IL1B/DPEP2/PLA2G4F/EDN1/ALOX5AP/PLA2G1B/HPGD/PTGS2/GGT1 |
| GO:0002833 | positive regulation of response to biotic stimulus | 11/280 | 180/18800 | 8.40E-05 | 0.0030119 | BMP6/FPR2/MNDA/PGC/KLRD1/SH2D1B/LILRA2/FFAR2/FCN1/OASL/TXK |
| GO:0002697 | regulation of immune effector process | 16/280 | 353/18800 | 8.47E-05 | 0.0030119 | ICAM1/IL1B/FCN3/UNC13D/TBX21/PGC/IL7R/KLRD1/SH2D1B/IL18R1/CX3CR1/FFAR2/TNF/FCN1/CD244/NCF1 |
| GO:0010721 | negative regulation of cell development | 11/280 | 181/18800 | 8.83E-05 | 0.0030989 | IL1B/CDKN2B/LRP4/WNT7A/IL1A/EDNRB/SPRY4/SEMA6A/TNR/SEMA3G/TNF |
| GO:0050766 | positive regulation of phagocytosis | 7/280 | 71/18800 | 8.89E-05 | 0.0030989 | IL1B/FPR2/FCN3/SLC11A1/TNF/FCN1/SIRPB1 |
| GO:0032642 | regulation of chemokine production | 8/280 | 97/18800 | 0.0001007 | 0.0034197 | IL1RL1/IL1B/LPL/MEFV/FFAR2/TNF/TSLP/ELANE |
| GO:0032755 | positive regulation of interleukin-6 production | 8/280 | 97/18800 | 0.0001007 | 0.0034197 | IL1B/IL17D/IL1A/LPL/LILRA2/LILRB2/TNF/TSLP |
| GO:0033674 | positive regulation of kinase activity | 19/280 | 476/18800 | 0.0001019 | 0.0034197 | GPRC5D/IL1B/ADRB2/NRG3/FPR2/SLC11A1/EDN1/BMP2/TAL1/HBEGF/PLA2G1B/EMP2/ADCY8/TNF/S100A12/RGCC/PRLR/NCF1/ELANE |
| GO:0034765 | regulation of ion transmembrane transport | 19/280 | 476/18800 | 0.0001019 | 0.0034197 | DPP6/ARC/ADRB2/HAP1/RAMP3/CATSPER1/UBASH3B/NOS1/EDN1/F2RL3/HECW2/KCNIP1/CACNA2D2/TNF/CX3CL1/KCNK17/CLIC3/PCSK9/STAC |
| GO:0032692 | negative regulation of interleukin-1 production | 6/280 | 51/18800 | 0.0001061 | 0.0034999 | NLRP12/MEFV/CX3CR1/CX3CL1/FFAR4/IL1R2 |
| GO:0045123 | cellular extravasation | 7/280 | 73/18800 | 0.0001062 | 0.0034999 | ICAM1/SELPLG/CX3CR1/TNF/SELL/CX3CL1/ELANE |
| GO:0002685 | regulation of leukocyte migration | 12/280 | 218/18800 | 0.0001097 | 0.0035838 | ICAM1/FPR2/IL1A/EDN1/SERPINE1/C5AR2/CCL4/CX3CR1/CCL24/TNF/CX3CL1/ELANE |
| GO:0001818 | negative regulation of cytokine production | 16/280 | 363/18800 | 0.0001171 | 0.0037925 | IL1RL1/CD83/SLC11A1/NLRP12/TBX21/LRRC32/MEFV/C5AR2/INHBA/CX3CR1/TNF/CX3CL1/RGCC/FFAR4/ELANE/IL1R2 |
| GO:0050901 | leukocyte tethering or rolling | 5/280 | 33/18800 | 0.0001193 | 0.0038266 | SELPLG/CX3CR1/TNF/SELL/ELANE |
| GO:0002827 | positive regulation of T-helper 1 type immune response | 4/280 | 18/18800 | 0.000125 | 0.0039411 | IL1B/SLC11A1/TBX21/IL18R1 |
| GO:0030730 | sequestering of triglyceride | 4/280 | 18/18800 | 0.000125 | 0.0039411 | ENPP1/IL1B/LPL/TNF |
| GO:0010876 | lipid localization | 18/280 | 446/18800 | 0.0001351 | 0.0042147 | ENPP1/IL1B/BMP6/STARD4/PLA2G4F/IL1A/EDN1/LPL/PLA2G1B/SLCO2A1/RBP2/INHBA/FFAR2/TNF/FABP4/SLC4A1/PCSK9/SFTPA1 |
| GO:0090066 | regulation of anatomical structure size | 19/280 | 487/18800 | 0.0001369 | 0.0042147 | ADRB2/MYADM/NOS1/ADRB1/IL7R/WNT7A/DBH/EDN1/SLC6A4/EDNRB/SEMA6A/LMOD3/TNR/SEMA3G/CCL24/PLEKHH2/TNF/PTGS2/GJA5 |
| GO:0061045 | negative regulation of wound healing | 7/280 | 76/18800 | 0.0001372 | 0.0042147 | UBASH3B/EDN1/SERPINE1/WFDC1/MYOZ1/TNF/THBD |
| GO:0006968 | cellular defense response | 6/280 | 54/18800 | 0.0001466 | 0.0043586 | GNLY/PRF1/MNDA/LILRB2/CX3CR1/NCF1 |
| GO:0010524 | positive regulation of calcium ion transport into cytosol | 6/280 | 54/18800 | 0.0001466 | 0.0043586 | HAP1/RAMP3/F2RL3/PLA2G1B/CX3CL1/TRPC3 |
| GO:0061900 | glial cell activation | 6/280 | 54/18800 | 0.0001466 | 0.0043586 | IL1B/FPR2/CST7/CX3CR1/TNF/CX3CL1 |
| GO:0071715 | icosanoid transport | 6/280 | 54/18800 | 0.0001466 | 0.0043586 | IL1B/PLA2G4F/IL1A/EDN1/PLA2G1B/SLCO2A1 |
| GO:0010522 | regulation of calcium ion transport into cytosol | 8/280 | 103/18800 | 0.0001534 | 0.004503 | HAP1/RAMP3/UBASH3B/NOS1/F2RL3/PLA2G1B/CX3CL1/TRPC3 |
| GO:0050806 | positive regulation of synaptic transmission | 10/280 | 161/18800 | 0.000154 | 0.004503 | ARC/HAP1/CA2/FAM107A/LILRB2/ADCY8/TNR/CX3CR1/TNF/PTGS2 |
| GO:0002695 | negative regulation of leukocyte activation | 11/280 | 193/18800 | 0.0001559 | 0.004503 | TSPAN32/IRF1/TBX21/MNDA/ILDR2/LRRC32/PAG1/CST7/LILRB2/INHBA/CX3CL1 |
| GO:0015732 | prostaglandin transport | 4/280 | 19/18800 | 0.0001565 | 0.004503 | IL1B/IL1A/EDN1/SLCO2A1 |
| GO:0006633 | fatty acid biosynthetic process | 10/280 | 162/18800 | 0.000162 | 0.0046266 | HACD1/IL1B/ACADL/PLA2G4F/EDN1/LPL/PLA2G1B/PTGS2/ACOT4/SCD |
| GO:0061041 | regulation of wound healing | 9/280 | 133/18800 | 0.0001725 | 0.0048874 | TNFRSF12A/UBASH3B/EDN1/SERPINE1/WFDC1/HBEGF/MYOZ1/TNF/THBD |
| GO:0048708 | astrocyte differentiation | 7/280 | 79/18800 | 0.0001751 | 0.0049218 | IL1B/FPR2/BMP2/TAL1/S100A8/TNF/LAMC3 |
| GO:0060284 | regulation of cell development | 19/280 | 500/18800 | 0.0001918 | 0.0053496 | IL1B/HAP1/CDKN2B/MYADM/UNC13D/LRP4/WNT7A/IL1A/BMP2/EDNRB/SPRY4/SEMA6A/LMOD3/DLL4/TNR/CX3CR1/SEMA3G/TNF/CX3CL1 |
| GO:0002274 | myeloid leukocyte activation | 12/280 | 232/18800 | 0.0001964 | 0.0054311 | IL1RL1/TSPAN32/FPR2/UNC13D/SLC11A1/LILRA2/CST7/CX3CR1/TNF/S100A12/CX3CL1/TSLP |
| GO:1903034 | regulation of response to wounding | 10/280 | 166/18800 | 0.0001977 | 0.0054311 | TNFRSF12A/UBASH3B/EDN1/SERPINE1/WFDC1/HBEGF/MYOZ1/TNR/TNF/THBD |
| GO:0050878 | regulation of body fluid levels | 16/280 | 382/18800 | 0.0002095 | 0.0057116 | TSPAN32/UBASH3B/NFE2/EDN1/SERPINE1/EDNRB/F2RL3/EMP2/CHRM1/HEG1/TXK/GJA5/MPIG6B/PRLR/THBD/SLC4A1 |
| GO:0045765 | regulation of angiogenesis | 15/280 | 345/18800 | 0.0002211 | 0.0059827 | IL1B/CEMIP2/IL1A/SERPINE1/BMPER/HYAL1/COL4A3/SEMA6A/EMP2/CX3CR1/ACVRL1/CCL24/TNF/RGCC/FUT1 |
| GO:0045444 | fat cell differentiation | 12/280 | 237/18800 | 0.000239 | 0.0063924 | ENPP1/ADRB2/SMAD6/ADRB1/LPL/BMP2/WIF1/FFAR2/TNF/PTGS2/FABP4/FFAR4 |
| GO:0031214 | biomineral tissue development | 10/280 | 170/18800 | 0.0002398 | 0.0063924 | ENPP1/BMP6/ADRB2/SLC24A4/KLF10/BMP2/PKDCC/ODAM/PTGS2/PHOSPHO1 |
| GO:1903532 | positive regulation of secretion by cell | 13/280 | 274/18800 | 0.0002507 | 0.0066048 | IL1B/BMP6/UNC13D/TTN/IL1A/EDN1/BMP2/SLC6A4/PLA2G1B/ADCY8/INHBA/RGCC/FFAR4 |
| GO:0060402 | calcium ion transport into cytosol | 10/280 | 171/18800 | 0.0002514 | 0.0066048 | HAP1/MCOLN3/SLC24A4/RAMP3/UBASH3B/NOS1/F2RL3/PLA2G1B/CX3CL1/TRPC3 |
| GO:0048640 | negative regulation of developmental growth | 8/280 | 111/18800 | 0.0002572 | 0.0067106 | ADRB2/ADRB1/STC2/SLC6A4/MYOZ1/SEMA6A/TNR/SEMA3G |
| GO:0110148 | biomineralization | 10/280 | 172/18800 | 0.0002634 | 0.0068232 | ENPP1/BMP6/ADRB2/SLC24A4/KLF10/BMP2/PKDCC/ODAM/PTGS2/PHOSPHO1 |
| GO:1901342 | regulation of vasculature development | 15/280 | 351/18800 | 0.0002659 | 0.0068387 | IL1B/CEMIP2/IL1A/SERPINE1/BMPER/HYAL1/COL4A3/SEMA6A/EMP2/CX3CR1/ACVRL1/CCL24/TNF/RGCC/FUT1 |
| GO:0010469 | regulation of signaling receptor activity | 10/280 | 173/18800 | 0.000276 | 0.0070478 | ARC/ADRB2/SLC24A4/RAMP3/EDN1/SERPINE1/HBEGF/TNF/NCF1/PCSK9 |
| GO:0032147 | activation of protein kinase activity | 9/280 | 142/18800 | 0.000282 | 0.0071495 | GPRC5D/ADRB2/NRG3/SLC11A1/TAL1/EMP2/ADCY8/RGCC/PRLR |
| GO:1904659 | glucose transmembrane transport | 8/280 | 113/18800 | 0.0002906 | 0.0073181 | ENPP1/IL1B/SLC2A3/EDN1/PLA2G1B/TNF/FFAR4/SLC2A14 |
| GO:0006959 | humoral immune response | 14/280 | 317/18800 | 0.0003046 | 0.0076171 | IL1B/MASP1/CD83/TREM1/FCN3/GNLY/SLC11A1/PGC/PLA2G1B/TNF/FCN1/S100A12/RGCC/ELANE |
| GO:0007188 | adenylate cyclase-modulating G protein-coupled receptor signaling pathway | 12/280 | 244/18800 | 0.0003118 | 0.0077428 | VIPR1/ADRB2/FPR2/RAMP3/FPR1/ADRB1/EDN1/ADCY8/CHRM1/PSAPL1/ADGRE3/S1PR5 |
| GO:0032731 | positive regulation of interleukin-1 beta production | 6/280 | 62/18800 | 0.000316 | 0.0077428 | NLRP12/MNDA/LPL/MEFV/LILRA2/TNF |
| GO:0060393 | regulation of pathway-restricted SMAD protein phosphorylation | 6/280 | 62/18800 | 0.000316 | 0.0077428 | BMP6/SMAD6/BMP2/BMPER/INHBA/ACVRL1 |
| GO:0010893 | positive regulation of steroid biosynthetic process | 4/280 | 23/18800 | 0.0003411 | 0.0082049 | BMP6/STARD4/IL1A/TNF |
| GO:0030728 | ovulation | 4/280 | 23/18800 | 0.0003411 | 0.0082049 | MMP19/INHBA/HPGD/PTGS2 |
| GO:0150077 | regulation of neuroinflammatory response | 5/280 | 41/18800 | 0.0003417 | 0.0082049 | IL1B/CST7/TNF/PTGS2/CX3CL1 |
| GO:0002699 | positive regulation of immune effector process | 12/280 | 248/18800 | 0.0003613 | 0.0085541 | IL1B/FCN3/UNC13D/TBX21/PGC/KLRD1/SH2D1B/IL18R1/FFAR2/TNF/FCN1/CD244 |
| GO:0008645 | hexose transmembrane transport | 8/280 | 117/18800 | 0.000368 | 0.0085541 | ENPP1/IL1B/SLC2A3/EDN1/PLA2G1B/TNF/FFAR4/SLC2A14 |
| GO:0031123 | RNA 3'-end processing | 8/280 | 117/18800 | 0.000368 | 0.0085541 | CT45A9/CT45A2/CT45A1/CT45A8/CT45A7/CT45A3/CT45A5/CT45A10 |
| GO:0032609 | interferon-gamma production | 8/280 | 117/18800 | 0.000368 | 0.0085541 | IL1RL1/IL1B/SLC11A1/IL18R1/INHBA/TNF/CD244/TXK |
| GO:0032649 | regulation of interferon-gamma production | 8/280 | 117/18800 | 0.000368 | 0.0085541 | IL1RL1/IL1B/SLC11A1/IL18R1/INHBA/TNF/CD244/TXK |
| GO:0050673 | epithelial cell proliferation | 17/280 | 443/18800 | 0.0003712 | 0.008574 | SDR16C5/BMP6/CDKN2B/WNT7A/BMP2/BMPER/EDNRB/HYAL1/C5AR2/COL4A3/DLL4/ODAM/ACVRL1/CCL24/TNF/RGCC/FUT1 |
| GO:1904064 | positive regulation of cation transmembrane transport | 9/280 | 148/18800 | 0.0003829 | 0.0087444 | ARC/ADRB2/HAP1/RAMP3/NOS1/EDN1/F2RL3/CX3CL1/STAC |
| GO:0030278 | regulation of ossification | 8/280 | 118/18800 | 0.0003898 | 0.0087444 | ENPP1/BMP6/ADRB2/LRP4/SMAD6/BMP2/PKDCC/PHOSPHO1 |
| GO:0071346 | cellular response to interferon-gamma | 8/280 | 118/18800 | 0.0003898 | 0.0087444 | IRF1/EDN1/CCL4/CCL24/TNF/CX3CL1/TXK/GBP4 |
| GO:0031589 | cell-substrate adhesion | 15/280 | 364/18800 | 0.0003907 | 0.0087444 | MYADM/UNC13D/SMAD6/ATRNL1/SERPINE1/MYO1G/SPRY4/FAM107A/EMP2/SPOCK2/ACVRL1/LAMC3/CX3CL1/FUT1/ITGA2B |
| GO:0042742 | defense response to bacterium | 15/280 | 364/18800 | 0.0003907 | 0.0087444 | TREM1/FPR2/GNLY/SLC11A1/PGC/IL7R/SERPINE1/S100A8/PLA2G1B/TNF/CLEC4E/S100A12/GBP4/ELANE/MPO |
| GO:0072330 | monocarboxylic acid biosynthetic process | 11/280 | 215/18800 | 0.0003954 | 0.0087967 | HACD1/IL1B/ACADL/STARD4/PLA2G4F/EDN1/LPL/PLA2G1B/PTGS2/ACOT4/SCD |
| GO:0015749 | monosaccharide transmembrane transport | 8/280 | 119/18800 | 0.0004126 | 0.009122 | ENPP1/IL1B/SLC2A3/EDN1/PLA2G1B/TNF/FFAR4/SLC2A14 |
| GO:0014002 | astrocyte development | 5/280 | 43/18800 | 0.0004284 | 0.0093572 | IL1B/FPR2/S100A8/TNF/LAMC3 |
| GO:0140353 | lipid export from cell | 5/280 | 43/18800 | 0.0004284 | 0.0093572 | IL1B/BMP6/IL1A/EDN1/INHBA |
| GO:0008217 | regulation of blood pressure | 10/280 | 183/18800 | 0.0004316 | 0.0093708 | ADRB2/NOS1/ADRB1/EDN1/EDNRB/EMP2/ACVRL1/TNF/PTGS2/GJA5 |
| GO:0060389 | pathway-restricted SMAD protein phosphorylation | 6/280 | 66/18800 | 0.0004445 | 0.0095713 | BMP6/SMAD6/BMP2/BMPER/INHBA/ACVRL1 |
| GO:0042063 | gliogenesis | 13/280 | 291/18800 | 0.0004461 | 0.0095713 | IL1B/CDKN2B/FPR2/BMP2/MYRF/TAL1/S100A8/ARHGEF10/CX3CR1/TNF/CHRM1/LAMC3/CX3CL1 |
| GO:0009953 | dorsal/ventral pattern formation | 7/280 | 92/18800 | 0.0004491 | 0.00958 | LRP4/SMAD6/WNT7A/EDN1/DISP1/DLL4/ACVRL1 |
| GO:0030282 | bone mineralization | 8/280 | 121/18800 | 0.0004614 | 0.0097697 | ENPP1/BMP6/ADRB2/KLF10/BMP2/PKDCC/PTGS2/PHOSPHO1 |
| GO:2001233 | regulation of apoptotic signaling pathway | 15/280 | 370/18800 | 0.0004634 | 0.0097697 | ICAM1/IL1B/ITPRIP/TNFRSF12A/IL1A/DBH/SERPINE1/S100A8/G0S2/INHBA/CX3CR1/TNF/PTGS2/CX3CL1/RTKN2 |
| GO:0014009 | glial cell proliferation | 5/280 | 44/18800 | 0.0004775 | 0.0098933 | IL1B/CDKN2B/TNF/CHRM1/CX3CL1 |
| GO:0045840 | positive regulation of mitotic nuclear division | 5/280 | 44/18800 | 0.0004775 | 0.0098933 | IL1B/IL1A/EDN1/TNF/RGCC |
| GO:2001239 | regulation of extrinsic apoptotic signaling pathway in absence of ligand | 5/280 | 44/18800 | 0.0004775 | 0.0098933 | IL1B/IL1A/INHBA/TNF/CX3CL1 |
| GO:1901224 | positive regulation of NIK/NF-kappaB signaling | 6/280 | 67/18800 | 0.0004822 | 0.0099343 | IL1B/NLRP12/EDN1/IL18R1/TNF/RTKN2 |
| GO:0007613 | memory | 8/280 | 122/18800 | 0.0004876 | 0.009988 | GRIA1/ARC/LRRN4/DBH/SLC6A4/ADCY8/CX3CR1/PTGS2 |
| GO:0040015 | negative regulation of multicellular organism growth | 3/280 | 11/18800 | 0.0004936 | 0.0100549 | ADRB2/ADRB1/STC2 |
| GO:0097191 | extrinsic apoptotic signaling pathway | 11/280 | 221/18800 | 0.0004986 | 0.0100998 | ICAM1/IL1B/ITPRIP/TNFRSF12A/IL1A/DBH/SERPINE1/G0S2/INHBA/TNF/CX3CL1 |
| GO:0051092 | positive regulation of NF-kappaB transcription factor activity | 9/280 | 154/18800 | 0.0005118 | 0.0103106 | IL1B/S100A8/IL18R1/PLA2G1B/CX3CR1/TNF/S100A12/CX3CL1/RTKN2 |
| GO:0042088 | T-helper 1 type immune response | 5/280 | 45/18800 | 0.0005307 | 0.0105153 | IL1RL1/IL1B/SLC11A1/TBX21/IL18R1 |
| GO:1900271 | regulation of long-term synaptic potentiation | 5/280 | 45/18800 | 0.0005307 | 0.0105153 | ARC/FAM107A/LILRB2/ADCY8/CX3CR1 |
| GO:2000273 | positive regulation of signaling receptor activity | 5/280 | 45/18800 | 0.0005307 | 0.0105153 | ARC/ADRB2/EDN1/HBEGF/NCF1 |
| GO:0003073 | regulation of systemic arterial blood pressure | 7/280 | 95/18800 | 0.0005455 | 0.0106923 | ADRB2/ADRB1/EDN1/EDNRB/EMP2/TNF/GJA5 |
| GO:0048661 | positive regulation of smooth muscle cell proliferation | 7/280 | 95/18800 | 0.0005455 | 0.0106923 | EDN1/HBEGF/HPGD/TNF/PTGS2/CX3CL1/ELANE |
| GO:0060401 | cytosolic calcium ion transport | 10/280 | 190/18800 | 0.0005791 | 0.0112902 | HAP1/MCOLN3/SLC24A4/RAMP3/UBASH3B/NOS1/F2RL3/PLA2G1B/CX3CL1/TRPC3 |
| GO:0032309 | icosanoid secretion | 5/280 | 46/18800 | 0.0005882 | 0.0113519 | IL1B/PLA2G4F/IL1A/EDN1/PLA2G1B |
| GO:0030856 | regulation of epithelial cell differentiation | 9/280 | 157/18800 | 0.0005886 | 0.0113519 | IL1B/BMP6/CDKN2B/IL1A/SERPINE1/TMEM100/ACVRL1/TNF/PRLR |
| GO:0003018 | vascular process in circulatory system | 12/280 | 263/18800 | 0.0006101 | 0.0116493 | BMP6/ADRB2/NOS1/ADRB1/SLC2A3/DBH/EDN1/SLC6A4/EDNRB/TNF/PTGS2/GJA5 |
| GO:0032722 | positive regulation of chemokine production | 6/280 | 70/18800 | 0.0006104 | 0.0116493 | IL1RL1/IL1B/LPL/FFAR2/TNF/TSLP |
| GO:0002532 | production of molecular mediator involved in inflammatory response | 7/280 | 97/18800 | 0.0006184 | 0.0116794 | IL17D/ALOX5AP/SERPINE1/MEFV/TNF/NCF1/IL1R2 |
| GO:2001237 | negative regulation of extrinsic apoptotic signaling pathway | 7/280 | 97/18800 | 0.0006184 | 0.0116794 | ICAM1/IL1B/ITPRIP/IL1A/SERPINE1/TNF/CX3CL1 |
| GO:0007611 | learning or memory | 12/280 | 264/18800 | 0.0006309 | 0.0118107 | GRIA1/ARC/LRRN4/NDRG4/DBH/AFF2/SLC6A4/LILRB2/ADCY8/TNR/CX3CR1/PTGS2 |
| GO:0050678 | regulation of epithelial cell proliferation | 15/280 | 382/18800 | 0.0006442 | 0.0118107 | BMP6/CDKN2B/WNT7A/BMP2/EDNRB/HYAL1/C5AR2/COL4A3/DLL4/ODAM/ACVRL1/CCL24/TNF/RGCC/FUT1 |
| GO:0002220 | innate immune response activating cell surface receptor signaling pathway | 4/280 | 27/18800 | 0.0006451 | 0.0118107 | KLRD1/LILRA2/FFAR2/FCN1 |
| GO:0001774 | microglial cell activation | 5/280 | 47/18800 | 0.0006504 | 0.0118107 | FPR2/CST7/CX3CR1/TNF/CX3CL1 |
| GO:0045601 | regulation of endothelial cell differentiation | 5/280 | 47/18800 | 0.0006504 | 0.0118107 | IL1B/BMP6/TMEM100/ACVRL1/TNF |
| GO:0045776 | negative regulation of blood pressure | 5/280 | 47/18800 | 0.0006504 | 0.0118107 | ADRB2/NOS1/ADRB1/TNF/GJA5 |
| GO:0001867 | complement activation, lectin pathway | 3/280 | 12/18800 | 0.0006509 | 0.0118107 | MASP1/FCN3/FCN1 |
| GO:0019932 | second-messenger-mediated signaling | 13/280 | 303/18800 | 0.0006514 | 0.0118107 | FPR2/SLC24A4/FPR1/NOS1/EDN1/TMEM100/EDNRB/MYOZ1/CCL4/CX3CR1/TNF/GUCY1A2/FFAR4 |
| GO:0001935 | endothelial cell proliferation | 10/280 | 194/18800 | 0.0006807 | 0.0122802 | BMP6/BMP2/BMPER/COL4A3/DLL4/ACVRL1/CCL24/TNF/RGCC/FUT1 |
| GO:0015908 | fatty acid transport | 7/280 | 99/18800 | 0.0006989 | 0.0124833 | IL1B/PLA2G4F/IL1A/EDN1/PLA2G1B/RBP2/FABP4 |
| GO:0050764 | regulation of phagocytosis | 7/280 | 99/18800 | 0.0006989 | 0.0124833 | IL1B/FPR2/FCN3/SLC11A1/TNF/FCN1/SIRPB1 |
| GO:0002253 | activation of immune response | 15/280 | 386/18800 | 0.0007165 | 0.012625 | IL1B/MASP1/FPR2/FCN3/FPR1/MNDA/KLRD1/MYO1G/C5AR2/LILRA2/FFAR2/FCN1/BTNL8/TXK/RGCC |
| GO:0006953 | acute-phase response | 5/280 | 48/18800 | 0.0007173 | 0.012625 | IL1B/IL1A/EDNRB/TNF/PTGS2 |
| GO:0061028 | establishment of endothelial barrier | 5/280 | 48/18800 | 0.0007173 | 0.012625 | ICAM1/IL1B/MYADM/EDNRB/TNF |
| GO:0042476 | odontogenesis | 8/280 | 130/18800 | 0.0007427 | 0.012968 | SLC24A4/LRP4/EDN1/SERPINE1/BMP2/INHBA/ODAM/SP6 |
| GO:0002758 | innate immune response-activating signal transduction | 4/280 | 28/18800 | 0.0007439 | 0.012968 | KLRD1/LILRA2/FFAR2/FCN1 |
| GO:0032732 | positive regulation of interleukin-1 production | 6/280 | 73/18800 | 0.0007636 | 0.0132484 | NLRP12/MNDA/LPL/MEFV/LILRA2/TNF |
| GO:0034767 | positive regulation of ion transmembrane transport | 9/280 | 163/18800 | 0.0007704 | 0.0133021 | ARC/ADRB2/HAP1/RAMP3/NOS1/EDN1/F2RL3/CX3CL1/STAC |
| GO:0034219 | carbohydrate transmembrane transport | 8/280 | 131/18800 | 0.000781 | 0.0134219 | ENPP1/IL1B/SLC2A3/EDN1/PLA2G1B/TNF/FFAR4/SLC2A14 |
| GO:0050873 | brown fat cell differentiation | 5/280 | 49/18800 | 0.0007892 | 0.0134975 | ADRB2/ADRB1/PTGS2/FABP4/FFAR4 |
| GO:0002430 | complement receptor mediated signaling pathway | 3/280 | 13/18800 | 0.0008369 | 0.0141139 | FPR2/FPR1/C5AR2 |
| GO:0032341 | aldosterone metabolic process | 3/280 | 13/18800 | 0.0008369 | 0.0141139 | BMP6/BMP2/EDNRB |
| GO:1900452 | regulation of long-term synaptic depression | 3/280 | 13/18800 | 0.0008369 | 0.0141139 | ARC/LILRB2/ADCY8 |
| GO:1904062 | regulation of cation transmembrane transport | 14/280 | 352/18800 | 0.000857 | 0.0143434 | DPP6/ARC/ADRB2/HAP1/RAMP3/UBASH3B/NOS1/EDN1/F2RL3/HECW2/KCNIP1/CX3CL1/PCSK9/STAC |
| GO:0002269 | leukocyte activation involved in inflammatory response | 5/280 | 50/18800 | 0.0008663 | 0.0143434 | FPR2/CST7/CX3CR1/TNF/CX3CL1 |
| GO:0006692 | prostanoid metabolic process | 5/280 | 50/18800 | 0.0008663 | 0.0143434 | IL1B/PLA2G4F/EDN1/HPGD/PTGS2 |
| GO:0006693 | prostaglandin metabolic process | 5/280 | 50/18800 | 0.0008663 | 0.0143434 | IL1B/PLA2G4F/EDN1/HPGD/PTGS2 |
| GO:0006809 | nitric oxide biosynthetic process | 6/280 | 75/18800 | 0.0008812 | 0.0145237 | IL1B/NOS1/EDN1/CX3CR1/TNF/PTGS2 |
| GO:0046394 | carboxylic acid biosynthetic process | 13/280 | 316/18800 | 0.0009587 | 0.0157303 | HACD1/IL1B/ACADL/STARD4/PLA2G4F/EDN1/ALOX5AP/LPL/PLA2G1B/PTGS2/ACOT4/GGT1/SCD |
| GO:0002064 | epithelial cell development | 10/280 | 203/18800 | 0.0009638 | 0.0157417 | ICAM1/IL1B/BMP6/MYADM/WNT7A/IL1A/EDNRB/TNF/HEG1/COL22A1 |
| GO:0060251 | regulation of glial cell proliferation | 4/280 | 30/18800 | 0.0009727 | 0.0157452 | IL1B/CDKN2B/TNF/CHRM1 |
| GO:0060292 | long-term synaptic depression | 4/280 | 30/18800 | 0.0009727 | 0.0157452 | GRIA1/ARC/LILRB2/ADCY8 |
| GO:0034308 | primary alcohol metabolic process | 7/280 | 105/18800 | 0.0009909 | 0.0159685 | SDR16C5/BMP6/CYP3A5/AWAT2/GPD1/BMP2/EDNRB |
| GO:0032729 | positive regulation of interferon-gamma production | 6/280 | 77/18800 | 0.0010122 | 0.0162191 | IL1B/SLC11A1/IL18R1/TNF/CD244/TXK |
| GO:0016053 | organic acid biosynthetic process | 13/280 | 318/18800 | 0.0010154 | 0.0162191 | HACD1/IL1B/ACADL/STARD4/PLA2G4F/EDN1/ALOX5AP/LPL/PLA2G1B/PTGS2/ACOT4/GGT1/SCD |
| GO:0006636 | unsaturated fatty acid biosynthetic process | 5/280 | 52/18800 | 0.0010373 | 0.0163935 | IL1B/PLA2G4F/EDN1/PTGS2/SCD |
| GO:0048660 | regulation of smooth muscle cell proliferation | 9/280 | 170/18800 | 0.0010378 | 0.0163935 | NDRG4/EDN1/BMP2/HBEGF/HPGD/TNF/PTGS2/CX3CL1/ELANE |
| GO:0042116 | macrophage activation | 7/280 | 106/18800 | 0.0010477 | 0.0163935 | IL1RL1/FPR2/SLC11A1/CST7/CX3CR1/TNF/CX3CL1 |
| GO:0008212 | mineralocorticoid metabolic process | 3/280 | 14/18800 | 0.0010534 | 0.0163935 | BMP6/BMP2/EDNRB |
| GO:0032306 | regulation of prostaglandin secretion | 3/280 | 14/18800 | 0.0010534 | 0.0163935 | IL1B/IL1A/EDN1 |
| GO:0032308 | positive regulation of prostaglandin secretion | 3/280 | 14/18800 | 0.0010534 | 0.0163935 | IL1B/IL1A/EDN1 |
| GO:0010827 | regulation of glucose transmembrane transport | 6/280 | 78/18800 | 0.0010831 | 0.0167838 | ENPP1/IL1B/EDN1/PLA2G1B/TNF/FFAR4 |
| GO:0061036 | positive regulation of cartilage development | 4/280 | 31/18800 | 0.0011038 | 0.0170317 | BMP6/BMP2/PKDCC/ACVRL1 |
| GO:0045785 | positive regulation of cell adhesion | 16/280 | 446/18800 | 0.0011213 | 0.0172275 | IL1B/CD83/MYADM/UNC13D/IL7R/FSTL3/IL1A/HYAL1/EMP2/LILRB2/SPOCK2/TNF/CX3CL1/FUT1/ELANE/SIRPB1 |
| GO:1901652 | response to peptide | 17/280 | 491/18800 | 0.0011647 | 0.0178197 | ENPP1/ICAM1/IL1B/ADRB2/FPR2/SLC24A4/RAMP3/CA2/EDN1/LPL/STC2/EDNRB/PLA2G1B/ADCY8/TNF/PTGS2/PCSK9 |
| GO:0048659 | smooth muscle cell proliferation | 9/280 | 173/18800 | 0.0011734 | 0.0178768 | NDRG4/EDN1/BMP2/HBEGF/HPGD/TNF/PTGS2/CX3CL1/ELANE |
| GO:0071695 | anatomical structure maturation | 11/280 | 246/18800 | 0.0012016 | 0.018015 | SLC24A4/EDN1/BMP2/EPAS1/GLDN/TAL1/EDNRB/CX3CR1/ACVRL1/PHOSPHO1/CX3CL1 |
| GO:0070482 | response to oxygen levels | 13/280 | 324/18800 | 0.0012023 | 0.018015 | NOS1/IL1A/EDN1/STC2/BMP2/EPAS1/SLC6A4/ALAS2/ACVRL1/TNF/PTGS2/GUCY1A2/RGCC |
| GO:0045089 | positive regulation of innate immune response | 8/280 | 140/18800 | 0.0012023 | 0.018015 | FPR2/MNDA/KLRD1/SH2D1B/LILRA2/FFAR2/FCN1/TXK |
| GO:0050768 | negative regulation of neurogenesis | 8/280 | 140/18800 | 0.0012023 | 0.018015 | IL1B/CDKN2B/LRP4/WNT7A/SEMA6A/TNR/SEMA3G/TNF |
| GO:0002449 | lymphocyte mediated immunity | 14/280 | 365/18800 | 0.0012115 | 0.0180777 | ICAM1/IL1B/UNC13D/PRF1/TBX21/IL7R/KLRD1/SH2D1B/MYO1G/IL18R1/GZMB/EMP2/ULBP2/TNF |
| GO:0001516 | prostaglandin biosynthetic process | 4/280 | 32/18800 | 0.0012469 | 0.0183787 | IL1B/PLA2G4F/EDN1/PTGS2 |
| GO:0046457 | prostanoid biosynthetic process | 4/280 | 32/18800 | 0.0012469 | 0.0183787 | IL1B/PLA2G4F/EDN1/PTGS2 |
| GO:1901889 | negative regulation of cell junction assembly | 4/280 | 32/18800 | 0.0012469 | 0.0183787 | IL1B/FAM107A/ACVRL1/TNF |
| GO:0023061 | signal release | 16/280 | 451/18800 | 0.0012583 | 0.0184423 | IL1B/BMP6/STX11/WNT7A/ILDR2/IL1A/EDN1/SLC6A4/SYN2/EDNRB/ADCY8/INHBA/FFAR2/TNF/RIMS4/FFAR4 |
| GO:0001666 | response to hypoxia | 12/280 | 286/18800 | 0.0012614 | 0.0184423 | NOS1/IL1A/EDN1/STC2/BMP2/EPAS1/SLC6A4/ALAS2/ACVRL1/TNF/PTGS2/RGCC |
| GO:0051090 | regulation of DNA-binding transcription factor activity | 16/280 | 452/18800 | 0.0012874 | 0.0187474 | ENPP1/IL1B/NLRP12/EDN1/BMP2/S100A8/IL18R1/PLA2G1B/ADCY8/CX3CR1/BEX1/TNF/S100A12/CX3CL1/RGCC/RTKN2 |
| GO:0010817 | regulation of hormone levels | 17/280 | 496/18800 | 0.0012987 | 0.0188138 | SDR16C5/IL1B/BMP6/CYP3A5/AWAT2/ILDR2/EDN1/STC2/DISP1/BMP2/EDNRB/SLCO4A1/ADCY8/INHBA/FFAR2/TNF/FFAR4 |
| GO:0032725 | positive regulation of granulocyte macrophage colony-stimulating factor production | 3/280 | 15/18800 | 0.0013023 | 0.0188138 | IL1B/IL17D/LILRA2 |
| GO:0030500 | regulation of bone mineralization | 6/280 | 81/18800 | 0.0013188 | 0.0189008 | ENPP1/BMP6/ADRB2/BMP2/PKDCC/PHOSPHO1 |
| GO:0046209 | nitric oxide metabolic process | 6/280 | 81/18800 | 0.0013188 | 0.0189008 | IL1B/NOS1/EDN1/CX3CR1/TNF/PTGS2 |
| GO:0015833 | peptide transport | 11/280 | 249/18800 | 0.0013242 | 0.0189041 | IL1B/CA2/ILDR2/EDN1/DISP1/S100A8/ADCY8/FFAR2/TNF/FFAR4/CLEC4M |
| GO:1904645 | response to amyloid-beta | 5/280 | 55/18800 | 0.001339 | 0.0190118 | ICAM1/ADRB2/FPR2/RAMP3/TNF |
| GO:0002768 | immune response-regulating cell surface receptor signaling pathway | 13/280 | 328/18800 | 0.0013423 | 0.0190118 | FPR2/FPR1/MNDA/KLRD1/MYO1G/C5AR2/LILRA2/LILRB2/FFAR2/FCN1/BTNL8/CLEC4E/TXK |
| GO:0071347 | cellular response to interleukin-1 | 7/280 | 111/18800 | 0.0013713 | 0.0191584 | IL1B/EDN1/HYAL1/CCL4/CCL24/CX3CL1/IL1R2 |
| GO:0034764 | positive regulation of transmembrane transport | 10/280 | 213/18800 | 0.0013847 | 0.0191584 | ARC/ADRB2/HAP1/RAMP3/NOS1/CA2/EDN1/F2RL3/CX3CL1/STAC |
| GO:0045940 | positive regulation of steroid metabolic process | 4/280 | 33/18800 | 0.0014024 | 0.0191584 | BMP6/STARD4/IL1A/TNF |
| GO:0051930 | regulation of sensory perception of pain | 4/280 | 33/18800 | 0.0014024 | 0.0191584 | IL1A/EDN1/TMEM100/EDNRB |
| GO:0051931 | regulation of sensory perception | 4/280 | 33/18800 | 0.0014024 | 0.0191584 | IL1A/EDN1/TMEM100/EDNRB |
| GO:0098801 | regulation of renal system process | 4/280 | 33/18800 | 0.0014024 | 0.0191584 | EDN1/EDNRB/EMP2/GJA5 |
| GO:1901099 | negative regulation of signal transduction in absence of ligand | 4/280 | 33/18800 | 0.0014024 | 0.0191584 | IL1B/IL1A/TNF/CX3CL1 |
| GO:2001240 | negative regulation of extrinsic apoptotic signaling pathway in absence of ligand | 4/280 | 33/18800 | 0.0014024 | 0.0191584 | IL1B/IL1A/TNF/CX3CL1 |
| GO:0042310 | vasoconstriction | 6/280 | 82/18800 | 0.0014054 | 0.0191584 | DBH/EDN1/SLC6A4/EDNRB/PTGS2/GJA5 |
| GO:2001057 | reactive nitrogen species metabolic process | 6/280 | 82/18800 | 0.0014054 | 0.0191584 | IL1B/NOS1/EDN1/CX3CR1/TNF/PTGS2 |
| GO:0010632 | regulation of epithelial cell migration | 12/280 | 290/18800 | 0.001419 | 0.0192702 | WNT7A/EDN1/BMPER/HYAL1/HBEGF/DLL4/EMP2/ACVRL1/TNF/PTGS2/RGCC/FUT1 |
| GO:0001936 | regulation of endothelial cell proliferation | 9/280 | 178/18800 | 0.0014308 | 0.019359 | BMP6/BMP2/COL4A3/DLL4/ACVRL1/CCL24/TNF/RGCC/FUT1 |
| GO:0061756 | leukocyte adhesion to vascular endothelial cell | 5/280 | 56/18800 | 0.0014527 | 0.0195521 | SELPLG/CX3CR1/TNF/SELL/ELANE |
| GO:0051235 | maintenance of location | 13/280 | 331/18800 | 0.0014559 | 0.0195521 | ENPP1/IL1B/HAP1/MCOLN3/STARD4/UBASH3B/NOS1/LPL/S100A8/F2RL3/FFAR2/TNF/CX3CL1 |
| GO:0001558 | regulation of cell growth | 15/280 | 415/18800 | 0.0014774 | 0.0197674 | ENPP1/PAPPA2/NRG3/EDN1/WFDC1/HYAL1/S100A8/HBEGF/SEMA6A/FAM107A/OSGIN1/INHBA/TNR/SEMA3G/ACVRL1 |
| GO:0051961 | negative regulation of nervous system development | 8/280 | 145/18800 | 0.0015048 | 0.0200599 | IL1B/CDKN2B/LRP4/WNT7A/SEMA6A/TNR/SEMA3G/TNF |
| GO:0006691 | leukotriene metabolic process | 4/280 | 34/18800 | 0.001571 | 0.0207429 | DPEP2/ALOX5AP/PLA2G1B/GGT1 |
| GO:0062013 | positive regulation of small molecule metabolic process | 8/280 | 146/18800 | 0.0015719 | 0.0207429 | IL1B/BMP6/STARD4/GPD1/NOS1/TNF/PTGS2/CD244 |
| GO:0007566 | embryo implantation | 5/280 | 57/18800 | 0.0015733 | 0.0207429 | IL1B/STC2/EMP2/PTGS2/PRLR |
| GO:0008228 | opsonization | 3/280 | 16/18800 | 0.0015853 | 0.0207429 | FCN3/FCN1/SFTPA1 |
| GO:0010810 | regulation of cell-substrate adhesion | 10/280 | 217/18800 | 0.0015903 | 0.0207429 | MYADM/UNC13D/SERPINE1/SPRY4/FAM107A/EMP2/SPOCK2/ACVRL1/CX3CL1/FUT1 |
| GO:0071222 | cellular response to lipopolysaccharide | 10/280 | 217/18800 | 0.0015903 | 0.0207429 | IL1B/BMP6/IL1A/SERPINE1/EDNRB/LILRA2/LILRB2/CX3CR1/TNF/CX3CL1 |
| GO:0021782 | glial cell development | 7/280 | 115/18800 | 0.0016825 | 0.0218285 | IL1B/FPR2/MYRF/S100A8/ARHGEF10/TNF/LAMC3 |
| GO:0021700 | developmental maturation | 12/280 | 296/18800 | 0.0016856 | 0.0218285 | SLC24A4/EDN1/BMP2/EPAS1/GLDN/TAL1/EDNRB/CX3CR1/ACVRL1/PHOSPHO1/CX3CL1/IGSF21 |
| GO:0046889 | positive regulation of lipid biosynthetic process | 6/280 | 85/18800 | 0.0016916 | 0.0218285 | IL1B/BMP6/STARD4/IL1A/TNF/PTGS2 |
| GO:0045766 | positive regulation of angiogenesis | 9/280 | 183/18800 | 0.0017319 | 0.0221906 | IL1B/IL1A/SERPINE1/BMPER/HYAL1/CX3CR1/ACVRL1/CCL24/FUT1 |
| GO:1904018 | positive regulation of vasculature development | 9/280 | 183/18800 | 0.0017319 | 0.0221906 | IL1B/IL1A/SERPINE1/BMPER/HYAL1/CX3CR1/ACVRL1/CCL24/FUT1 |
| GO:0007616 | long-term memory | 4/280 | 35/18800 | 0.0017531 | 0.022305 | GRIA1/ARC/LRRN4/ADCY8 |
| GO:0010737 | protein kinase A signaling | 4/280 | 35/18800 | 0.0017531 | 0.022305 | ADRB2/RAMP3/TTN/EDN1 |
| GO:0019915 | lipid storage | 6/280 | 86/18800 | 0.0017962 | 0.0227729 | ENPP1/IL1B/STARD4/LPL/FFAR2/TNF |
| GO:0036293 | response to decreased oxygen levels | 12/280 | 299/18800 | 0.0018337 | 0.0230395 | NOS1/IL1A/EDN1/STC2/BMP2/EPAS1/SLC6A4/ALAS2/ACVRL1/TNF/PTGS2/RGCC |
| GO:0002218 | activation of innate immune response | 5/280 | 59/18800 | 0.0018363 | 0.0230395 | MNDA/KLRD1/LILRA2/FFAR2/FCN1 |
| GO:0051785 | positive regulation of nuclear division | 5/280 | 59/18800 | 0.0018363 | 0.0230395 | IL1B/IL1A/EDN1/TNF/RGCC |
| GO:0042326 | negative regulation of phosphorylation | 14/280 | 382/18800 | 0.0018536 | 0.023143 | ENPP1/IL1B/CDKN2B/MYADM/UBASH3B/NLRP12/SMAD6/DUSP6/BMP2/SPRY4/INHBA/HEG1/FABP4/SLC4A1 |
| GO:0015718 | monocarboxylic acid transport | 7/280 | 117/18800 | 0.0018573 | 0.023143 | IL1B/PLA2G4F/IL1A/EDN1/PLA2G1B/SLCO2A1/TNF |
| GO:0002544 | chronic inflammatory response | 3/280 | 17/18800 | 0.0019039 | 0.0233232 | UNC13D/S100A8/TNF |
| GO:0032310 | prostaglandin secretion | 3/280 | 17/18800 | 0.0019039 | 0.0233232 | IL1B/IL1A/EDN1 |
| GO:0032604 | granulocyte macrophage colony-stimulating factor production | 3/280 | 17/18800 | 0.0019039 | 0.0233232 | IL1B/IL17D/LILRA2 |
| GO:0032645 | regulation of granulocyte macrophage colony-stimulating factor production | 3/280 | 17/18800 | 0.0019039 | 0.0233232 | IL1B/IL17D/LILRA2 |
| GO:0042481 | regulation of odontogenesis | 3/280 | 17/18800 | 0.0019039 | 0.0233232 | EDN1/BMP2/SP6 |
| GO:0030308 | negative regulation of cell growth | 9/280 | 186/18800 | 0.0019356 | 0.0236318 | ENPP1/WFDC1/HYAL1/SEMA6A/OSGIN1/INHBA/TNR/SEMA3G/ACVRL1 |
| GO:1903037 | regulation of leukocyte cell-cell adhesion | 13/280 | 344/18800 | 0.0020449 | 0.0248815 | IL1B/CD83/IRF1/TBX21/IL7R/ILDR2/IL1A/LRRC32/PAG1/LILRB2/TNF/ELANE/SIRPB1 |
| GO:0002819 | regulation of adaptive immune response | 9/280 | 188/18800 | 0.0020818 | 0.025246 | IL1RL1/IL1B/IRF1/SLC11A1/TBX21/IL7R/KLRD1/IL18R1/TNF |
| GO:0034113 | heterotypic cell-cell adhesion | 5/280 | 61/18800 | 0.0021302 | 0.025661 | IL1B/MYADM/GLDN/LILRB2/TNF |
| GO:0045428 | regulation of nitric oxide biosynthetic process | 5/280 | 61/18800 | 0.0021302 | 0.025661 | IL1B/EDN1/CX3CR1/TNF/PTGS2 |
| GO:0003230 | cardiac atrium development | 4/280 | 37/18800 | 0.0021605 | 0.0258543 | BMP2/DLL4/HEG1/GJA5 |
| GO:0045730 | respiratory burst | 4/280 | 37/18800 | 0.0021605 | 0.0258543 | SLC11A1/CD52/NCF1/MPO |
| GO:0097553 | calcium ion transmembrane import into cytosol | 8/280 | 154/18800 | 0.0021988 | 0.0262265 | HAP1/MCOLN3/SLC24A4/RAMP3/UBASH3B/NOS1/F2RL3/CX3CL1 |
| GO:0007599 | hemostasis | 10/280 | 227/18800 | 0.0022142 | 0.0263239 | TSPAN32/UBASH3B/NFE2/EDN1/SERPINE1/F2RL3/TXK/MPIG6B/THBD/SLC4A1 |
| GO:0031663 | lipopolysaccharide-mediated signaling pathway | 5/280 | 62/18800 | 0.0022893 | 0.0271019 | IL1B/BMP6/LILRA2/TNF/CX3CL1 |
| GO:0006869 | lipid transport | 14/280 | 391/18800 | 0.0022946 | 0.0271019 | IL1B/BMP6/STARD4/PLA2G4F/IL1A/EDN1/PLA2G1B/SLCO2A1/RBP2/INHBA/FABP4/SLC4A1/PCSK9/SFTPA1 |
| GO:0071219 | cellular response to molecule of bacterial origin | 10/280 | 229/18800 | 0.0023599 | 0.0277827 | IL1B/BMP6/IL1A/SERPINE1/EDNRB/LILRA2/LILRB2/CX3CR1/TNF/CX3CL1 |
| GO:0008643 | carbohydrate transport | 8/280 | 156/18800 | 0.0023826 | 0.0279594 | ENPP1/IL1B/SLC2A3/EDN1/PLA2G1B/TNF/FFAR4/SLC2A14 |
| GO:0010594 | regulation of endothelial cell migration | 10/280 | 230/18800 | 0.0024356 | 0.0283971 | WNT7A/EDN1/BMPER/DLL4/EMP2/ACVRL1/TNF/PTGS2/RGCC/FUT1 |
| GO:2001234 | negative regulation of apoptotic signaling pathway | 10/280 | 230/18800 | 0.0024356 | 0.0283971 | ICAM1/IL1B/ITPRIP/IL1A/SERPINE1/CX3CR1/TNF/PTGS2/CX3CL1/RTKN2 |
| GO:0070542 | response to fatty acid | 5/280 | 63/18800 | 0.0024569 | 0.028532 | EDN1/LPL/FFAR2/PTGS2/SCD |
| GO:0002764 | immune response-regulating signaling pathway | 16/280 | 482/18800 | 0.0024634 | 0.028532 | FPR2/IRF1/FPR1/MNDA/KLRD1/MYO1G/C5AR2/LILRA2/LILRB2/FFAR2/TNF/FCN1/OASL/BTNL8/CLEC4E/TXK |
| GO:0006909 | phagocytosis | 12/280 | 310/18800 | 0.0024708 | 0.028532 | IL1B/FPR2/FCN3/BIN2/UNC13D/SLC11A1/MYO1G/TNF/FCN1/ELANE/SIRPB1/SFTPA1 |
| GO:0001655 | urogenital system development | 13/280 | 352/18800 | 0.0024963 | 0.0287352 | BMP6/LRP4/SMAD6/COL4A4/GREB1L/BMP2/BMPER/EDNRB/COL4A3/HPGD/PRLR/PSAPL1/PCSK9 |
| GO:0045088 | regulation of innate immune response | 10/280 | 231/18800 | 0.0025132 | 0.0288385 | FPR2/IRF1/MNDA/KLRD1/SH2D1B/LILRA2/FFAR2/FCN1/TXK/NCF1 |
| GO:0046683 | response to organophosphorus | 7/280 | 124/18800 | 0.0025825 | 0.0294839 | IL1B/GPD1/SLC6A4/MMP19/PTGS2/TRPC3/THBD |
| GO:0045936 | negative regulation of phosphate metabolic process | 15/280 | 440/18800 | 0.0025939 | 0.0294839 | ENPP1/IL1B/CDKN2B/MYADM/UBASH3B/NLRP12/SMAD6/DUSP6/BMP2/SPRY4/INHBA/TNF/HEG1/FABP4/SLC4A1 |
| GO:0051960 | regulation of nervous system development | 15/280 | 440/18800 | 0.0025939 | 0.0294839 | IL1B/HAP1/CDKN2B/LRP4/WNT7A/BMP2/MYRF/CST7/SEMA6A/DLL4/TNR/CX3CR1/SEMA3G/TNF/CX3CL1 |
| GO:0080164 | regulation of nitric oxide metabolic process | 5/280 | 64/18800 | 0.0026333 | 0.0295206 | IL1B/EDN1/CX3CR1/TNF/PTGS2 |
| GO:0010563 | negative regulation of phosphorus metabolic process | 15/280 | 441/18800 | 0.0026501 | 0.0295206 | ENPP1/IL1B/CDKN2B/MYADM/UBASH3B/NLRP12/SMAD6/DUSP6/BMP2/SPRY4/INHBA/TNF/HEG1/FABP4/SLC4A1 |
| GO:0032332 | positive regulation of chondrocyte differentiation | 3/280 | 19/18800 | 0.0026541 | 0.0295206 | BMP6/PKDCC/ACVRL1 |
| GO:0035743 | CD4-positive, alpha-beta T cell cytokine production | 3/280 | 19/18800 | 0.0026541 | 0.0295206 | IL1B/TBX21/IL18R1 |
| GO:0045603 | positive regulation of endothelial cell differentiation | 3/280 | 19/18800 | 0.0026541 | 0.0295206 | BMP6/TMEM100/ACVRL1 |
| GO:0048245 | eosinophil chemotaxis | 3/280 | 19/18800 | 0.0026541 | 0.0295206 | CCL4/CCL24/CX3CL1 |
| GO:0060039 | pericardium development | 3/280 | 19/18800 | 0.0026541 | 0.0295206 | BMP2/DLL4/HEG1 |
| GO:0030858 | positive regulation of epithelial cell differentiation | 5/280 | 65/18800 | 0.0028186 | 0.0311592 | BMP6/CDKN2B/SERPINE1/TMEM100/ACVRL1 |
| GO:0072577 | endothelial cell apoptotic process | 5/280 | 65/18800 | 0.0028186 | 0.0311592 | ICAM1/SERPINE1/COL4A3/TNF/RGCC |
| GO:0051250 | negative regulation of lymphocyte activation | 8/280 | 161/18800 | 0.0028952 | 0.0319083 | IRF1/TBX21/MNDA/ILDR2/LRRC32/PAG1/LILRB2/INHBA |
| GO:0002703 | regulation of leukocyte mediated immunity | 10/280 | 236/18800 | 0.0029316 | 0.0322125 | ICAM1/IL1B/UNC13D/TBX21/IL7R/KLRD1/SH2D1B/IL18R1/CX3CR1/TNF |
| GO:0048167 | regulation of synaptic plasticity | 9/280 | 198/18800 | 0.0029498 | 0.0323146 | GRIA1/ARC/FAM107A/LILRB2/ADCY8/TNR/CX3CR1/PTGS2/CX3CL1 |
| GO:0010812 | negative regulation of cell-substrate adhesion | 5/280 | 66/18800 | 0.0030132 | 0.0326145 | SERPINE1/SPRY4/FAM107A/ACVRL1/CX3CL1 |
| GO:0038034 | signal transduction in absence of ligand | 5/280 | 66/18800 | 0.0030132 | 0.0326145 | IL1B/IL1A/INHBA/TNF/CX3CL1 |
| GO:0071677 | positive regulation of mononuclear cell migration | 5/280 | 66/18800 | 0.0030132 | 0.0326145 | FPR2/SERPINE1/CCL4/CX3CR1/TNF |
| GO:0097192 | extrinsic apoptotic signaling pathway in absence of ligand | 5/280 | 66/18800 | 0.0030132 | 0.0326145 | IL1B/IL1A/INHBA/TNF/CX3CL1 |
| GO:0035313 | wound healing, spreading of epidermal cells | 3/280 | 20/18800 | 0.0030883 | 0.0331451 | WNT7A/HBEGF/ACVRL1 |
| GO:0090026 | positive regulation of monocyte chemotaxis | 3/280 | 20/18800 | 0.0030883 | 0.0331451 | FPR2/SERPINE1/CX3CR1 |
| GO:0015711 | organic anion transport | 13/280 | 361/18800 | 0.0030988 | 0.0331451 | IL1B/SLC26A9/SLC22A10/PLA2G4F/SLC2A3/IL1A/EDN1/SLCO4A1/PLA2G1B/SLCO2A1/TNF/SLC4A1/SLC2A14 |
| GO:0050767 | regulation of neurogenesis | 13/280 | 361/18800 | 0.0030988 | 0.0331451 | IL1B/HAP1/CDKN2B/LRP4/WNT7A/BMP2/SEMA6A/DLL4/TNR/CX3CR1/SEMA3G/TNF/CX3CL1 |
| GO:0045429 | positive regulation of nitric oxide biosynthetic process | 4/280 | 41/18800 | 0.0031623 | 0.0336258 | IL1B/EDN1/TNF/PTGS2 |
| GO:1905314 | semi-lunar valve development | 4/280 | 41/18800 | 0.0031623 | 0.0336258 | SMAD6/BMP2/DLL4/GJA5 |
| GO:0045600 | positive regulation of fat cell differentiation | 5/280 | 67/18800 | 0.0032173 | 0.034111 | LPL/BMP2/WIF1/PTGS2/FFAR4 |
| GO:0009612 | response to mechanical stimulus | 9/280 | 201/18800 | 0.0032595 | 0.0344574 | IL1B/BMP6/IRF1/TTN/EDN1/SCEL/TNF/PTGS2/MPO |
| GO:0019722 | calcium-mediated signaling | 9/280 | 202/18800 | 0.0033682 | 0.0355031 | FPR2/SLC24A4/EDN1/TMEM100/EDNRB/MYOZ1/CCL4/CX3CR1/TNF |
| GO:0030501 | positive regulation of bone mineralization | 4/280 | 42/18800 | 0.0034548 | 0.0363106 | BMP6/ADRB2/BMP2/PKDCC |
| GO:0060291 | long-term synaptic potentiation | 6/280 | 98/18800 | 0.0034694 | 0.036358 | ARC/FAM107A/LILRB2/ADCY8/TNR/CX3CR1 |
| GO:0003085 | negative regulation of systemic arterial blood pressure | 3/280 | 21/18800 | 0.0035637 | 0.036815 | ADRB2/ADRB1/TNF |
| GO:0007252 | I-kappaB phosphorylation | 3/280 | 21/18800 | 0.0035637 | 0.036815 | CX3CR1/TNF/CX3CL1 |
| GO:0019370 | leukotriene biosynthetic process | 3/280 | 21/18800 | 0.0035637 | 0.036815 | ALOX5AP/PLA2G1B/GGT1 |
| GO:0032305 | positive regulation of icosanoid secretion | 3/280 | 21/18800 | 0.0035637 | 0.036815 | IL1B/IL1A/EDN1 |
| GO:0035809 | regulation of urine volume | 3/280 | 21/18800 | 0.0035637 | 0.036815 | EDN1/EDNRB/SLC4A1 |
| GO:1902105 | regulation of leukocyte differentiation | 11/280 | 283/18800 | 0.0035793 | 0.0368711 | CD83/IRF1/UBASH3B/TBX21/IL7R/FSTL3/KLF10/TAL1/LILRB2/INHBA/TNF |
| GO:0019233 | sensory perception of pain | 6/280 | 99/18800 | 0.0036481 | 0.037473 | IL1A/EDN1/TMEM100/EDNRB/TNF/PTGS2 |
| GO:0044703 | multi-organism reproductive process | 9/280 | 205/18800 | 0.0037115 | 0.038017 | IL1B/EDN1/STC2/SLC6A4/EMP2/HPGD/PTGS2/PRLR/THBD |
| GO:0032691 | negative regulation of interleukin-1 beta production | 4/280 | 43/18800 | 0.0037652 | 0.0383502 | MEFV/CX3CR1/CX3CL1/FFAR4 |
| GO:1904407 | positive regulation of nitric oxide metabolic process | 4/280 | 43/18800 | 0.0037652 | 0.0383502 | IL1B/EDN1/TNF/PTGS2 |
| GO:0002460 | adaptive immune response based on somatic recombination of immune receptors built from immunoglobulin superfamily domains | 13/280 | 370/18800 | 0.003815 | 0.0387482 | IL1RL1/ICAM1/IL1B/UNC13D/PRF1/SLC11A1/TBX21/IL7R/KLRD1/MYO1G/IL18R1/EMP2/TNF |
| GO:0002367 | cytokine production involved in immune response | 6/280 | 100/18800 | 0.0038336 | 0.0388284 | IL1B/TREM1/TBX21/IL18R1/FFAR2/TNF |
| GO:0014074 | response to purine-containing compound | 7/280 | 134/18800 | 0.0039741 | 0.0400595 | IL1B/GPD1/SLC6A4/MMP19/PTGS2/TRPC3/THBD |
| GO:0042886 | amide transport | 11/280 | 287/18800 | 0.0039772 | 0.0400595 | IL1B/CA2/ILDR2/EDN1/DISP1/S100A8/ADCY8/FFAR2/TNF/FFAR4/CLEC4M |
| GO:0071900 | regulation of protein serine/threonine kinase activity | 13/280 | 372/18800 | 0.0039911 | 0.0400875 | IL1B/ADRB2/CDKN2B/SERTAD1/EDN1/BMP2/SPRY4/PLA2G1B/TNF/HEG1/S100A12/RGCC/ELANE |
| GO:0070167 | regulation of biomineral tissue development | 6/280 | 101/18800 | 0.004026 | 0.0403265 | ENPP1/BMP6/ADRB2/BMP2/PKDCC/PHOSPHO1 |
| GO:0010829 | negative regulation of glucose transmembrane transport | 3/280 | 22/18800 | 0.0040814 | 0.0404383 | ENPP1/IL1B/TNF |
| GO:0032303 | regulation of icosanoid secretion | 3/280 | 22/18800 | 0.0040814 | 0.0404383 | IL1B/IL1A/EDN1 |
| GO:0046885 | regulation of hormone biosynthetic process | 3/280 | 22/18800 | 0.0040814 | 0.0404383 | BMP6/STC2/BMP2 |
| GO:0071902 | positive regulation of protein serine/threonine kinase activity | 9/280 | 208/18800 | 0.0040817 | 0.0404383 | IL1B/ADRB2/EDN1/BMP2/PLA2G1B/TNF/S100A12/RGCC/ELANE |
| GO:1904427 | positive regulation of calcium ion transmembrane transport | 5/280 | 71/18800 | 0.0041341 | 0.0407341 | HAP1/RAMP3/F2RL3/CX3CL1/STAC |
| GO:2001259 | positive regulation of cation channel activity | 5/280 | 71/18800 | 0.0041341 | 0.0407341 | ARC/ADRB2/HAP1/EDN1/STAC |
| GO:0034612 | response to tumor necrosis factor | 10/280 | 249/18800 | 0.0042836 | 0.0420927 | GPD1/EDN1/HYAL1/CCL4/AFF3/CCL24/TNF/PTGS2/FABP4/CX3CL1 |
| GO:0001503 | ossification | 14/280 | 420/18800 | 0.0043421 | 0.0425526 | ENPP1/BMP6/ADRB2/LRP4/SMAD6/FSTL3/KLF10/BMP2/PKDCC/RANBP3L/TNF/PTGS2/PHOSPHO1/FFAR4 |
| GO:0050810 | regulation of steroid biosynthetic process | 5/280 | 72/18800 | 0.0043896 | 0.0429024 | BMP6/STARD4/IL1A/BMP2/TNF |
| GO:0110149 | regulation of biomineralization | 6/280 | 103/18800 | 0.0044321 | 0.0431778 | ENPP1/BMP6/ADRB2/BMP2/PKDCC/PHOSPHO1 |
| GO:1904646 | cellular response to amyloid-beta | 4/280 | 45/18800 | 0.0044416 | 0.0431778 | ICAM1/ADRB2/FPR2/TNF |
| GO:0002822 | regulation of adaptive immune response based on somatic recombination of immune receptors built from immunoglobulin superfamily domains | 8/280 | 173/18800 | 0.0044766 | 0.0433209 | IL1RL1/IL1B/SLC11A1/TBX21/IL7R/KLRD1/IL18R1/TNF |
| GO:0050679 | positive regulation of epithelial cell proliferation | 9/280 | 211/18800 | 0.0044802 | 0.0433209 | BMP6/WNT7A/BMP2/HYAL1/C5AR2/ODAM/ACVRL1/CCL24/TNF |
| GO:0072677 | eosinophil migration | 3/280 | 23/18800 | 0.0046424 | 0.0446863 | CCL4/CCL24/CX3CL1 |
| GO:0019218 | regulation of steroid metabolic process | 6/280 | 104/18800 | 0.0046461 | 0.0446863 | BMP6/ACADL/STARD4/IL1A/BMP2/TNF |
| GO:0044706 | multi-multicellular organism process | 9/280 | 213/18800 | 0.0047623 | 0.0456829 | IL1B/EDN1/STC2/SLC6A4/EMP2/HPGD/PTGS2/PRLR/THBD |
| GO:0046890 | regulation of lipid biosynthetic process | 8/280 | 175/18800 | 0.0047946 | 0.0458709 | IL1B/BMP6/ACADL/STARD4/IL1A/BMP2/TNF/PTGS2 |
| GO:0001974 | blood vessel remodeling | 4/280 | 46/18800 | 0.0048086 | 0.0458843 | DBH/EPAS1/DLL4/ACVRL1 |
| GO:0003007 | heart morphogenesis | 10/280 | 254/18800 | 0.0049186 | 0.0468103 | NDRG4/TTN/SMAD6/EDN1/BMP2/TMEM100/DLL4/ACVRL1/HEG1/GJA5 |
| GO:0060840 | artery development | 6/280 | 106/18800 | 0.0050968 | 0.0482534 | SMAD6/EDN1/DLL4/HPGD/ACVRL1/GJA5 |
| GO:1903707 | negative regulation of hemopoiesis | 6/280 | 106/18800 | 0.0050968 | 0.0482534 | IL17D/IRF1/UBASH3B/TBX21/FSTL3/INHBA |
| GO:0071216 | cellular response to biotic stimulus | 10/280 | 256/18800 | 0.0051924 | 0.0487916 | IL1B/BMP6/IL1A/SERPINE1/EDNRB/LILRA2/LILRB2/CX3CR1/TNF/CX3CL1 |
| GO:0002228 | natural killer cell mediated immunity | 5/280 | 75/18800 | 0.0052237 | 0.0487916 | UNC13D/KLRD1/SH2D1B/GZMB/ULBP2 |
| GO:0050848 | regulation of calcium-mediated signaling | 5/280 | 75/18800 | 0.0052237 | 0.0487916 | SLC24A4/TMEM100/MYOZ1/CCL4/TNF |
| GO:0070555 | response to interleukin-1 | 7/280 | 141/18800 | 0.0052435 | 0.0487916 | IL1B/EDN1/HYAL1/CCL4/CCL24/CX3CL1/IL1R2 |
| GO:0002922 | positive regulation of humoral immune response | 3/280 | 24/18800 | 0.0052479 | 0.0487916 | IL1B/PGC/TNF |
| GO:0019835 | cytolysis | 3/280 | 24/18800 | 0.0052479 | 0.0487916 | PRF1/GZMB/GZMH |
| GO:0048143 | astrocyte activation | 3/280 | 24/18800 | 0.0052479 | 0.0487916 | IL1B/FPR2/TNF |
| GO:0001933 | negative regulation of protein phosphorylation | 12/280 | 341/18800 | 0.0052851 | 0.0490123 | ENPP1/IL1B/CDKN2B/MYADM/UBASH3B/NLRP12/SMAD6/DUSP6/BMP2/SPRY4/HEG1/FABP4 |
| GO:0003015 | heart process | 10/280 | 257/18800 | 0.0053337 | 0.0492124 | RAMP3/TTN/NOS1/ADRB1/EDN1/EPAS1/EDNRB/HBEGF/TNF/GJA5 |
| GO:0007229 | integrin-mediated signaling pathway | 6/280 | 107/18800 | 0.0053338 | 0.0492124 | TSPAN32/LAMA3/EMP2/TXK/MPIG6B/ITGA2B |
| GO:0062012 | regulation of small molecule metabolic process | 12/280 | 342/18800 | 0.0054066 | 0.0497575 | ENPP1/IL1B/BMP6/ACADL/STARD4/GPD1/NOS1/BMP2/TNF/PTGS2/CD244/SLC4A1 |
