# Supplementary material for: Integrated bulk and single-cell RNA-sequencing reveals SPOCK2 as a novel biomarker gene in the development of congenital pulmonary airway malformation
Source: Respir Res. 2023 May 10;24:127. doi: 10.1186/s12931-023-02436-z (PMC10170809; doi:10.1186/s12931-023-02436-z)
Supplement: Supplementary file 4 — Additional file 4: table S4 GO enrichment analysis (molecular function, MF) of up-regulated and down-regulated genes [file 12931_2023_2436_MOESM4_ESM.docx]

**Table S4a GO enrichment analysis (molecular function, MF) of up-regulated genes**

| **ID** | **Description** | **Gene Ratio** | **Bg Ratio** | **P-value** | **FDR** | **Gene ID** |
| --- | --- | --- | --- | --- | --- | --- |
| GO:0003823 | antigen binding | 65/985 | 174/18410 | 6.10E-38 | 4.52E-35 | IGHV1-18/IGHV3-23/IGKV3-20/IGHV2-26/IGHV2-5/IGLV3-19/IGLC2/IGHV3-15/IGKV3-15/IGLV6-57/IGKV1D-33/IGHV4-39/IGLL5/IGHG1/IGHG4/IGLV1-40/IGHV3-48/IGLV3-21/IGKV2D-28/IGHG3/IGKV1-5/IGHV3-33/IGLV3-1/IGHV4-61/IGKC/IGHV3-11/IGLV2-11/IGHV4-34/IGKV1-16/IGKV2-30/IGHA2/IGLV2-23/IGHV3-43/IGLC3/IGHV3-74/IGKV1D-39/IGHV1-3/IGLV1-47/IGHV6-1/IGHV1-69/IGLV1-44/IGHV3-13/JCHAIN/IGHV3-21/IGHV5-51/IGKV1-17/IGLV1-51/IGHA1/IGHV3-64/IGLV3-25/IGHG2/IGHV4-59/IGKV4-1/IGHV3-30/PLG/IGKV3D-11/IGHV3-7/IGHV1OR15-1/IGLC1/IGHV3-49/IGLV2-14/IGKV1-39/IGLC6/IGHV3-66/IGKV5-2 |
| GO:0034987 | immunoglobulin receptor binding | 39/985 | 80/18410 | 1.57E-28 | 5.83E-26 | IGHV1-18/IGHV3-23/IGHV2-26/IGHV2-5/IGLC2/IGHV3-15/IGHV4-39/IGLL5/IGHG1/IGHG4/IGHV3-48/IGHG3/IGHV3-33/IGHV4-61/IGKC/IGHV3-11/IGHV4-34/IGHA2/IGHV3-43/IGLC3/IGHV3-74/IGHV1-3/IGHV6-1/IGHV1-69/IGHV3-13/JCHAIN/IGHV3-21/IGHV5-51/IGHA1/IGHV3-64/IGHG2/IGHV4-59/IGHV3-30/IGHV3-7/IGHV1OR15-1/IGLC1/IGHV3-49/IGLC6/IGHV3-66 |
| GO:0008569 | minus-end-directed microtubule motor activity | 11/985 | 18/18410 | 2.19E-10 | 5.42E-08 | DNAH6/DNAH7/DNAH5/DNAH9/DNAH11/DNAH12/DNAH2/DNAH10/DNAH3/DYNC2H1/DNAH1 |
| GO:0003777 | microtubule motor activity | 18/985 | 67/18410 | 8.58E-09 | 1.59E-06 | DNAI2/DNAH6/DNAH7/DNAH5/KIF19/DYNLRB2/DNAH9/DNAH11/KIF24/DNAH12/KIF6/DNAH2/DNAH10/DNAH3/KIF21A/KIF1A/DYNC2H1/DNAH1 |
| GO:0051959 | dynein light intermediate chain binding | 10/985 | 27/18410 | 6.73E-07 | 9.98E-05 | DNAH6/DNAH7/DNAH5/DNAH9/DNAH11/DNAH2/DNAH10/DNAH3/DYNC2H1/DNAH1 |
| GO:0015631 | tubulin binding | 44/985 | 376/18410 | 9.14E-07 | 0.000113 | STMND1/PACRG/GAS2L2/KIF19/SPEF1/SAXO2/PIFO/C9orf24/DCDC1/EFHC2/SPAG8/SPAG6/PPP1R42/RP1/KIF24/TOGARAM2/TTLL9/CCDC170/KIF6/CETN2/CFAP157/DNAL1/CCDC181/MAP6/CCDC187/MAP1A/TTLL6/DLEC1/KIF21A/KIF1A/MAPRE3/TRPV4/IFT81/FAM161A/EML6/EFHC1/AGBL4/STRBP/SPATA4/TRAF3IP1/REEP2/TPPP3/KATNAL2/KIF26B |
| GO:0003774 | cytoskeletal motor activity | 20/985 | 111/18410 | 1.58E-06 | 0.0001575 | DNAI2/DNAH6/DNAH7/DNAH5/DNAI1/KIF19/DYNLRB2/DNAH9/DNAH11/KIF24/DNAH12/KIF6/DNAH2/DNAH10/DNAH3/KIF21A/KIF1A/DYNC2H1/MYO16/DNAH1 |
| GO:0045505 | dynein intermediate chain binding | 11/985 | 36/18410 | 1.70E-06 | 0.0001575 | DNAH6/DNAH7/DNAH5/DYNLRB2/DNAH9/DNAH11/DNAH2/DNAH10/DNAH3/DYNC2H1/DNAH1 |
| GO:0008017 | microtubule binding | 30/985 | 272/18410 | 0.0001423 | 0.0117349 | GAS2L2/KIF19/SPEF1/SAXO2/DCDC1/SPAG8/SPAG6/RP1/KIF24/TOGARAM2/CCDC170/KIF6/CETN2/CFAP157/CCDC181/MAP6/CCDC187/MAP1A/KIF21A/KIF1A/MAPRE3/TRPV4/FAM161A/EML6/STRBP/SPATA4/TRAF3IP1/REEP2/KATNAL2/KIF26B |
| GO:0004550 | nucleoside diphosphate kinase activity | 6/985 | 18/18410 | 0.0002459 | 0.0182485 | NME5/AK8/AK7/AK9/NME7/NME9 |
| GO:0004252 | serine-type endopeptidase activity | 21/985 | 174/18410 | 0.0004143 | 0.0279447 | MMP7/KLK13/HABP2/KLK11/KLK12/LTF/MMP1/MMP11/TMPRSS7/TMPRSS3/TMPRSS4/RHBDL2/KLK10/PCSK4/MMP10/PLG/KLK14/PRSS12/PRSS2/HTRA4/MMP12 |

**Table S4b GO enrichment analysis (molecular function, MF) of down-regulated genes**

| **ID** | **Description** | **Gene Ratio** | **Bg Ratio** | **P-value** | **FDR** | **Gene ID** |
| --- | --- | --- | --- | --- | --- | --- |
| GO:0140375 | immune receptor activity | 13/272 | 148/18410 | 3.11E-07 | 0.0001553 | IL1RL1/FPR2/FPR1/IL7R/KLRD1/C5AR2/LILRA2/IL18R1/LILRA1/LILRB2/CX3CR1/PRLR/IL1R2 |
| GO:0030246 | carbohydrate binding | 17/272 | 270/18410 | 5.93E-07 | 0.0001553 | ENPP1/LGALSL/FCN3/KLRD1/ATRNL1/SLC2A3/HK3/DBH/OLR1/FCN1/SIGLEC5/SELL/CLEC4E/SIGLEC11/P3H2/CLEC4M/SFTPA1 |
| GO:0031406 | carboxylic acid binding | 13/272 | 173/18410 | 1.85E-06 | 0.0003223 | NOS1/DBH/ALOX5AP/S100A8/PLA2G1B/RBP2/FCN1/SIGLEC5/FABP4/SIGLEC11/P3H2/FFAR4/PADI4 |
| GO:0005125 | cytokine activity | 13/272 | 235/18410 | 4.98E-05 | 0.0054219 | IL1B/BMP6/IL17D/WNT7A/IL1A/EDN1/BMP2/CCL4/INHBA/CCL24/TNF/CX3CL1/TSLP |
| GO:0030546 | signaling receptor activator activity | 20/272 | 496/18410 | 5.17E-05 | 0.0054219 | IL1B/BMP6/NRG3/IL17D/WNT7A/IL1A/EDN1/STC2/BMP2/TAL1/HBEGF/SEMA6A/CCL4/OSGIN1/INHBA/SEMA3G/CCL24/TNF/CX3CL1/TSLP |
| GO:0048018 | receptor ligand activity | 19/272 | 489/18410 | 0.0001293 | 0.0112957 | IL1B/BMP6/NRG3/IL17D/WNT7A/IL1A/EDN1/STC2/BMP2/HBEGF/SEMA6A/CCL4/OSGIN1/INHBA/SEMA3G/CCL24/TNF/CX3CL1/TSLP |
| GO:0050786 | RAGE receptor binding | 3/272 | 10/18410 | 0.0003545 | 0.0265333 | FPR1/S100A8/S100A12 |
| GO:0070696 | transmembrane receptor protein serine/threonine kinase binding | 4/272 | 25/18410 | 0.0004616 | 0.0302372 | BMP6/SMAD6/BMP2/INHBA |
| GO:0005178 | integrin binding | 9/272 | 156/18410 | 0.0005297 | 0.0302842 | ICAM1/IL1B/LAMA3/ESM1/COL4A3/EMP2/ICAM2/CX3CL1/ITGA2B |
| GO:0004875 | complement receptor activity | 3/272 | 12/18410 | 0.0006357 | 0.0302842 | FPR2/FPR1/C5AR2 |
| GO:0032396 | inhibitory MHC class I receptor activity | 3/272 | 12/18410 | 0.0006357 | 0.0302842 | LILRA2/LILRA1/LILRB2 |
| GO:0005504 | fatty acid binding | 5/272 | 49/18410 | 0.0007608 | 0.0332203 | ALOX5AP/S100A8/RBP2/FABP4/FFAR4 |
| GO:0033612 | receptor serine/threonine kinase binding | 4/272 | 29/18410 | 0.0008275 | 0.0333533 | BMP6/SMAD6/BMP2/INHBA |
| GO:0019955 | cytokine binding | 8/272 | 141/18410 | 0.0011944 | 0.0416697 | IL1RL1/LRRC32/IL18R1/CX3CR1/ACVRL1/PRLR/ELANE/IL1R2 |
| GO:0033293 | monocarboxylic acid binding | 6/272 | 81/18410 | 0.0012649 | 0.0416697 | ALOX5AP/S100A8/PLA2G1B/RBP2/FABP4/FFAR4 |
| GO:0036041 | long-chain fatty acid binding | 3/272 | 15/18410 | 0.0012724 | 0.0416697 | ALOX5AP/S100A8/FABP4 |
| GO:0008528 | G protein-coupled peptide receptor activity | 8/272 | 148/18410 | 0.0016273 | 0.0487439 | VIPR1/FPR2/RAMP3/FPR1/EDNRB/F2RL3/CX3CR1/SSTR3 |
| GO:0048306 | calcium-dependent protein binding | 6/272 | 87/18410 | 0.0018289 | 0.0487439 | MASP1/SLC24A4/NOS1/S100A8/S100A12/CLEC4M |
| GO:0008970 | phospholipase A1 activity | 3/272 | 17/18410 | 0.0018605 | 0.0487439 | PLA2G4F/LPL/PLA1A |
| GO:0032393 | MHC class I receptor activity | 3/272 | 17/18410 | 0.0018605 | 0.0487439 | LILRA2/LILRA1/LILRB2 |
