# Supplementary material for: Integrated bulk and single-cell RNA-sequencing reveals SPOCK2 as a novel biomarker gene in the development of congenital pulmonary airway malformation
Source: Respir Res. 2023 May 10;24:127. doi: 10.1186/s12931-023-02436-z (PMC10170809; doi:10.1186/s12931-023-02436-z)
Supplement: Supplementary file 5 — Additional file 5: table S5 GO enrichment analysis (cellular component, CC) of up-regulated and down-regulated genes [file 12931_2023_2436_MOESM5_ESM.docx]

**Table S5a GO enrichment analysis (cellular component, CC) of up-regulated genes**

| **ID** | **Description** | **Gene Ratio** | **Bg Ratio** | **P-value** | **FDR** | **Gene ID** |
| --- | --- | --- | --- | --- | --- | --- |
| GO:0019814 | immunoglobulin complex | 80/1016 | 167/19594 | 1.06E-57 | 4.97E-55 | IGKV2-28/PIGR/IGHV1-18/IGHV3-23/IGKV3-20/IGHV2-26/IGHV2-5/IGLV3-19/IGLC2/IGHV3-15/IGKV3-15/IGLV6-57/IGKV1D-33/IGHV4-39/IGLL5/IGHG1/IGHG4/IGLV1-40/IGHV3-48/IGLV3-21/IGKV2D-28/IGHG3/IGKV1-5/IGHV3-33/IGLV3-1/IGHV4-61/IGKC/IGHV3-11/IGLV2-11/IGHV4-34/IGKV1-16/IGKV2-30/IGHA2/IGKV3D-15/IGKJ1/IGLV2-18/IGLV2-23/IGHV3-43/IGLC3/IGHV3-74/IGKV1D-39/IGHV1-3/IGLV1-47/IGHV6-1/IGHV1-69/IGLV1-44/IGHV3-13/JCHAIN/IGHV3-21/IGHV5-51/IGKV1-17/IGLV1-51/IGKV2-24/IGHA1/IGHV3-64/IGLV3-25/IGKV2D-30/IGHG2/IGLV3-9/IGHV4-59/IGKV4-1/IGLV5-45/IGLV3-10/IGHV3-30/IGKV3D-11/IGHV3-7/IGKV3D-20/IGKV1-12/IGHV1OR15-1/IGLC1/IGHV3-49/IGLV2-14/IGLV4-69/IGKV1-39/IGLC6/IGKV2D-29/IGLV8-61/IGHV3-66/IGLV7-46/IGKV5-2 |
| GO:0031514 | motile cilium | 85/1016 | 227/19594 | 1.60E-50 | 3.76E-48 | ENKUR/NME5/TCTE1/CFAP61/DRC1/CCDC65/RSPH1/RSPH4A/TEKT1/AK8/PACRG/RSPH9/DRC7/AK7/ZBBX/DNAI2/IQCG/CATSPERD/CFAP43/CFAP52/DNAH5/DNAI1/TEKT4/MNS1/CFAP45/SPEF1/SAXO2/TEKT3/CFAP58/SPA17/TTC29/TEKT2/DRC3/ROPN1L/SPAG6/IQCD/CFAP65/ENO4/CABCOCO1/CFAP300/DNAH9/DNAH11/DNAJB13/CCDC39/CFAP73/TSGA10/CFAP100/TTLL9/CETN2/DNAH2/CCDC103/CFAP221/SPACA9/SPAG16/CFAP47/CFAP99/CCDC181/SPAG17/FLACC1/DNALI1/CFAP70/IQUB/CFAP69/MAK/IFT81/ROPN1B/NPHP1/DYNC2H1/IQCA1/SORD/SPEF2/CFAP206/AKAP3/CFAP161/IFT46/ROPN1/IFT172/EFCAB2/CATSPERE/CFAP44/FBXL13/IFT27/TACR1/CABYR/DNAH1 |
| GO:0005930 | axoneme | 61/1016 | 131/19594 | 3.07E-43 | 4.79E-41 | CFAP61/DRC1/CCDC65/RSPH4A/AK8/RSPH9/CCDC113/DNAI2/DNAH6/CFAP43/LCA5L/CFAP52/DNAH7/DNAH5/DNAI1/MNS1/KIF19/CFAP45/SPEF1/SAXO2/AKAP14/DRC3/EFHC2/SPAG6/RP1/DNAH9/DNAH11/DNAJB13/CCDC39/CFAP73/CFAP100/CCDC96/RPGRIP1L/DNAH2/CCDC103/HYDIN/CFAP221/SPAG16/DNAAF1/DNAL1/DNAH10/CFAP74/SPAG17/CFAP54/DNALI1/DNAH3/DZIP1L/CFAP70/CFAP46/MAK/EFHC1/TTC30B/DYNC2H1/SPATA4/CCDC40/CFAP206/TRAF3IP1/TTC30A/IFT172/LCA5/DNAH1 |
| GO:0097014 | ciliary plasm | 61/1016 | 132/19594 | 5.43E-43 | 6.35E-41 | CFAP61/DRC1/CCDC65/RSPH4A/AK8/RSPH9/CCDC113/DNAI2/DNAH6/CFAP43/LCA5L/CFAP52/DNAH7/DNAH5/DNAI1/MNS1/KIF19/CFAP45/SPEF1/SAXO2/AKAP14/DRC3/EFHC2/SPAG6/RP1/DNAH9/DNAH11/DNAJB13/CCDC39/CFAP73/CFAP100/CCDC96/RPGRIP1L/DNAH2/CCDC103/HYDIN/CFAP221/SPAG16/DNAAF1/DNAL1/DNAH10/CFAP74/SPAG17/CFAP54/DNALI1/DNAH3/DZIP1L/CFAP70/CFAP46/MAK/EFHC1/TTC30B/DYNC2H1/SPATA4/CCDC40/CFAP206/TRAF3IP1/TTC30A/IFT172/LCA5/DNAH1 |
| GO:0032838 | plasma membrane bounded cell projection cytoplasm | 66/1016 | 220/19594 | 1.33E-32 | 1.24E-30 | CFAP61/DRC1/CCDC65/RSPH4A/AK8/RSPH9/CCDC113/DNAI2/DNAH6/CFAP43/LCA5L/CFAP52/DNAH7/DNAH5/DNAI1/MNS1/KIF19/CFAP45/SPEF1/SAXO2/AKAP14/DRC3/EFHC2/SPAG6/RP1/DNAH9/DNAH11/DNAJB13/CCDC39/CFAP73/CFAP100/CCDC96/RPGRIP1L/DNAH2/CCDC103/HYDIN/CFAP221/SPAG16/DNAAF1/DNAL1/DNAH10/CFAP74/SPAG17/MAP1A/CFAP54/DNALI1/DNAH3/DZIP1L/CFAP70/KIF1A/CFAP46/MAK/EFHC1/TTC30B/DYNC2H1/AGBL4/SPATA4/CCDC40/CFAP206/TRAF3IP1/TTC30A/IFT172/GRIK2/LCA5/AP3B2/DNAH1 |
| GO:0042571 | immunoglobulin complex, circulating | 41/1016 | 77/19594 | 1.80E-32 | 1.40E-30 | PIGR/IGHV1-18/IGHV3-23/IGKV3-20/IGHV2-26/IGHV2-5/IGLC2/IGHV3-15/IGHV4-39/IGLL5/IGHG1/IGHG4/IGHV3-48/IGHG3/IGHV3-33/IGHV4-61/IGKC/IGHV3-11/IGHV4-34/IGHA2/IGHV3-43/IGLC3/IGHV3-74/IGHV1-3/IGHV6-1/IGHV1-69/IGHV3-13/JCHAIN/IGHV3-21/IGHV5-51/IGHA1/IGHV3-64/IGHG2/IGHV4-59/IGHV3-30/IGHV3-7/IGHV1OR15-1/IGLC1/IGHV3-49/IGLC6/IGHV3-66 |
| GO:0097729 | 9+2 motile cilium | 53/1016 | 149/19594 | 1.58E-30 | 1.05E-28 | ENKUR/NME5/TCTE1/RSPH1/AK8/PACRG/RSPH9/DNAI2/IQCG/CATSPERD/CFAP43/CFAP52/DNAH5/DNAI1/TEKT4/MNS1/CFAP45/SPEF1/SAXO2/TEKT3/CFAP58/SPA17/TTC29/DRC3/SPAG6/CFAP65/ENO4/CABCOCO1/DNAH9/DNAH11/DNAJB13/CCDC39/CETN2/DNAH2/CFAP221/SPACA9/SPAG16/CFAP47/CCDC181/FLACC1/DNALI1/CFAP70/CFAP69/IFT81/SPEF2/AKAP3/IFT172/EFCAB2/CATSPERE/IFT27/TACR1/CABYR/DNAH1 |
| GO:0099568 | cytoplasmic region | 67/1016 | 259/19594 | 8.34E-29 | 4.88E-27 | CFAP61/DRC1/CCDC65/RSPH4A/AK8/RSPH9/CCDC113/DNAI2/DNAH6/CFAP43/LCA5L/CFAP52/DNAH7/DNAH5/DNAI1/MNS1/KIF19/CFAP45/SPEF1/SAXO2/AKAP14/DRC3/EFHC2/SPAG6/RP1/DNAH9/DNAH11/DNAJB13/CCDC39/CFAP73/CFAP100/CCDC96/RPGRIP1L/DNAH2/CCDC103/HYDIN/CFAP221/SPAG16/DNAAF1/DNAL1/DNAH10/CFAP74/SPAG17/MAP1A/CFAP54/DNALI1/DNAH3/DZIP1L/CFAP70/KIF1A/CFAP46/MAK/EFHC1/TTC30B/DYNC2H1/AGBL4/SPATA4/CCDC40/CFAP206/TRAF3IP1/TTC30A/IFT172/GRIK2/LCA5/AP3B2/DNAH1/HAMP |
| GO:0036126 | sperm flagellum | 42/1016 | 136/19594 | 1.02E-21 | 5.29E-20 | ENKUR/NME5/TCTE1/RSPH1/AK8/PACRG/DNAI2/IQCG/CATSPERD/TEKT4/MNS1/SAXO2/TEKT3/CFAP58/SPA17/TTC29/DRC3/SPAG6/CFAP65/ENO4/CABCOCO1/DNAJB13/DNAH2/CFAP221/SPACA9/SPAG16/CFAP47/CCDC181/FLACC1/DNALI1/CFAP70/CFAP69/IFT81/SPEF2/AKAP3/IFT172/EFCAB2/CATSPERE/IFT27/TACR1/CABYR/DNAH1 |
| GO:0036064 | ciliary basal body | 42/1016 | 161/19594 | 1.18E-18 | 5.52E-17 | CCDC65/CFAP126/CCDC113/GAS2L2/SAXO2/PIFO/AGBL2/EFHC2/IQCD/USH1G/FANK1/CFAP100/TTLL9/CCDC170/CCDC96/CETN2/C5orf49/CFAP157/RPGRIP1L/SPACA9/BBOF1/WHRN/B9D1/TTLL6/C11orf97/MAPK15/DZIP1L/CFAP70/TTC26/IFT81/FAM161A/AGBL4/PPP1R32/CEP19/CFAP206/TRAF3IP1/IFT46/IFT172/MLF1/RABL2B/LCA5/CCDC178 |
| GO:0005858 | axonemal dynein complex | 14/1016 | 23/19594 | 4.91E-13 | 2.09E-11 | DRC1/CCDC65/DNAI2/DNAH6/DNAH7/DNAH5/DNAI1/DNAH9/DNAH2/CCDC103/DNAL1/DNAH3/CFAP70/DNAH1 |
| GO:0030286 | dynein complex | 20/1016 | 54/19594 | 9.82E-13 | 3.83E-11 | DRC1/CCDC65/DNAI2/DNAH6/DNAH7/DNAH5/DNAI1/DYNLRB2/DNAH9/DNAH11/DNAH12/DNAH2/CCDC103/DNAL1/DNAH10/DNALI1/DNAH3/CFAP70/DYNC2H1/DNAH1 |
| GO:0072562 | blood microparticle | 30/1016 | 147/19594 | 8.73E-11 | 3.14E-09 | CP/IGHV3-23/IGKV3-20/IGLC2/IGKV3-15/IGKV1D-33/IGHG1/IGHG4/IGLV3-21/IGKV2D-28/IGHG3/IGKV1-5/IGKC/IGKV2-30/IGHA2/IGLC3/IGLV1-47/PZP/IGHV3-13/JCHAIN/IGKV1-17/IGHA1/IGLV3-25/IGHG2/IGKV4-1/PLG/IGKV3D-11/IGHV3-7/IGLC1/IGKV1-39 |
| GO:0005874 | microtubule | 55/1016 | 435/19594 | 8.83E-10 | 2.95E-08 | TEKT1/DNAI2/DNAH6/DNAH7/DNAH5/DNAI1/GAS2L2/TUBA4B/KIF19/SPEF1/SAXO2/TEKT2/DCDC1/EFHC2/SPAG6/RP1/DYNLRB2/DNAH9/DNAH11/DCDC2B/KIF24/TOGARAM2/DNAH12/TTLL9/C4orf47/KIF6/RPGRIP1L/DNAH2/SPACA9/DNAL1/DNAH10/CCDC181/MAP6/SPAG17/MAP1A/TTLL6/DNAH3/KIF21A/KIF1A/MAPRE3/TRPV4/FAM161A/NEK2/EML6/TTC30B/DYNC2H1/CFAP206/REEP2/TTC30A/TPPP3/SAA1/KATNAL2/KIF26B/TUBB4B/DNAH1 |
| GO:0035869 | ciliary transition zone | 18/1016 | 69/19594 | 8.84E-09 | 2.76E-07 | RP1/TMEM231/USH1G/CETN2/RPGRIP1L/WHRN/B9D1/MAK/FAM161A/TMEM67/NPHP1/TRAF3IP1/TMEM107/TCTN1/CC2D2A/TMEM17/LCA5/TCTN2 |
| GO:0002177 | manchette | 9/1016 | 15/19594 | 9.87E-09 | 2.89E-07 | PACRG/IQCG/C9orf24/PPP1R42/CCDC181/LRGUK/MEIG1/STRBP/SPEF2 |
| GO:0009897 | external side of plasma membrane | 54/1016 | 455/19594 | 1.19E-08 | 3.28E-07 | CEACAM5/VTCN1/CD24/DNAI2/SPA17/HHLA2/IGHV1-18/IGHV3-23/IGHV2-26/IGHV2-5/IGLC2/IL5RA/IGHV3-15/IGHV4-39/IGLL5/IGHG1/IGHG4/IGHV3-48/IGHG3/ABCB1/IGHV3-33/IGHV4-61/IGKC/IGHV3-11/IGHV4-34/IGHA2/IGHV3-43/IGLC3/IGHV3-74/IGHV1-3/IGHV6-1/IGHV1-69/IGHV3-13/IGHV3-21/IGHV5-51/IGHA1/IL13RA2/IGHV3-64/ENPP3/IGHG2/IGHV4-59/ITGA11/IGHV3-30/PLG/IGHV3-7/IGHV1OR15-1/PRND/IGLC1/IGHV3-49/CD207/MS4A2/IGLC6/IGHV3-66/CRLF1 |
| GO:0120293 | dynein axonemal particle | 10/1016 | 20/19594 | 1.54E-08 | 4.01E-07 | DNAI2/DNAI1/DNAAF3/ZMYND10/DNAAF4/SPAG1/DNALI1/RUVBL1/RUVBL2/NME9 |
| GO:0030992 | intraciliary transport particle B | 9/1016 | 17/19594 | 4.36E-08 | 1.07E-06 | TTC26/IFT22/IFT81/TTC30B/TRAF3IP1/IFT46/TTC30A/IFT172/IFT27 |
| GO:0036038 | MKS complex | 8/1016 | 13/19594 | 5.19E-08 | 1.21E-06 | TMEM231/B9D1/TMEM67/TMEM107/TCTN1/CC2D2A/TMEM17/TCTN2 |
| GO:0005875 | microtubule associated complex | 27/1016 | 160/19594 | 5.48E-08 | 1.22E-06 | DRC1/CCDC65/DNAI2/DNAH6/DNAH7/DNAH5/DNAI1/KIF19/RP1/DYNLRB2/DNAH9/DNAH11/DNAH12/KIF6/DNAH2/CCDC103/DNAL1/DNAH10/MAP1A/DNALI1/DNAH3/KIF21A/CFAP70/KIF1A/DYNC2H1/KIF26B/DNAH1 |
| GO:0097228 | sperm principal piece | 11/1016 | 29/19594 | 1.01E-07 | 2.14E-06 | ENKUR/CATSPERD/SPA17/SPAG6/ENO4/IFT81/AKAP3/IFT172/EFCAB2/CATSPERE/IFT27 |
| GO:0030990 | intraciliary transport particle | 10/1016 | 24/19594 | 1.35E-07 | 2.76E-06 | TTC26/IFT22/IFT81/TTC30B/TRAF3IP1/TTC21A/IFT46/TTC30A/IFT172/IFT27 |
| GO:0036157 | outer dynein arm | 7/1016 | 11/19594 | 2.71E-07 | 5.29E-06 | DNAI2/DNAH5/DNAI1/DNAH9/CCDC103/DNAL1/CFAP70 |
| GO:0097542 | ciliary tip | 12/1016 | 47/19594 | 3.43E-06 | 6.42E-05 | SPEF1/RP1/DYNLRB2/TTC26/IFT22/IFT81/TTC30B/DYNC2H1/TRAF3IP1/IFT46/IFT172/IFT27 |
| GO:0097225 | sperm midpiece | 10/1016 | 38/19594 | 1.68E-05 | 0.0003026 | PACRG/CFAP58/CFAP65/CFAP69/IFT81/SPEF2/AKAP3/IFT172/IFT27/TACR1 |
| GO:0005881 | cytoplasmic microtubule | 13/1016 | 75/19594 | 0.0001149 | 0.0019923 | SAXO2/TOGARAM2/C4orf47/RPGRIP1L/SPACA9/MAPRE3/TRPV4/FAM161A/TTC30B/CFAP206/REEP2/TTC30A/SAA1 |
| GO:0032391 | photoreceptor connecting cilium | 9/1016 | 41/19594 | 0.0002039 | 0.0034077 | RP1/USH1G/CETN2/RPGRIP1L/WHRN/MAK/FAM161A/NPHP1/LCA5 |
| GO:0097546 | ciliary base | 9/1016 | 42/19594 | 0.0002477 | 0.0039969 | FAM183A/FANK1/SPACA9/C11orf97/DNALI1/CEP126/TTC26/TRAF3IP1/MOK |
| GO:0098878 | neurotransmitter receptor complex | 9/1016 | 45/19594 | 0.0004281 | 0.006678 | GRIN3B/GRIK5/SHISA6/CNIH2/GRIN1/GRIK2/HTR3E/SHISA8/VWC2L |
| GO:0008328 | ionotropic glutamate receptor complex | 8/1016 | 40/19594 | 0.0008876 | 0.0133995 | GRIN3B/GRIK5/SHISA6/CNIH2/GRIN1/GRIK2/SHISA8/VWC2L |
| GO:0099061 | integral component of postsynaptic density membrane | 9/1016 | 51/19594 | 0.0011124 | 0.0162695 | GRIK5/SHISA6/LRRC4/CNIH2/ERBB4/EFNB3/ADRA2A/EPHA7/CLSTN2 |
| GO:0045177 | apical part of cell | 37/1016 | 424/19594 | 0.0014249 | 0.0202073 | CEACAM5/SPTBN2/AQP6/CFAP126/AQP5/SPEF1/PROM1/ACY3/KCNK2/ZMYND10/CETN2/OXTR/PROM2/SLC44A4/SLC26A4/SLC22A4/CDH2/SLC23A1/F2RL2/ABCB1/TRPV4/DYNC2H1/EPB41L4B/CLDN1/ANXA13/MUC13/ECRG4/SLC15A1/MUC20/ATP12A/ENPP3/SLC4A11/USH1C/ATP4B/ATP6V1B1/AJAP1/HAMP |
| GO:0099146 | intrinsic component of postsynaptic density membrane | 9/1016 | 54/19594 | 0.0016925 | 0.0232966 | GRIK5/SHISA6/LRRC4/CNIH2/ERBB4/EFNB3/ADRA2A/EPHA7/CLSTN2 |
| GO:0001669 | acrosomal vesicle | 15/1016 | 124/19594 | 0.0019088 | 0.0255231 | ENKUR/TEKT3/SPAG8/SPAG6/CFAP65/MORN2/SPACA9/LRGUK/IQUB/RND2/TMEM190/AKAP3/PCSK4/SYT8/SV2B |
| GO:0016324 | apical plasma membrane | 32/1016 | 358/19594 | 0.0019862 | 0.02582 | CEACAM5/SPTBN2/AQP6/CFAP126/AQP5/SPEF1/PROM1/ACY3/KCNK2/ZMYND10/OXTR/PROM2/SLC44A4/SLC26A4/SLC22A4/CDH2/SLC23A1/F2RL2/ABCB1/TRPV4/CLDN1/ANXA13/MUC13/ECRG4/SLC15A1/MUC20/ATP12A/ENPP3/SLC4A11/ATP4B/ATP6V1B1/AJAP1 |
| GO:0005902 | microvillus | 12/1016 | 90/19594 | 0.0023186 | 0.0293272 | AQP5/SPEF1/ESPN/PROM1/FOXA1/OXTR/PROM2/FMN2/MUC20/USH1C/ATP6V1B1/MTTP |
| GO:0097730 | non-motile cilium | 18/1016 | 166/19594 | 0.00248 | 0.0305431 | RSPH9/PROM1/CERKL/RP1/USH1G/C4orf47/CETN2/RPGRIP1L/WHRN/TTLL6/DNAAF4/CFAP69/MAK/FAM161A/NPHP1/IFTAP/USH1C/LCA5 |
| GO:0097731 | 9+0 non-motile cilium | 15/1016 | 132/19594 | 0.0035157 | 0.041246 | PROM1/CERKL/RP1/USH1G/C4orf47/CETN2/RPGRIP1L/WHRN/TTLL6/MAK/FAM161A/NPHP1/IFTAP/USH1C/LCA5 |
| GO:0098992 | neuronal dense core vesicle | 4/1016 | 13/19594 | 0.0035253 | 0.041246 | KIF1A/CHGA/SYT5/PENK |

**Table S5b GO enrichment analysis (cellular component, CC) of down-regulated genes**

| **ID** | **Description** | **Gene Ratio** | **Bg Ratio** | **P-value** | **FDR** | **Gene ID** |
| --- | --- | --- | --- | --- | --- | --- |
| GO:0032039 | integrator complex | 8/291 | 27/19594 | 3.74E-09 | 1.12E-06 | CT45A9/CT45A2/CT45A1/CT45A8/CT45A7/CT45A3/CT45A5/CT45A10 |
| GO:0030667 | secretory granule membrane | 17/291 | 312/19594 | 4.66E-06 | 0.000701 | FPR2/FPR1/SLC11A1/SLC2A3/DBH/CEACAM3/PLA1A/LAMP3/MCEMP1/OLR1/LILRB2/SIGLEC5/SELL/ITGA2B/FCGR3B/ADGRE3/SIRPB1 |
| GO:0009897 | external side of plasma membrane | 20/291 | 455/19594 | 1.68E-05 | 0.0016869 | IL1RL1/ICAM1/CD83/IL7R/KLRD1/TRGC1/CX3CR1/ULBP2/TNF/FCN1/HEG1/BTNL8/CD244/CLEC4E/PRLR/ITGA2B/THBD/IGSF21/PCSK9/CLEC4M |
| GO:0044194 | cytolytic granule | 4/291 | 13/19594 | 3.07E-05 | 0.0019612 | GNLY/PRF1/GZMB/GZMH |
| GO:0101003 | ficolin-1-rich granule membrane | 7/291 | 61/19594 | 3.26E-05 | 0.0019612 | FPR2/FPR1/SLC11A1/SLC2A3/LILRB2/SIGLEC5/ADGRE3 |
| GO:0005581 | collagen trimer | 8/291 | 86/19594 | 4.18E-05 | 0.0020993 | FCN3/COL4A4/GLDN/COL12A1/COL4A3/FCN1/COL22A1/SFTPA1 |
| GO:0070821 | tertiary granule membrane | 7/291 | 73/19594 | 0.0001046 | 0.0036282 | FPR2/SLC11A1/SLC2A3/MCEMP1/OLR1/LILRB2/SIGLEC5 |
| GO:0101002 | ficolin-1-rich granule | 11/291 | 185/19594 | 0.0001051 | 0.0036282 | FPR2/BIN2/FPR1/SLC11A1/MNDA/SLC2A3/HK3/LILRB2/FCN1/SIGLEC5/ADGRE3 |
| GO:0045121 | membrane raft | 15/291 | 326/19594 | 0.0001165 | 0.0036282 | ICAM1/ARC/MYADM/LRP4/NOS1/SELPLG/SLC6A4/PAG1/EDNRB/OLR1/EMP2/ADCY8/TNR/TNF/PTGS2 |
| GO:0098857 | membrane microdomain | 15/291 | 327/19594 | 0.0001205 | 0.0036282 | ICAM1/ARC/MYADM/LRP4/NOS1/SELPLG/SLC6A4/PAG1/EDNRB/OLR1/EMP2/ADCY8/TNR/TNF/PTGS2 |
| GO:0062023 | collagen-containing extracellular matrix | 17/291 | 429/19594 | 0.0002501 | 0.0068424 | ICAM1/FCN3/ANGPTL6/ATRNL1/COL4A4/SERPINE1/COL12A1/S100A8/LAMA3/COL4A3/TNR/FCN1/FBN3/LAMC3/FIBCD1/P3H2/ELANE |
| GO:1905286 | serine-type peptidase complex | 3/291 | 11/19594 | 0.0004897 | 0.0122838 | FCN3/FCN1/THBD |
| GO:0098685 | Schaffer collateral - CA1 synapse | 6/291 | 72/19594 | 0.0007002 | 0.0158365 | ADRB1/WNT7A/SYN2/ADCY8/TNR/CHRM1 |
| GO:0001891 | phagocytic cup | 4/291 | 28/19594 | 0.0007366 | 0.0158365 | BIN2/MYO1G/TNF/CLEC4E |
| GO:0070820 | tertiary granule | 9/291 | 164/19594 | 0.0007911 | 0.0158738 | FPR2/FPR1/SLC11A1/SLC2A3/MCEMP1/OLR1/LILRB2/SIGLEC5/ADGRE3 |
| GO:0042599 | lamellar body | 3/291 | 17/19594 | 0.0018893 | 0.0337132 | LAMP3/NAPSA/SFTPA1 |
| GO:0030139 | endocytic vesicle | 13/291 | 342/19594 | 0.0019041 | 0.0337132 | GRIA1/ADRB2/GNLY/SLC11A1/IL7R/WNT7A/HBEGF/CLEC4E/FFAR4/NCF1/ELANE/MPO/SFTPA1 |
| GO:0005604 | basement membrane | 6/291 | 95/19594 | 0.0029347 | 0.0490744 | ATRNL1/COL4A4/LAMA3/COL4A3/LAMC3/P3H2 |
