# Supplementary material for: Integrated bulk and single-cell RNA-sequencing reveals SPOCK2 as a novel biomarker gene in the development of congenital pulmonary airway malformation
Source: Respir Res. 2023 May 10;24:127. doi: 10.1186/s12931-023-02436-z (PMC10170809; doi:10.1186/s12931-023-02436-z)
Supplement: Supplementary file 6 — Additional file 6: table S6 Gene list for the top 10 modules performed by iWGCNA [file 12931_2023_2436_MOESM6_ESM.docx]

**Table S6 Gene list for the top 10 modules performed by iWGCNA**

| **Module** | **Gene** |
| --- | --- |
| P15_I4_M3 | DEDD TP53BP2 IL1A KLHL23 HECW2 VIPR1 RPN1 LINC00968 PRDM11 ZDHHC5 PXN ANKLE2 PACS2 CHP1 PSTPIP1 IL16 RHBDF1 IL4R SEC14L1 ADGRE5 B3GNT8 ELMO2 |
| P6_I8_M3 | PLA2G5 MCOLN3 GNLY ATG9A PLA1A FGFBP2 ARHGAP24 GZMA RNF144B NPM1P10 ENPP1 GPER1 RAMP3 LINC02577 TMEM140 AC083862.1 DPP6 TBC1D2 SLC46A2 HACD1 PRF1 GRK5 LMO2 CHRM1 JPH4 GZMB SMAD6 LINC01169 LINC01996 TBX21 ITGA3 TMEM100 RGS9 HID1-AS1 MATK EVI5L ZNF331 APOL3 Z95114.3 BEX1 PLXNB3 RNU6-242P |
| P3_I23_M12 | NECAP2 PCSK9 LINC02785 SCAMP3 EVA1A DNPEP PPARG MAPKAPK3 EFCC1 QDPR SNX25 FGFR4 C2 PBX2 ILRUN WNT2 DOK2 FABP4 CA2 OR2S1P FGD3 SFTPA2 SFTPA1 STN1 TOLLIP CD44 TMEM109 EHD1 LINC02367 OLR1 WIF1 DRAM1 ALDH2 GPR65 RASGRF1 N4BP1 DOK4 TNFSF13 MMP28 FZD2 TBX2 GAA CABLES1 NPC1 MAPK4 CCBE1 S1PR4 TINCR MCEMP1 SIGLEC5 SMIM25 LRRC3 GUCD1 VSIG4 AC236972.3 |
| P1_I60_M11 | HEYL ZFYVE9 ST6GALNAC5 FAM89A MATN3 AC092667.1 EPB41L5 FZD5 SLC19A3 WNT7A MGLL ARAP3 FLT4 AL355499.1 STX11 DPYSL2 ADCY8 FAM219A GPSM1 AL590226.1 SPOCK2 MYRF PLCB3 HDAC7 LIN7A TBX5-AS1 C12orf49 OASL SPRYD7 NDRG2 INF2 GLDN ARL4D SRSF2 AC004490.1 KANK3 S1PR5 HSPA12B RIN2 CCM2L EMID1 MGAT3 |
| P14_I5_M2 | CACNA1S AL591686.2 LRRTM4 PPM1L AC147067.2 ADRB2 MEST AL135841.1 ADAMTSL2 MYOZ1 CCND1 GPRC5A TBX5 PRIMA1 CSPG4 CBFA2T3 HMGN2P15 AC245884.11 CDC25B BCRP3 |
| P2_I41_M13 | CAMTA1 SLC5A9 AL353807.5 CHML SOWAHC MYO7B OXSR1 UBA7 ARHGEF26 ARAP2 FRAS1 FAM13A-AS1 NPNT TRPC3 CLIC5 ADGRF5 ADGRG6 BMPER DENND2A RAB11FIP1 UNC13B NEBL LRP4 OVCH1 RBMS2 AC027288.1 VSIG10 ADGRD1 USP12 LMO7 AL137140.1 PTPN21 SLC24A4 PAQR5 LINC02185 SPN FENDRR EZH1 NMT1 C17orf67 TBX2-AS1 ANKRD29 |
| P3_I23_M10 | MTHFR CTPS1 SLC25A44 RTN4 SESTD1 STAT4 LANCL1-AS1 PLCD4 CAMK1 ALS2CL MXD4 TENT4A HSD17B4 ETF1 TMEM170B PSMB8-AS1 TAF11 TFEB DBNL PON2 BCAP29 WDR91 ERICH1 UBAP1 RUSC2 TOR1B FAM78A RBM17 HPS5 RASGRP2 SNX15 CABP4 ATG16L2 UBASH3B KLRK1 WBP4 ITM2B BCL2L2 TINF2 SPTSSA DGLUCY EVL KLC1 AKT1 SNX1 AC108134.3 DCTN5 TAOK2 GOSR2 REXO1 DDA1 COLGALT1 ARHGEF1 PPM1N TRPC4AP PLCG1 CD40 PPM1F PACSIN2 WWC3 ACOT9 CXorf38 |
| P6_I8_M11 | CTNNBIP1 MFSD2A FCGR3A TUBA4A AC010980.2 SLC25A20 ATP1B3 INTS12 SNX2 SRSF3 CPSF4 PPP3CC WDYHV1 NPDC1 PELI3 C1QTNF5 DDIT3 TOX4 ITPK1 EMP2 TRPV2 CORO6 TMEM104 ACAA2 ARHGAP45 RAB3D GMFG NAPSA TMEM150B DESI1 |
| P4_I22_M11 | HIVEP3 PGM1 ROR1 AL109741.1 CD101 TUFT1 CD244 FMO2 HSD11B1 SIPA1L2 CCDC85A CCDC141 ARHGEF3 FRMD4B NDNF HPGD GRIA1 KHDRBS2 SOSTDC1 AC006033.2 TRBJ2-4 SLC1A1 CDKN2B SPAAR SH2D4B NRG3 PAPSS2 TSPAN32 STX3 CTSW CASP12 APLP2 AMIGO2 LINC00551 GZMH ARHGAP27 SMAD7 ARID3A MKNK2 DNM2 PRX MAOA |
| P5_I9_M6 | PLCH2 WNT4 AL136985.3 CACHD1 MIR205HG EDARADD AC069404.1 AC073257.2 GPR87 TMEM144 PITX1 TPBG CD24 WIPF3 DTX2P1 AC011899.1 AGPAT5 AL445489.1 RET ALDH18A1 AC068896.3 RASSF10-DT PAK1 TENM4 AC023510.2 AL121820.2 CKMT1A MPV17L MEIS3P2 TMEM59L PTPRT CDH26 LINC02532 AC091214.1 CD24P4 |
