# Supplementary figures and images for: Integrated bulk and single-cell RNA-sequencing reveals SPOCK2 as a novel biomarker gene in the development of congenital pulmonary airway malformation
Source: Respir Res. 2023 May 10;24:127. doi: 10.1186/s12931-023-02436-z (PMC10170809; doi:10.1186/s12931-023-02436-z)

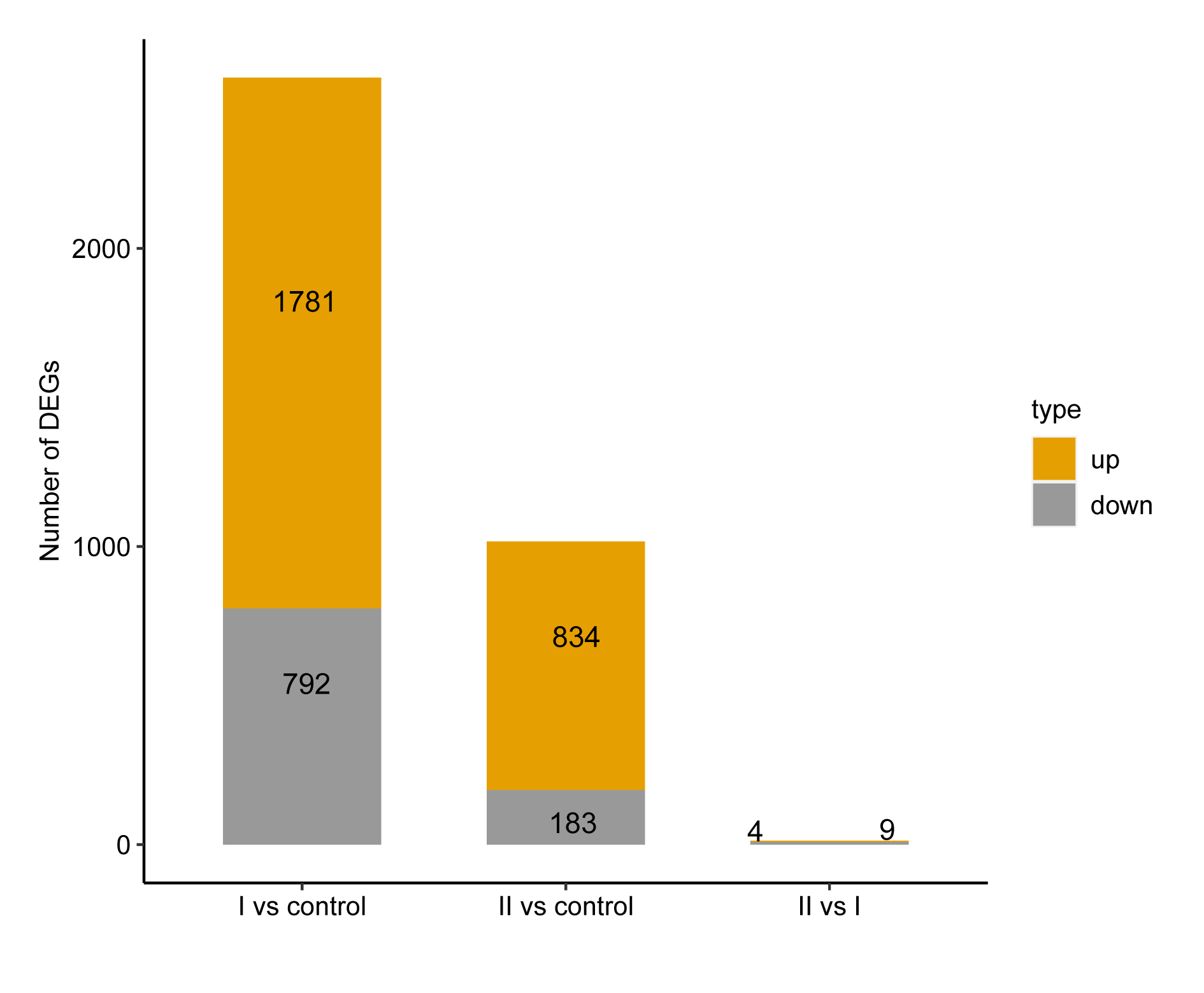

Supplement: Supplementary file 7 — Additional file 7: figure S1 The numbers of DEGs in different groups [file 12931_2023_2436_MOESM7_ESM.tif]

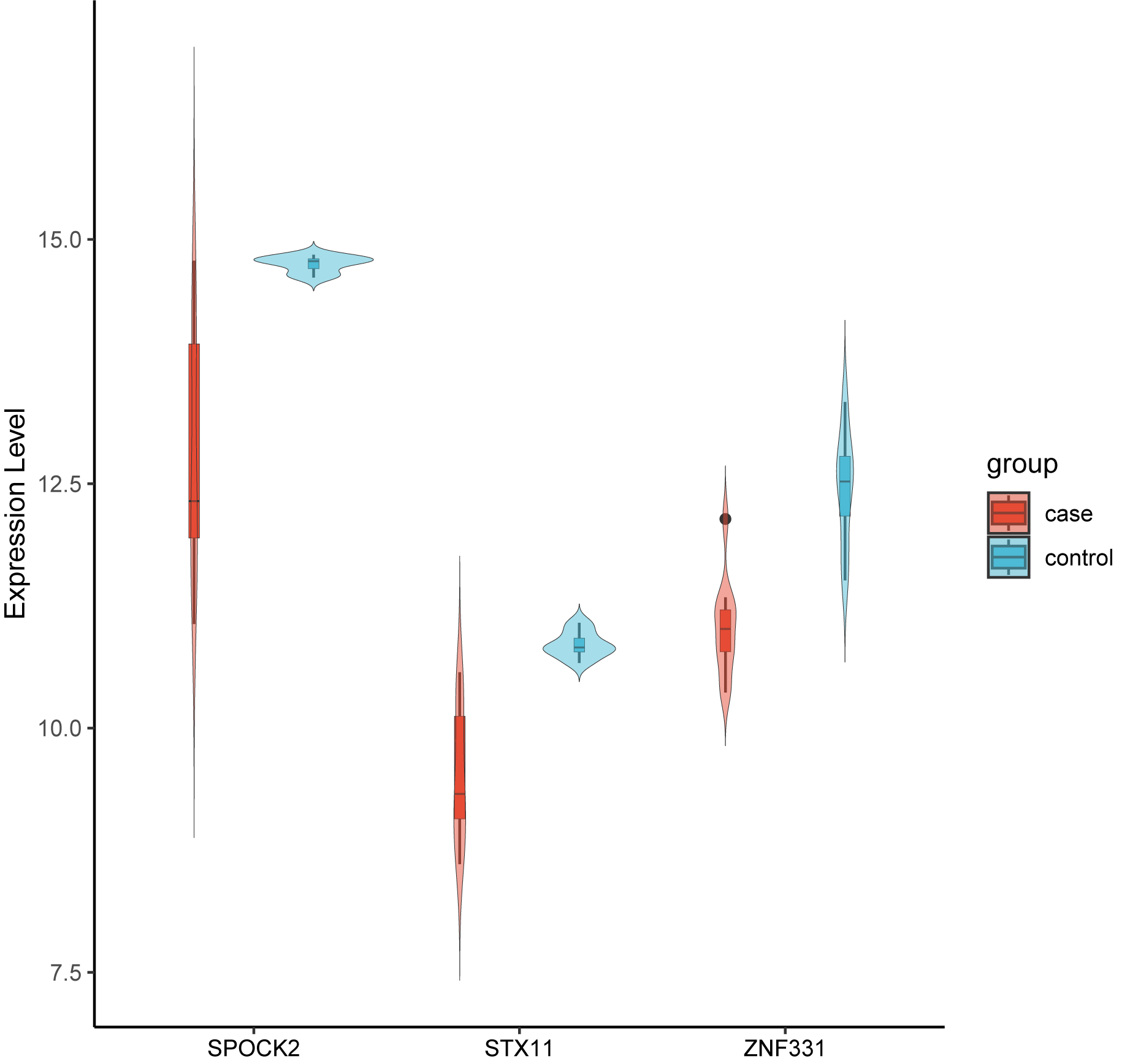

Supplement: Supplementary file 8 — Additional file 8: figure S2 Expressions of SPOCK2, ZNF331 and STX11 in RNA-seq data [file 12931_2023_2436_MOESM8_ESM.tif]

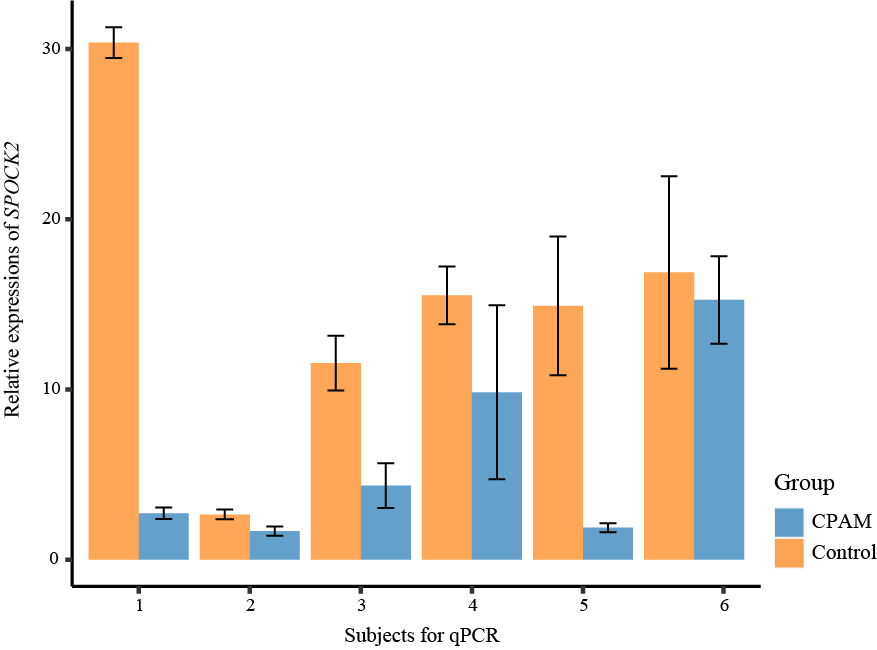

Supplement: Supplementary file 9 — Additional file 9: figure S3 Validation of SPOCK2 by qPCR analysis in samples of 6 CPAMs and 6 simply controls [file 12931_2023_2436_MOESM9_ESM.tif]
